# Functional Metabolomics Describes the Yeast Biosynthetic Regulome

**DOI:** 10.1016/j.cell.2016.09.007

**Published:** 2016-10-06

**Authors:** Michael Mülleder, Enrica Calvani, Mohammad Tauqeer Alam, Richard Kangda Wang, Florian Eckerstorfer, Aleksej Zelezniak, Markus Ralser

**Affiliations:** 1 Department of Biochemistry and Cambridge Systems Biology Centre, University of Cambridge, Cambridge CB2 1GA, UK; 2 The Francis Crick Institute, Mill Hill Laboratory, Mill Hill, London NW7 1AA, UK

**Keywords:** metabolism, amino acids, functional metabolomics, mass spectrometry, yeast gene deletion collection, target of rapamycin (TOR), vesicle mediated transport, functional gene annotation, unknown gene function

## Abstract

Genome-metabolism interactions enable cell growth. To probe the extent of these interactions and delineate their functional contributions, we quantified the *Saccharomyces* amino acid metabolome and its response to systematic gene deletion. Over one-third of coding genes, in particular those important for chromatin dynamics, translation, and transport, contribute to biosynthetic metabolism. Specific amino acid signatures characterize genes of similar function. This enabled us to exploit functional metabolomics to connect metabolic regulators to their effectors, as exemplified by TORC1, whose inhibition in exponentially growing cells is shown to match an interruption in endomembrane transport. Providing orthogonal information compared to physical and genetic interaction networks, metabolomic signatures cluster more than half of the so far uncharacterized yeast genes and provide functional annotation for them. A major part of coding genes is therefore participating in gene-metabolism interactions that expose the metabolism regulatory network and enable access to an underexplored space in gene function.

## Highlights

• One-third of coding genes significantly impact yeast biosynthetic metabolism
• The amino acid metabolome is most sensitive to chromatin and transport proteins
• TORC1 affects biosynthetic amino acid metabolism via vesicle-mediated transport
• Metabolic signatures are gene specific and cluster 3,923 genes according to function

## Graphical Abstract

**Figure:**
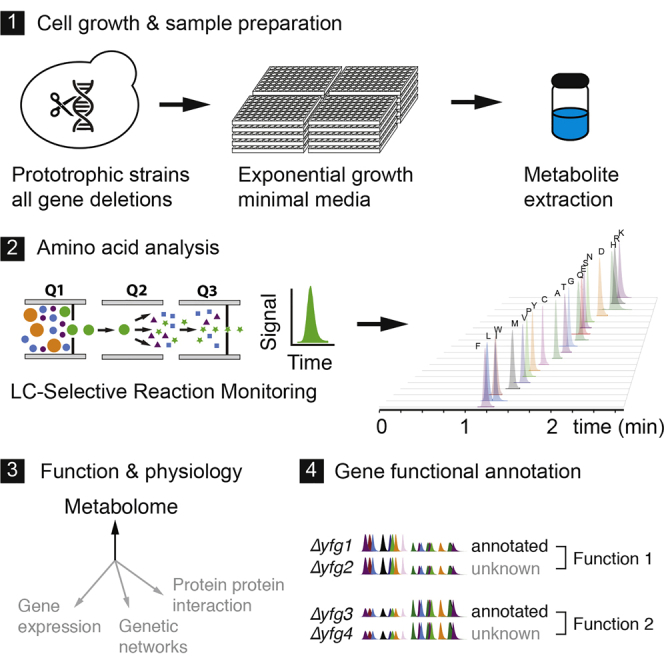

## Teaser

The metabolome of a yeast gene deletion collection connects metabolism to gene function.

## Introduction

Metabolic alterations play a key role in cancer, metabolic disease, pathogen infections, microbial communities, caloric restriction, and driving the aging process. Consequently, conserved metabolic regulators and sensors, including the target of rapamycin (TOR) pathway, have regained focus in medicine and biotechnology (Cornu et al., 2013). However, in the early days of molecular biology, the metabolic network was regularly regarded as a static, chemical “housekeeping” network (Wolstenholme and O’Connor, 1959). Metabolism consequently received limited attention in the context of gene regulatory networks, and many experiments in yeast and other organisms have been conducted under supplemented growth conditions that render large parts of biosynthetic metabolism artificially dispensable (Gibney et al., 2013, Mülleder et al., 2012). For these reasons, the genome-scale picture of the regulation of biosynthesis and the interactions between genome and metabolome is so far incomplete.

Accounting for a major fraction of the metabolic flux and up to three-quarters of the total mass of polar metabolites, amino acid biosynthesis, homeostasis, and catabolism constitute a quantitatively dominating component of the metabolic network (Nissen et al., 1997, Park et al., 2016). Amino acids serve as precursors for protein synthesis and as intermediates in nucleotide, cofactor, and lipid synthesis, carbon and nitrogen sensing and homeostasis, and redox buffering (Ljungdahl and Daignan-Fornier, 2012). To enable functional genomic approaches to study their metabolism, we and others have recently repaired amino acid auxotrophies in a *Saccharomyces cerevisiae* genome-scale knockout collection (Winzeler et al., 1999) that facilitates cell growth in the absence of amino acid supplements (Gibney et al., 2013, Mülleder et al., 2012). We here exploit targeted metabolomics to record precise amino acid concentration profiles for all of the strains and assemble the obtained information in a genome-scale gene-metabolism interaction map. By picturing the metabolic impact of transcription and signaling that operates predominantly at the chromatin level or via homeostatic feedback by metabolism-dependent systems, the ribosome, or protein transport, we achieve global insight into the regulation and homeostasis of metabolism during cell growth.

Gene deletions impact the amino acid metabolome with an unanticipated precision. This renders the absolute quantitative amino acid signatures informative about gene function, as they cluster genetic and pharmacological perturbations according to functional similarity on the genomic scale. We find that functional metabolomics provides orthogonal information in comparison to existing functional genomic data, in particular compared to physical and genetic interaction networks, and is found to be a rich resource to annotate so far uncharacterized genes.

## Results

### Gene Deletions that Impact the Biosynthetic Metabolome

**Figure 1:**
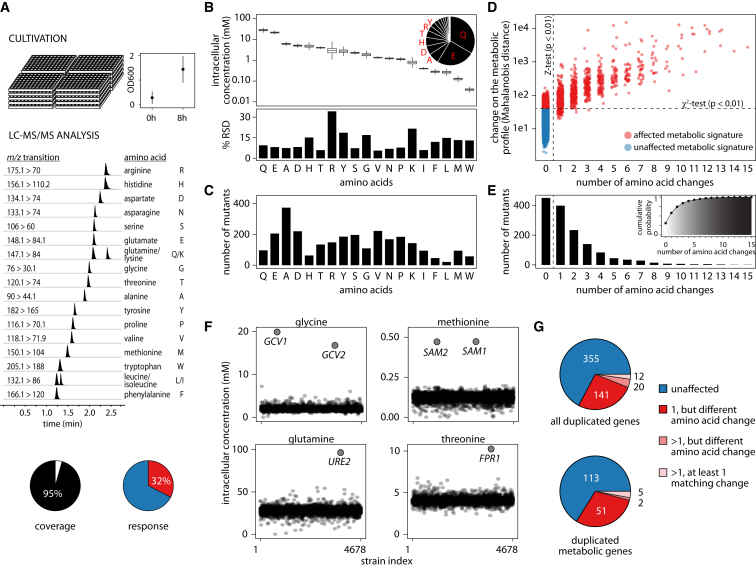
Genome-Metabolome Interactions that Affect Amino Acid Biosynthetic Metabolism (A) Amino acid quantification in a genome-scale collection of *Saccharomyces* gene deletion strains, grown in minimal synthetic medium, harvested in exponential phase (mean ± SD, n_t0_ = 92, n_t8_ = 83), and processed by hydrophilic interaction liquid chromatography (LC) selective reaction monitoring (SRM) for the precise quantification of amino acids. Middle: representative SRM chromatogram (scaled). Bottom: 95% of viable deletion strains (99% of strains growing in minimal medium) were successfully analyzed; 32% possess a significantly changed metabolome. (B) Amino acid concentrations span over three orders of magnitude and are affected by gene deletions between 6% and 36%. (C) Amino acid concentrations are sensitive for up to 373 gene deletions per metabolite. (D) 1,519 gene deletions significantly affect the amino acid profile as identified by multivariate statistics (χ^2^-test, adjusted p value < 0.01, 1,459 deletion strains) and univariate statistics (*Z*-test, adjusted p value < 0.01, 1,069 deletion strains). (E) Amino acid signatures are predominantly specific, expressed as simultaneously changing amino acid concentrations (bar chart) and as cumulative percentage (insert). 75% of metabolic profiles are characterized by two or fewer concomitant changes. (F) Examples of quantitatively high and uniquely specific gene-metabolite interactions. (G) Duplicated genes specialize in metabolic function. 161 out of 173 genes in homology clusters (53 out of 58 metabolic genes) trigger qualitatively different metabolic signatures.

**Figure S1:**
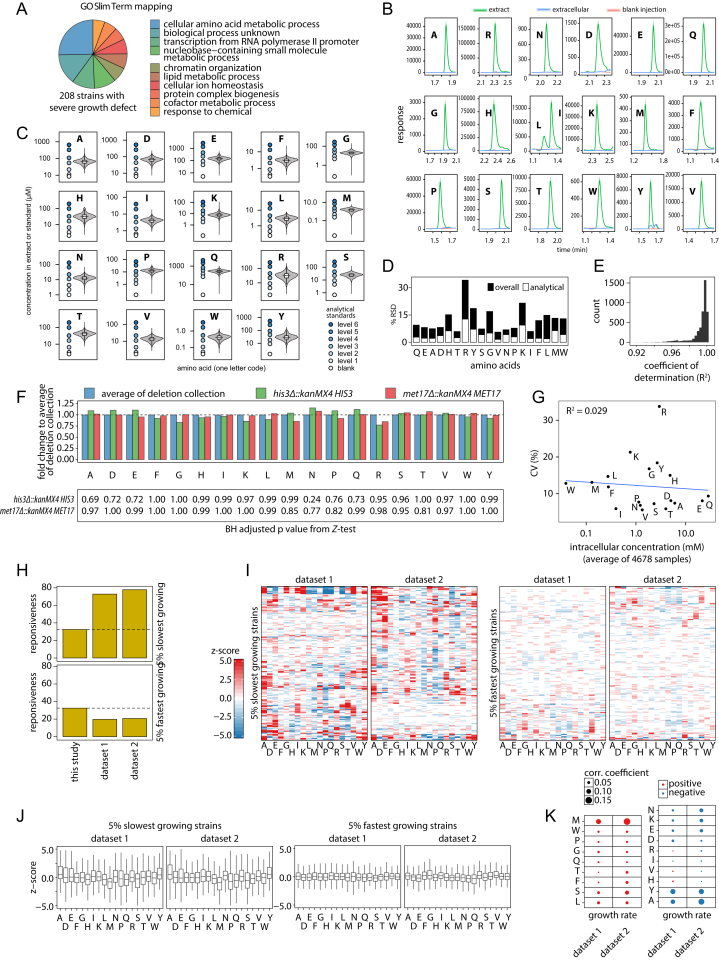
Properties of the Genome-Scale Metabolic Map of Amino Acid Metabolism, Related to Figure 1 (A) Strains inviable on SM medium are frequently deficient genes in biosynthetic metabolism and auxotrophs. The 10 quantitatively most enriched Gene Ontology (GO) biological process (Slim terms) are shown. (B) Extracellular amino acids are diluted by the protocol used, and do not interfere with reported quantities. A representative sample (n = 3) was grown to OD_600_ of 1.5, the cells were collected and the amino acid concentration determined after extraction with boiling ethanol for both cell pellet with residual medium (extract) and the supernatant containing only the extracellular metabolites (extracellular); line: average, ribbon: SD; red: blank measurement (no yeast present). (C) Linearity over the dynamic range of the amino acid metabolome. An analytical standard mix was prepared at 6 concentrations to cover the full dynamic range of physiological amino acid concentrations. The combined violin- and boxplot illustrate the determined amino acid concentration for the 4678 deletion strains before normalization; circles refer to the concentration of the standard mix. (D) Variation of acquired data as relative standard deviation (RSD). Overall variation (technical plus biological) was robustly estimated with the Minimum Covariance Determinant (MCD), the technical variation was calculated from a repeatedly analyzed quality control sample (n = 237). (E) Quality of external calibration with “bracketing” showing an R^2^ value greater 0.99 in 95% of 4518 curves. (F) The MCD center estimate for the average of all analyzed strains is identical to the WT amino acid profile; shown for the complemented deletion strains *his3Δ::kanMX4* and *met17Δ::kanMX4*. (G) The relative magnitude of genetic impact of the single gene deletions is independent of the concentration of the respective amino acid. (H) Strains with a significantly changed amino acid profile are overrepresented in 5% slowest growing and underrepresented in the 5% fastest growing mutants. (I) A growth specific amino acid profile was not detected for slow or fast growing strains. Amino acid profiles are ordered by hierarchical clustering (Euclidean distance, Ward’s criterion). (J) Among slow and fast growing strains we frequently find amino acids concentrated significantly lower and higher than average, confirming that there is no growth specific change in a certain amino acid. (K) Growth correlates weakly with amino acid concentrations (Spearman’s rank correlation coefficient). Growth datasets were obtained from previous systematic studies: dataset 1, growth determined on synthetic minimal medium of a prototrophic derivative of the deletion collection (VanderSluis et al., 2014); dataset 2, an auxotrophic derivative of the deletion collection tested in a competition assay in minimal dropout medium (Breslow et al., 2008).

4,913 gene-deletion strains that are viable in the absence of amino acid supplements (Figure S1A; Mülleder et al., 2012) were cultivated in synthetic minimal medium and grown to exponential phase, and their amino acid profile was determined by a precise, targeted analysis (Figure 1A). Glutamine was found to be the highest concentrated amino acid, being three orders of magnitude more abundant than the lowest concentrated amino acid, tryptophan (Figure 1B). The average amino acid concentration was sensitive to 163 gene deletions (median, *Z*-test, adjusted p value < 0.01), with alanine most frequently (431) and leucine least frequently affected (25) (Figure 1C). A combined multivariate (χ^2^-test based on robustly estimated Mahalanobis distance, adjusted p value < 0.01) and univariate statistic (*Z*-test, adjusted p value < 0.01) identified a total of 1,519 (32%) genes of significant impact (Figure 1D). The individual amino acid responded by 6%–34% in terms of relative SD (Figure 1B). The concentration of amino acids did not correlate with their variability (Figure S1G), with arginine being the most variable (34%) and serine (7%), threonine (6%), valine (6%), and isoleucine (6%) the most concentration stable (Figure 1B).

Amino acid signatures were not a consequence of growth rate (Figure S1I), as the average amino acid profile of slow- and fast-growing strains corresponded largely to that of an average strain (Figure S1J) and only a weak correlation of growth rate and individual concentrations was detected (absolute Spearman’s rank correlation coefficient < 0.15; Figure S1K). However, several of the 5% most slow-growing strains (Breslow et al., 2008, VanderSluis et al., 2014) possessed altered amino acid signatures (Figure S1H). Changes in amino acid metabolism caused by gene deletion hence manifest upstream of the growth rate.

Indeed, amino acid signatures were highly gene specific. 455 profiles were characterized by a single concentration change, while only 378 (25%) were characterized by more than two amino acid changes (Figure 1E). As extreme cases of specificity, we detect a genome-wide unique accumulation of glycine in the absence of the mitochondrial glycine decarboxylase subunits *GCV1* and *GCV2*, of methionine upon deletion of the S-adenosylmethionine synthetases *SAM1* and *SAM2*, of glutamine in yeast lacking the transcriptional regulator *URE2*, and of threonine in the absence of its feedback regulator, *FPR1* (Figure 1F).

The metabolic signatures of genes grouped according to sequence homology (Kuepfer et al., 2005) revealed metabolic specialization of typical paralogs. In 173 cases, solely one paralog altered amino acids, followed by 20 cases where each paralog created a specific signature. At least one amino acid was commonly affected in only 12 cases. Specialization is observed for all genes, as well as the subset of metabolic enzymes (Figure 1G).

### General Transcriptional Control and Protein Transport Have the Strongest Metabolic Impact

**Figure 2:**
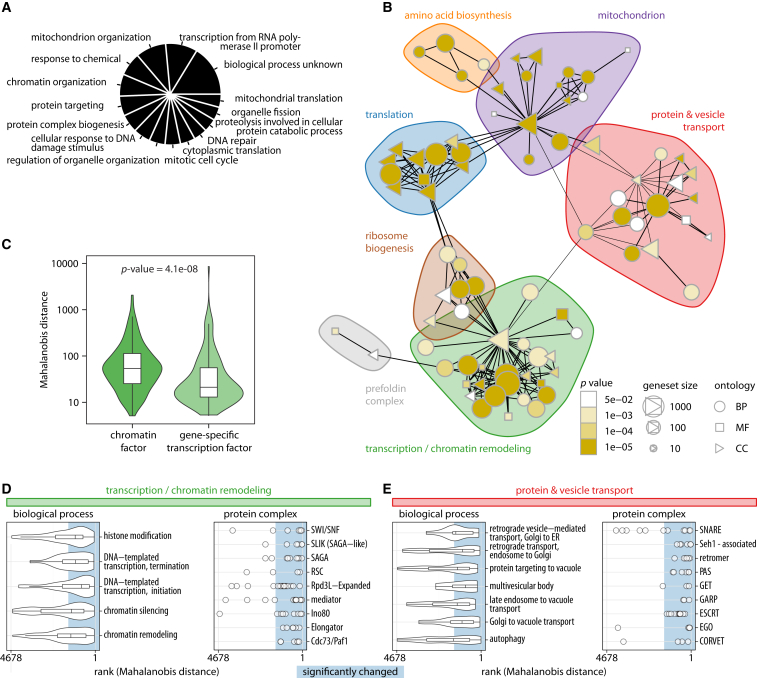
Chromatin and Intracellular Transport Dominate Strong Metabolic Signatures (A) Top 15 Gene Ontology (GO) slim categories of gene deletions affecting the biosynthetic metabolome. (B) Functional “perturbation network” constructed on the basis of a gene set enrichment analysis (GSEA). Each node represents an enriched GO term (adjusted p value < 0.05), which is connected when the gene overlap is >50%. Edges are drawn for shortest paths of one or two; distinct groups were identified by clustering of the edge betweenness. BP, biological process; MF, molecular function; CC, cellular component. A fully labeled version is given in Figure S2. (C) Chromatin- and histone-modifying proteins, as part of the general gene expression machinery, have a significantly stronger impact than gene-specific transcription factors (Wilcoxon rank-sum test). (D) Protein complexes associated with general transcriptional control at the chromatin level trigger strong metabolic signatures (4,678: most similar to wild-type; 1: most different to wild-type). (E) Protein complexes associated with vesicle-mediated transport processes that are responsible for strong metabolic signatures (4,678: most similar to wild-type; 1: most different to wild-type).

**Figure S2:**
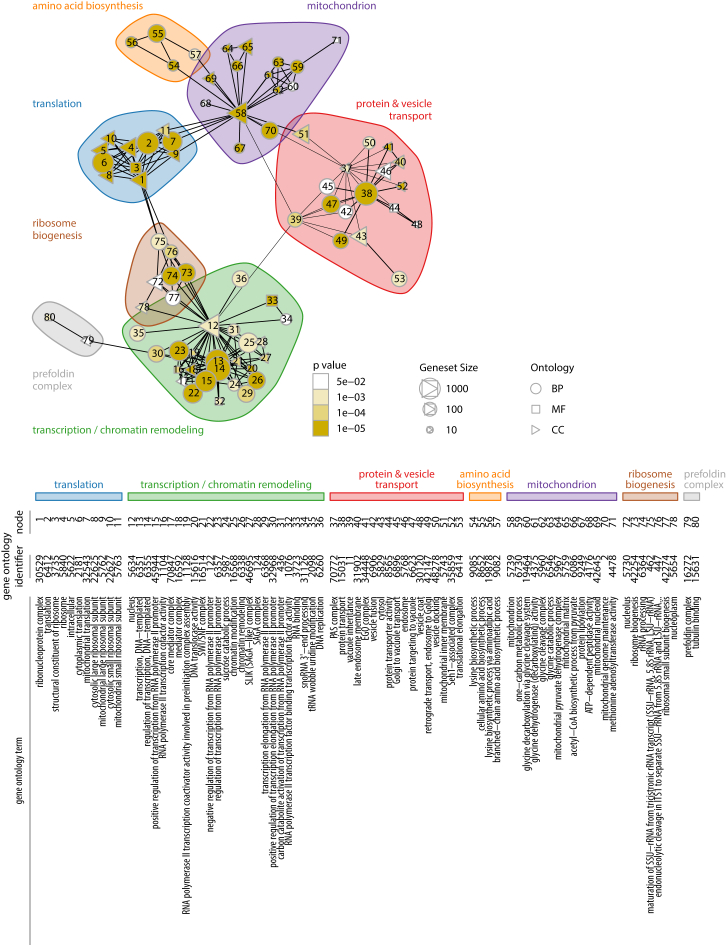
Identification of Cellular Processes and Components Important for Amino Acid Homeostasis over a Gene Set Enrichment Analysis, Related to Figures 2B–2E and Table S1 Comprehensive labeling and complete probabilistic amino acid profiles for the directly associated and enriched GO terms among significantly changed deletion strains. BP, biological process; MF, molecular function; CC, cellular component.

Genes functioning in transcription, chromatin biology, translation, protein transport, and mitochondrial biology were found to be responsible for most amino acid concentration changes in an analysis for Gene Ontology (GO) slim terms (Figure 2A). As these groups constitute large gene classes, we continued to test for specific enrichments in a gene set enrichment analysis (GSEA) (Väremo et al., 2013) based on GO term associations stored in the *Saccharomyces* genome database (Cherry et al., 2012). 523 of 6,806 GO functional categories were significant enriched (adjusted p value < 0.05; Table S1). 80 directly associated GO terms were assembled in a metabolic “perturbation network,” which connects the terms according to genes that overlap (Figure 2A). Genes participating in “translation,” “protein and vesicle transport,” “amino acid biosynthesis,” the “mitochondrion,” “ribosome biogenesis,” and “transcription/chromatin remodeling” alter metabolic profiles most frequently (Figures 2B and S2; Table S1). The network reveals a close association of amino acid biosynthesis and chromatin remodeling (Figure 2B). This is in contrast to gene-specific transcription factors (TFs) (Kemmeren et al., 2014) that were not enriched in this analysis and found to create smaller perturbations in a direct comparison (Figures 2C and 2D). Ranking of all gene deletions according to their metabolic impact highlights the dominating role of histone modifications and the implicated protein machinery (SWI/SNF [SWItch/Sucrose Non-Fermentable], RSC [remodel the structure of chromatin], INO80, SAGA [Spt-Ada-Gcn5-acetyltransferase], and SAGA-like as well as the mediator, Cdc73/PAF1, and elongator complexes) (Figure 2D). Only intracellular transport had a comparably strong metabolic impact. This process is required for the recycling of amino acid by protein degradation and their intracellular transport. The strongest profiles are triggered by Golgi associated retrograde protein (GARP), Golgi to ER traffic (GET), and endosomal sorting complex required for transport (ESCRT) complexes important for trafficking, class C core vacuole/endosome tethering (CORVET) and SNARE proteins important vesicle fusion, as well as the PIKfyve/ArPIKfyve/Sac3 (PAS) complex required for phosphatidylinositol 3-phosphate synthesis (Figure 2E).

### The TORC1 Network, but Not the *TOR1* Kinase, Dominates Metabolic Signaling under Exponential Growth

**Figure 3:**
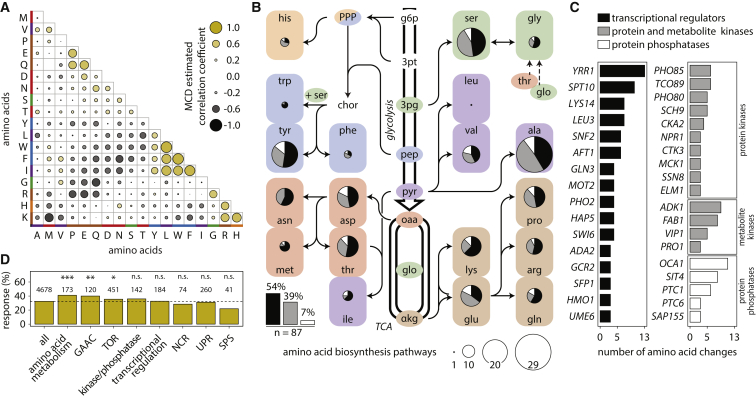
Amino Acid Concentrations Correlate on the Genomic Scale and Depend on Genetic Components of the Transcriptional and Posttranscriptional Regulatory Machinery (A) Correlation analysis of amino acid levels, color-coded according to biosynthetic precursor (illustrated in B). (B) Amino acid biosynthetic pathways, colored according to the origin of their carbon backbone. Superimposed are the regulatory mutants that significantly alter the concentrations (adjusted p < 0.01). Pie charts indicate the relative contribution of TFs, kinases, and phosphatases; the size is scaled to total numbers (Table S2). (C) Transcriptional and phospho-signaling mutants ranked by broadest metabolic impact (≥3), defined by simultaneously changing metabolites (adjusted p < 0.01). (D) General regulatory networks and cellular processes tested for enrichment (GSEA). Abbreviations: PPP, pentose phosphate pathway; TCA, tricarboxylic acid cycle; g6p, glucose 6-phosphate; 3pt, 3-phosphotrioses; 3pg, 3-phosphoglycerate; pep, phosphoenolpyruvate; oaa, oxaloacetate; glo, glyoxylate; αkg, α-keto-glutarate; amino acids in three letter code; ^∗^p < 0.05, ^∗∗^p < 0.01, ^∗∗∗^p < 0.001, Benjamini-Hochberg (BH) adjusted.

On the genomic scale, threonine, alanine, serine, and valine change independently to other metabolites (Figure 3A), but the remaining amino acids could be assigned to groups that reflect shared biological properties. Correlated amino acids originate from a common precursor (e.g., branched chain or aromatic amino acids), share chemical similarity and function as nitrogen sensors and donors (glutamine, glutamate), or share their principal storage compartment, such as the positively charged arginine, histidine, and lysine. Furthermore, branched-chain and aromatic amino acids, which share with the Ehrlich pathway the same catabolic system, were correlated (Figure 3A).

Indicative of co-regulation through the shared pathways, we addressed the function of transcription factors and kinases. Despite not being enriched as a GO category (Figure 3), 8% of altered metabolic signatures are attributed to the deletion of individual signaling kinases (51), phosphatases (11), and transcription factors (62) (*Z*-test, adjusted p value < 0.01). While many amino acids were dependent on both signaling and transcription factors, tryptophan was almost exclusively affected by transcriptional regulators, while two-thirds of genes affecting glutamate levels were kinases and phosphatases (Figure 3B; Table S2). Both transcriptional regulation and post-transcriptional regulation are hence important to maintain the amino acid metabolome, but their relative contribution differs between metabolites.

TFs, kinases, and phosphatases were then ranked to identify the most severe perturbations, which confirmed the importance of chromatin remodeling (*SPT10*, *SNF2*, *UME6*, and *ADA2*) (Figure 3C) and highlighted the importance of sensing nitrogen (*GLN3*), phosphate (*PHO85*, *PHO80*, *PHO2*, *VIP1*, and *ADK1*), and iron (*AFT1*), as well as the regulation of carbohydrate and energy metabolism (*GCR2*, *HAP5*, and *ELM1*). Of note, some of the key transcriptional regulators active under starvation and stress were only of moderate importance under the exponential growth conditions studied. This includes the unfolded protein response (UPR), the Ssy1p-Ptr3p-Ssy5p (SPS)-sensor systems, and *GCN4* of the general amino acid control (GAAC) pathway, which under nutrient limitation affects all amino acid pathways except cysteine (Natarajan et al., 2001) (Figure 3D).

A similar result was obtained for the *TOR1* kinase. Despite the deletion of *TOR1* causes a strong amino acid signature under starvation (Zhang et al., 2011), under exponential growth, its metabolic impact was not significant. However, in a network analysis, TORC1 signaling was significantly overrepresented (Figure 3D). Deletion of the TORC1 subunit *TCO89*, the downstream kinases *SCH9* and subunits *SIT4* and *SAP155* of the PP2A-related protein phosphatase complex, *NPR1*, and the transcription factor *SFP1* (Conrad et al., 2014, Loewith and Hall, 2011) (Figure 3C), all triggered strong and characteristic amino acid signatures. The metabolic signatures attribute particular importance to metabolic signaling kinases, which, as exemplified by *FAB1* that generates phosphatidylinositol 3,5-bisphosphate (Yamamoto et al., 1995), were characterized by stronger profiles as protein kinases (Figure 3C).

### The Metabolic Signature Connects the Dominating TORC1 Function to Endomembrane Transport

**Figure 4:**
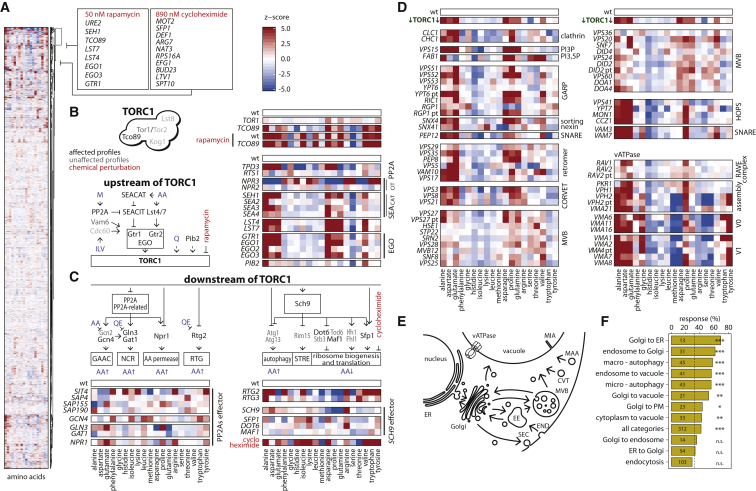
Amino Acid Signatures of TORC1 Inhibition during Exponential Growth Reflect an Interruption of Vesicle-Mediated Transport (A) Metabolic signatures, co-clustered on the genome scale, associate rapamycin with the TORC1 complex and cycloheximide with ribosome biogenesis (see Data S1 for the full map). (B) The deletion of the TORC1 subunit *TCO89* and TORC1 upstream regulators creates a metabolic signature matching rapamycin treatment; the deletion of TORC1 inhibitors mirrors this concentration profile. (C) Neither individual perturbation of TORC1 downstream effectors nor translation inhibition can explain the amino acid profile of TORC1 inhibition. (D) Perturbation of the vesicle-mediated transport machinery at the endosome triggers a metabolic signature that matches TORC1 inhibition (green, average profile of the TORC1 network). (E) Scheme: vesicle-mediated protein transport pathways in the endomembrane system. (F) Vesicle-mediated transport and the endomembrane system are crucial for maintaining amino acid homeostasis under exponential growth. Golgi to ER transport has the highest relative response rate, followed by endosome to Golgi and endosome to vacuole transport. ^∗^p < 0.05; ^∗∗^p < 0.01; ^∗∗∗^p < 0.001 (GSEA). Abbreviations: EE, early endosome; MVB, multivesicular body pathway/late endosome; CVT, cytoplasm to vacuole trafficking; MIA, micro-autophagy; MAA, macroautophagy; END, endocytosis; SEC, secretory vesicles; pt, partial deletion of gene.

**Figure S3:**
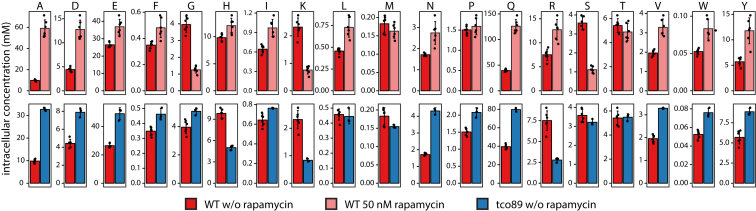
Reduced TORC1 Activity Produces a Specific and Informative Amino Acid Profile, Related to Figure 4 The application of sublethal rapamycin concentration (50 nM) generates a strong response on all amino acid concentrations. Alanine, aspartate, asparagine, glutamate, glutamine, branched chain amino acids and aromatic amino acids were substantially elevated whereas glycine, serine and lysine were greatly reduced and this response was strikingly similar to a deletion of the TORC1 component *TCO89*. All strains were grown for 6 hr and collected in exponential phase. Amino acid concentrations were determined as described for untreated WT and *tco89*Δ (n = 3) and for treated WT (n = 6) and are represented as mean ± SD.

We treated yeast with rapamycin to inhibit TORC1 pharmacologically and obtained a strongly altered amino acid signature, characterized by an increase in alanine, aspartate, asparagine, glutamate, glutamine, branched-chain amino acids, and aromatic amino acids as well as reduction in glycine, serine, and lysine (Figures 4A and S3). We mapped this profile to the genome-scale data and identified the Δ*tco89* profile to be of close resemblance (Figure 4A; Data S1, hierarchical cluster analysis). Revealing the metabolic signature to be a direct consequence of TORC1 inhibition, rapamycin treatment marginally further changed the profile of the *Δtco89* strain (Figure 4B).

We corroborated the metabolic effects by perturbation of TORC1 activating proteins. This includes the exit from G_0_ (EGO) complex (*EGO1*, *EGO2*, *EGO3*, and *GTR1*) and the GTPase activating proteins for Gtr2p (*LST4*/*LST7*) (Figure 4B). Also, the absence of other activators, such as the Seh1-associated activating TORC1 signaling (SEACAT) complex, the type 2A phosphatase scaffold protein *TPD3* (Sutter et al., 2013), or the activator *PIB2* (Kim and Cunningham, 2015) resulted in a matching signature (Figure 4B). Conversely, the deletion of *NPR3* of the Seh1-associated complex TORC1-inhibiting (SEACIT) complex (Neklesa and Davis, 2009) mirrored the metabolic profile (Figure 4B). Hence, the perturbation of TOR upstream activators mimics the metabolic signatures of rapamycin and *TCO89* deletion, while the deletion of inhibitors causes opposing metabolic defects.

Next, we exploited the metabolic signatures to identify the causative downstream processes. The metabolic signature of the major TORC1 effector pathways through *SCH9* (Urban et al., 2007) and type 2A and type 2A-related phosphatases (Düvel et al., 2003) and the inhibition of translation with cycloheximide did not match the metabolic profile of TORC1 inhibition (Figures 4A and 4C). Instead, a comprehensive set of genes required for protein transport through the endomembrane system revealed similar metabolic defects. This included retrograde trafficking from endosome to Golgi and Golgi to ER, the multivesicular body (MVB) pathway, the machinery essential for vesicle fusion (homotypic fusion and vacuole protein sorting (HOPS)/CORVET complexes), and subunits of the vacuolar ATPase (Figures 4D and 4E). 69.2% and 61.3% of mutants associated with Golgi to ER and endosome to Golgi transport (GO terms) showed altered amino acid levels. In contrast, mutants in ER to Golgi and Golgi to endosome transport, which are also not sensitive to TORC1 inhibition (Thomas et al., 2014), were underrepresented (Figure 4F). The closest resemblance to TORC1 inhibition was obtained for the endosomal transport processes, in particular when interrupting the Golgi-associated retrograde protein (GARP) complex, the retromer complex, and the sorting nexins Snx4p and Snx41p (Figure 4D). Indeed, a TORC1-like signature was triggered by all subcomplexes of the ESCRT apparatus and its associated genes, *DOA1* and *DOA4*. Furthermore, the TORC1 signature was shared upon deletion of the SNARE protein Pep12p and the tethering factor Vps3p (Figure 4D). Finally, despite slow growth, yeast lacking the clathrin light or heavy chain genes (*CLC1*, *CHC1*) and yeast with a deficiency in the integral membrane lipids phosphatidylinositol 3-phosphate (PI3P) and phosphatidylinositol 3,5-bisphosphate (PI(3,5)P_2_) by deletion of *VPS15* and *FAB1*, respectively, had matching profiles. Overall, similar but quantitatively stronger changes were obtained upon perturbation of the vesicle fusion machinery located at the vacuole. This includes the HOPS complex, the t-SNARES *VAM3* and *VAM7*, and the vacuolar ATPase, of which our screen contained the regulatory components *RAV1* and *RAV2* and the assembly factors *PRK1* and *VPH1*. Furthermore, despite excluding mutants for the V_0_ and V_1_ ATPase subunits and assembly factors *VPH2* and *VMA21* from the genome-scale analysis due to strong growth defects, their profiles matched too (Figure 4D). However, other processes across the genome did not match these metabolic changes, implying that TORC1 function in exponentially growing cells connects to amino acid metabolism via its role in the endomembrane transport system.

### Metabolic Signatures Provide Orthogonal Functional-Genomic Information

**Figure S4:**
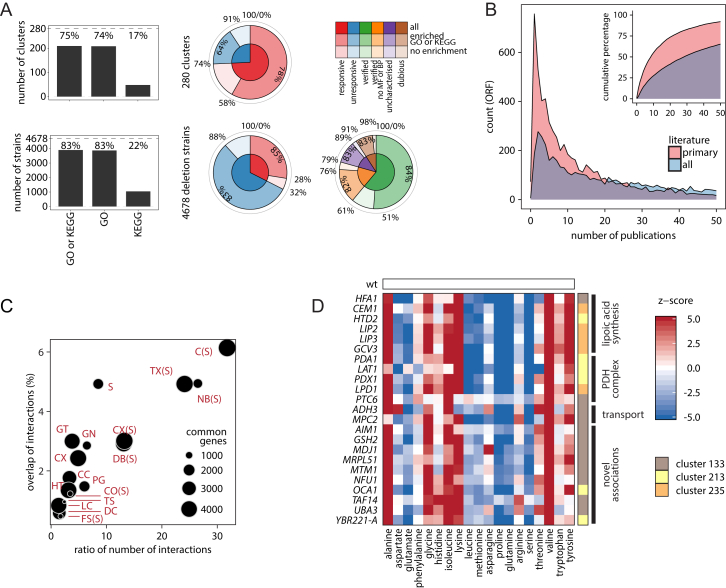
Metabolic Signatures Add New Functional Information to Incompletely Characterized *S. cerevisiae* Genomes, Related to Figure 5 (A) (top, left panel) Functional enrichment analysis in 280 clusters for GO and KEGG terms. Enrichment was comparable in clusters with at least one significantly changed amino acid (responsive) or in clusters without (unresponsive), reflecting that small co-occurring amino acid concentration changes reveal functionally relevant signature (top, middle panel); functional enrichment analysis as described in STAR Methods. For 83% of gene deletions strains, a functional association was predicted. (bottom panel) 39% percent of deletion strains are not well characterized according to our definition (no GO or function or process term associated, annotated as ‘uncharacterized’). A functional association is possible for 83% of them. (B) To quantitatively enumerate the occurrence of so far poorly and uncharacterized genes in budding yeast, we orient on the most comprehensive collection of functional information in budding yeast, the *Saccharomyces* genome database, which combines large-scale, small scale, literature mining and community-driven approaches (Cherry et al., 2012). Primary literature contains information about “function, biological role, cellular location, phenotype, regulation, structure, or disease homologs in other species for the gene or gene product.” All literature further incorporates reviews and publications with “experimental evidence for the gene or describe homologs in other species, but for which the gene is not the paper’s principal focus.” 26.7% of yeast ORFs have so far been analyzed in only three to zero dedicated studies, 22% of open reading frames in SGD are considered functionally fully uncharacterized according to systematic annotation, while 35.8% lack so far a gene ontology function or process term association. Genes that are annotated as uncharacterized or lack a GO annotation sum up to 1823 (39%) of the strains analyzed in our study. Orthogonality of functional metabolomics to other molecular data. Abbreviations are as described in YeastNet resource, for every network links are inferred from a different source, as following: CC - co-citation of two genes across 46,111 PubMed Medline article abstracts for yeast biology; CX - co-expression pattern of two genes (based on high-dimensional gene expression data); DC - co-occurrence of protein domains between two coding genes; GN - similar genomic context of bacterial orthologs of two yeast genes; GT - similar profiles of genetic interaction partner; HT - high-throughput protein-protein interactions; LC - small/medium-scale protein-protein interactions (collected from protein-protein interaction databases); PG - similar phylogenetic profiles between two yeast genes; TS - 3-D protein structure of interacting orthologous proteins between two yeast proteins. The rest interaction networks based on evidences collected in STRING database, i.e., DB(S) - based on pathways from curated databases; NB(S) - interactions inferred from genomic neighbors; CX(S) - interactions inferred using gene co-expression; CO(S) - gene co-occurrence in phylogenetic trees; TX(S) - interactions based on automated unsupervised text mining when searching for proteins that are frequently mentioned together; F(S) - genes that are sometimes fused together; C(S) - based on all combined evidences in STRING. (C) Perturbation of lipoic acid synthesis or the pyruvate dehydrogenase reaction triggers a highly similar amino acid profile and allows to associate a novel gene function to cluster members. Clusters 133, 213 and 235 are joined at greater branch height and form a cluster separated from the remaining deletion strain profiles (Data S1). The deleted ORF GO, gene ontology; KEGG, Kyoto Encyclopedia of Genes and Genomes; BP, biological process; MF, molecular function.

Metabolic signatures were sufficiently specific to genomically cluster rapamycin to TORC1 and cycloheximide to translation (Figure 4A) and hence imply a potential for identifying drug targets and for functional annotation. The 4,678 metabolic profiles were analyzed by robust consensus clustering (Monti et al., 2003) using pairwise Mahalanobis distances and an optimal cluster division calculated by adaptive branch pruning (Langfelder et al., 2008). 75% of 280 obtained clusters (Figure 6A; Table S1) were statistically enriched for GO (74%) or Kyoto Encyclopedia of Genes and Genomes (KEGG) (17%) functional terms (Figure S4A). Propagating the cluster enrichment to its members, 3,923 genes (83%) of gene deletions strains were associated with functional terms. The clustering was efficient for genes possessing both strong and weak amino acid signatures and is hence not a function of the strength of metabolic impact, as long as metabolic changes are detectable (Data S1).

**Figure 5:**
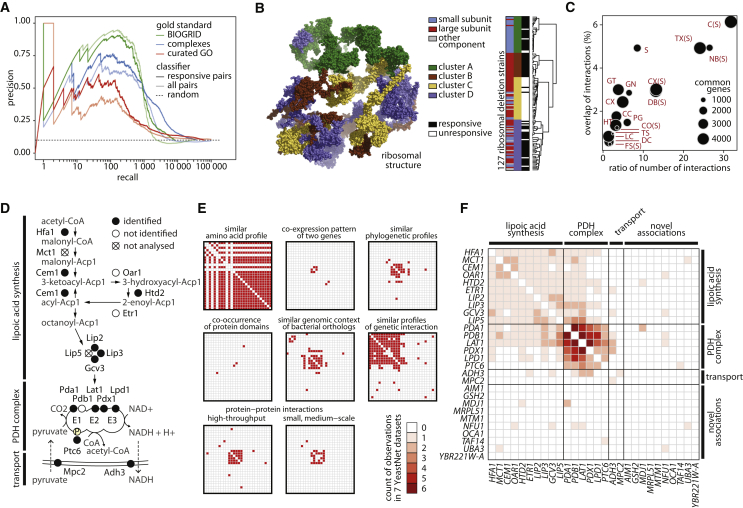
Amino Acid Signatures Are Informative about Gene Function and Provide Orthogonal Information to Other Molecular Networks (A) Precision-recall analysis evaluating metabolite profiles in comparison to curated protein complexes, low-throughput genetic and physical protein-protein interactions, and GO terms. The different gold standards are color coded and the set of metabolic signatures distinguished by transparency; the dashed line corresponds to the random classifier. (B) Metabolic signatures reflect structural proximity of the 127 non-essential ribosomal proteins, mapped to the ribosomal structure (PDB: 4V8Y) (Fernández et al., 2013). (C) Orthogonality of functional metabolomics to other molecular data. Metabolic signatures were assembled in a phenotypic association network and compared to STRING and YeastNet functional genomic datasets. y axis is the overlap of edges between the genes (nodes) present in the compared networks, whereas the x axis is the ratio of edges, comparing the STRING or YeastNet, to the metabolic clustering. Circle size indicates the number of genes compared. Abbreviations as in YeastNet (given in full in Figure S4C). Metabolic signatures provide highly orthogonal information, and the best agreement is obtained upon combining all data as contained in STRING (C(S)). (D) Metabolic signatures provide orthogonal information to existing molecular networks, as exemplified by the lipoic acid (LA) biosynthesis pathway, which provides the cofactor for the pyruvate dehydrogenase (PDH) complex. Scheme of the pathway is shown. (E) Association of metabolic signatures (LA and PDH cluster), in comparison to yeast genetic and physical interaction networks as accessed through YeastNet. A majority of the associations among the LA biosynthetic genes are obtained only over the metabolic signature. (F) Summarizing the information content of all molecular networks (E), except metabolic signature, in comparison to the association obtained over the metabolic signature (full matrix). When combined, genetic and physical interaction networks capture the PDH complex but provide only partial coverage of the LA pathway.

As no “gold standard” dataset for evaluating metabolism-driven gene annotation is available, we tested our dataset in respect to protein complexes (Complex Enrichment Analysis Tool [COMPLEAT]; Vinayagam et al., 2013), low-throughput genetic and physical protein-protein interactions (Chatr-Aryamontri et al., 2015), and curated gene ontologies (Myers et al., 2006). The gene pairs were ranked by their probability to co-cluster (consensus index) and subjected to a precision-recall analysis. At a true-positive recall of 1,000 gene pairs, we obtained a precision, the ratio of true positives (TP) over true and false positives (TP + FP), that was 4.8 times, 4 times, and 2 times higher than by random expectation (10%), respectively (Figure 5A). Restricting gene pairs to those showing significant concentration changes, we recover 5,000 gene pairs with a precision 2.2 times better than at random. Keeping in mind that metabolic profiles associate genes according to phenotype and are by nature different in terms of physical interactions, this result revealed a substantial recall of existing knowledge. To illustrate the potential of the approach, we cluster the 127 non-essential ribosomal genes and obtain a correct subunit allocation (Figure 5B).

We then questioned to which extent functional metabolomics would provide new information compared to existing physical, and genetic interaction networks. We compared a graph constructed from the metabolic clusters, to the comprehensive sets of functional genomic networks as stored in Search Tool for the Retrieval of Interacting Genes/Proteins (STRING) (Szklarczyk et al., 2015) and YeastNet (Kim et al., 2014) databases. We found that metabolic signatures provide highly orthogonal information (Figure 5C). The maximum overlap in connectivity was 0.06 and was obtained upon combining all STRING networks (Figure 5C). The relationship between metabolic-signature-based association and the preexisting functional genomic data is illustrated for the lipoic acid (LA) biosynthetic pathway, which has recently been solved by classic biochemical experiments (Cronan, 2016). The metabolic pathway, plus its association with the PDH complex that requires lipoic acid as cofactor, are comprehensively captured over their metabolomic signature (Figures 5D and 5E; Table S3). The other datasets fully capture the pyruvate dehydrogenase (PDH) complex, but despite being much more comprehensive, they largely failed to connect it to the LA pathway (Figures 5E and 5F).

**Figure 6:**
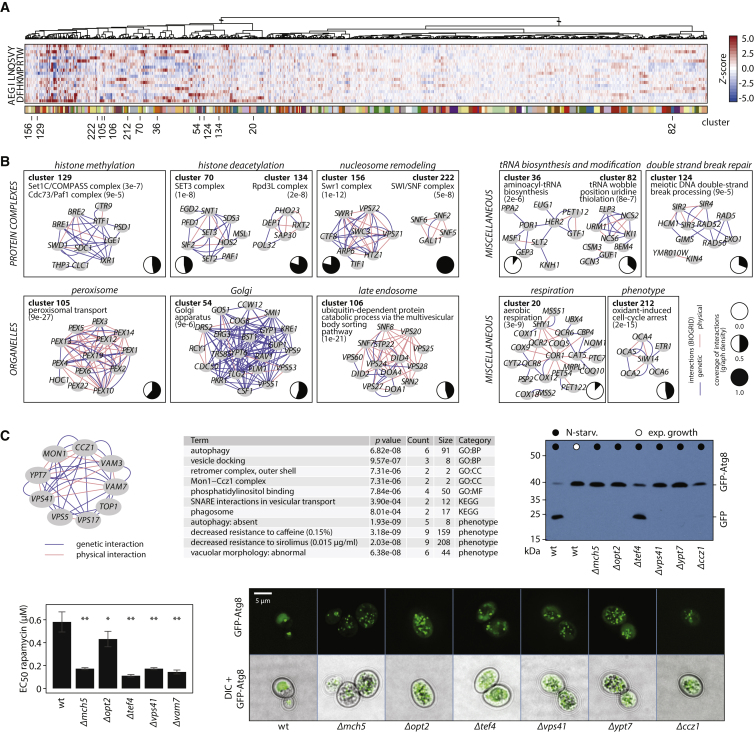
Association of Gene Function through Functional Metabolomics (A) Metabolic signatures divide the genome according to function. The full heatmap detailing the hierarchical cluster analyses spans over 280 clusters (Data S1; 47 pages); 75% of the clusters, spanning over 3,923 gene deletions, are significantly enriched for functional GO and KEGG terms. (B) Gene function in biological processes is captured by metabolic signature similarity (ten examples are given here; all clusters in Data S1 and Table S3). Yeast genetic and physical interaction networks (BIOGRID) support these clusters to different extents (coverage of 42%–100% of nodes and 10%–100% of edges; graph density, pie charts). (C) Phenotypes of gene deletion strains (top left) confirm new associations as in cluster 135, representing vesicle fusion at the vacuole (top middle). The gene-deletion strains are characterized by autophagy deficiency upon nitrogen starvation, visualized by a GFP-ATG8 cleavage deficiency (top right), sensitivity to rapamycin as expressed in a dose-response curve (bottom left) (mean ± SD, n = 4), and abnormal vacuolar morphology upon 6-hr nitrogen starvation (bottom right). ^∗^p < 0.05; ^∗∗^p < 0.01; ^∗∗∗^p < 0.001 (Student’s t test).

Next, we illustrate ten unrelated cellular processes captured over their metabolic signature and compare the associations with physical and genetic interaction data (Figure 6B). Functional metabolomics captured transcriptional regulation via chromatin, including histone methylation (cluster 129), deacetylation (clusters 70 and 134), and nucleosome remodeling (clusters 156 and 222), organellar biology (cluster 105, peroxisome; cluster 54, Golgi; cluster 106, the ESCRT machinery), tRNA biosynthesis and modification (clusters 36 and 82), double-strand break repair (cluster 124), respiratory metabolism (cluster 20), and the barely understood phenotype of oxidant-induced cell-cycle arrest (*OCA2-5* genes, cluster 212). Genes within these clusters are assembled in a fully connected graph (graph density of 1) for a comparison to the existing networks. We reveal a coverage of 10%–100% of the metabolically derived connections, which illustrates the robustness of the approach as well as its orthogonal information content (Figure 6B).

**Figure S5:**
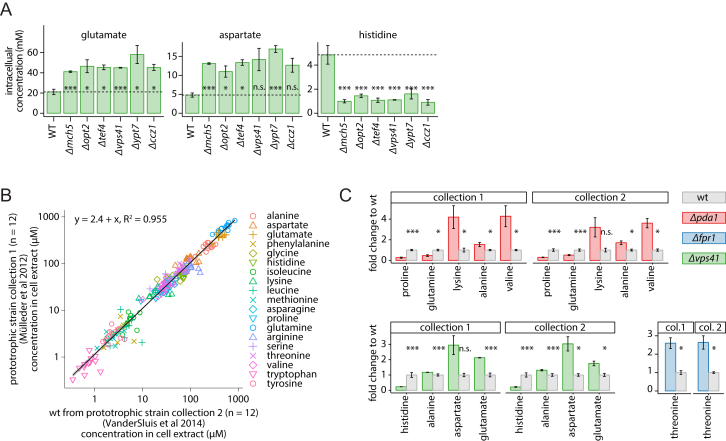
Metabolic Signatures and Gene Function Associations, Related to Figure 6 (A) Amino acid concentration levels determined for selected deletion strains required for vesicle fusion at the vacuole (mean ± SD, n = 3). (B) Confirmation of observed free amino acid levels in two different prototrophic derivatives of the deletion collection: Collection 1 was created by transformation with a minichromosome carrying the genes *HIS3*, *LEU2*, *URA3* and *MET17* (Mülleder et al., 2012), collection 2 used the synthetic genetic array strategy and the magic marker (*can1Δ::STE2pr-SpHIS5*) resulting in the genotype *can1Δ::STE2pr-SpHIS5 lyp1 his3 ho−* (Gibney et al., 2013, VanderSluis et al., 2014). Both collections are derived from the BY4741 deletion collection (Winzeler et al., 1999), have the mating type *MAT*a and contain a complemented *his3* deletion strain representing the isogenic reference wt strain. (left panel) The amino acid levels of the WT strains (n = 12) are compared. Analysis by linear regression, slope of 1 and an R^2^ value of 0.955, indicate that there is no difference between the respective profiles. (C) Verification of the metabolic profile of selected deletion strains described in the main article (mean ± SD, n = 3): *fpr1* in Figure 1F, *pda1* in Figures 5D and S4C, and *vps41* in Figure 6C. ^∗^p < 0.05, ^∗∗^p < 0.01, ^∗∗∗^p < 0.001, Student’s t test.

We experimentally validated cluster 135, characterized by high levels of alanine, aspartate, glutamate, and proline and reduced levels of histidine (Figures 6C and S5A, cluster 135; Table S3). The cluster contains genes important both for vesicle fusion at the vacuole and for autophagy, i.e., the class B *vps* mutants *Δvps5*, *Δvps17*, *Δvps41*, *Δvam3*, *Δvam7*, the Rab GTPase Ypt7p and its heterodimeric guanine nucleotide exchange factor (Mon1p/Ccz1p) (Figure 6C, left), and three unrelated deletion strains (*mch5Δ, opt2Δ*, and *tef4Δ*) (Figure 6C, middle). We confirmed the metabolic profile in replicate measurements (Figure S5A), increased rapamycin sensitivity (Figure 6C, left bottom), and, except for *Δtef4*, detected a characteristic phenotype of autophagy deficiency, the impaired cleavage of GFP-Atg8 upon nitrogen starvation (Suzuki et al., 2001) (Figure 6C, right). Further, fluorescence microscopy showed abnormal vacuolar morphology (Figure 6C, right bottom) and a diminished release of GFP-Atg8 to the vacuolar lumen (Figure 6C, bottom). Other high-throughput studies strengthen these associations (Figure 6C, top left); *Δmch5*, *Δopt2*, and *Δtef4* genes have been classified as rapamycin sensitive (Parsons et al., 2004, Xie et al., 2005) and were scored to possess vacuolar morphological defects (Michaillat and Mayer, 2013). *Δmch5* and *Δopt2* deletion strains have further been associated with *Δmon1*, *Δccz1*, *Δvam7*, and *Δvps41* in a screen for H_2_S accumulation (Sambade et al., 2005), while *MCH5* and *OPT2* transcripts change dramatically upon a shift from a rich to poor nitrogen source (both ∼10-fold change) that is impaired upon perturbation of the protein sorting machinery (*Δvps45*) (Kingsbury and Cardenas, 2016).

## Discussion

Despite the importance of amino acids as a key component of metabolism is universally accepted, a comprehensive picture of the genetic network behind their regulation is still missing. Indeed, metabolite concentrations and metabolic fluxes can so far not be calculated from transcriptome and proteome data or from other molecular networks, including protein-protein and genetic interaction data. The metabolome hence provides an orthogonal set of information compared to the other molecular levels (Allen et al., 2003). We here present a genome-scale quantitative metabolic map created by precisely measuring amino acid concentration changes upon deletion of the nonessential *Saccharomyces* coding genome. As a key requirement to map the interactions between genome and biosynthetic metabolism, we study prototrophic cells grown to exponential phase and in minimal-nutrient medium. We focus on amino acids, as they are responsible for a major fraction of the metabolic flux in exponentially growing cells, represent a large proportion of the total metabolite mass, and, while precisely regulated, converge a large fraction of biosynthetic metabolism. Furthermore, we profit from the chemical properties of amino acids that enabled us to develop precise, fast, and reliable analytics to capture large and small concentration changes on the genomic scale.

We found that the yeast amino acid metabolome is sensitive to at least 1,519 gene deletions and that a majority of them specifically impact the metabolome. Obtaining specific metabolic signatures on the genomic scale contradicts assumptions that global physiological factors such as growth rate or external nutrient supply would be the prime regulators of metabolism; each amino acid concentration can be independently affected by a gene deletion, but we find that some amino acids that share functional or biosynthetic properties robustly correlate on the genomic scale. In total, more than 1,000 non-essential genes are found to participate in genome-metabolism interaction, and their joint action creates the amino acid signature of a cell. A majority of these genes are however not direct “metabolic regulators” in a sense that they would be transcription factors, signaling kinases, or metabolic enzymes. Instead, they are “indirect” participants in metabolism and a part of the protein translation machinery, organelles, and transport pathways or operate at the chromatin level to enable gene expression. Of note, the latter were highly enriched for metabolic alterations, but not gene-specific transcription factors, which created much smaller effects. Potentially, an evolutionary explanation for this finding exists. Metabolism is one of the oldest cellular systems that require regulation (Luisi, 2014). The general gene expression machinery, older than gene-specific transcription factors, could have evolved to regulate metabolism. Consistently, we find that the sophisticated transcriptional systems that adapt metabolism in stress situations, like *GCN4*, the SPS sensor, or the nitrogen catabolite repression systems, are largely dispensable for exponential metabolism. This includes *tor1Δ*, whose metabolic signature is strong under nitrogen starvation (Zhang et al., 2011) but was weak and not significant in our growth conditions (Figure 4B). However, TORC1 function is not in essence bound to the presence of the *TOR1* kinase, as its paralog, *TOR2*, can assume its function (Helliwell et al., 1994). Indeed, we found a strong metabolic impact of the kinase network that centers on the TORC1 complex. Deletion of TORC1 upstream activators and cells treated with rapamycin share a similar metabolic signature. Our genome-scale data allowed us to map the responsible downstream processes. We did obtain a genome-wide unique match in the vesicle-mediated transport machinery. Previous studies support a function of TORC1 in the regulation of vesicle-mediated transport. Sec13p and Vam6p, which are required for endomembrane dynamics, also play a role in TORC1 activation (Binda et al., 2009, Panchaud et al., 2013). Furthermore, the small GTPases Vps21p, Ypt1p, Ypt6p, and Ypt7p regulate membrane fusion events via HOPS, CORVET, and GARP, and their deletion causes rapamycin sensitivity and reduced TORC1 activity (Bridges et al., 2012, Thomas et al., 2014). Together with our study, these results imply that at least in respect to amino acid metabolism, the prevalent function of TORC1 in exponentially growing cells is to maintain endomembrane transport. It is worth speculating that the connection between TORC1 and these transport processes is explained by the importance of amino acid recycling. While protein catabolism is highly important for amino acid homeostasis during starvation and in the stationary phase where biomass is maintained, during exponential cell growth, amino acids are synthesized de novo to gain biomass. TORC1 could balance between these metabolic states.

We exploit the unanticipated precision of metabolic signatures for functional annotation on the genomic scale. Amino acid signatures were sufficiently specific to assign rapamycin to TORC1, cycloheximide to translation (Figure 4A), and to group the genome in 280 functional-metabolic clusters. The cluster associations contain orthogonal information in respect to physical, molecular, and genetic interaction networks. Assessing an understudied functional level, functional metabolomics is hence particularly informative about genes that were not annotated by these canonical approaches. Indeed, the number of poorly characterized genes is substantial (Botstein and Fink, 2011) and at present includes up to ∼2,000 yeast open reading frames (ORFs) (Figures S4A and S4B). Revealing their importance, one-third of the essential genes in an engineered minimal genome are required for unknown reasons (Hutchison et al., 2016). The amino acid signatures associate functional terms with 56.6% of the 1,823 barely and uncharacterized *Saccharomyces* genes that were part of our study (Figure S4A). Indicating relevance for a broad number of species, 823 of these genes could be assigned to orthology groups using eggNOG 4.5 (Huerta-Cepas et al., 2016), of which 440 contain human homologs.

It was unanticipated to achieve such a high degree of functional information solely through the amino acid metabolome. However, one needs to keep in mind that amino acids are among the most important and most abundant metabolites and, as a consequence, are tightly regulated, and by originating from different branches of carbon metabolism, they converge on a broad spectrum of metabolic activity. The absolute quantitative nature of our data allows a combination with future studies to address other metabolites, metabolic fluxes, or different growth conditions. Such multiomic attempts will further increase the precision of gene annotation. Indeed, despite assigning >1,500 genes to metabolism, our study is far from providing a complete picture of genome-metabolome interactions, as experiments that depend on exponential growth conditions fail to capture essential, condition-, and stress-responsive genes. Important orthogonal information would be provided by flux measurements, as a change in amino acid concentrations will not always reflect changes in flux, and vice versa. Finally, as in other functional-genomics studies, ours depends on the quality of the genetic library and the natural stability of the eukaryotic genome. Indeed, many genetic modifications trigger compensatory or secondary mutations, which have to be taken into account when interpreting individual phenotypes (Teng et al., 2013).

In conclusion, we present a precise, genome-spanning functional-metabolomic map in budding yeast. Converging a major fraction of cellular metabolism, and by being precisely captured by targeted analytics, the amino acid metabolome provides system-scale insights into the regulation and homeostasis of biosynthetic metabolism in the exponentially growing cell. The biosynthetic metabolome is sensitive to the concerted action of more than 1,500 genes. In most instances, their deletion creates highly specific concentration signatures that attribute dominating roles of the metabolism-regulatory network to gene regulation at the chromatin level, protein translation, and the protein transport machinery. The genomic-scale view of metabolism connects the regulatory networks with their main effectors, as exemplified by TORC1, whose main metabolic impact under exponential growth is associated with vesicle-mediated transport. Functional metabolomics is thus revealed to possesses a high potential for functional gene annotation, as exemplified by assigning 127 non-essential proteins to the correct structural element of the ribosome, reconstructing the pathway of LA biosynthesis and its association with the pyruvate dehydrogenase complex. By providing orthogonal information in respect to existing physical and genetic interaction networks, these phenotype-based associations are particularly effective for annotating genes that have so far remained uncharacterized. As a result, more than half of the so far poorly characterized yeast genes, many of which possess orthologs in other species (up to humans), receive a putative functional association over their metabolic signature.

## STAR★Methods

### Key Resources Table

**Table:** 

REAGENT or RESOURCE	SOURCE	IDENTIFIER
**Antibodies**		

Goat anti-rabbit IgG-HRP	Santa Cruz Biotechnology	Cat# 170-6516, RRID: AB_11125547
Rabbit polyclonal to GFP	Abcam	Cat# ab290, RRID: AB_2313768

**Chemicals, Peptides, and Recombinant Proteins**		

Algae hydrolysate amino acid mixture (U-^13^C, 97-99%; U-15N, 97-99%)	Cambridge Isotope Laboratories	Cat# CNLM-452-0.5
L-Amino acids analytical standard	Sigma-Aldrich	Cat# LAA21
Acetonitrile UPLC grade	Greyhound BIOSOLVE	Cat# Bio-012041
Ultra-pure water, ULC-MS grade	Greyhound BIOSOLVE	Cat# 23214125
Methanol Absolute ULC-MS grade	Greyhound BIOSOLVE	Cat# BIO-13684102
Ammonium formate	Fluka	Cat# 14266
Formic acid	Fluka	Cat# O6454

**Deposited Data**		

Amino acid concentration profiling data for the *S. cerevisiae* deletion collection (BY4741)	This study	Table S3 and is further available through Metabolights database (http://www.ebi.ac.uk/metabolights/) and Mendeley Data http://dx.doi.org/10.17632/bnzdhd6ck8.1
A metabolic gene cards, an individual report sheet, summarizing the results for each analyzed open reading frame	This study	http://ralser.sysbiol.cam.ac.uk/metabogenecards and Mendeley Data http://dx.doi.org/10.17632/bnzdhd6ck8.1
Cryo-EM reconstruction of the 80S-eIF5B-Met-itRNAMet Eukaryotic Translation Initiation Complex	Fernández et al., 2013	PDB: 4V8Y
List of transcriptional regulators from: YEASTRACT and YeTFaSCo databases	de Boer and Hughes, 2012, Teixeira et al., 2014	http://www.yeastract.com/
		http://yetfasco.ccbr.utoronto.ca/
List of kinases and phosphatases from the Yeast Kinase and Phosphatase Interactome (KPI) Resource	Breitkreutz et al., 2010	http://yeastkinome.org/
List of genes for the unfolded protein response (UPR)	Travers et al., 2000	N/A
List of genes for nitrogen catabolite repression (NCR)	Godard et al., 2007	N/A
List of genes for general amino acid control (GAAC)	Moxley et al., 2009	N/A
List of genes for the TOR network	Fournier et al., 2010	N/A
List of genes for SPS-sensing pathway	Eckert-Boulet et al., 2004	N/A
Curated genetic information, gene ontology, literature and phenotype annotation from the Saccharomyces Genome Database (SGD)	Cherry et al., 2012	http://www.yeastgenome.org/
Manually curated gene ontology terms for precision-recall analysis.	Myers et al., 2006	N/A
Protein complexes annotation from the COMPLEAT database	Vinayagam et al., 2013	http://www.flyrnai.org/compleat/
Physical and genetic interaction between gene pairs from the BIOGRID database	Chatr-Aryamontri et al., 2015	http://thebiogrid.org/

**Experimental Models: Organisms/Strains**		

Prototrophic *Saccharomyces cerevisiae* gene deletion collection (*MAT*a, monoclonal, prototrophy restored episomally)	Mülleder et al., 2012	http://www.euroscarf.deindex.php?name=News
Prototrophic *Saccharomyces cerevisiae* gene deletion collection (*MAT*a, polyclonal, genomically restored prototrophy)	Gibney et al., 2013	N/A

**Recombinant DNA**		

pHLUM	Mülleder et al., 2012	Addgene ID: 40276
pRS316-GFP-AUT7 (expresses GFP-Atg8)	Suzuki et al., 2001	N/A
pHLM	This study	Addgene ID: 64168

**Software and Algorithms**		

R: A Language for Data Analysis and Graphics, version 2.1	http://www.r-project.org	N/A
grofit	http://cran.r-project.org/web/packages/grofit/index.html	N/A
ImageJ, version 1.49 g	https://imagej.nih.gov/ij/	N/A
MassHunter software suite	Agilent Technologies	N/A
Softworx	Deltavision	N/A

**Other**		

ACQUITY UPLC BEH amide column (130Å, 1.7 μm, 2.1 mm × 100 mm)	Waters	Cat# 186004801

### Contact for Reagent and Resource Sharing

Further information and requests for reagents may be directed to, and will be fulfilled by the Lead Contact Markus Ralser (markus.ralser@crick.ac.uk).

### Experimental Model and Subject Details

#### Yeast

Batch assignments: The 4913 deletion strains were arrayed on 62 96-well plates, joining plates 50 and 73 as well as plates 71 and F (Table S4), in comparison to the original arrangement of the prototrophic library (Mülleder et al., 2012). The original *MAT*a deletion collection contains plates, which are enriched for deletion mutants showing growth defects: plates 50, 51 and 70 - 75 (Winzeler et al., 1999). Plates A-G from the prototrophic library creation add to these plates as they contain strains, which did not easily transform with the prototrophy conferring plasmid (Mülleder et al., 2012). To avoid any influence of the heterogeneous assembly of the strains, we assigned the plates to 11 batches distributing the plates 50-51, 70-75 and A-G evenly over the course of analysis (Table S4).

The strains were transferred to synthetic minimal (SM) agar medium (6.7 g/l yeast nitrogen base without amino acids (Y0626, SIGMA), 2% glucose, 2% agar) using a robot (Rotor, Singer instruments) and incubated for 48 hr at 30°C. These spots were used for the inoculation of cultures in liquid SM (96-well plate, 150 μl/well) and were grown for 24 hr, diluted 1:20 and finally cultured in 96-deep well plates with 1.685 ml SM medium and one 2 mm borosilicate bead/well for enhanced stirring. These plates were incubated for 8 hr in a Heidolph Titramax 1000 (900 rpm, 30°C).

Samples for verification experiments (Figure S5) were generated similarly in multi-well format. For chemical perturbation experiments (Figure S3), cells were inoculated in 3 ml SM medium and grown ON. Cultures were inoculated at an OD_600_ of 0.15 in 10 ml SM medium containing rapamycin at 50 nM or cycloheximide at 890 nM and were incubated for 8 hr before an aliquot corresponding to 1.5 OD_600_ was collected. Starvation medium, SD (–N), was prepared with 1.7 g/l yeast nitrogen base without amino acids and ammonium sulfate (Y1251 SIGMA) and 2% glucose.

### Method Details

#### Quantification of Amino Acids in High Throughput

Strains from the same batch were cultivated and their metabolites extracted on the same day and analyzed together by LC-MS/MS. Before analysis by LC-MS/MS the order of samples within the same batch was randomized. During analysis a quality control sample (QC) was assessed every 24 samples and in total 237 times. The QC was prepared by joining the extracts of the first batch including metabolites of 351 deletion strains. Eventually, it was used to monitor analytical performance and to calculate the technical variation (Figure S1B). In a second screening round, the 479 deletion strains that showed lowest metabolite concentrations were arranged on 6 additional plates and the analysis was repeated (batch 12).

#### Metabolite Extraction

Cells were collected by centrifugation, the medium discarded and the samples extracted with 200 μl 80°C hot ethanol containing isotope labeled amino acid standards (50 μg/ml algae hydrolysate amino acid mixture (U-13C, 97%–99%; U-15N, 97%–99%; Cambridge Isotope Laboratories CNLM-452-0.5) that were used for quality control and to detect outliers. Residual medium resulted in a final ethanol concentration of approximately 80%. The extract was heated for 2 min at 80°C, vigorously mixed on a vortex mixer and incubated for further 2 min at 80°C. The extract was cleared from debris by centrifugation and stored at −80°C. Before analysis, extracts were thawed and sonicated for 5 min. For chemical perturbation experiments, the supernatant was removed completely and the cells extracted with 200 μl 80% ethanol.

#### Analysis by LC-MS/MS

Amino acids were separated by hydrophilic interaction liquid chromatography (HILIC) using an ACQUITY UPLC BEH amide column (130Å, 1.7 μm, 2.1 mm X 100 mm) on a liquid chromatography (Agilent 1290 Infinity) and tandem mass spectrometry (Agilent 6460) system. The method separates the amino acids by chromatographic means and/or mass transition (Figure 1A), is specific for intracellular amino acids (Figure S1B), and fully covers the dynamic range of physiological amino acid concentrations (Figure S1C) (Mülleder et al., 2016).

Buffer A was composed of 50:50 acetonitrile/water (Greyhound Bio-012041, Greyhound 23214125), 10 mM ammonium formate (Fluka 14266), 0.176% formic acid (Fluka O6454) and buffer B of 95:5:5 acetonitrile/methanol/water (Greyhound BIO-13684102), 10 mM ammonium formate, 0.176% formic acid. The gradient elution was performed at a constant flow rate of 0.9 ml/min. Starting conditions were 85% buffer B, after 0.7 min the concentration of buffer B was decreased gradually to 5% until 2.55 min and kept for further 0.05 min before returning to initial conditions. The column was then equilibrated resulting in a total runtime of 3.25 min. Compounds were identified by matching retention time and fragmentation (MS^2^) with commercially obtained standards (LAA21, Sigma-Aldrich). Signals for free amino acids were then acquired in dynamic SRM mode in Masshunter software. The *m/z* transition and retention times are listed in Table S5.

#### Rapamycin Sensitivity Assay

Strains were inoculated at 0.1 OD_600_ at 11 rapamycin concentrations from 7 to 1000 nM (n = 4) in a 384 multi well plate (70 μl/well) in SM medium and growth was monitored for 48 hr. The maximum specific growth rate was determined with R package grofit and used to determine the EC_50_ value in a dose response curve (Figure 6C).

#### Visualization of Proteolytic Cleavage of GFP-Atg8 by Western Blot

Prototrophic strains expressing GFP-Atg8 were collected in SM medium during exponential growth, and inoculated in nitrogen-starvation medium SD (–N) for 6 hr at 30°C. Aliquots corresponding to 5 OD_600_ were collected, washed with 10% TCA and ice-cold acetone and proteins mechanically extracted in MURB buffer using a FastPrep instrument and acid-washed glass beads. The samples were heated 10 min at 70°C, an aliquot corresponding to 0.3 OD_600_ was loaded and GFP and GFP-Atg8 were visualized with chemiluminescence on a nitrocellulose membrane using rabbit polyclonal to GFP (Abcam, ab290) and goat anti-rabbit IgG-HRP, (Santa Cruz Biotechnology, sc-2004) antibodies.

#### Fluorescence Microscopy

Cells were collected during exponential growth in SM medium, starved for 6 hr in SD (–N) medium and mounted on 1% agarose pads prepared with SD (–N) medium. Images were acquired on an Olympus IX81 wide field microscope (Deltavision, GE Healthcare) equipped with a 100 × /1.4 numerical aperture oil immersion lens (UPLSApo Oil, Olympus) and a CoolSNAP HQ2 CCD camera (Figure 6C). The filter set used for GFP was Ex 490/20 Em 528/38. Image stacks were collected in 0.2 μm increments and for deconvolution the ratio conservative algorithm was used in Deltavision Softworx software. Five to ten slices of one image Z-stack were selected, background noise calculated and removed, and the slices projected with the “sum slices” method and brightness and contrast adjusted in ImageJ.

### Quantification and Statistical Analysis

All statistical analysis was performed in R (http://www.r-project.org).

#### Quantification of Free Amino Acids by External Calibration

To reduce effects of instrumental drift we used a “bracketing” method, measuring every 24 samples a set of standards. External calibration was then performed including both sets of standards, before and after the set of samples. For each amino acid we chose either a linear regression or linear regression after log transformation (power-law fit) (Table S5). Analytical standards that were more than 4 times higher or lower in concentration than the highest or lowest concentrated sample were excluded.

### Quality Control and Data Correction

#### Strain Quality Management

We were able to obtain 4678 strain specific free amino acid profiles, were not however able to do so for another 259 deletion strains, mostly for the reasons of lethality on minimal medium ( = auxotrophy). 208 strains did not show any growth or highly impaired growth in the absence of amino acid supplementation, as defined by the following criteria: we first determined the number of average cell divisions by random selection of 175 strains and determination of their optical density (OD_595_). The median OD_595_ after 7.5 hr of cultivation was 1.46 and the number of cell divisions 2.79. We next calculated a probabilistic dilution factor (probabilistic quotient normalization, PQN) for each deletion strain. A dilution factor of 0.25, corresponding to approximately 0.8 cell division, was chosen as a cutoff criterion; all strains below this threshold were defined as slow growers.

Additionally, we excluded the complemented deletion strains *his3Δ::kanMX4* and *met17Δ::kanMX4*, 3 deletions strains that were identified to be cross-contaminated (*aco1Δ*, *lip5Δ* and *mtc1Δ*), and 24 strains with outdated ORF annotations (SGD).

#### Batch Normalization and Correction for Sample Dilution

For batch normalization we adapted the approach from the MIPHENO normalization workflow (Bell et al., 2012), where a scaling factor is calculated to bring the batch means to a global mean. First we calculated the overall mean considering all samples. Next, and in contrast to the previously described workflow, we compensated for dilution effects within batches by PQN before determining the mean for each batch. By dividing each sample by its respective batch mean and multiplying by the overall mean, we bring each batch to the same level. Finally, we corrected for sample dilution using PQN normalization.

#### Robust Estimation of Summary Statistics and Identification of Altered Metabolic Profiles

The unperturbed value for each metabolite is determined with robust statistical methods by excluding the contribution of few, significantly changing metabolites. We used the Minimum Covariance Determinant (MCD) estimator to obtain robust values for mean, standard deviation, correlation and covariance for each amino acid (Figures 1 and 2A). The robustly estimated average amino acid concentration of all analyzed strains, was identical to that of the wild-type background strain (Figure S1F). With the robust mean and covariance matrix we calculated Mahalanobis distances as robust multivariate metric for dissimilarity from the unaffected amino acid profile. *p-value*s for the amino acid concentrations were calculated using the *Z*-test statistic. A *p-value* for the change in the overall amino acid profile was calculated from the Mahalanobis distance using the χ^2^ statistic. All *p-value*s were adjusted for multiple testing with the Benjamini & Hochberg method (BH) to identify significantly altered metabolic profiles (Figure 1) (Table S3).

#### Estimation of Intracellular Concentration

To estimate the intracellular concentration of free amino acids (Figures 1B and 1F) we determined the median OD_595_ of the analyzed samples (1.46), the approximate extraction volume (200 μl) and used the values for cells/OD_595_ (3.2^∗^10^7^) and cell volume (45.54 fL) for the strain BY4741 in synthetic minimal medium.

#### Quality Control and Completeness

Eventually, we completed a metabolic profile for 4678 yeast deletion strains, capturing 99% of the gene-knockouts that do not possess a strong growth defect in minimal medium, corresponding to 95% of all viable strains (Figure 1A, Table S3). Cysteine was omitted from further analysis, due to its property of being quickly oxidized upon cell lysis and hence giving imprecise results. The precision of the remaining amino acid quantifications is reflected in an R^2^ greater 0.99 in 95% of 4518 calibration curves, revealing low technical variability (Figures S1D and S1E). To assess the quality of the used prototrophic library used (Mülleder et al., 2012) we have compared the results of selected strains isolated from an analogous genomic library (Gibney et al., 2013) and obtained comparable metabolic footprints (Figures S5B and S5C).

### Cluster and Enrichment Analysis

#### Consensus Clustering

Consensus clustering was based on hierarchical clustering using pairwise Mahalanobis distance (Monti et al., 2003) and Ward’s method as agglomeration method. Before calculating the consensus metrics, we perturbed the data by randomly removing 0%–20% of amino acid profiles and cut the dendrogram at 8 different depths with the dynamicTreeCut package in R, and repeated this in total 500 times. The obtained consensus matrix was then subtracted from 1 and used as the distance metric for hierarchical cluster analysis (Ward’s method). 280 clusters were obtained with following settings: minClusterSize = 2, respectSmallClusters = T, deepSplit = 4.

#### Mapping the Response to Rapamycin or Cycloheximide to the Profiles of the Deletion Collection

Yeast was treated with 50 nM rapamycin or 890 nM cycloheximide in exponentially growing culture in minimal medium, and an amino acid concentration profile recorded. To enable a comparison of the small scale perturbation and high-throughput screening experiments, the amino acid concentrations of the small scale experiments were standardized by first subtracting the values of the untreated isogenic parental strain and then by dividing by the robust standard deviation (MCD) determined for the deletion collection. The 4678 deletion strains together with the rapamycin or cycloheximide treated wt were then hierarchically clustered (pairwise Mahalanobis distance using the robust covariance matrix (MCD) determined for the deletion collection, Ward’s method) (Figure 4A).

### Enrichment Analysis

Gene set enrichment analysis (GSEA) (Figures 2B and 3D) was performed with piano (Väremo et al., 2013) and was based on the χ^2^-test statistic. Enrichment analysis GO categories related to vesicle mediated transport was calculated by hypergeometric testing (phyper function in R) (Figure 4F).

#### Enrichment Analysis in Clusters

The list of genes obtained for each of the clusters was tested for enrichment of GO and KEGG annotation in R with the GOstats package.

### Enrichment Analysis in Clusters for Each Open Reading Frame

To improve the predictive value for functional characterization of open reading frames (ORF), we removed the respectively studied ORF from the cluster and from the background-set of genes in all analyses.

### Enrichment Analysis for Metabolic Gene Cards

Phenotypic descriptions and literature annotations were obtained from SGD on 10.08.2016 and the significance of overrepresentation calculated using hypergeometric testing. Obtained *p-value*s were corrected with the FDR method from Benjamini & Hochberg (p.adjust function in R). Enrichment for gene ontology and KEGG categories was calculated in R with the GOstat package (10.08.2016).

### Construction of a Gene Ontology Map

The gene ontology database for *Saccharomyces cerevisiae* annotates 4678 analyzed genes by 6806 functional terms (Cherry et al., 2012). This includes direct annotations, or annotations inferred or propagated from children to their parent terms through the GO graph. Considering only direct gene annotations, we detect an enrichment for 80 GO terms by GSEA (BH adjusted p < 0.05) and illustrate them in a network for systematic evaluation. In this gene ontology map GO terms constitute the nodes that are connected by gene overlap, when two terms share more than 50% of their annotated genes. Edges are drawn for nodes that have a shortest path length of 1 or 2 and redundant edges are removed. We chose a force-directed graph layout (Fruchterman–Reingold algorithm) and identified clusters based on edge betweenness (igraph package in R).

### Evaluation of Functional Metabolomics Compared to Available Datasets

#### Precision Recall Analysis

The gold standard dataset used for gene ontology terms contained only manually curated annotations (Myers et al., 2006). The true positive (TP) sets for protein complexes are based on the COMPLEAT database (Vinayagam et al., 2013) and for physical and genetic interaction we used the low-throughput annotation in the BIOGRID database (Chatr-Aryamontri et al., 2015). A true negative gene set (TN) for protein complexes was generated from proteins belonging to distinct complexes and being annotated to different subcellular locations (gene ontology), excluding the cytosol, and for physical and genetic interaction by randomly selecting genes of non-interaction pairs (BIOGRID). Before analyzing the predictive power of amino acid profile similarity in the three datasets, the TN sets were reduced to 9 times the size of the TP datasets to fix the precision of a random classifier at 10%.

#### Orthogonality of Metabolic Signatures

To compare functional annotations of amino acid profiles with other known functional networks, each of the 280 clusters for amino acid profile similarity was considered as a fully connected graph that resulted in a network with 4678 nodes and 61429 edges. We downloaded multiple yeast functional annotation networks from STRING v10 (http://string-db.org/) (Szklarczyk et al., 2015) and YeasNet v.3 (http://www.inetbio.org/yeastnet/) (Kim et al., 2014) resources and compared whether edges based on amino acid profile similarity were found in any known functional network (Figures 5C and S4C).

### Data Retrieved From Primary Literature or Databases

Curated genetic information, literature and phenotype annotation was collected from the *Saccharomyces* Genome Database (SGD) (Cherry et al., 2012). A list of transcriptional regulators was compiled from the overlap of the YEASTRACT and YeTFaSCo databases (de Boer and Hughes, 2012, Teixeira et al., 2014), a list of kinases and phosphatases was obtained from the Yeast Kinase and Phosphatase Interactome (KPI) Resource (Breitkreutz et al., 2010). We obtained gene sets for following regulatory pathways: unfolded protein response (UPR) (Travers et al., 2000), nitrogen catabolite repression (NCR) (Godard et al., 2007), general amino acid control (GAAC) (Moxley et al., 2009), TOR network (Fournier et al., 2010), the SPS-sensing pathway (Eckert-Boulet et al., 2004) and a list of genes of amino acid metabolism (Figure 3D) and vesicle mediated transport (Figure 4F) from *Saccharomyces cerevisiae* gene ontology. The Cryo-EM structure of the 80S-eIF5B-Met-itRNAMet eukaryotic translation initiation complex (PDB: 4V8Y) (Fernández et al., 2013) was obtained from RCSB Protein Data Bank and subunits colored according to their amino acid profile, related to Figure 5B.

### Data and Software Availability

The complete dataset is provided in Table S3 and is further available through Metabolights database (http://www.ebi.ac.uk/metabolights/) and Mendeley Data (http://dx.doi.org/10.17632/bnzdhd6c).

#### Metabolic Gene Cards

To make the metabolism-driven associations broadly accessible, we have created a ‘metabolic gene card’ for each of the 4678 genes studied. This gene card lists the functional associations each gene receives over its metabolic signatures, and depicts the molecular network it participates in. The short description for each open reading frames was obtained from SGD. Calculation and illustration of the perturbation on the amino acid concentration and identification of deletion strains with similar metabolic profile was performed as described above. The gene cards are accessible at http://ralser.sysbiol.cam.ac.uk/metabogenecards/ and deposited in Mendeley Data (http://dx.doi.org/10.17632/bnzdhd6c), and linked with the *Saccharomyces* Genome Database (SGD).

## Author Contributions

Conceptualization, M.R.; Methodology, M.M.; Investigation, M.M., E.C., R.K.W., and F.E.; Formal Analysis, M.M., M.T.A, and A.Z.; Writing, M.M. and M.R.; Funding Acquisition, M.R.; Supervision, M.M. and M.R.

## Supplemental Information

Table S1. Enrichment Analysis of Gene Function, Related to Figures 2, 4, 5, and 6 Gene set enrichment analysis, and functional enrichment analysis, of gene deletions dependent on their metabolomics signature.

Document S1. Tables S2, S4, and S5

Table S3. Amino Acid Profiling: Complete Dataset, Related to Figures 1, 2, 3, 4, 5, and 6 Quantitative data about amino acid concentration changes, as measured by liquid chromatography/selective reaction monitoring in yeast gene deletion strains growing exponentially and in minimal medium.

Data S1. Association of Deletion Strains by Co-clustering
