# Supplementary material for: Functional Metabolomics Describes the Yeast Biosynthetic Regulome
Source: Cell. 2016 Oct 6;167(2):553–565.e12. doi: 10.1016/j.cell.2016.09.007 (PMC5055083; doi:10.1016/j.cell.2016.09.007)
Supplement: Document S1. Tables S2, S4, and S5 [file mmc2.pdf]

**Table S2. Transcriptional Regulators, Kinases and Phosphatases that Affect Amino Acid Metabolism. Related to Figure 3 B-C.**

| amino acid | transcriptional regulator (number) | kinase (number) | phosphatase (number) | total No |
|---|---|---|---|---|
| alanine | *ADA2 GAT1 GCR2 HAP5 HMO1 HOT1 LEU3 MOT2 RGT1 SPT10 USV1 YRR1* **(12)** | *ADK1 BUB1 CLB2 DBF2 ELM1 FAB1 GIN4 MCK1 PCL7 PHO80 PHO85 SKY1 TCO89 VIP1* **(14)** | *OCA1 PTC6 SAP155* **(3)** | 29 |
| aspartate | *HAP5 HIR2 IXR1 MOT2 SIR3 SPT10 UME6 XBP1* **(8)** | *MCK1 PHO80 SCH9 SLT2 SPS1 VIP1* **(6)** | *OCA1 PTC1 SIT4* **(3)** | 17 |
| glutamate | *GCR2 GLN3 NRG1 RGT1 SPT10 YRR1* **(6)** | *ADK1 DBF2 ELM1 FAB1 KIN4 MRK1 NPR1 SKM1* **(8)** | *OCA1 SAP155 SIT4* **(3)** | 17 |
| phenylalanine | *SPT10 YRR1* **(2)** | *CTK3 PHO85 VIP1* **(3)** | *SIT4* **(1)** | 6 |
| glycine | *SKO1 SNF2 SOK2 TYE7 YRR1* **(5)** | *FRK1 PHO80 SSN3* **(3)** | *OCA1* **(1)** | 9 |
| histidine | *PHO2 YRR1* **(2)** | *CGI121 FAB1 SSN8 TCO89* **(4)** | *SIT4* **(1)** | 7 |
| isoleucine | *AFT1 SFL1 SPT10 SWI6 YRR1* **(5)** | *YGK3* **(1)** | *OCA1 PTC6* **(2)** | 8 |
| lysine | *ADA2 CIN5 HIR2 LYS14 PHO2 SFL1 SFP1 SIR2 SNF2 ZAP1* **(10)** | *ADK1 MRK1 PHO85 SSN8* **(4)** | *OCA1 SIT4* **(2)** | 16 |
| leucine | *YRR1* **(1)** | | | 1 |
| methionine | *AFT1 MIG1 MOT2 SFP1 SPT10 YRR1* **(6)** | *ADK1* **(1)** | *SIT4* **(1)** | 8 |
| asparagine | *CIN5 GLN3 HAP2 HAP5 LYS14 SFP1 UME6 YRR1* **(8)** | *ADK1 CKA2 FAB1 SCH9 TCO89 YCK3* **(6)** | | 14 |
| proline | *AFT1 GCR2 GLN3 HMO1 LEU3 LYS14 SPT10 SWI6* **(8)** | *ADK1 CKA2 CTK3 FAB1 NPR1 PHO85 SCH9* **(7)** | *OCA1 SAP155* **(2)** | 17 |
| glutamine | *ADA2 GLN3 HAP2 HAP5 LEU3 SWI6 YRR1* **(7)** | *CKA2 PHO85 SCH9* **(3)** | *OCA1 PTC1* **(2)** | 12 |
| arginine | *AFT1 ARG81 DOT6 LEU3 LYS14 PHO2 SWI6* **(7)** | *CGI121 MCK1 NPR1 PBS2 SCH9* **(5)** | *SIT4* **(1)** | 13 |
| serine | *CHA4 HMO1 IXR1 LEU3 LYS14 RPH1 SKO1 SNF2 SPT2 USV1 YRR1* **(11)** | *ADK1 ELM1 FAB1 PCL1 PCL5 PHO80 PRO1 SKY1 SSN3 TCO89* **(10)** | *OCA1 PTC1* **(2)** | 23 |
| threonine | *ADR1 DAL81 GZF3 HOT1 SIR2 SNF2 SPT10 UME6 YRR1* **(9)** | *ADK1 CKA2 CTK3 FAB1 PHO80 YGK3* **(6)** | *PTC1 SAP4* **(2)** | 17 |
| valine | *AFT1 AFT2 DAL81 LEU3 SNF2 SPT10* **(6)** | *FAB1 FRK1 PKH2 PRO1 TCO89* **(5)** | *OCA1 PTC1 PTC6* **(3)** | 14 |
| tryptophan | *GCN4 LYS14 SUM1 YRR1* **(4)** | *VIP1* **(1)** | | 5 |
| tyrosine | *AFT1 ASH1 LEU3 LYS14 MAC1 MOT2 PHO2 SNF2 SPT10 SUM1 ZAP1* **(11)** | *ADK1 PBS2 PHO85 PRO1 SSN8 TCO89 VIP1* **(7)** | *OCA1 PTC1 SIT4* **(3)** | 21 |

**Table S4. Assignment of strains to batches. Related to STAR Methods.**

| batch 1 | batch 2 | batch 3 | batch 4 | batch 5 | batch 6 | batch 7 | batch 8 | batch 9 | batch 10 | batch 11 |
|---|---|---|---|---|---|---|---|---|---|---|
| 43 | 23 | 16 | 38 | 25 | 13 | 21 | 27 | 17 | 32 | 26 |
| 28 | 7 | 31 | 20 | 5 | 9 | 36 | 8 | 14 | 42 | 4 |
| 41 | 1 | 37 | 11 | 12 | 29 | 19 | 10 | 18 | 34 | 30 |
| 15 | 44 | 24 | 22 | 6 | 40 | 3 | 35 | 2 | 33 | 39 |
| | | B | C | 47 | 45 | G | E | D | A | 46 |
| | | 49 | 74 | 71&F | 70 | 72 | 48 | 50 | 51&73 | 75 |

**Table S5. Details for the analytical method to determine amino acids levels by LC-MS/MS; SRM transitions. Related to STAR Methods.**

| amino acid | compound abbreviation | SRM transition | fragmentor (V) | collision energy (V) | retention time (min) | external calibration |
|---|---|---|---|---|---|---|
| alanine | A | 90 > 44.1 | 50 | 8 | 1.87 | linear |
| arginine | R | 175.1 > 70 | 100 | 15 | 2.37 | power |
| asparagine | N | 133.1 > 74 | 80 | 9 | 2.12 | linear |
| aspartate | D | 134.1 > 74 | 80 | 10 | 2.24 | linear |
| glutamate | E | 148.1 > 84.1 | 75 | 10 | 2.09 | power |
| glutamine | Q | 147.1 > 84 | 50 | 16 | 2.07 | power |
| glycine | G | 76 > 30.1 | 50 | 5 | 1.98 | linear |
| histidine | H | 156.1 > 110.2 | 80 | 12 | 2.36 | power |
| isoleucine | I | 132.1 > 86 | 80 | 8 | 1.33 | power |
| leucine | L | 132.1 > 86 | 80 | 8 | 1.24 | power |
| lysine | K | 147.1 > 84 | 50 | 16 | 2.41 | power |
| methionine | M | 150.1 > 104 | 40 | 8 | 1.47 | linear |
| phenylalanine | F | 166.1 > 120 | 100 | 9 | 1.23 | power |
| proline | P | 116.1 > 70.1 | 100 | 13 | 1.60 | power |
| serine | S | 106 > 60 | 40 | 9 | 2.10 | power |
| threonine | T | 120.1 > 74 | 80 | 9 | 1.96 | power |
| tryptophane | W | 205.1 > 188 | 85 | 5 | 1.29 | linear |
| tyrosine | Y | 182 > 165 | 90 | 5 | 1.64 | power |
| valine | V | 118.1 > 71.9 | 100 | 10 | 1.57 | power |
