# Supplementary material for: Functional Metabolomics Describes the Yeast Biosynthetic Regulome
Source: Cell. 2016 Oct 6;167(2):553–565.e12. doi: 10.1016/j.cell.2016.09.007 (PMC5055083; doi:10.1016/j.cell.2016.09.007)
Supplement: Data S1. Association of Deletion Strains by Co-clustering [file mmc4.pdf]

Association of deletion strains by co-clustering

page 2 - 48: **Mapping the rapamycin- and cycloheximide-induced amino acid profile with that of the knock out collection**

hierarchical-clustering of all deletion strains with wt samples treated with 50 nM rapamycin or 890 nM cycloheximide

distance matrix: pairwise Mahalanobis distance
agglomeration method: Ward's method

page 49 - 95: **280 similarity clusters informative of gene function**

consensus-clustering of all deletion strains

optimal number of 280 clusters were identified using the adaptive branch pruning algorithm in the dynamicTreeCut package in R.

Mapping the rapamycin- and cycloheximide-induced amino acid profile with that of the knock out collection

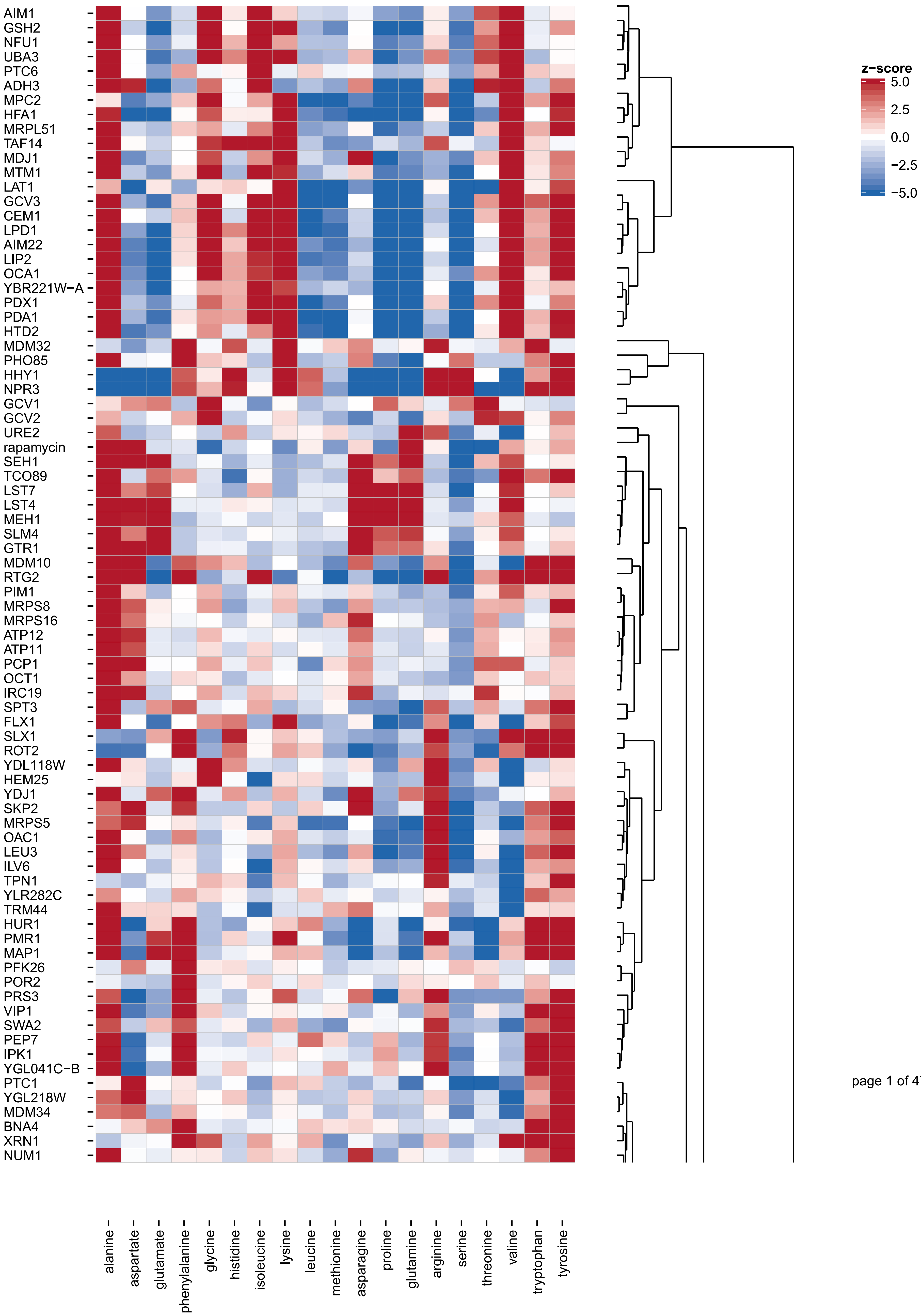

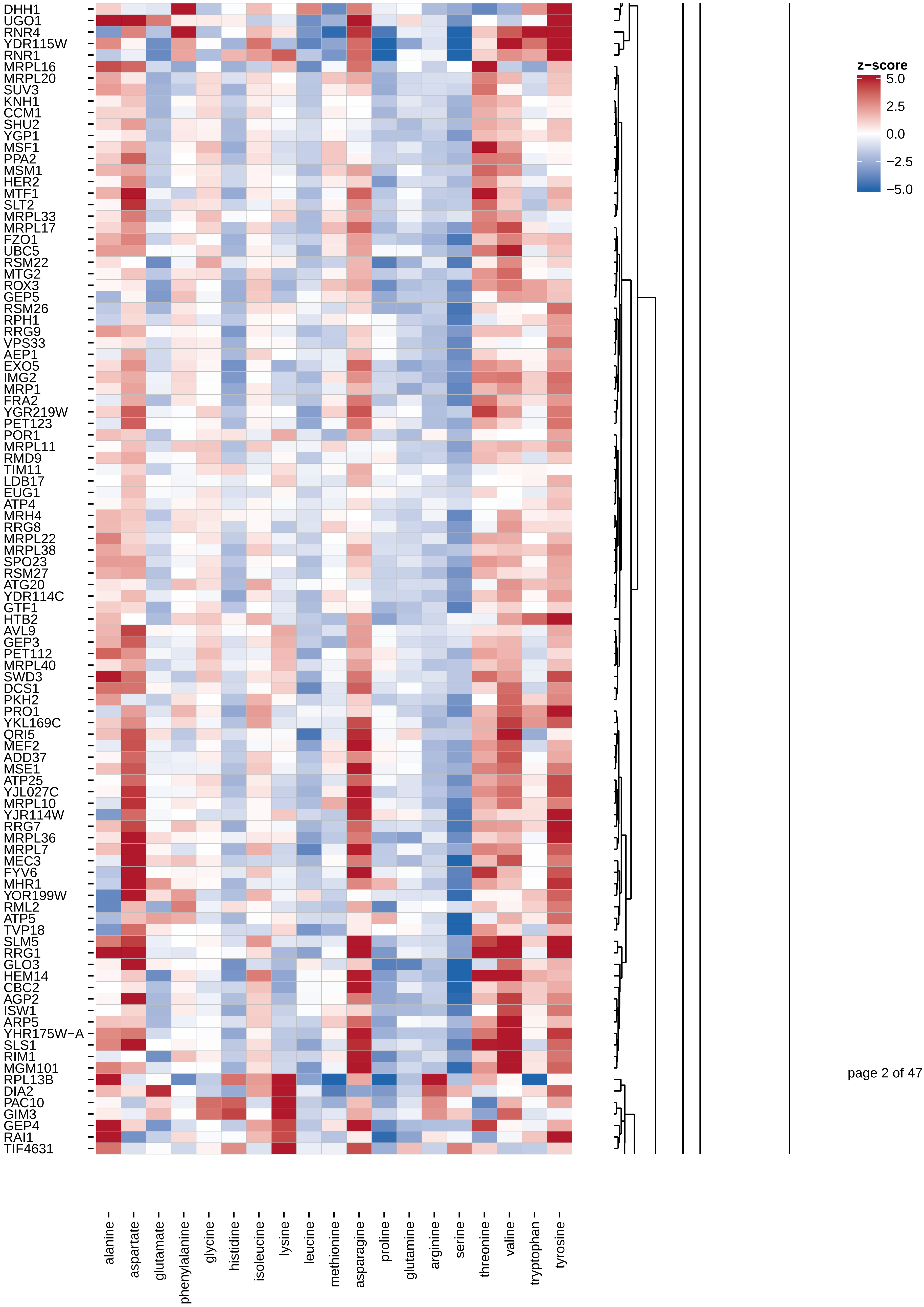
z−score
5.0
2.5
0.0
−2.5
−5.0
DHH1
UGO1
RNR4
YDR115W
RNR1
MRPL16
MRPL20
SUV3
KNH1
CCM1
SHU2
YGP1
MSF1
PPA2
MSM1
HER2
MTF1
SLT2
MRPL33
MRPL17
FZO1
UBC5
RSM22
MTG2
ROX3
GEP5
RSM26
RPH1
RRG9
VPS33
AEP1
EXO5
IMG2
MRP1
FRA2
YGR219W
PET123
POR1
MRPL11
RMD9
TIM11
LDB17
EUG1
ATP4
MRH4
RRG8
MRPL22
MRPL38
SPO23
RSM27
ATG20
YDR114C
GTF1
HTB2
AVL9
GEP3
PET112
MRPL40
SWD3
DCS1
PKH2
PRO1
YKL169C
QRI5
MEF2
ADD37
MSE1
ATP25
YJL027C
MRPL10
YJR114W
RRG7
MRPL36
MRPL7
MEC3
FYV6
MHR1
YOR199W
RML2
ATP5
TVP18
SLM5
RRG1
GLO3
HEM14
CBC2
AGP2
ISW1
ARP5
YHR175W−A
SLS1
RIM1
MGM101
RPL13B
DIA2
PAC10
GIM3
GEP4
RAI1
TIF4631
alanine
aspartate
glutamate
phenylalanine
glycine
histidine
isoleucine
lysine
leucine
methionine
asparagine
proline
glutamine
arginine
serine
threonine
valine
tryptophan
tyrosine

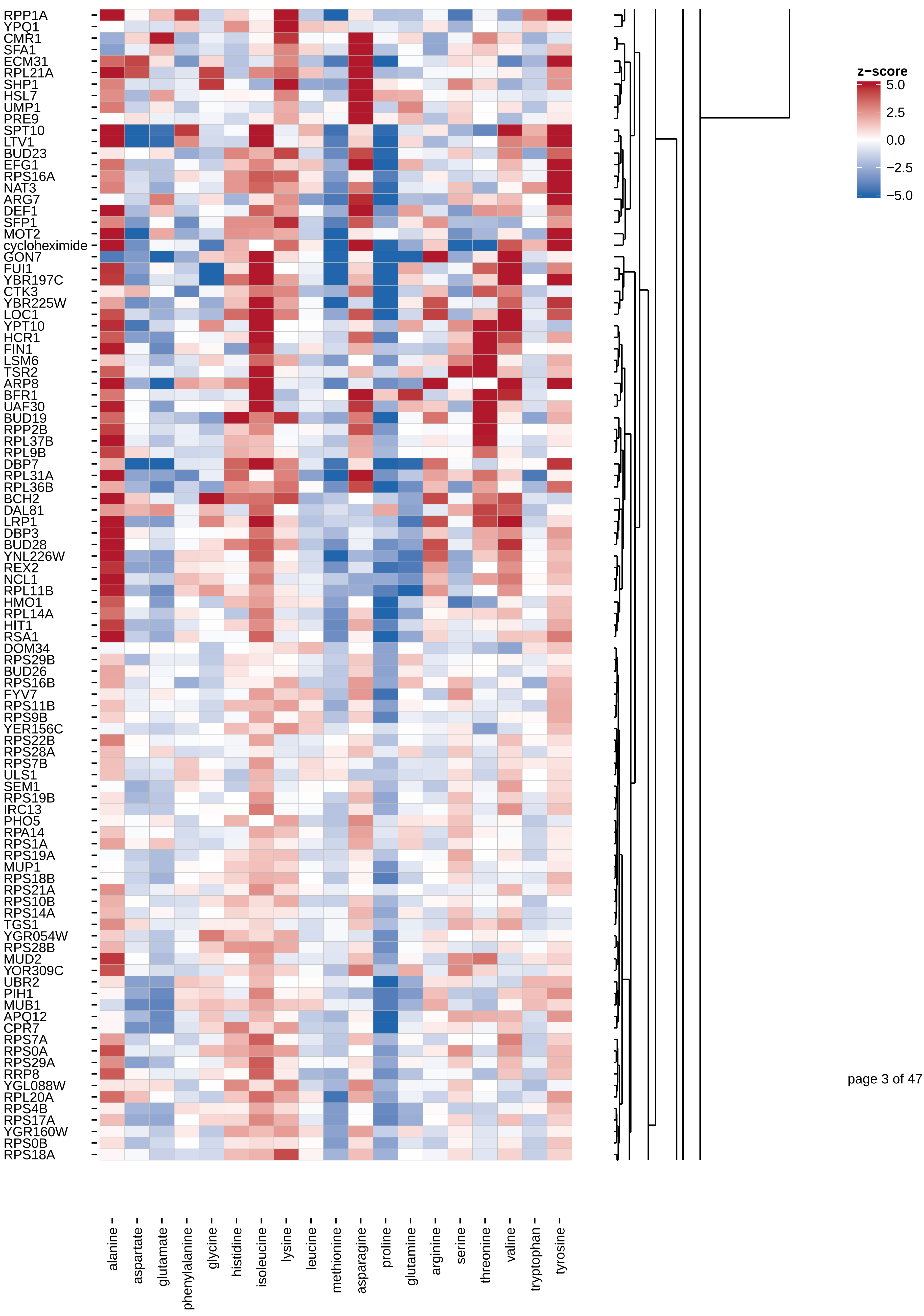
RPP1A
YPQ1
CMR1
SFA1
ECM31
RPL21A
SHP1
HSL7
UMP1
PRE9
SPT10
LTV1
BUD23
EFG1
RPS16A
NAT3
ARG7
DEF1
SFP1
MOT2
cycloheximide
GON7
FUI1
YBR197C
CTK3
YBR225W
LOC1
YPT10
HCR1
FIN1
LSM6
TSR2
ARP8
BFR1
UAF30
BUD19
RPP2B
RPL37B
RPL9B
DBP7
RPL31A
RPL36B
BCH2
DAL81
LRP1
DBP3
BUD28
YNL226W
REX2
NCL1
RPL11B
HMO1
RPL14A
HIT1
RSA1
DOM34
RPS29B
BUD26
RPS16B
FYV7
RPS11B
RPS9B
YER156C
RPS22B
RPS28A
RPS7B
ULS1
SEM1
RPS19B
IRC13
PHO5
RPA14
RPS1A
RPS19A
MUP1
RPS18B
RPS21A
RPS10B
RPS14A
TGS1
YGR054W
RPS28B
MUD2
YOR309C
UBR2
PIH1
MUB1
APQ12
CPR7
RPS7A
RPS0A
RPS29A
RRP8
YGL088W
RPL20A
RPS4B
RPS17A
YGR160W
RPS0B
RPS18A
alanine
aspartate
glutamate
phenylalanine
glycine
histidine
isoleucine
lysine
leucine
methionine
asparagine
proline
glutamine
arginine
serine
threonine
valine
tryptophan
tyrosine
z−score
5.0
2.5
0.0
−2.5
−5.0

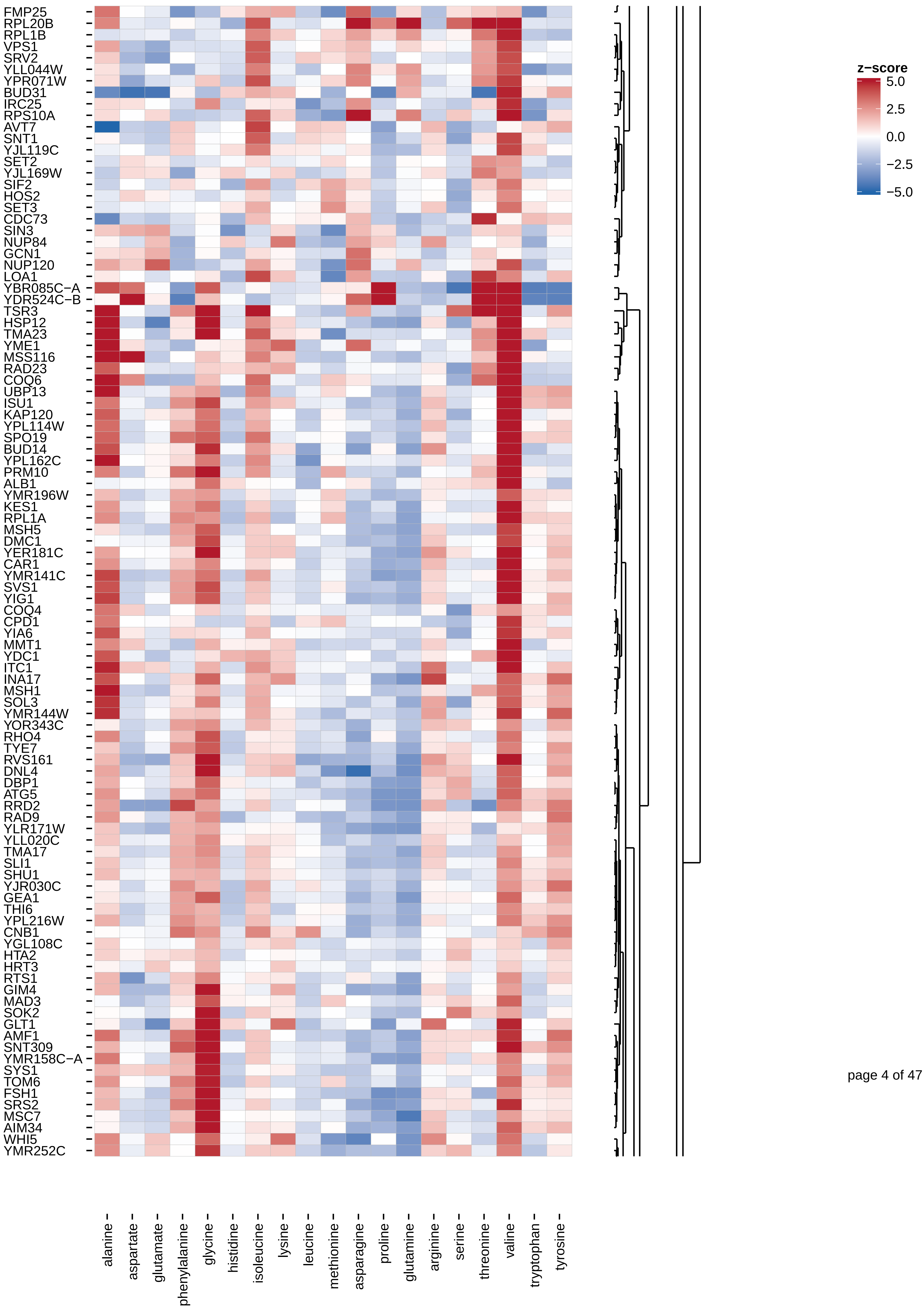
FMP25
RPL20B
RPL1B
VPS1
SRV2
YLL044W
YPR071W
BUD31
IRC25
RPS10A
AVT7
SNT1
YJL119C
SET2
YJL169W
SIF2
HOS2
SET3
CDC73
SIN3
NUP84
GCN1
NUP120
LOA1
YBR085C−A
YDR524C−B
TSR3
HSP12
TMA23
YME1
MSS116
RAD23
COQ6
UBP13
ISU1
KAP120
YPL114W
SPO19
BUD14
YPL162C
PRM10
ALB1
YMR196W
KES1
RPL1A
MSH5
DMC1
YER181C
CAR1
YMR141C
SVS1
YIG1
COQ4
CPD1
YIA6
MMT1
YDC1
ITC1
INA17
MSH1
SOL3
YMR144W
YOR343C
RHO4
TYE7
RVS161
DNL4
DBP1
ATG5
RRD2
RAD9
YLR171W
YLL020C
TMA17
SLI1
SHU1
YJR030C
GEA1
THI6
YPL216W
CNB1
YGL108C
HTA2
HRT3
RTS1
GIM4
MAD3
SOK2
GLT1
AMF1
SNT309
YMR158C−A
SYS1
TOM6
FSH1
SRS2
MSC7
AIM34
WHI5
YMR252C
alanine
aspartate
glutamate
phenylalanine
glycine
histidine
isoleucine
lysine
leucine
methionine
asparagine
proline
glutamine
arginine
serine
threonine
valine
tryptophan
tyrosine
z−score
5.0
2.5
0.0
−2.5
−5.0

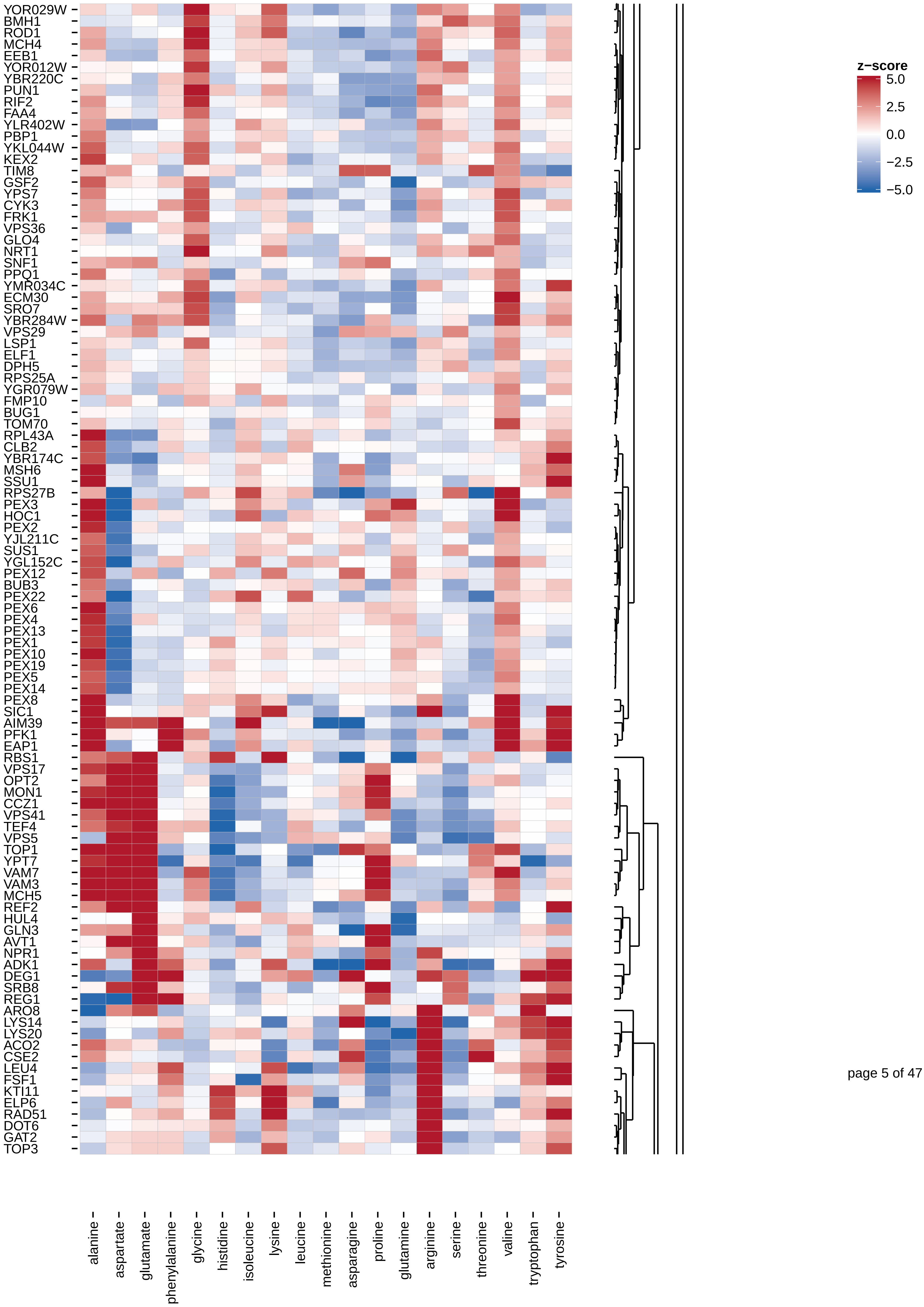

YOR029W
BMH1
ROD1
MCH4
EEB1
YOR012W
YBR220C
PUN1
RIF2
FAA4
YLR402W
PBP1
YKL044W
KEX2
TIM8
GSF2
YPS7
CYK3
FRK1
VPS36
GLO4
NRT1
SNF1
PPQ1
YMR034C
ECM30
SRO7
YBR284W
VPS29
LSP1
ELF1
DPH5
RPS25A
YGR079W
FMP10
BUG1
TOM70
RPL43A
CLB2
YBR174C
MSH6
SSU1
RPS27B
PEX3
HOC1
PEX2
YJL211C
SUS1
YGL152C
PEX12
BUB3
PEX22
PEX6
PEX4
PEX13
PEX1
PEX10
PEX19
PEX5
PEX14
PEX8
SIC1
AIM39
PFK1
EAP1
RBS1
VPS17
OPT2
MON1
CCZ1
VPS41
TEF4
VPS5
TOP1
YPT7
VAM7
VAM3
MCH5
REF2
HUL4
GLN3
AVT1
NPR1
ADK1
DEG1
SRB8
REG1
ARO8
LYS14
LYS20
ACO2
CSE2
LEU4
FSF1
KTI11
ELP6
RAD51
DOT6
GAT2
TOP3
alanine
aspartate
glutamate
phenylalanine
glycine
histidine
isoleucine
lysine
leucine
methionine
asparagine
proline
glutamine
arginine
serine
threonine
valine
tryptophan
tyrosine
z−score
5.0
2.5
0.0
−2.5
−5.0

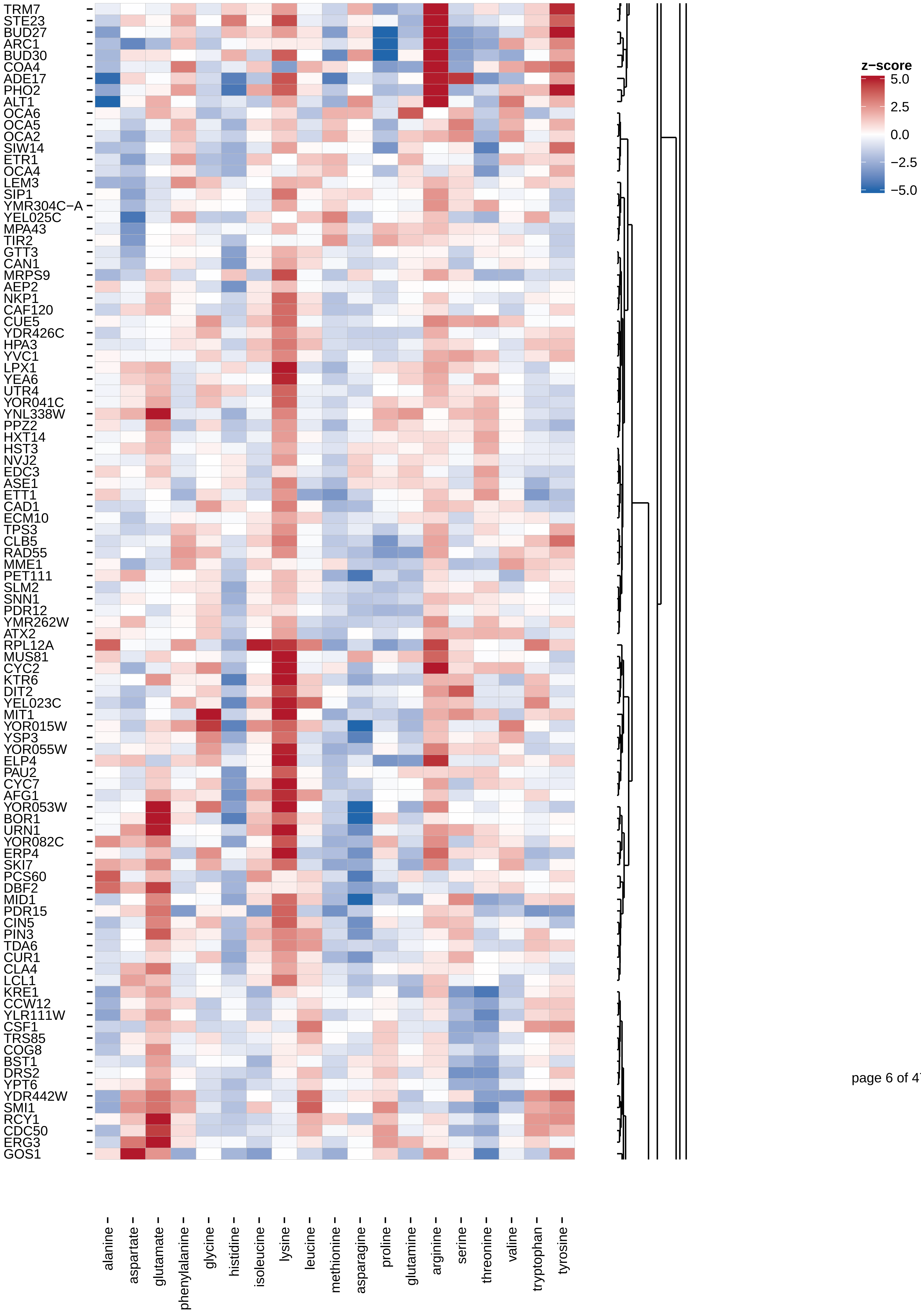
TRM7
STE23
BUD27
ARC1
BUD30
COA4
ADE17
PHO2
ALT1
OCA6
OCA5
OCA2
SIW14
ETR1
OCA4
LEM3
SIP1
YMR304C−A
YEL025C
MPA43
TIR2
GTT3
CAN1
MRPS9
AEP2
NKP1
CAF120
CUE5
YDR426C
HPA3
YVC1
LPX1
YEA6
UTR4
YOR041C
YNL338W
PPZ2
HXT14
HST3
NVJ2
EDC3
ASE1
ETT1
CAD1
ECM10
TPS3
CLB5
RAD55
MME1
PET111
SLM2
SNN1
PDR12
YMR262W
ATX2
RPL12A
MUS81
CYC2
KTR6
DIT2
YEL023C
MIT1
YOR015W
YSP3
YOR055W
ELP4
PAU2
CYC7
AFG1
YOR053W
BOR1
URN1
YOR082C
ERP4
SKI7
PCS60
DBF2
MID1
PDR15
CIN5
PIN3
TDA6
CUR1
CLA4
LCL1
KRE1
CCW12
YLR111W
CSF1
TRS85
COG8
BST1
DRS2
YPT6
YDR442W
SMI1
RCY1
CDC50
ERG3
GOS1
alanine
aspartate
glutamate
phenylalanine
glycine
histidine
isoleucine
lysine
leucine
methionine
asparagine
proline
glutamine
arginine
serine
threonine
valine
tryptophan
tyrosine
z−score
5.0
2.5
0.0
−2.5
−5.0

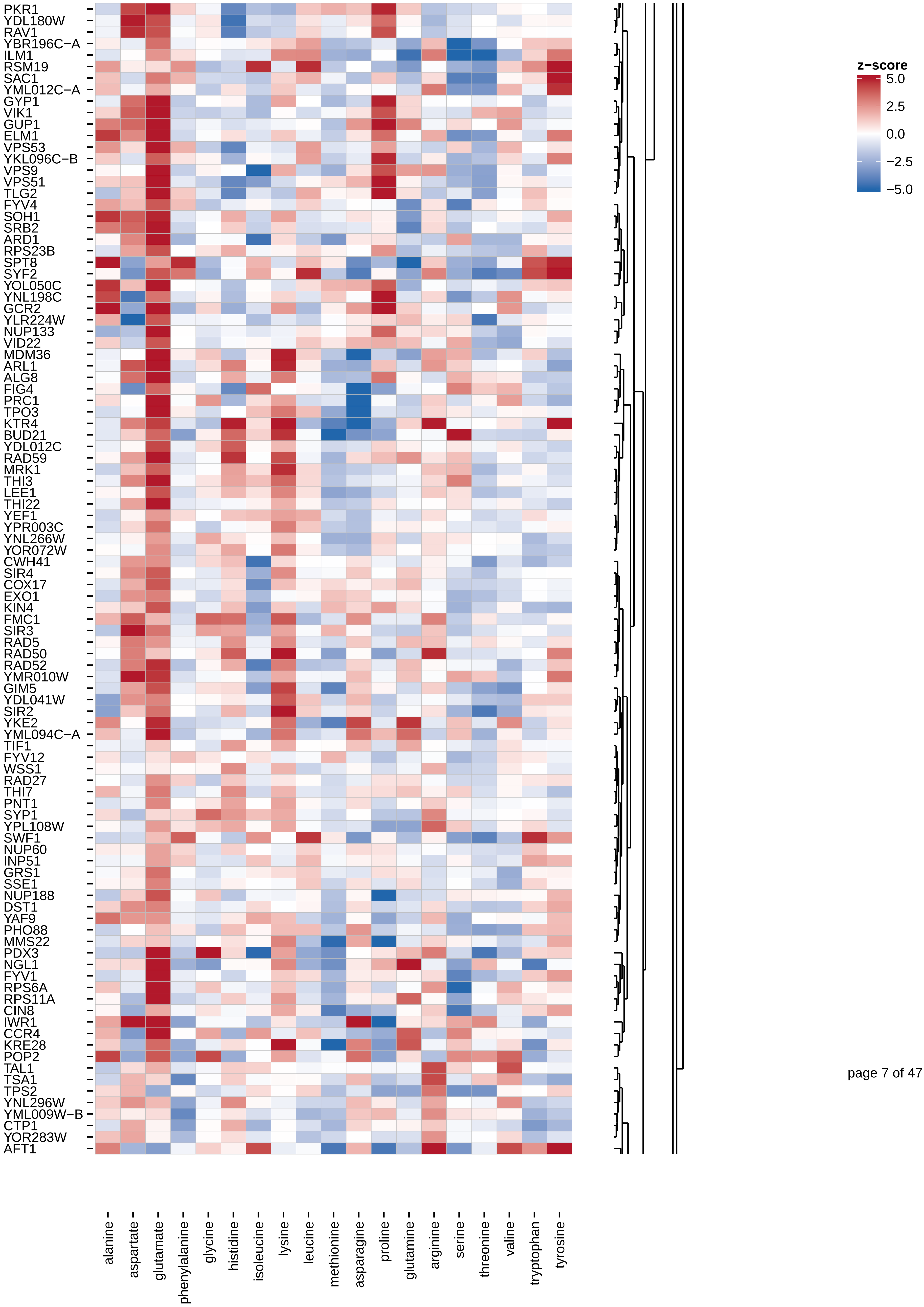
z−score
5.0
2.5
0.0
−2.5
−5.0
PKR1
YDL180W
RAV1
YBR196C−A
ILM1
RSM19
SAC1
YML012C−A
GYP1
VIK1
GUP1
ELM1
VPS53
YKL096C−B
VPS9
VPS51
TLG2
FYV4
SOH1
SRB2
ARD1
RPS23B
SPT8
SYF2
YOL050C
YNL198C
GCR2
YLR224W
NUP133
VID22
MDM36
ARL1
ALG8
FIG4
PRC1
TPO3
KTR4
BUD21
YDL012C
RAD59
MRK1
THI3
LEE1
THI22
YEF1
YPR003C
YNL266W
YOR072W
CWH41
SIR4
COX17
EXO1
KIN4
FMC1
SIR3
RAD5
RAD50
RAD52
YMR010W
GIM5
YDL041W
SIR2
YKE2
YML094C−A
TIF1
FYV12
WSS1
RAD27
THI7
PNT1
SYP1
YPL108W
SWF1
NUP60
INP51
GRS1
SSE1
NUP188
DST1
YAF9
PHO88
MMS22
PDX3
NGL1
FYV1
RPS6A
RPS11A
CIN8
IWR1
CCR4
KRE28
POP2
TAL1
TSA1
TPS2
YNL296W
YML009W−B
CTP1
YOR283W
AFT1
alanine
aspartate
glutamate
phenylalanine
glycine
histidine
isoleucine
lysine
leucine
methionine
asparagine
proline
glutamine
arginine
serine
threonine
valine
tryptophan
tyrosine

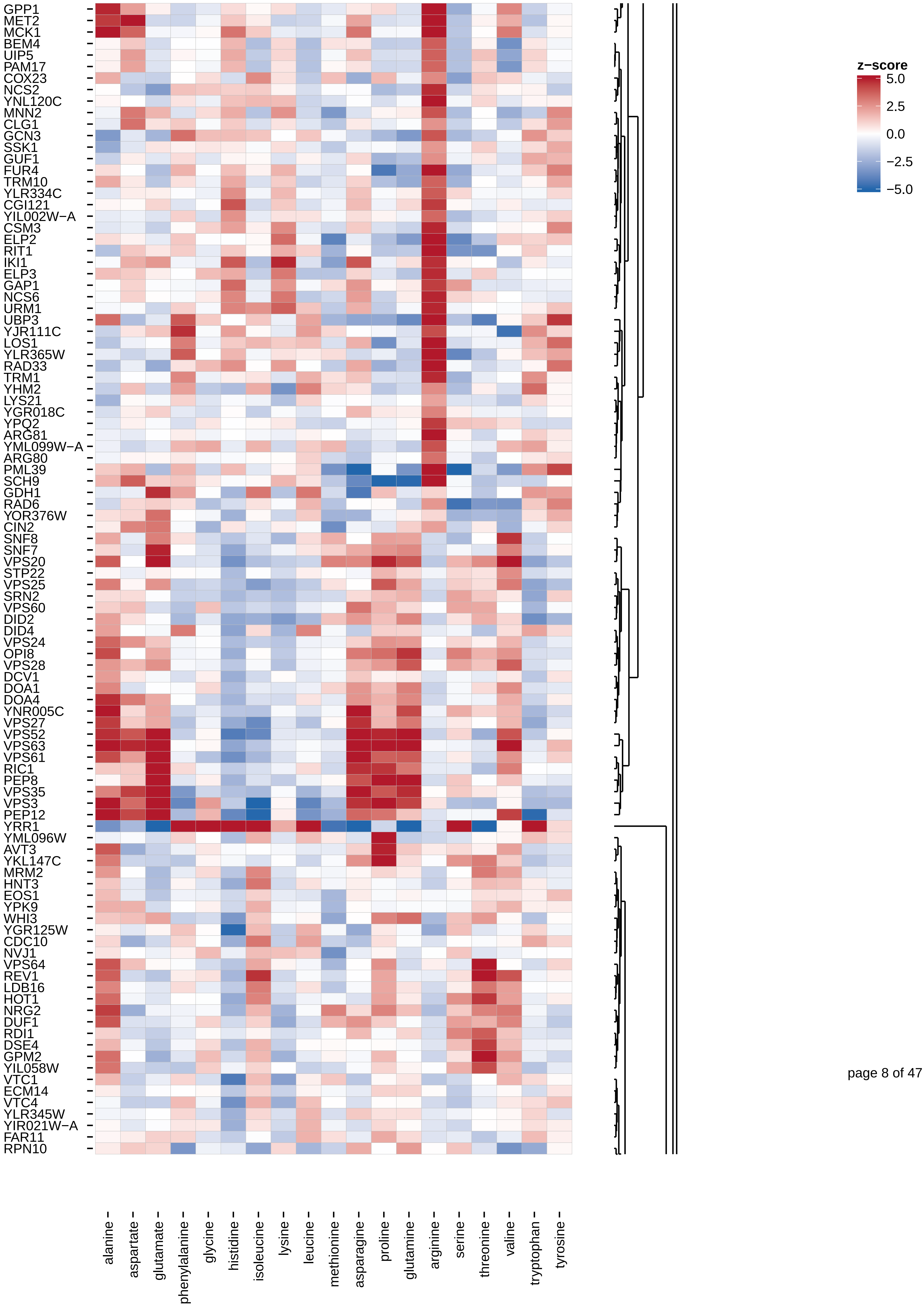
z−score
5.0
2.5
0.0
−2.5
−5.0
GPP1
MET2
MCK1
BEM4
UIP5
PAM17
COX23
NCS2
YNL120C
MNN2
CLG1
GCN3
SSK1
GUF1
FUR4
TRM10
YLR334C
CGI121
YIL002W−A
CSM3
ELP2
RIT1
IKI1
ELP3
GAP1
NCS6
URM1
UBP3
YJR111C
LOS1
YLR365W
RAD33
TRM1
YHM2
LYS21
YGR018C
YPQ2
ARG81
YML099W−A
ARG80
PML39
SCH9
GDH1
RAD6
YOR376W
CIN2
SNF8
SNF7
VPS20
STP22
VPS25
SRN2
VPS60
DID2
DID4
VPS24
OPI8
VPS28
DCV1
DOA1
DOA4
YNR005C
VPS27
VPS52
VPS63
VPS61
RIC1
PEP8
VPS35
VPS3
PEP12
YRR1
YML096W
AVT3
YKL147C
MRM2
HNT3
EOS1
YPK9
WHI3
YGR125W
CDC10
NVJ1
VPS64
REV1
LDB16
HOT1
NRG2
DUF1
RDI1
DSE4
GPM2
YIL058W
VTC1
ECM14
VTC4
YLR345W
YIR021W−A
FAR11
RPN10
alanine
aspartate
glutamate
phenylalanine
glycine
histidine
isoleucine
lysine
leucine
methionine
asparagine
proline
glutamine
arginine
serine
threonine
valine
tryptophan
tyrosine

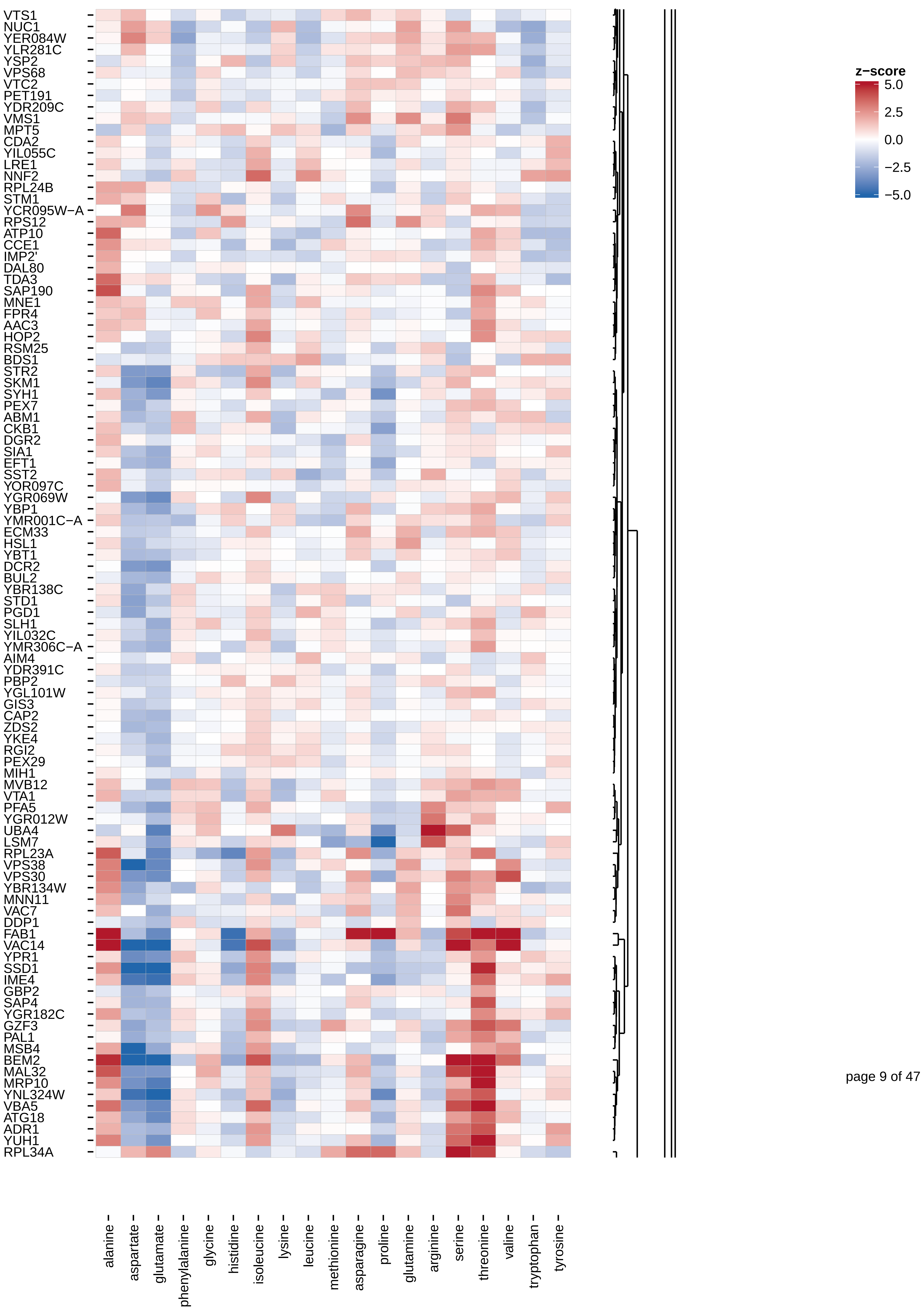

VTS1
NUC1
YER084W
YLR281C
YSP2
VPS68
VTC2
PET191
YDR209C
VMS1
MPT5
CDA2
YIL055C
LRE1
NNF2
RPL24B
STM1
YCR095W−A
RPS12
ATP10
CCE1
IMP2'
DAL80
TDA3
SAP190
MNE1
FPR4
AAC3
HOP2
RSM25
BDS1
STR2
SKM1
SYH1
PEX7
ABM1
CKB1
DGR2
SIA1
EFT1
SST2
YOR097C
YGR069W
YBP1
YMR001C−A
ECM33
HSL1
YBT1
DCR2
BUL2
YBR138C
STD1
PGD1
SLH1
YIL032C
YMR306C−A
AIM4
YDR391C
PBP2
YGL101W
GIS3
CAP2
ZDS2
YKE4
RGI2
PEX29
MIH1
MVB12
VTA1
PFA5
YGR012W
UBA4
LSM7
RPL23A
VPS38
VPS30
YBR134W
MNN11
VAC7
DDP1
FAB1
VAC14
YPR1
SSD1
IME4
GBP2
SAP4
YGR182C
GZF3
PAL1
MSB4
BEM2
MAL32
MRP10
YNL324W
VBA5
ATG18
ADR1
YUH1
RPL34A
alanine
aspartate
glutamate
phenylalanine
glycine
histidine
isoleucine
lysine
leucine
methionine
asparagine
proline
glutamine
arginine
serine
threonine
valine
tryptophan
tyrosine
z−score
5.0
2.5
0.0
−2.5
−5.0

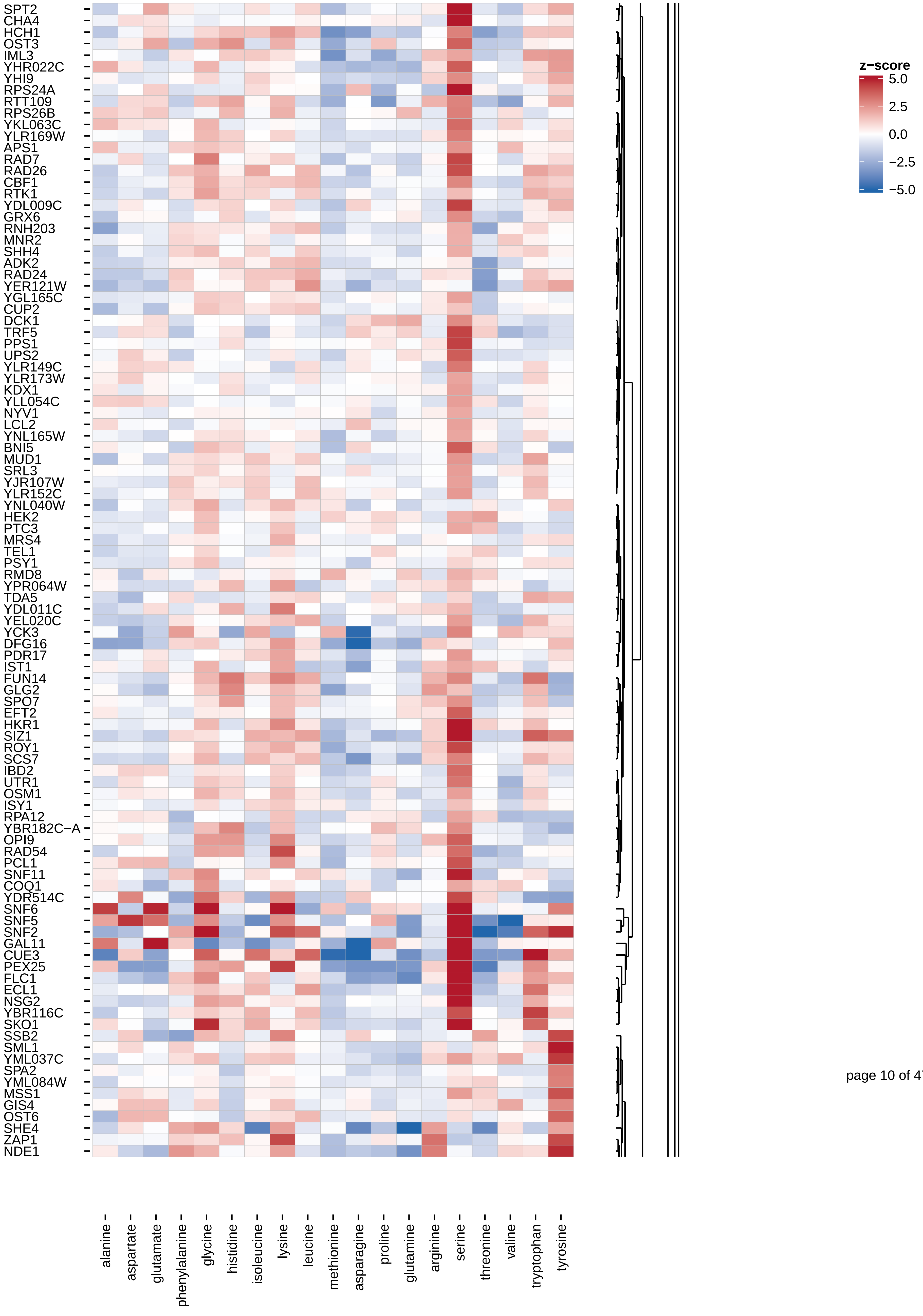
SPT2
CHA4
HCH1
OST3
IML3
YHR022C
YHI9
RPS24A
RTT109
RPS26B
YKL063C
YLR169W
APS1
RAD7
RAD26
CBF1
RTK1
YDL009C
GRX6
RNH203
MNR2
SHH4
ADK2
RAD24
YER121W
YGL165C
CUP2
DCK1
TRF5
PPS1
UPS2
YLR149C
YLR173W
KDX1
YLL054C
NYV1
LCL2
YNL165W
BNI5
MUD1
SRL3
YJR107W
YLR152C
YNL040W
HEK2
PTC3
MRS4
TEL1
PSY1
RMD8
YPR064W
TDA5
YDL011C
YEL020C
YCK3
DFG16
PDR17
IST1
FUN14
GLG2
SPO7
EFT2
HKR1
SIZ1
ROY1
SCS7
IBD2
UTR1
OSM1
ISY1
RPA12
YBR182C−A
OPI9
RAD54
PCL1
SNF11
COQ1
YDR514C
SNF6
SNF5
SNF2
GAL11
CUE3
PEX25
FLC1
ECL1
NSG2
YBR116C
SKO1
SSB2
SML1
YML037C
SPA2
YML084W
MSS1
GIS4
OST6
SHE4
ZAP1
NDE1
alanine
aspartate
glutamate
phenylalanine
glycine
histidine
isoleucine
lysine
leucine
methionine
asparagine
proline
glutamine
arginine
serine
threonine
valine
tryptophan
tyrosine
z−score
5.0
2.5
0.0
−2.5
−5.0

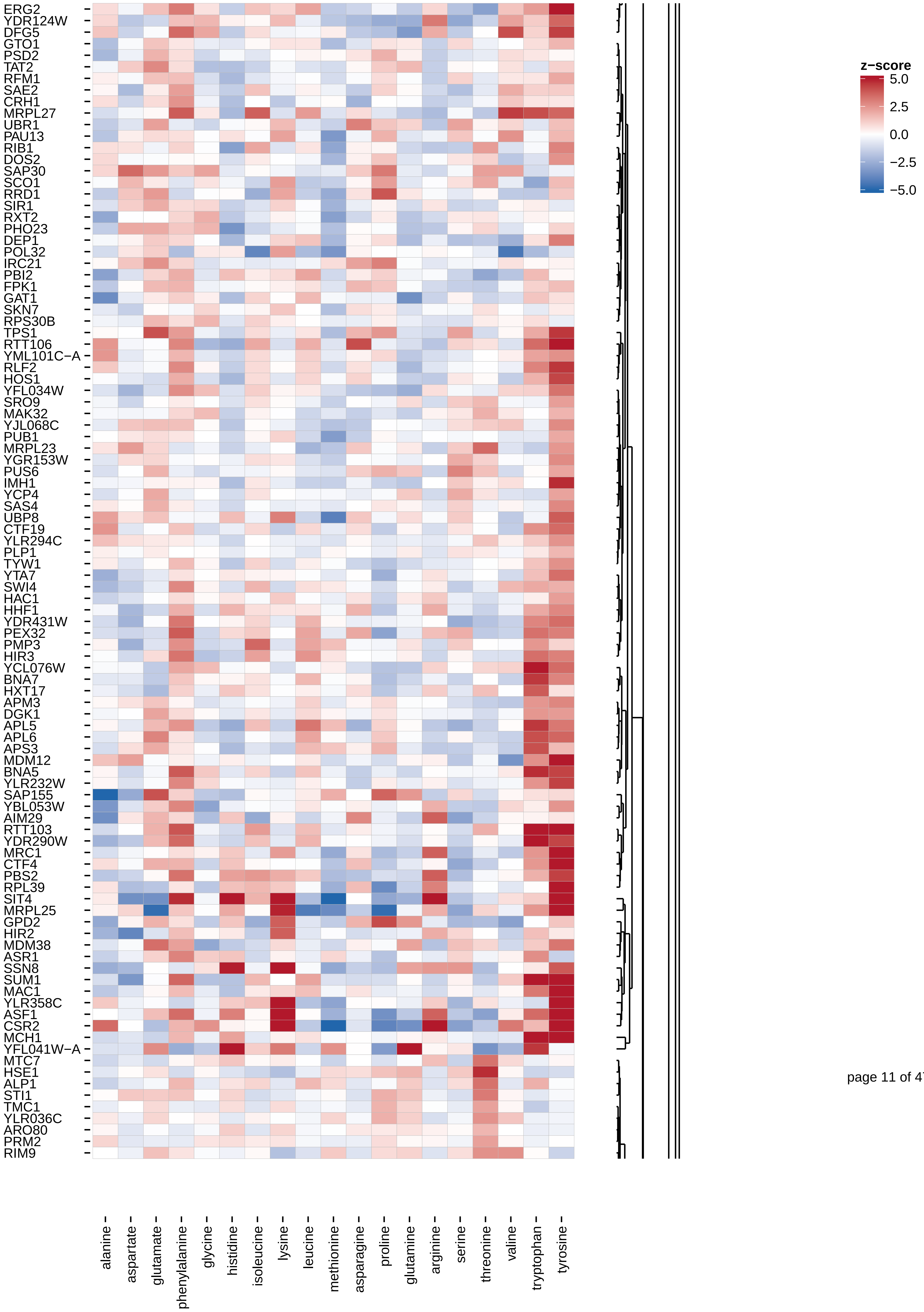

z−score
5.0
2.5
0.0
−2.5
−5.0
ERG2
YDR124W
DFG5
GTO1
PSD2
TAT2
RFM1
SAE2
CRH1
MRPL27
UBR1
PAU13
RIB1
DOS2
SAP30
SCO1
RRD1
SIR1
RXT2
PHO23
DEP1
POL32
IRC21
PBI2
FPK1
GAT1
SKN7
RPS30B
TPS1
RTT106
YML101C−A
RLF2
HOS1
YFL034W
SRO9
MAK32
YJL068C
PUB1
MRPL23
YGR153W
PUS6
IMH1
YCP4
SAS4
UBP8
CTF19
YLR294C
PLP1
TYW1
YTA7
SWI4
HAC1
HHF1
YDR431W
PEX32
PMP3
HIR3
YCL076W
BNA7
HXT17
APM3
DGK1
APL5
APL6
APS3
MDM12
BNA5
YLR232W
SAP155
YBL053W
AIM29
RTT103
YDR290W
MRC1
CTF4
PBS2
RPL39
SIT4
MRPL25
GPD2
HIR2
MDM38
ASR1
SSN8
SUM1
MAC1
YLR358C
ASF1
CSR2
MCH1
YFL041W−A
MTC7
HSE1
ALP1
STI1
TMC1
YLR036C
ARO80
PRM2
RIM9
alanine
aspartate
glutamate
phenylalanine
glycine
histidine
isoleucine
lysine
leucine
methionine
asparagine
proline
glutamine
arginine
serine
threonine
valine
tryptophan
tyrosine

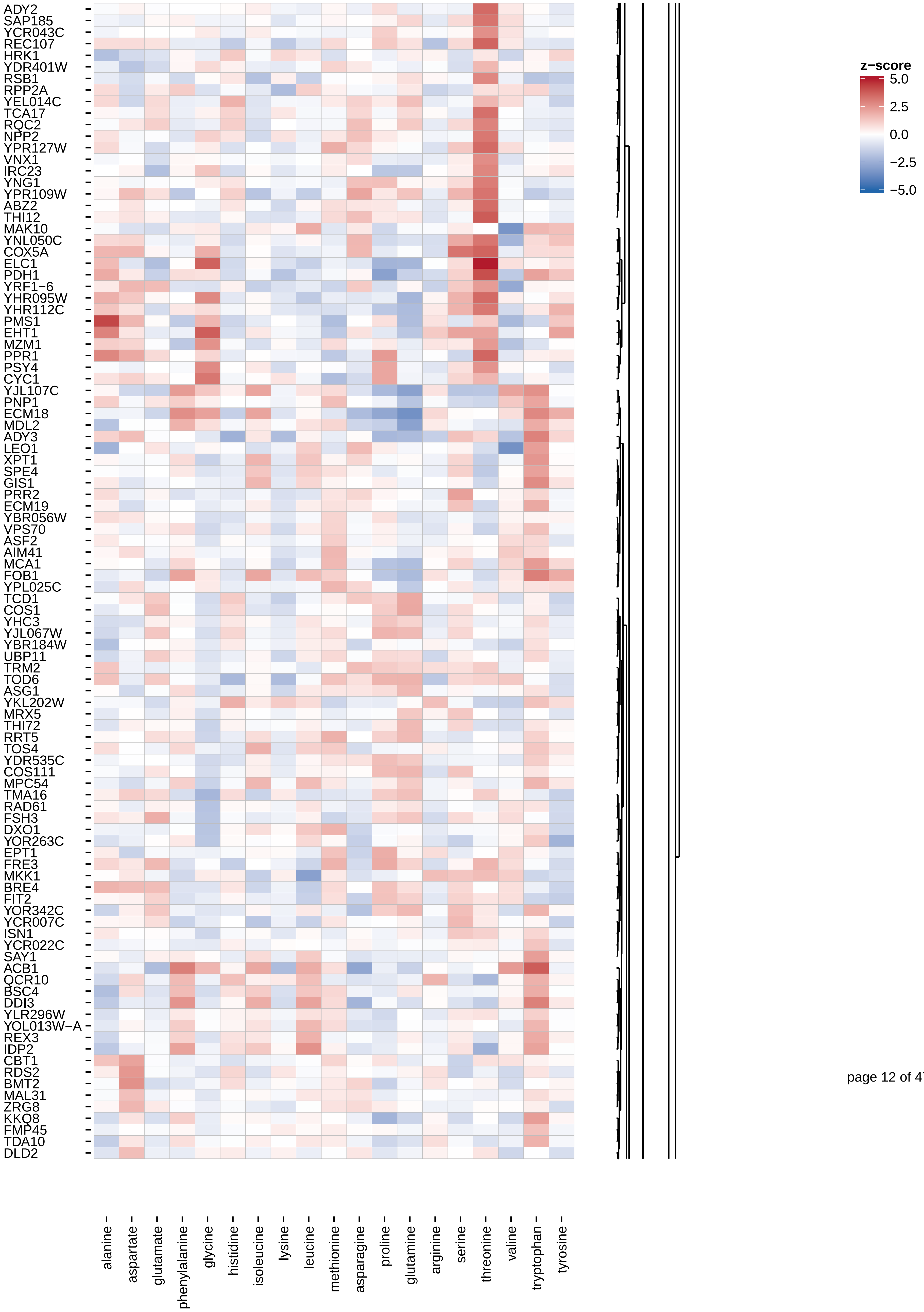
z−score
5.0
2.5
0.0
−2.5
−5.0
ADY2
SAP185
YCR043C
REC107
HRK1
YDR401W
RSB1
RPP2A
YEL014C
TCA17
RQC2
NPP2
YPR127W
VNX1
IRC23
YNG1
YPR109W
ABZ2
THI12
MAK10
YNL050C
COX5A
ELC1
PDH1
YRF1−6
YHR095W
YHR112C
PMS1
EHT1
MZM1
PPR1
PSY4
CYC1
YJL107C
PNP1
ECM18
MDL2
ADY3
LEO1
XPT1
SPE4
GIS1
PRR2
ECM19
YBR056W
VPS70
ASF2
AIM41
MCA1
FOB1
YPL025C
TCD1
COS1
YHC3
YJL067W
YBR184W
UBP11
TRM2
TOD6
ASG1
YKL202W
MRX5
THI72
RRT5
TOS4
YDR535C
COS111
MPC54
TMA16
RAD61
FSH3
DXO1
YOR263C
EPT1
FRE3
MKK1
BRE4
FIT2
YOR342C
YCR007C
ISN1
YCR022C
SAY1
ACB1
QCR10
BSC4
DDI3
YLR296W
YOL013W−A
REX3
IDP2
CBT1
RDS2
BMT2
MAL31
ZRG8
KKQ8
FMP45
TDA10
DLD2
alanine
aspartate
glutamate
phenylalanine
glycine
histidine
isoleucine
lysine
leucine
methionine
asparagine
proline
glutamine
arginine
serine
threonine
valine
tryptophan
tyrosine

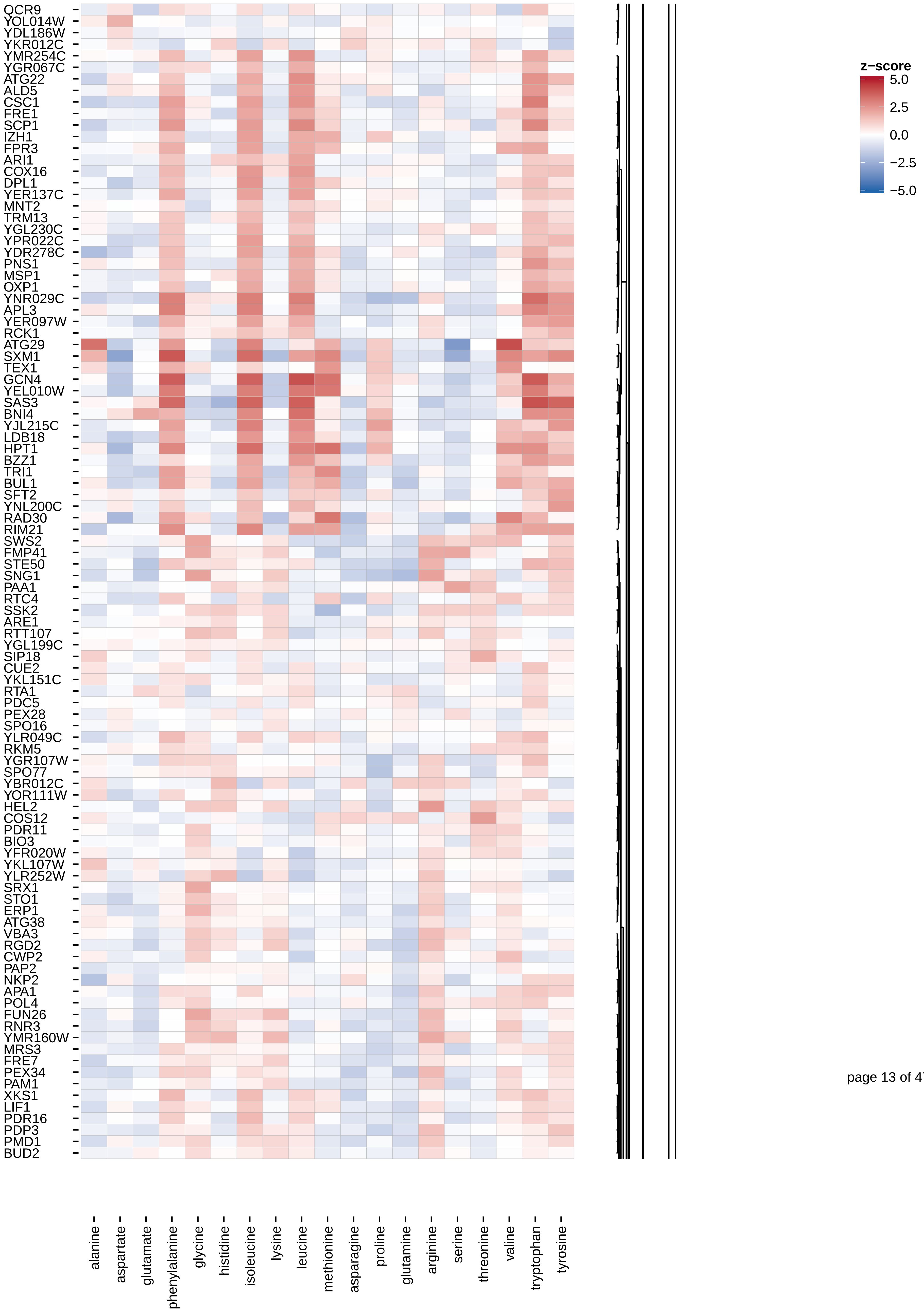

z−score
5.0
2.5
0.0
−2.5
−5.0
QCR9
YOL014W
YDL186W
YKR012C
YMR254C
YGR067C
ATG22
ALD5
CSC1
FRE1
SCP1
IZH1
FPR3
ARI1
COX16
DPL1
YER137C
MNT2
TRM13
YGL230C
YPR022C
YDR278C
PNS1
MSP1
OXP1
YNR029C
APL3
YER097W
RCK1
ATG29
SXM1
TEX1
GCN4
YEL010W
SAS3
BNI4
YJL215C
LDB18
HPT1
BZZ1
TRI1
BUL1
SFT2
YNL200C
RAD30
RIM21
SWS2
FMP41
STE50
SNG1
PAA1
RTC4
SSK2
ARE1
RTT107
YGL199C
SIP18
CUE2
YKL151C
RTA1
PDC5
PEX28
SPO16
YLR049C
RKM5
YGR107W
SPO77
YBR012C
YOR111W
HEL2
COS12
PDR11
BIO3
YFR020W
YKL107W
YLR252W
SRX1
STO1
ERP1
ATG38
VBA3
RGD2
CWP2
PAP2
NKP2
APA1
POL4
FUN26
RNR3
YMR160W
MRS3
FRE7
PEX34
PAM1
XKS1
LIF1
PDR16
PDP3
PMD1
BUD2
alanine
aspartate
glutamate
phenylalanine
glycine
histidine
isoleucine
lysine
leucine
methionine
asparagine
proline
glutamine
arginine
serine
threonine
valine
tryptophan
tyrosine

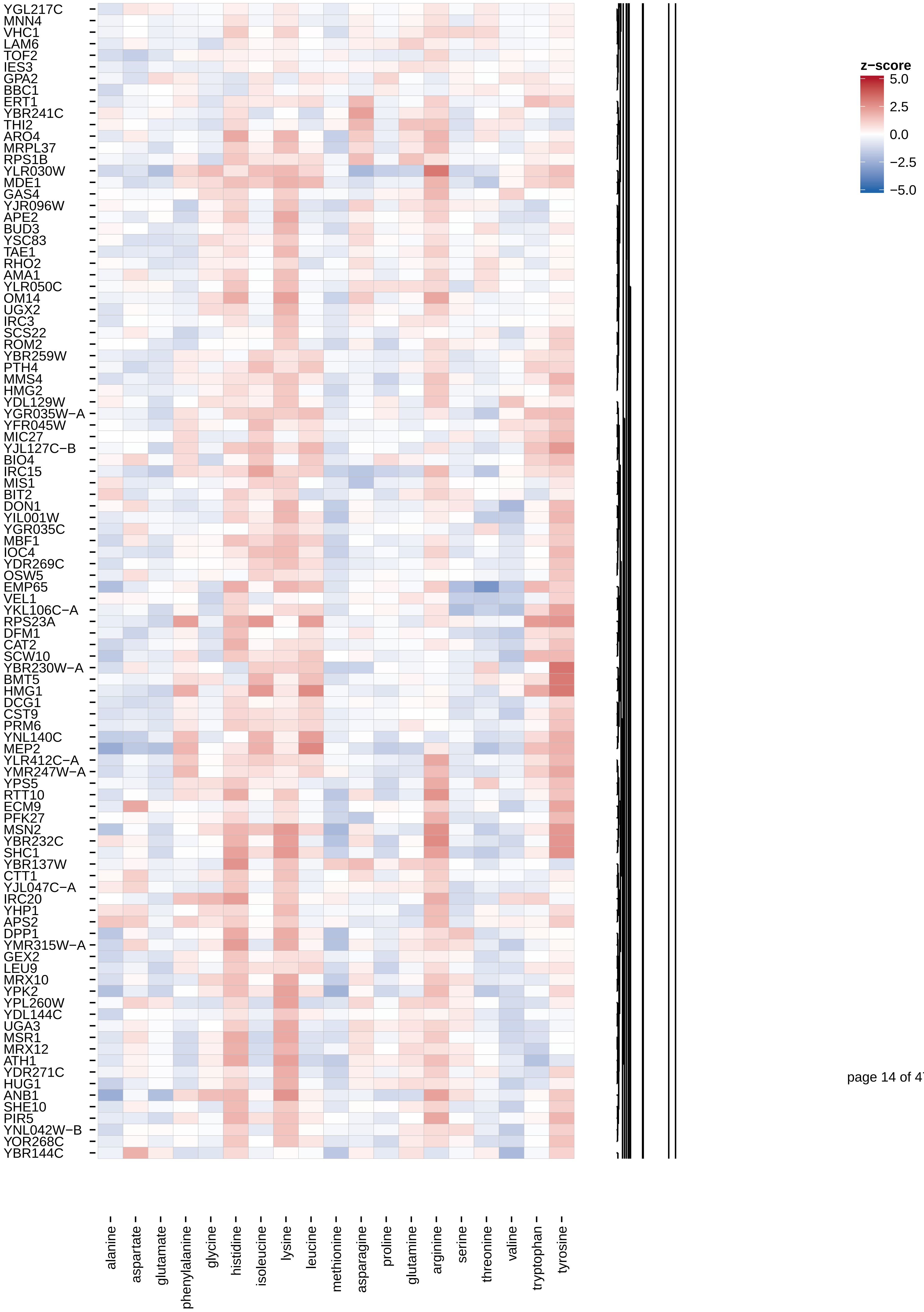
z−score
5.0
2.5
0.0
−2.5
−5.0
YGL217C
MNN4
VHC1
LAM6
TOF2
IES3
GPA2
BBC1
ERT1
YBR241C
THI2
ARO4
MRPL37
RPS1B
YLR030W
MDE1
GAS4
YJR096W
APE2
BUD3
YSC83
TAE1
RHO2
AMA1
YLR050C
OM14
UGX2
IRC3
SCS22
ROM2
YBR259W
PTH4
MMS4
HMG2
YDL129W
YGR035W−A
YFR045W
MIC27
YJL127C−B
BIO4
IRC15
MIS1
BIT2
DON1
YIL001W
YGR035C
MBF1
IOC4
YDR269C
OSW5
EMP65
VEL1
YKL106C−A
RPS23A
DFM1
CAT2
SCW10
YBR230W−A
BMT5
HMG1
DCG1
CST9
PRM6
YNL140C
MEP2
YLR412C−A
YMR247W−A
YPS5
RTT10
ECM9
PFK27
MSN2
YBR232C
SHC1
YBR137W
CTT1
YJL047C−A
IRC20
YHP1
APS2
DPP1
YMR315W−A
GEX2
LEU9
MRX10
YPK2
YPL260W
YDL144C
UGA3
MSR1
MRX12
ATH1
YDR271C
HUG1
ANB1
SHE10
PIR5
YNL042W−B
YOR268C
YBR144C
alanine
aspartate
glutamate
phenylalanine
glycine
histidine
isoleucine
lysine
leucine
methionine
asparagine
proline
glutamine
arginine
serine
threonine
valine
tryptophan
tyrosine

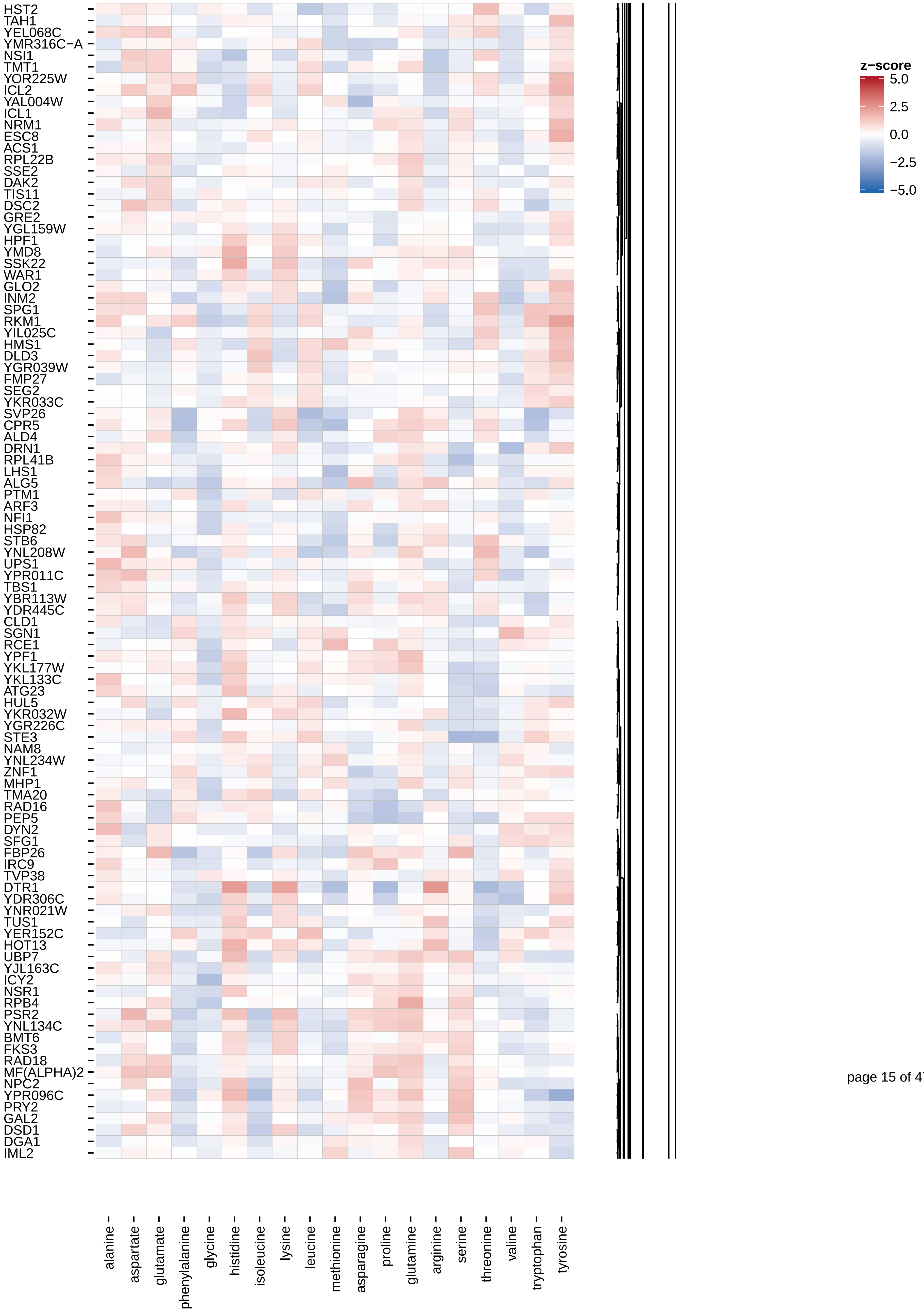
HST2
TAH1
YEL068C
YMR316C−A
NSI1
TMT1
YOR225W
ICL2
YAL004W
ICL1
NRM1
ESC8
ACS1
RPL22B
SSE2
DAK2
TIS11
DSC2
GRE2
YGL159W
HPF1
YMD8
SSK22
WAR1
GLO2
INM2
SPG1
RKM1
YIL025C
HMS1
DLD3
YGR039W
FMP27
SEG2
YKR033C
SVP26
CPR5
ALD4
DRN1
RPL41B
LHS1
ALG5
PTM1
ARF3
NFI1
HSP82
STB6
YNL208W
UPS1
YPR011C
TBS1
YBR113W
YDR445C
CLD1
SGN1
RCE1
YPF1
YKL177W
YKL133C
ATG23
HUL5
YKR032W
YGR226C
STE3
NAM8
YNL234W
ZNF1
MHP1
TMA20
RAD16
PEP5
DYN2
SFG1
FBP26
IRC9
TVP38
DTR1
YDR306C
YNR021W
TUS1
YER152C
HOT13
UBP7
YJL163C
ICY2
NSR1
RPB4
PSR2
YNL134C
BMT6
FKS3
RAD18
MF(ALPHA)2
NPC2
YPR096C
PRY2
GAL2
DSD1
DGA1
IML2
alanine
aspartate
glutamate
phenylalanine
glycine
histidine
isoleucine
lysine
leucine
methionine
asparagine
proline
glutamine
arginine
serine
threonine
valine
tryptophan
tyrosine
z−score
5.0
2.5
0.0
−2.5
−5.0

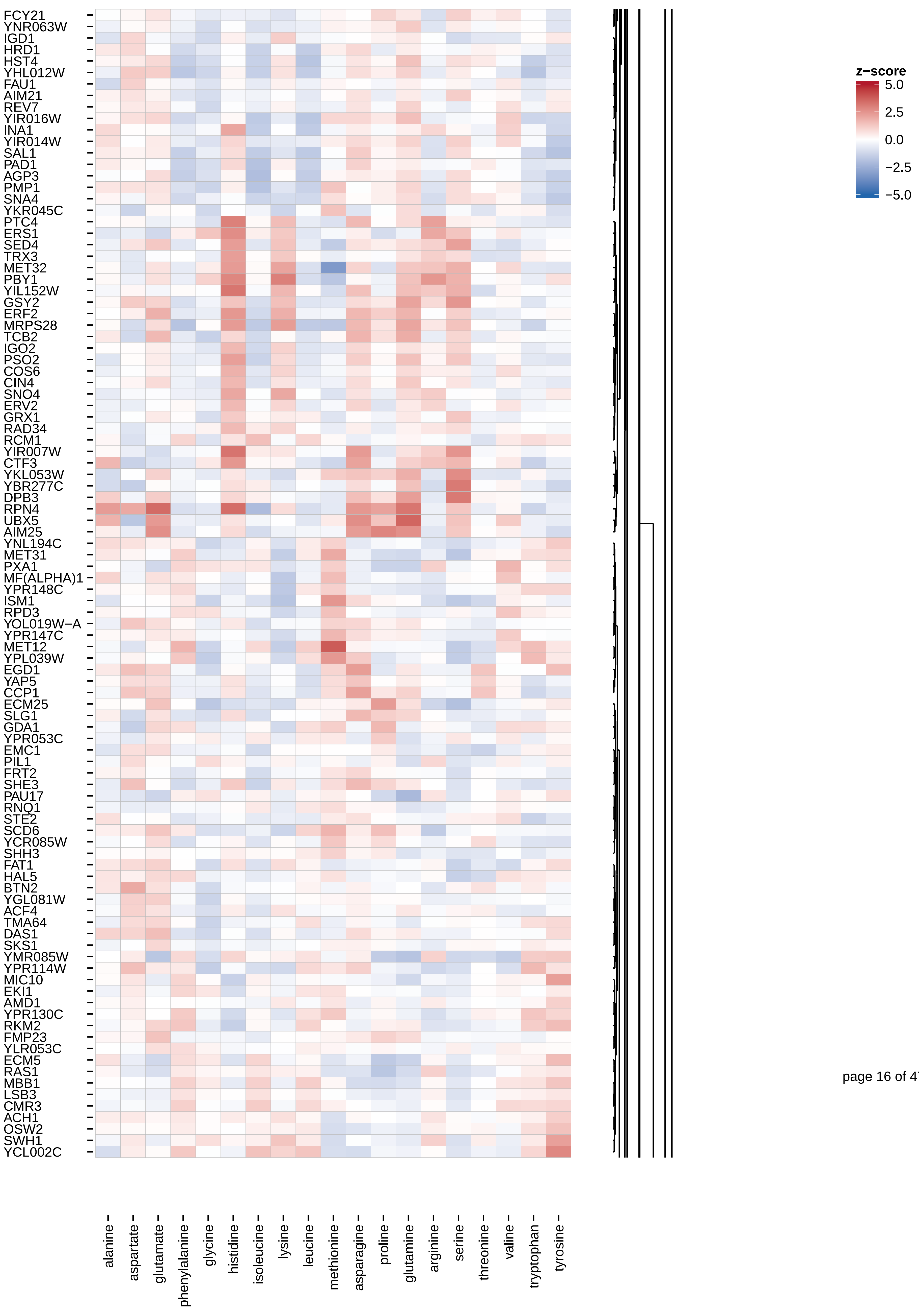
FCY21
YNR063W
IGD1
HRD1
HST4
YHL012W
FAU1
AIM21
REV7
YIR016W
INA1
YIR014W
SAL1
PAD1
AGP3
PMP1
SNA4
YKR045C
PTC4
ERS1
SED4
TRX3
MET32
PBY1
YIL152W
GSY2
ERF2
MRPS28
TCB2
IGO2
PSO2
COS6
CIN4
SNO4
ERV2
GRX1
RAD34
RCM1
YIR007W
CTF3
YKL053W
YBR277C
DPB3
RPN4
UBX5
AIM25
YNL194C
MET31
PXA1
MF(ALPHA)1
YPR148C
ISM1
RPD3
YOL019W−A
YPR147C
MET12
YPL039W
EGD1
YAP5
CCP1
ECM25
SLG1
GDA1
YPR053C
EMC1
PIL1
FRT2
SHE3
PAU17
RNQ1
STE2
SCD6
YCR085W
SHH3
FAT1
HAL5
BTN2
YGL081W
ACF4
TMA64
DAS1
SKS1
YMR085W
YPR114W
MIC10
EKI1
AMD1
YPR130C
RKM2
FMP23
YLR053C
ECM5
RAS1
MBB1
LSB3
CMR3
ACH1
OSW2
SWH1
YCL002C
alanine
aspartate
glutamate
phenylalanine
glycine
histidine
isoleucine
lysine
leucine
methionine
asparagine
proline
glutamine
arginine
serine
threonine
valine
tryptophan
tyrosine
z−score
5.0
2.5
0.0
−2.5
−5.0

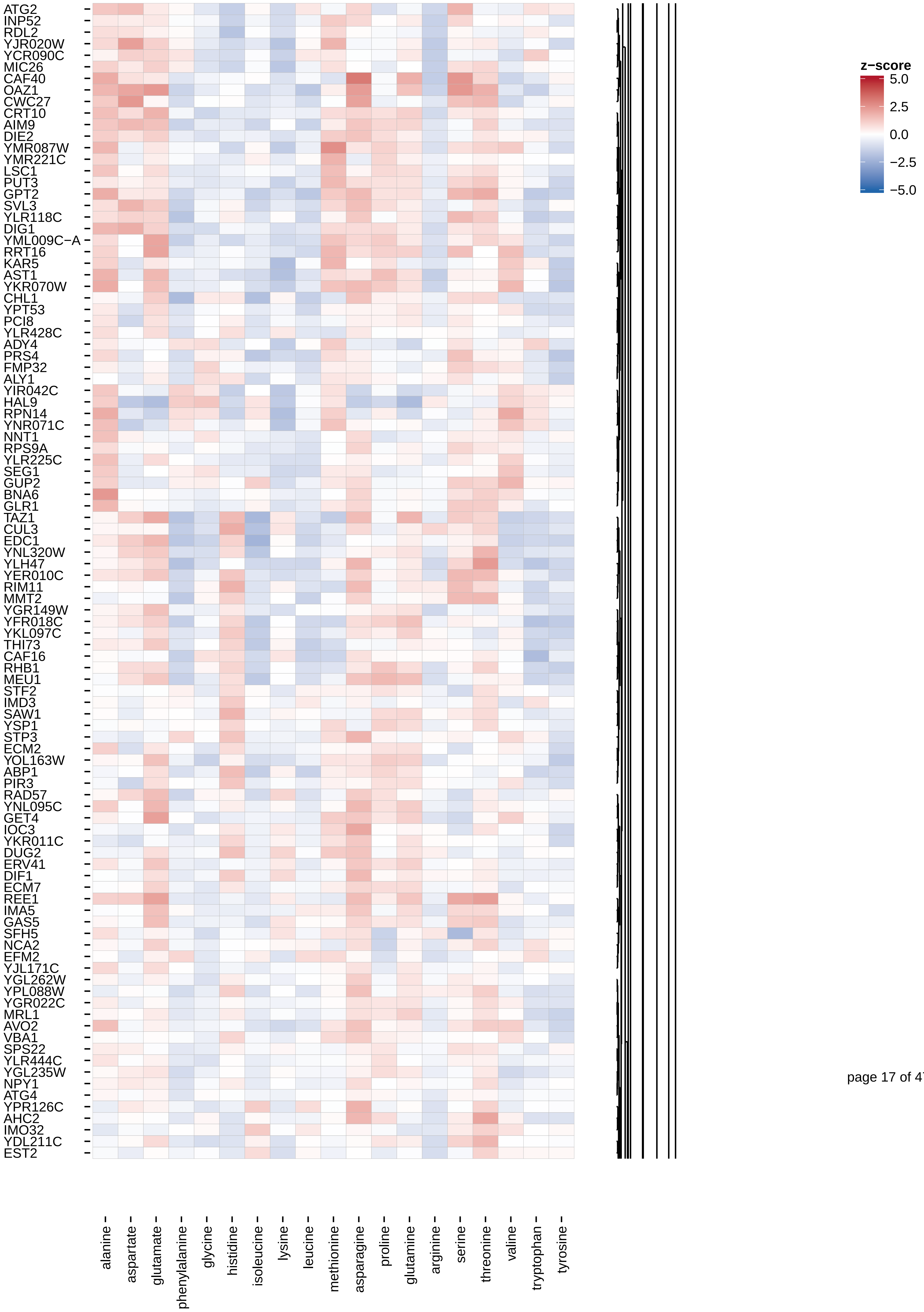
ATG2
INP52
RDL2
YJR020W
YCR090C
MIC26
CAF40
OAZ1
CWC27
CRT10
AIM9
DIE2
YMR087W
YMR221C
LSC1
PUT3
GPT2
SVL3
YLR118C
DIG1
YML009C−A
RRT16
KAR5
AST1
YKR070W
CHL1
YPT53
PCI8
YLR428C
ADY4
PRS4
FMP32
ALY1
YIR042C
HAL9
RPN14
YNR071C
NNT1
RPS9A
YLR225C
SEG1
GUP2
BNA6
GLR1
TAZ1
CUL3
EDC1
YNL320W
YLH47
YER010C
RIM11
MMT2
YGR149W
YFR018C
YKL097C
THI73
CAF16
RHB1
MEU1
STF2
IMD3
SAW1
YSP1
STP3
ECM2
YOL163W
ABP1
PIR3
RAD57
YNL095C
GET4
IOC3
YKR011C
DUG2
ERV41
DIF1
ECM7
REE1
IMA5
GAS5
SFH5
NCA2
EFM2
YJL171C
YGL262W
YPL088W
YGR022C
MRL1
AVO2
VBA1
SPS22
YLR444C
YGL235W
NPY1
ATG4
YPR126C
AHC2
IMO32
YDL211C
EST2
alanine
aspartate
glutamate
phenylalanine
glycine
histidine
isoleucine
lysine
leucine
methionine
asparagine
proline
glutamine
arginine
serine
threonine
valine
tryptophan
tyrosine
z−score
5.0
2.5
0.0
−2.5
−5.0

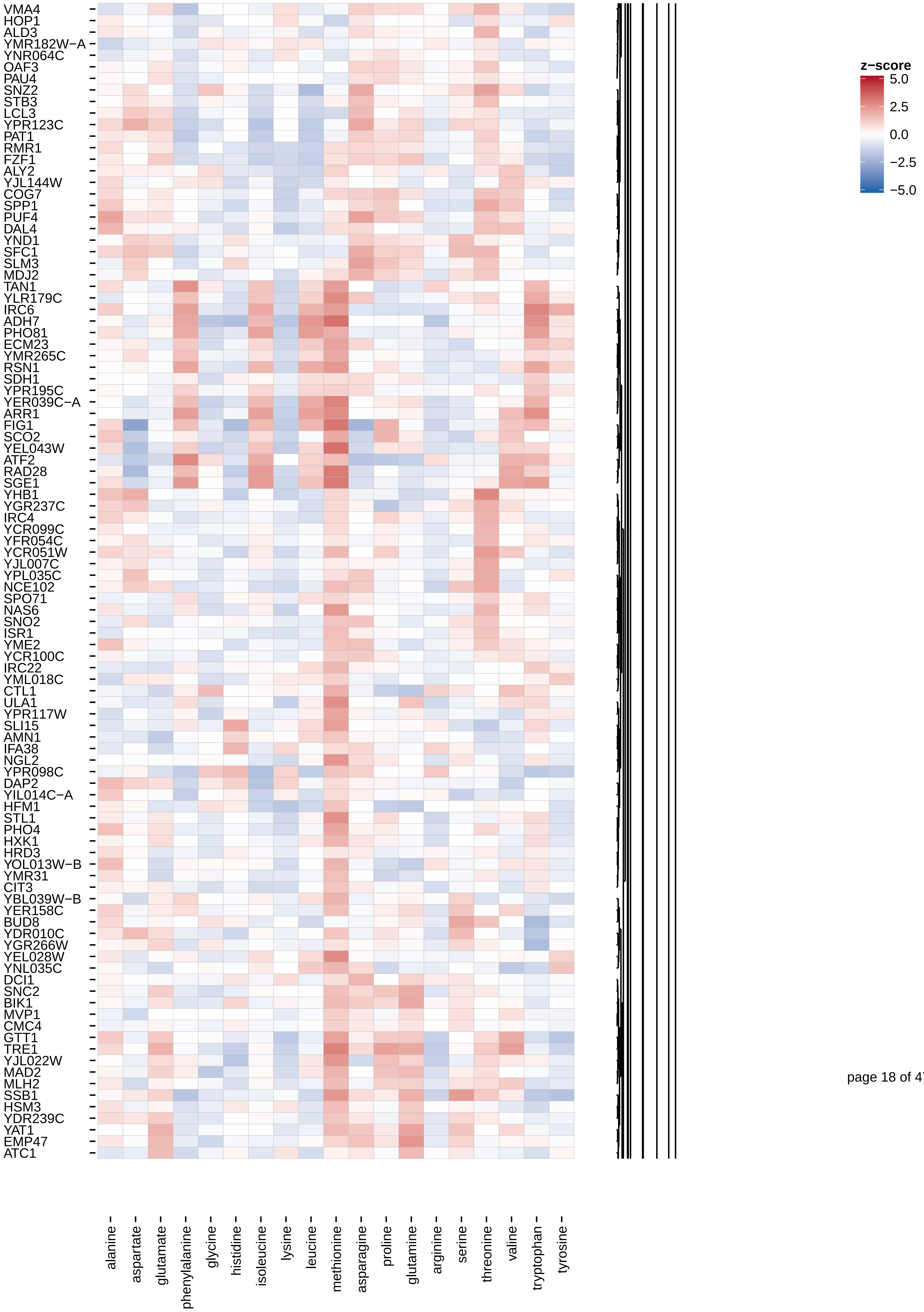
z−score
5.0
2.5
0.0
−2.5
−5.0
VMA4
HOP1
ALD3
YMR182W−A
YNR064C
OAF3
PAU4
SNZ2
STB3
LCL3
YPR123C
PAT1
RMR1
FZF1
ALY2
YJL144W
COG7
SPP1
PUF4
DAL4
YND1
SFC1
SLM3
MDJ2
TAN1
YLR179C
IRC6
ADH7
PHO81
ECM23
YMR265C
RSN1
SDH1
YPR195C
YER039C−A
ARR1
FIG1
SCO2
YEL043W
ATF2
RAD28
SGE1
YHB1
YGR237C
IRC4
YCR099C
YFR054C
YCR051W
YJL007C
YPL035C
NCE102
SPO71
NAS6
SNO2
ISR1
YME2
YCR100C
IRC22
YML018C
CTL1
ULA1
YPR117W
SLI15
AMN1
IFA38
NGL2
YPR098C
DAP2
YIL014C−A
HFM1
STL1
PHO4
HXK1
HRD3
YOL013W−B
YMR31
CIT3
YBL039W−B
YER158C
BUD8
YDR010C
YGR266W
YEL028W
YNL035C
DCI1
SNC2
BIK1
MVP1
CMC4
GTT1
TRE1
YJL022W
MAD2
MLH2
SSB1
HSM3
YDR239C
YAT1
EMP47
ATC1
alanine
aspartate
glutamate
phenylalanine
glycine
histidine
isoleucine
lysine
leucine
methionine
asparagine
proline
glutamine
arginine
serine
threonine
valine
tryptophan
tyrosine

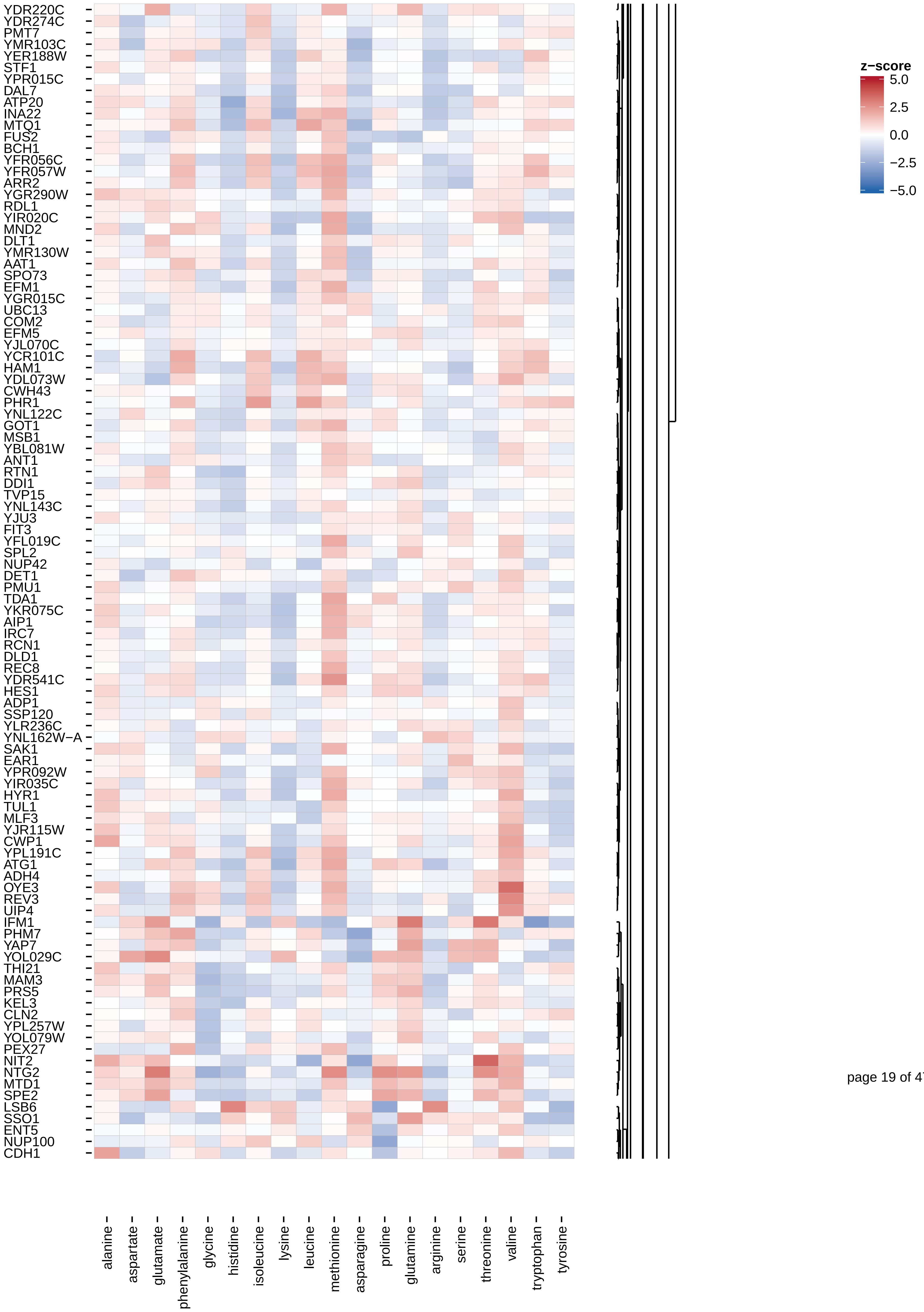
YDR220C
YDR274C
PMT7
YMR103C
YER188W
STF1
YPR015C
DAL7
ATP20
INA22
MTQ1
FUS2
BCH1
YFR056C
YFR057W
ARR2
YGR290W
RDL1
YIR020C
MND2
DLT1
YMR130W
AAT1
SPO73
EFM1
YGR015C
UBC13
COM2
EFM5
YJL070C
YCR101C
HAM1
YDL073W
CWH43
PHR1
YNL122C
GOT1
MSB1
YBL081W
ANT1
RTN1
DDI1
TVP15
YNL143C
YJU3
FIT3
YFL019C
SPL2
NUP42
DET1
PMU1
TDA1
YKR075C
AIP1
IRC7
RCN1
DLD1
REC8
YDR541C
HES1
ADP1
SSP120
YLR236C
YNL162W−A
SAK1
EAR1
YPR092W
YIR035C
HYR1
TUL1
MLF3
YJR115W
CWP1
YPL191C
ATG1
ADH4
OYE3
REV3
UIP4
IFM1
PHM7
YAP7
YOL029C
THI21
MAM3
PRS5
KEL3
CLN2
YPL257W
YOL079W
PEX27
NIT2
NTG2
MTD1
SPE2
LSB6
SSO1
ENT5
NUP100
CDH1
alanine
aspartate
glutamate
phenylalanine
glycine
histidine
isoleucine
lysine
leucine
methionine
asparagine
proline
glutamine
arginine
serine
threonine
valine
tryptophan
tyrosine
z−score
5.0
2.5
0.0
−2.5
−5.0

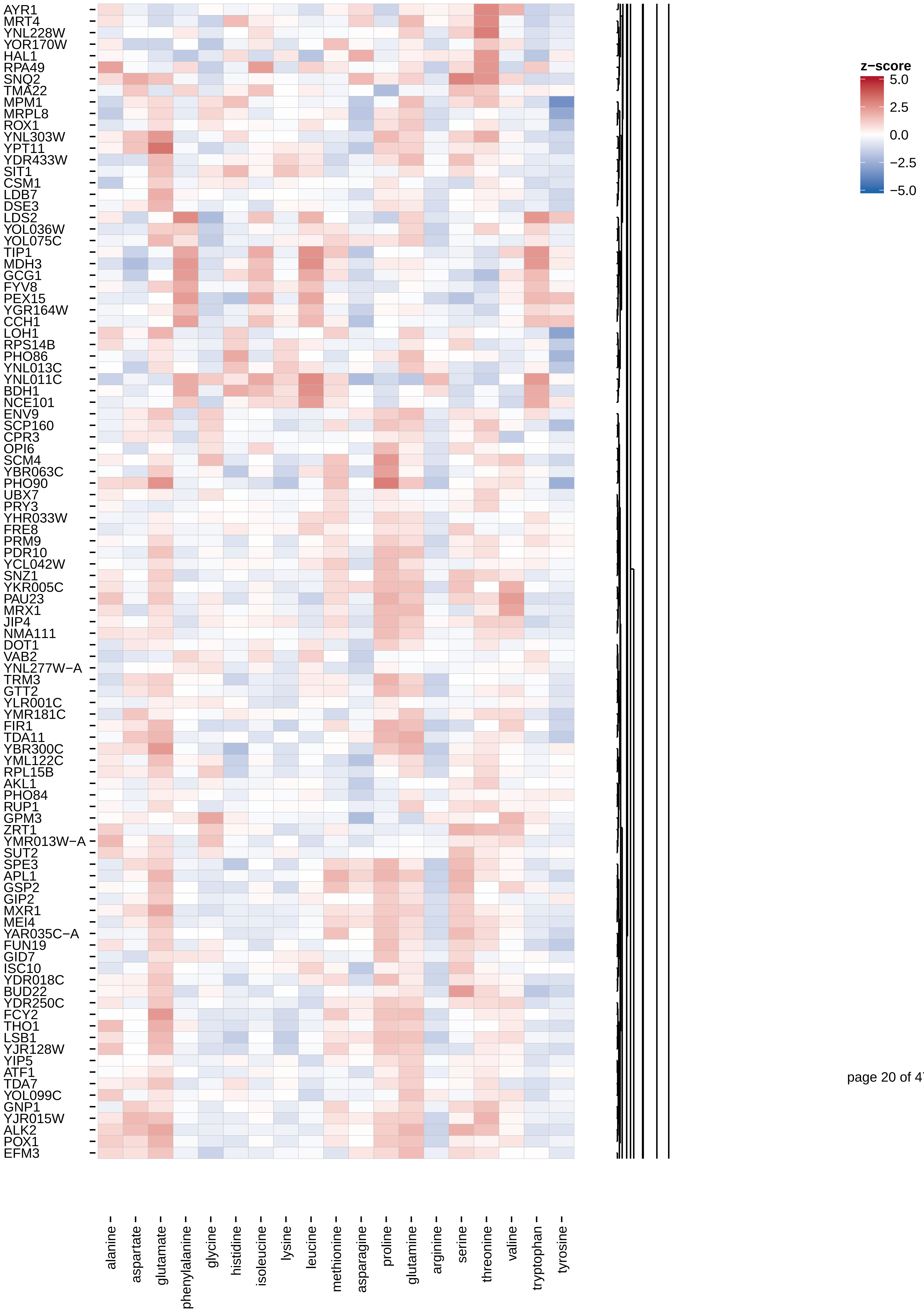
AYR1
MRT4
YNL228W
YOR170W
HAL1
RPA49
SNQ2
TMA22
MPM1
MRPL8
ROX1
YNL303W
YPT11
YDR433W
SIT1
CSM1
LDB7
DSE3
LDS2
YOL036W
YOL075C
TIP1
MDH3
GCG1
FYV8
PEX15
YGR164W
CCH1
LOH1
RPS14B
PHO86
YNL013C
YNL011C
BDH1
NCE101
ENV9
SCP160
CPR3
OPI6
SCM4
YBR063C
PHO90
UBX7
PRY3
YHR033W
FRE8
PRM9
PDR10
YCL042W
SNZ1
YKR005C
PAU23
MRX1
JIP4
NMA111
DOT1
VAB2
YNL277W−A
TRM3
GTT2
YLR001C
YMR181C
FIR1
TDA11
YBR300C
YML122C
RPL15B
AKL1
PHO84
RUP1
GPM3
ZRT1
YMR013W−A
SUT2
SPE3
APL1
GSP2
GIP2
MXR1
MEI4
YAR035C−A
FUN19
GID7
ISC10
YDR018C
BUD22
YDR250C
FCY2
THO1
LSB1
YJR128W
YIP5
ATF1
TDA7
YOL099C
GNP1
YJR015W
ALK2
POX1
EFM3
alanine
aspartate
glutamate
phenylalanine
glycine
histidine
isoleucine
lysine
leucine
methionine
asparagine
proline
glutamine
arginine
serine
threonine
valine
tryptophan
tyrosine
z−score
5.0
2.5
0.0
−2.5
−5.0

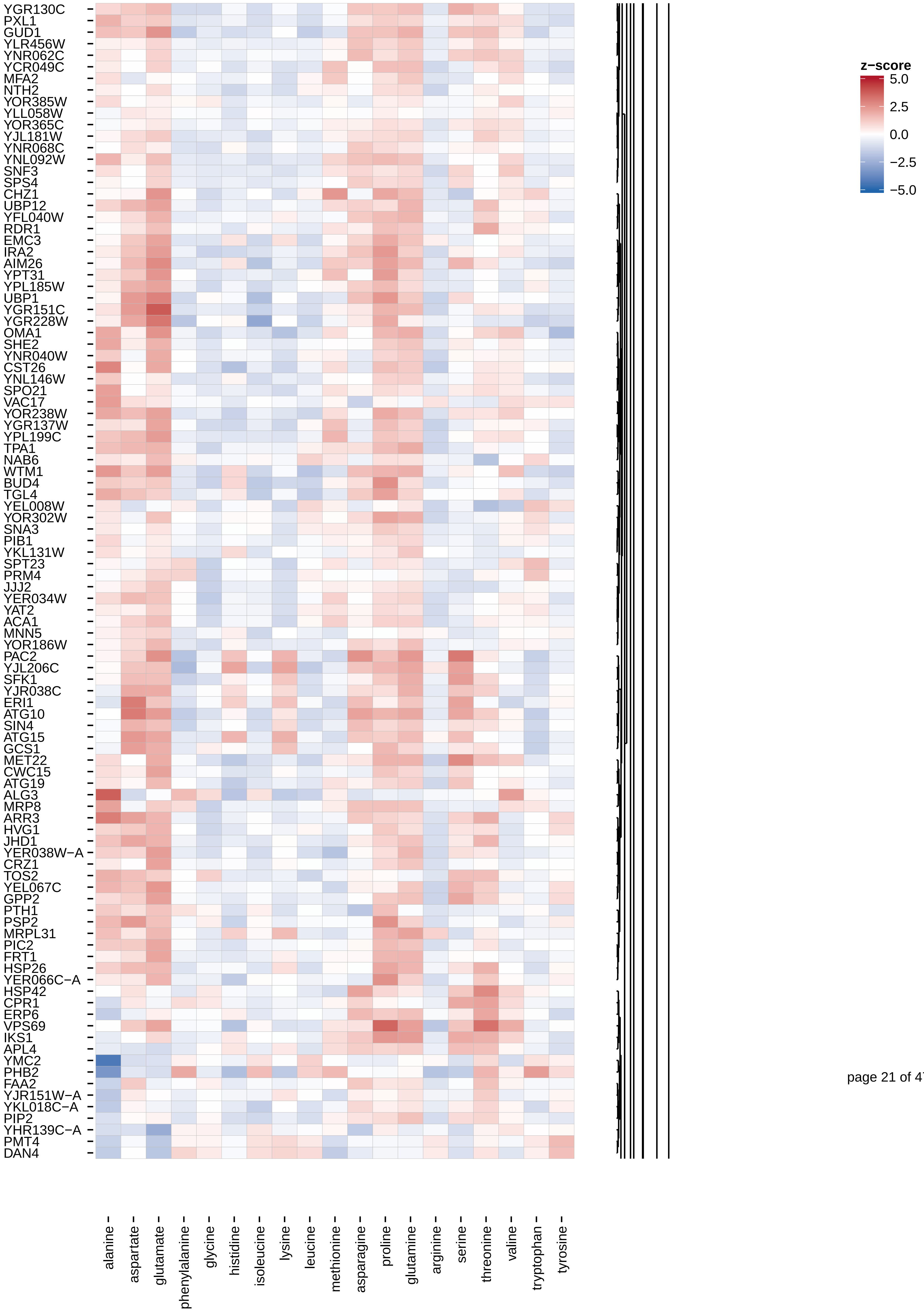
z−score
5.0
2.5
0.0
−2.5
−5.0
YGR130C
PXL1
GUD1
YLR456W
YNR062C
YCR049C
MFA2
NTH2
YOR385W
YLL058W
YOR365C
YJL181W
YNR068C
YNL092W
SNF3
SPS4
CHZ1
UBP12
YFL040W
RDR1
EMC3
IRA2
AIM26
YPT31
YPL185W
UBP1
YGR151C
YGR228W
OMA1
SHE2
YNR040W
CST26
YNL146W
SPO21
VAC17
YOR238W
YGR137W
YPL199C
TPA1
NAB6
WTM1
BUD4
TGL4
YEL008W
YOR302W
SNA3
PIB1
YKL131W
SPT23
PRM4
JJJ2
YER034W
YAT2
ACA1
MNN5
YOR186W
PAC2
YJL206C
SFK1
YJR038C
ERI1
ATG10
SIN4
ATG15
GCS1
MET22
CWC15
ATG19
ALG3
MRP8
ARR3
HVG1
JHD1
YER038W−A
CRZ1
TOS2
YEL067C
GPP2
PTH1
PSP2
MRPL31
PIC2
FRT1
HSP26
YER066C−A
HSP42
CPR1
ERP6
VPS69
IKS1
APL4
YMC2
PHB2
FAA2
YJR151W−A
YKL018C−A
PIP2
YHR139C−A
PMT4
DAN4
alanine
aspartate
glutamate
phenylalanine
glycine
histidine
isoleucine
lysine
leucine
methionine
asparagine
proline
glutamine
arginine
serine
threonine
valine
tryptophan
tyrosine

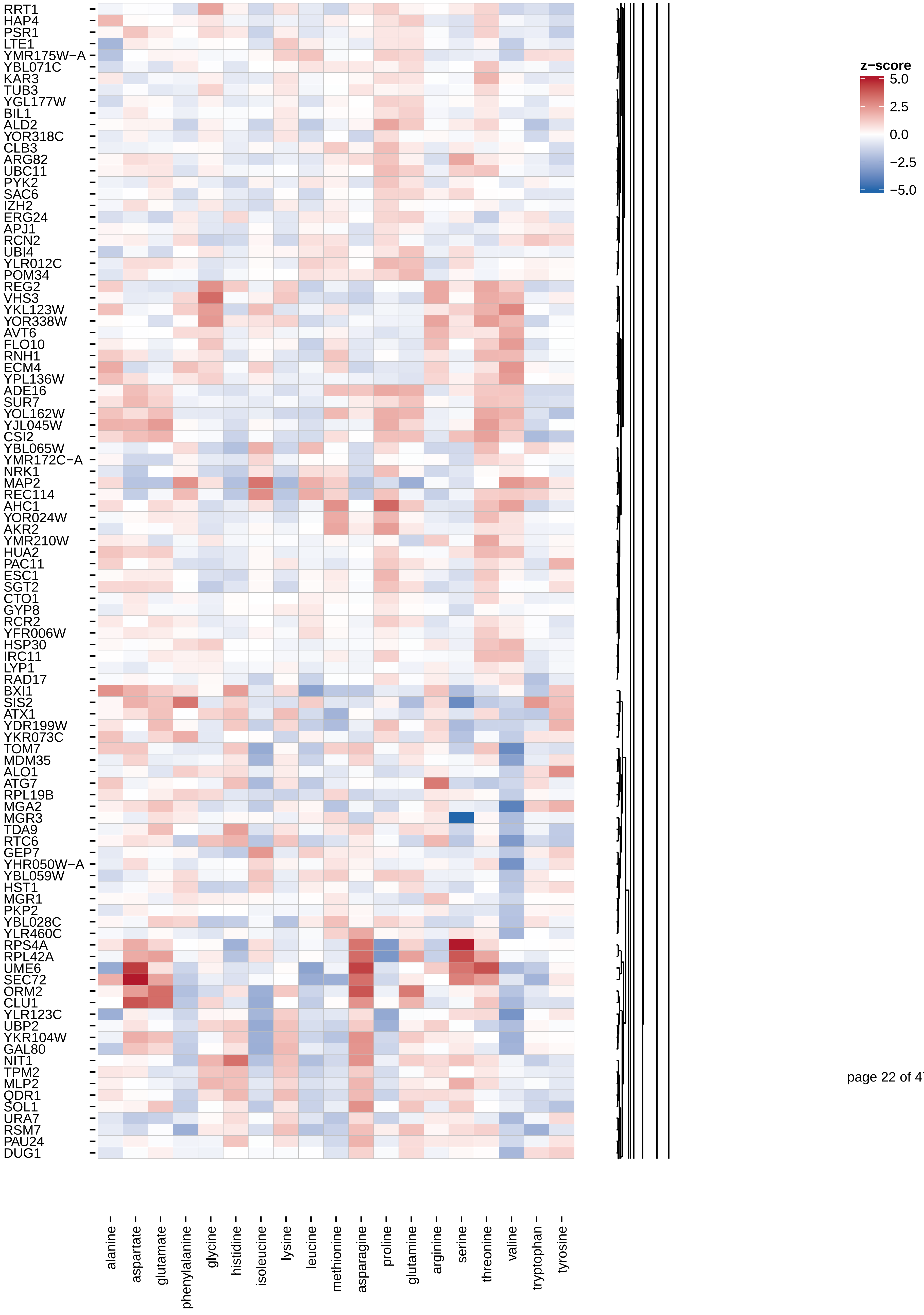

RRT1
HAP4
PSR1
LTE1
YMR175W−A
YBL071C
KAR3
TUB3
YGL177W
BIL1
ALD2
YOR318C
CLB3
ARG82
UBC11
PYK2
SAC6
IZH2
ERG24
APJ1
RCN2
UBI4
YLR012C
POM34
REG2
VHS3
YKL123W
YOR338W
AVT6
FLO10
RNH1
ECM4
YPL136W
ADE16
SUR7
YOL162W
YJL045W
CSI2
YBL065W
YMR172C−A
NRK1
MAP2
REC114
AHC1
YOR024W
AKR2
YMR210W
HUA2
PAC11
ESC1
SGT2
CTO1
GYP8
RCR2
YFR006W
HSP30
IRC11
LYP1
RAD17
BXI1
SIS2
ATX1
YDR199W
YKR073C
TOM7
MDM35
ALO1
ATG7
RPL19B
MGA2
MGR3
TDA9
RTC6
GEP7
YHR050W−A
YBL059W
HST1
MGR1
PKP2
YBL028C
YLR460C
RPS4A
RPL42A
UME6
SEC72
ORM2
CLU1
YLR123C
UBP2
YKR104W
GAL80
NIT1
TPM2
MLP2
QDR1
SOL1
URA7
RSM7
PAU24
DUG1
alanine
aspartate
glutamate
phenylalanine
glycine
histidine
isoleucine
lysine
leucine
methionine
asparagine
proline
glutamine
arginine
serine
threonine
valine
tryptophan
tyrosine
z−score
5.0
2.5
0.0
−2.5
−5.0

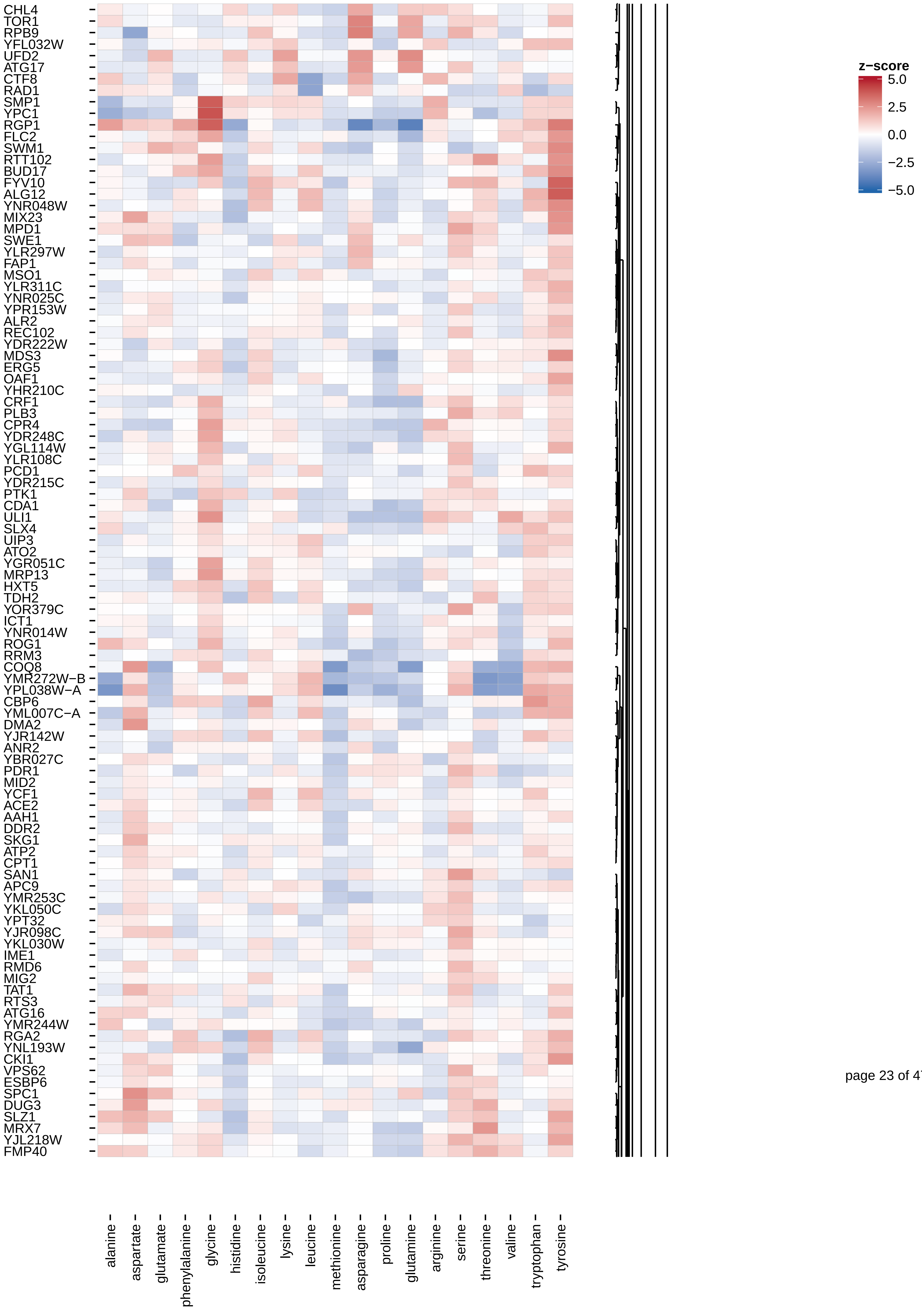

CHL4
TOR1
RPB9
YFL032W
UFD2
ATG17
CTF8
RAD1
SMP1
YPC1
RGP1
FLC2
SWM1
RTT102
BUD17
FYV10
ALG12
YNR048W
MIX23
MPD1
SWE1
YLR297W
FAP1
MSO1
YLR311C
YNR025C
YPR153W
ALR2
REC102
YDR222W
MDS3
ERG5
OAF1
YHR210C
CRF1
PLB3
CPR4
YDR248C
YGL114W
YLR108C
PCD1
YDR215C
PTK1
CDA1
ULI1
SLX4
UIP3
ATO2
YGR051C
MRP13
HXT5
TDH2
YOR379C
ICT1
YNR014W
ROG1
RRM3
COQ8
YMR272W−B
YPL038W−A
CBP6
YML007C−A
DMA2
YJR142W
ANR2
YBR027C
PDR1
MID2
YCF1
ACE2
AAH1
DDR2
SKG1
ATP2
CPT1
SAN1
APC9
YMR253C
YKL050C
YPT32
YJR098C
YKL030W
IME1
RMD6
MIG2
TAT1
RTS3
ATG16
YMR244W
RGA2
YNL193W
CKI1
VPS62
ESBP6
SPC1
DUG3
SLZ1
MRX7
YJL218W
FMP40
alanine
aspartate
glutamate
phenylalanine
glycine
histidine
isoleucine
lysine
leucine
methionine
asparagine
proline
glutamine
arginine
serine
threonine
valine
tryptophan
tyrosine
z−score
5.0
2.5
0.0
−2.5
−5.0

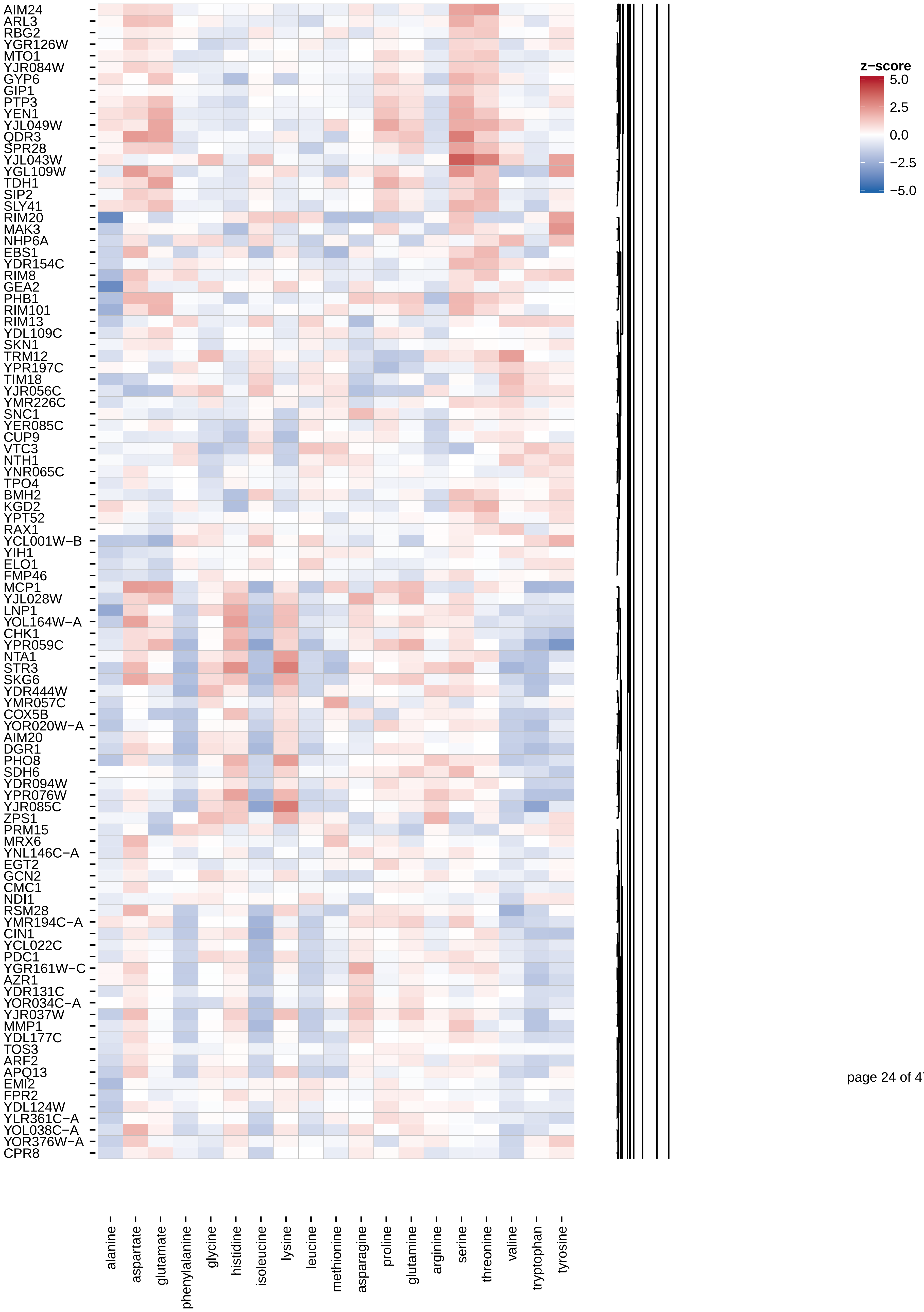

AIM24
ARL3
RBG2
YGR126W
MTO1
YJR084W
GYP6
GIP1
PTP3
YEN1
YJL049W
QDR3
SPR28
YJL043W
YGL109W
TDH1
SIP2
SLY41
RIM20
MAK3
NHP6A
EBS1
YDR154C
RIM8
GEA2
PHB1
RIM101
RIM13
YDL109C
SKN1
TRM12
YPR197C
TIM18
YJR056C
YMR226C
SNC1
YER085C
CUP9
VTC3
NTH1
YNR065C
TPO4
BMH2
KGD2
YPT52
RAX1
YCL001W−B
YIH1
ELO1
FMP46
MCP1
YJL028W
LNP1
YOL164W−A
CHK1
YPR059C
NTA1
STR3
SKG6
YDR444W
YMR057C
COX5B
YOR020W−A
AIM20
DGR1
PHO8
SDH6
YDR094W
YPR076W
YJR085C
ZPS1
PRM15
MRX6
YNL146C−A
EGT2
GCN2
CMC1
NDI1
RSM28
YMR194C−A
CIN1
YCL022C
PDC1
YGR161W−C
AZR1
YDR131C
YOR034C−A
YJR037W
MMP1
YDL177C
TOS3
ARF2
APQ13
EMI2
FPR2
YDL124W
YLR361C−A
YOL038C−A
YOR376W−A
CPR8
alanine
aspartate
glutamate
phenylalanine
glycine
histidine
isoleucine
lysine
leucine
methionine
asparagine
proline
glutamine
arginine
serine
threonine
valine
tryptophan
tyrosine
z−score
5.0
2.5
0.0
−2.5
−5.0

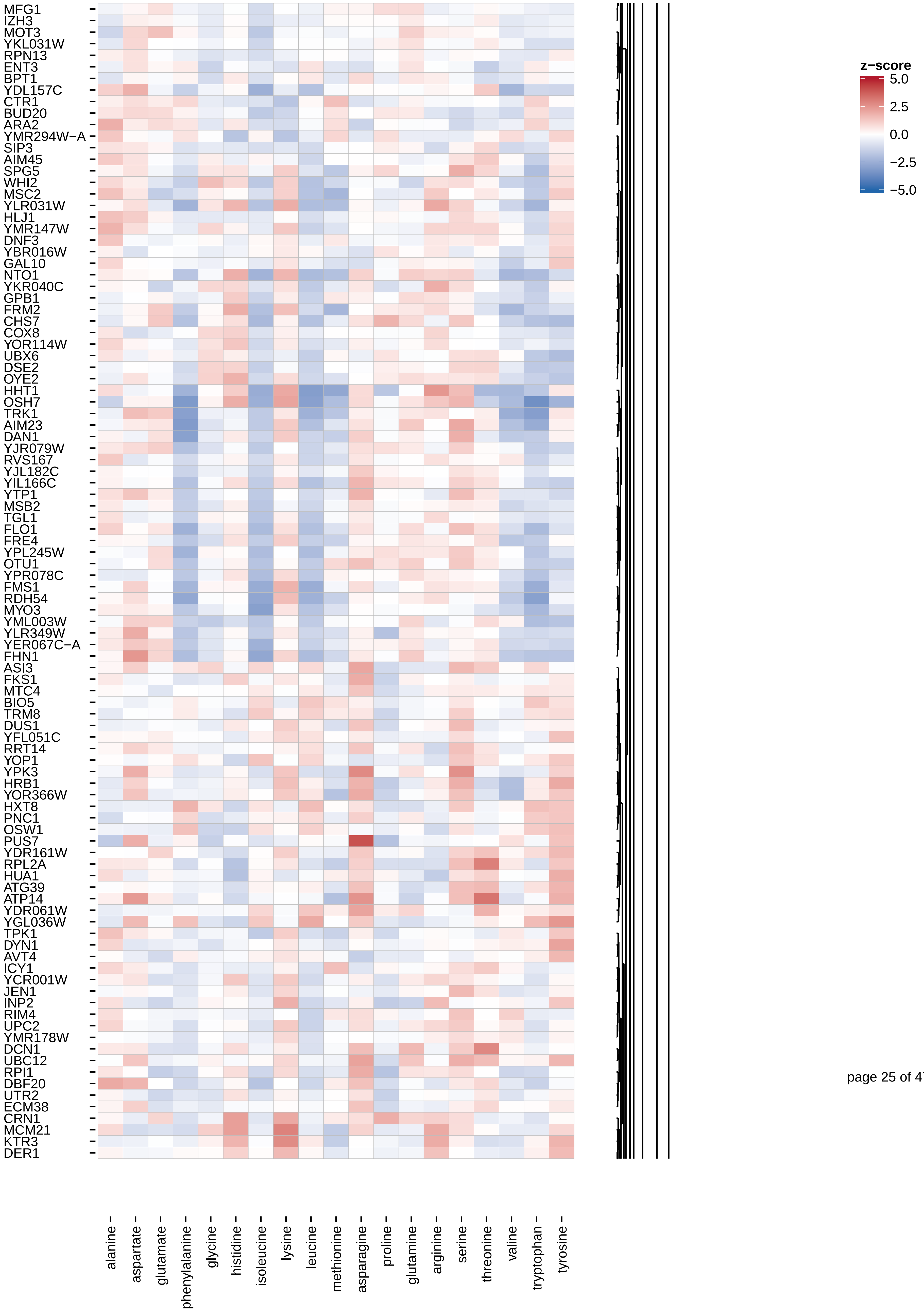
MFG1
IZH3
MOT3
YKL031W
RPN13
ENT3
BPT1
YDL157C
CTR1
BUD20
ARA2
YMR294W−A
SIP3
AIM45
SPG5
WHI2
MSC2
YLR031W
HLJ1
YMR147W
DNF3
YBR016W
GAL10
NTO1
YKR040C
GPB1
FRM2
CHS7
COX8
YOR114W
UBX6
DSE2
OYE2
HHT1
OSH7
TRK1
AIM23
DAN1
YJR079W
RVS167
YJL182C
YIL166C
YTP1
MSB2
TGL1
FLO1
FRE4
YPL245W
OTU1
YPR078C
FMS1
RDH54
MYO3
YML003W
YLR349W
YER067C−A
FHN1
ASI3
FKS1
MTC4
BIO5
TRM8
DUS1
YFL051C
RRT14
YOP1
YPK3
HRB1
YOR366W
HXT8
PNC1
OSW1
PUS7
YDR161W
RPL2A
HUA1
ATG39
ATP14
YDR061W
YGL036W
TPK1
DYN1
AVT4
ICY1
YCR001W
JEN1
INP2
RIM4
UPC2
YMR178W
DCN1
UBC12
RPI1
DBF20
UTR2
ECM38
CRN1
MCM21
KTR3
DER1
alanine
aspartate
glutamate
phenylalanine
glycine
histidine
isoleucine
lysine
leucine
methionine
asparagine
proline
glutamine
arginine
serine
threonine
valine
tryptophan
tyrosine
z−score
5.0
2.5
0.0
−2.5
−5.0

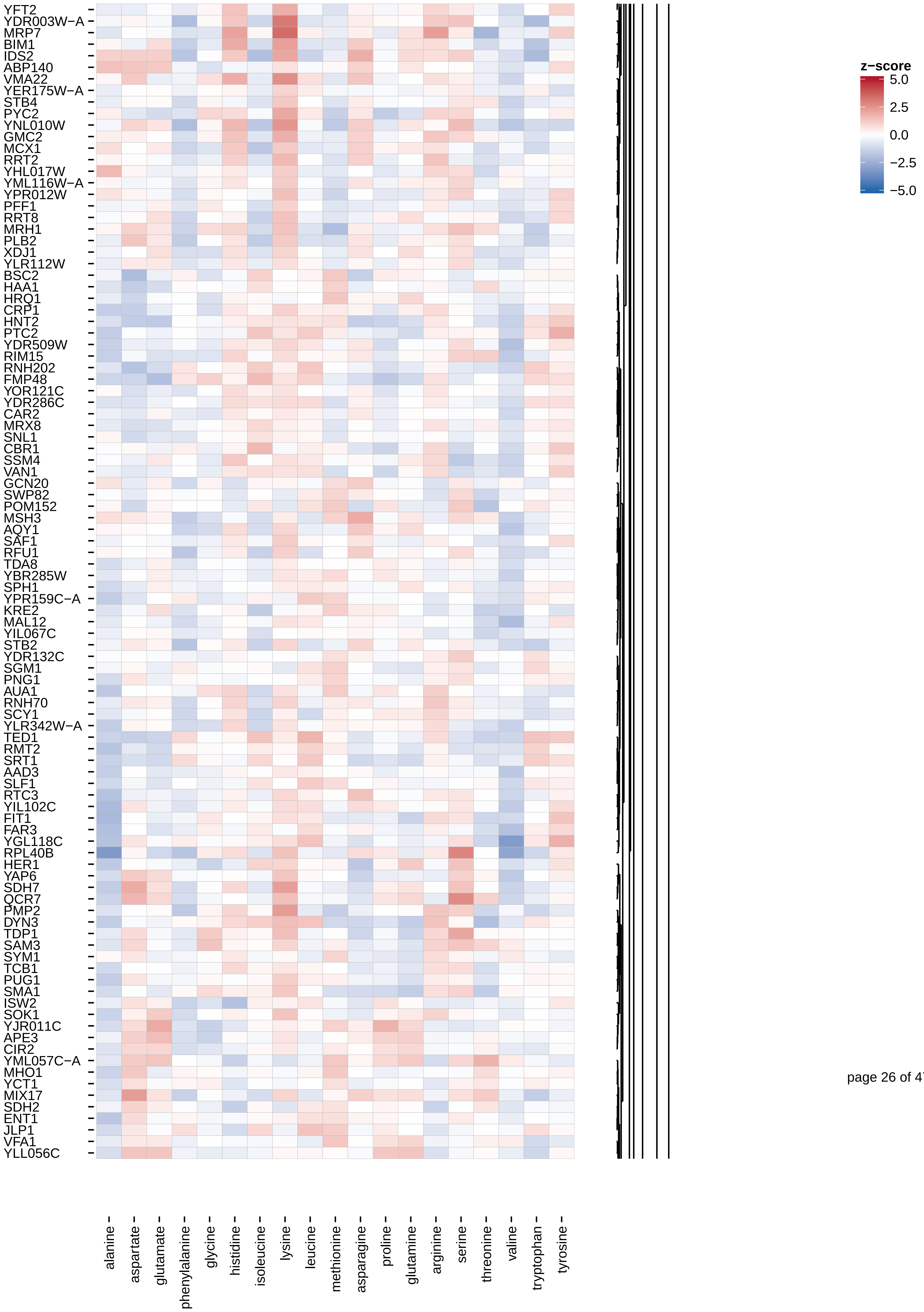

YFT2
YDR003W−A
MRP7
BIM1
IDS2
ABP140
VMA22
YER175W−A
STB4
PYC2
YNL010W
GMC2
MCX1
RRT2
YHL017W
YML116W−A
YPR012W
PFF1
RRT8
MRH1
PLB2
XDJ1
YLR112W
BSC2
HAA1
HRQ1
CRP1
HNT2
PTC2
YDR509W
RIM15
RNH202
FMP48
YOR121C
YDR286C
CAR2
MRX8
SNL1
CBR1
SSM4
VAN1
GCN20
SWP82
POM152
MSH3
AQY1
SAF1
RFU1
TDA8
YBR285W
SPH1
YPR159C−A
KRE2
MAL12
YIL067C
STB2
YDR132C
SGM1
PNG1
AUA1
RNH70
SCY1
YLR342W−A
TED1
RMT2
SRT1
AAD3
SLF1
RTC3
YIL102C
FIT1
FAR3
YGL118C
RPL40B
HER1
YAP6
SDH7
QCR7
PMP2
DYN3
TDP1
SAM3
SYM1
TCB1
PUG1
SMA1
ISW2
SOK1
YJR011C
APE3
CIR2
YML057C−A
MHO1
YCT1
MIX17
SDH2
ENT1
JLP1
VFA1
YLL056C
alanine
aspartate
glutamate
phenylalanine
glycine
histidine
isoleucine
lysine
leucine
methionine
asparagine
proline
glutamine
arginine
serine
threonine
valine
tryptophan
tyrosine
z−score
5.0
2.5
0.0
−2.5
−5.0

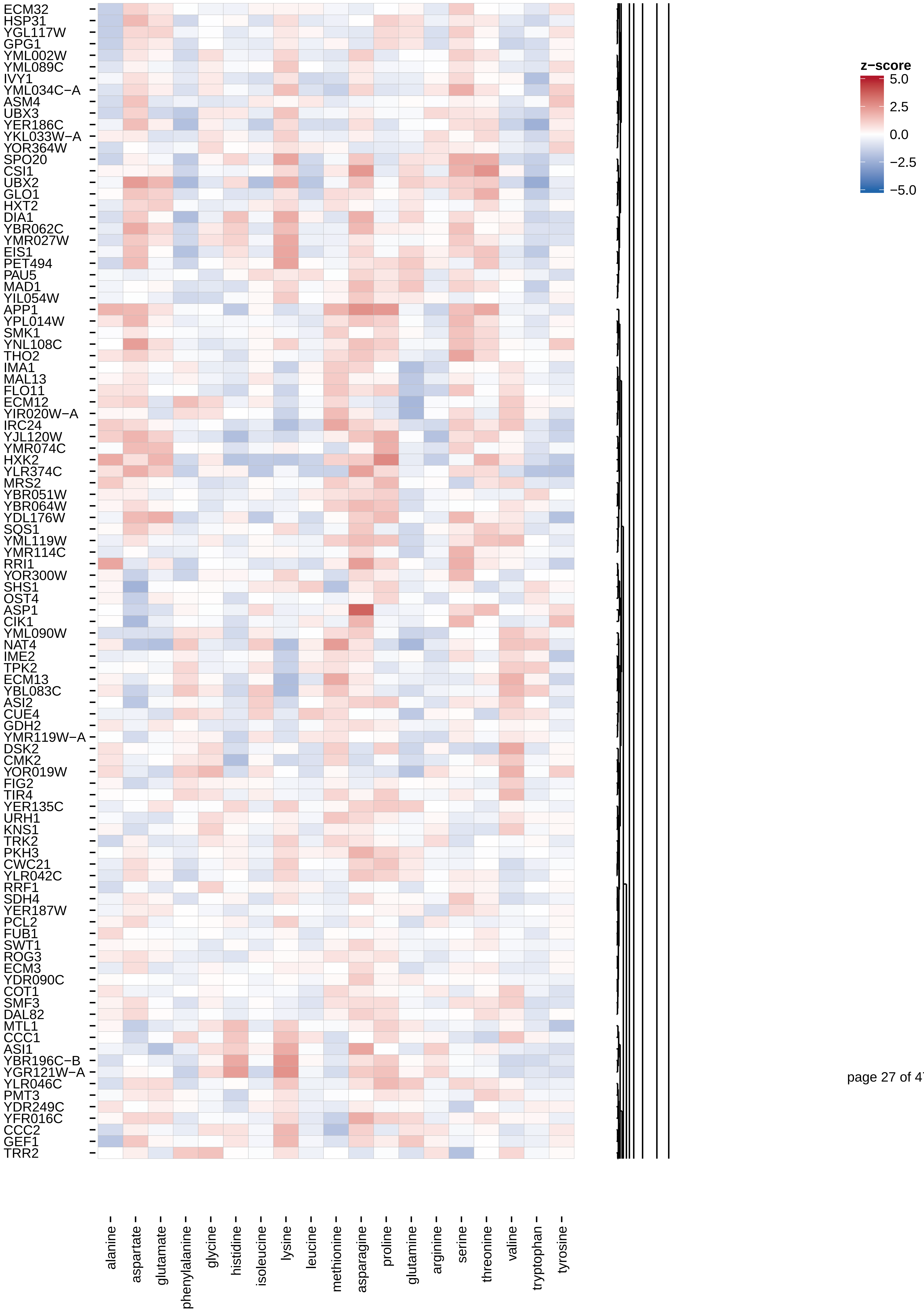
ECM32
HSP31
YGL117W
GPG1
YML002W
YML089C
IVY1
YML034C−A
ASM4
UBX3
YER186C
YKL033W−A
YOR364W
SPO20
CSI1
UBX2
GLO1
HXT2
DIA1
YBR062C
YMR027W
EIS1
PET494
PAU5
MAD1
YIL054W
APP1
YPL014W
SMK1
YNL108C
THO2
IMA1
MAL13
FLO11
ECM12
YIR020W−A
IRC24
YJL120W
YMR074C
HXK2
YLR374C
MRS2
YBR051W
YBR064W
YDL176W
SQS1
YML119W
YMR114C
RRI1
YOR300W
SHS1
OST4
ASP1
CIK1
YML090W
NAT4
IME2
TPK2
ECM13
YBL083C
ASI2
CUE4
GDH2
YMR119W−A
DSK2
CMK2
YOR019W
FIG2
TIR4
YER135C
URH1
KNS1
TRK2
PKH3
CWC21
YLR042C
RRF1
SDH4
YER187W
PCL2
FUB1
SWT1
ROG3
ECM3
YDR090C
COT1
SMF3
DAL82
MTL1
CCC1
ASI1
YBR196C−B
YGR121W−A
YLR046C
PMT3
YDR249C
YFR016C
CCC2
GEF1
TRR2
alanine
aspartate
glutamate
phenylalanine
glycine
histidine
isoleucine
lysine
leucine
methionine
asparagine
proline
glutamine
arginine
serine
threonine
valine
tryptophan
tyrosine
z−score
5.0
2.5
0.0
−2.5
−5.0

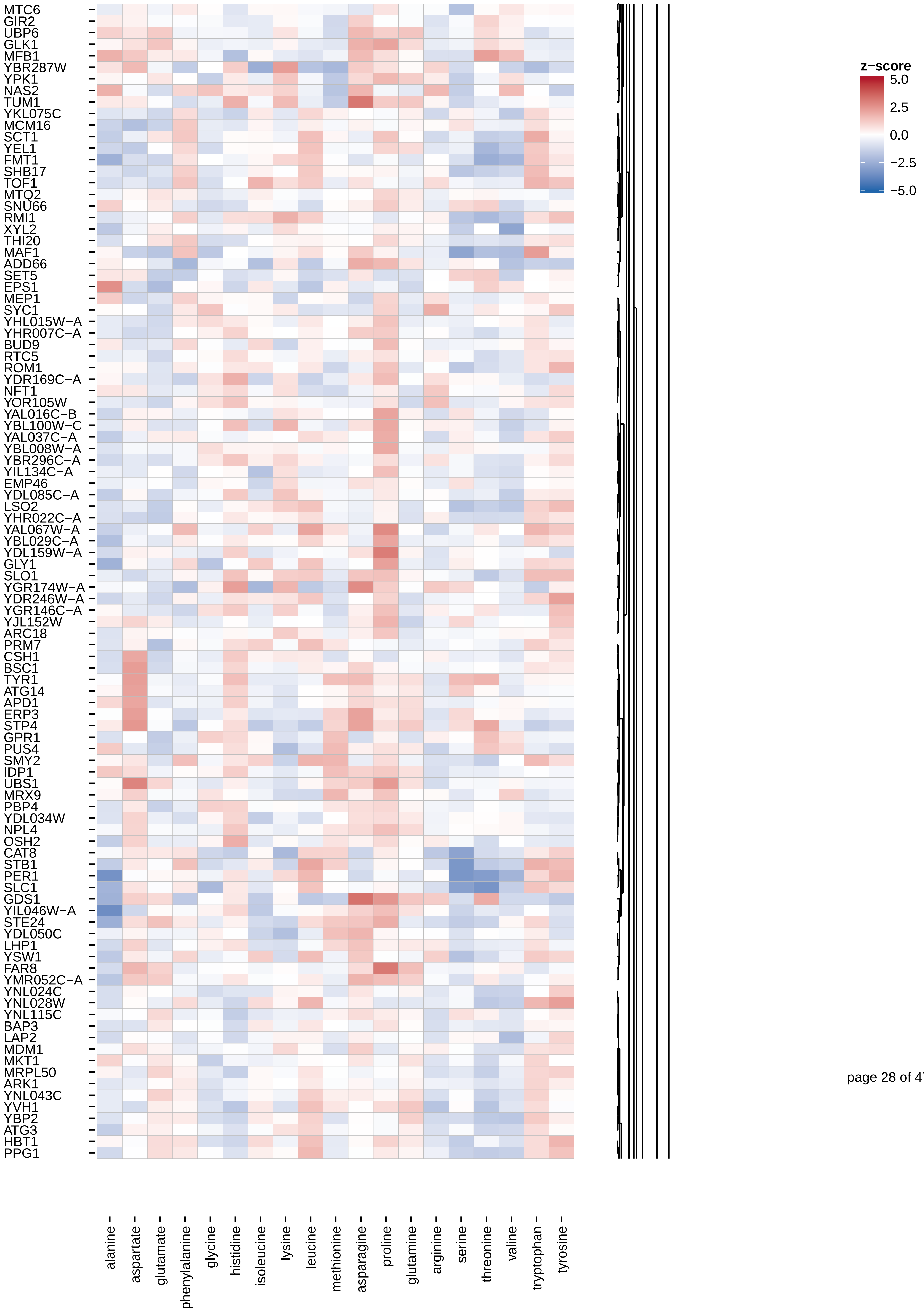
MTC6
GIR2
UBP6
GLK1
MFB1
YBR287W
YPK1
NAS2
TUM1
YKL075C
MCM16
SCT1
YEL1
FMT1
SHB17
TOF1
MTQ2
SNU66
RMI1
XYL2
THI20
MAF1
ADD66
SET5
EPS1
MEP1
SYC1
YHL015W−A
YHR007C−A
BUD9
RTC5
ROM1
YDR169C−A
NFT1
YOR105W
YAL016C−B
YBL100W−C
YAL037C−A
YBL008W−A
YBR296C−A
YIL134C−A
EMP46
YDL085C−A
LSO2
YHR022C−A
YAL067W−A
YBL029C−A
YDL159W−A
GLY1
SLO1
YGR174W−A
YDR246W−A
YGR146C−A
YJL152W
ARC18
PRM7
CSH1
BSC1
TYR1
ATG14
APD1
ERP3
STP4
GPR1
PUS4
SMY2
IDP1
UBS1
MRX9
PBP4
YDL034W
NPL4
OSH2
CAT8
STB1
PER1
SLC1
GDS1
YIL046W−A
STE24
YDL050C
LHP1
YSW1
FAR8
YMR052C−A
YNL024C
YNL028W
YNL115C
BAP3
LAP2
MDM1
MKT1
MRPL50
ARK1
YNL043C
YVH1
YBP2
ATG3
HBT1
PPG1
alanine
aspartate
glutamate
phenylalanine
glycine
histidine
isoleucine
lysine
leucine
methionine
asparagine
proline
glutamine
arginine
serine
threonine
valine
tryptophan
tyrosine
z−score
5.0
2.5
0.0
−2.5
−5.0

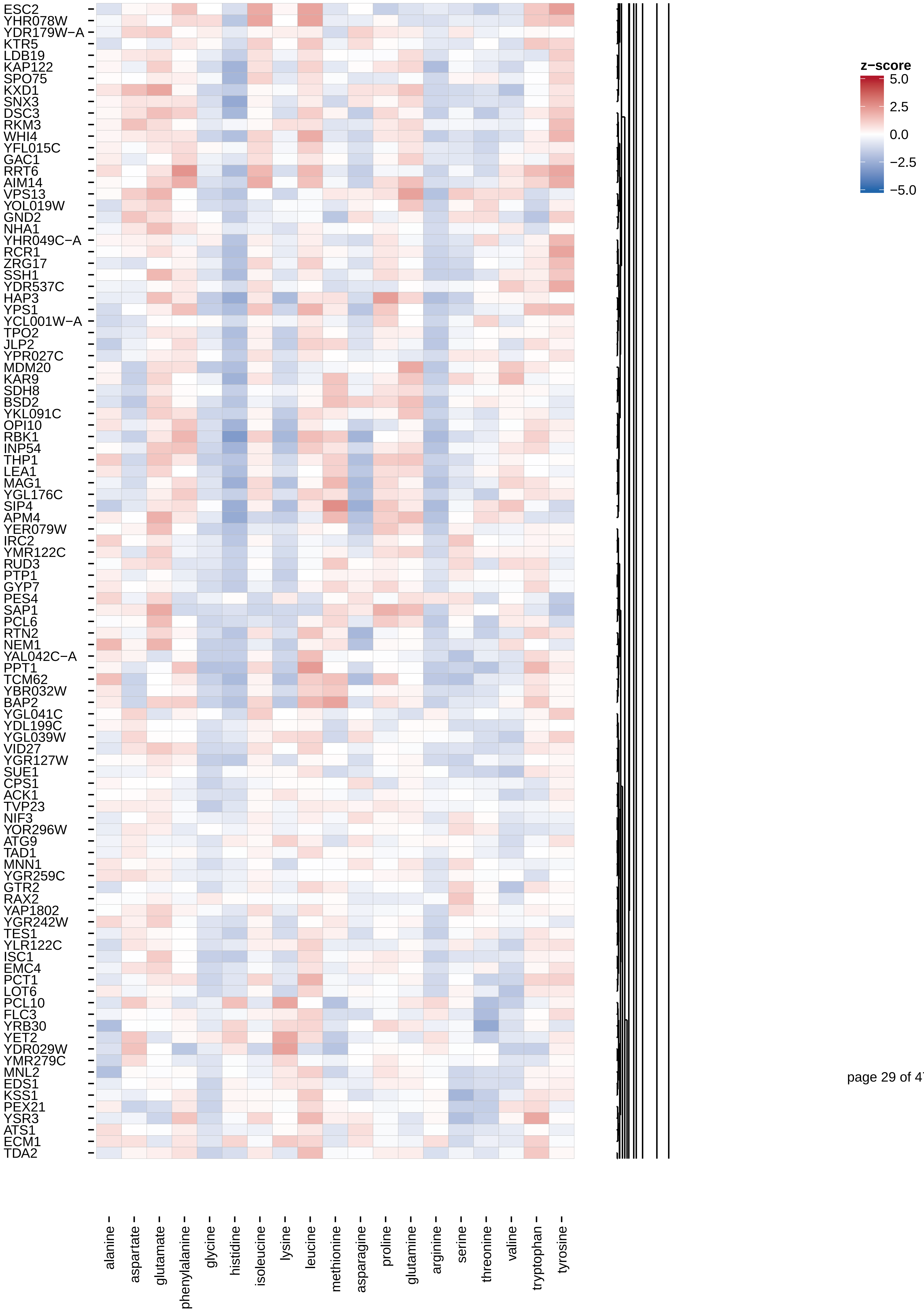
ESC2
YHR078W
YDR179W−A
KTR5
LDB19
KAP122
SPO75
KXD1
SNX3
DSC3
RKM3
WHI4
YFL015C
GAC1
RRT6
AIM14
VPS13
YOL019W
GND2
NHA1
YHR049C−A
RCR1
ZRG17
SSH1
YDR537C
HAP3
YPS1
YCL001W−A
TPO2
JLP2
YPR027C
MDM20
KAR9
SDH8
BSD2
YKL091C
OPI10
RBK1
INP54
THP1
LEA1
MAG1
YGL176C
SIP4
APM4
YER079W
IRC2
YMR122C
RUD3
PTP1
GYP7
PES4
SAP1
PCL6
RTN2
NEM1
YAL042C−A
PPT1
TCM62
YBR032W
BAP2
YGL041C
YDL199C
YGL039W
VID27
YGR127W
SUE1
CPS1
ACK1
TVP23
NIF3
YOR296W
ATG9
TAD1
MNN1
YGR259C
GTR2
RAX2
YAP1802
YGR242W
TES1
YLR122C
ISC1
EMC4
PCT1
LOT6
PCL10
FLC3
YRB30
YET2
YDR029W
YMR279C
MNL2
EDS1
KSS1
PEX21
YSR3
ATS1
ECM1
TDA2
alanine
aspartate
glutamate
phenylalanine
glycine
histidine
isoleucine
lysine
leucine
methionine
asparagine
proline
glutamine
arginine
serine
threonine
valine
tryptophan
tyrosine
z−score
5.0
2.5
0.0
−2.5
−5.0

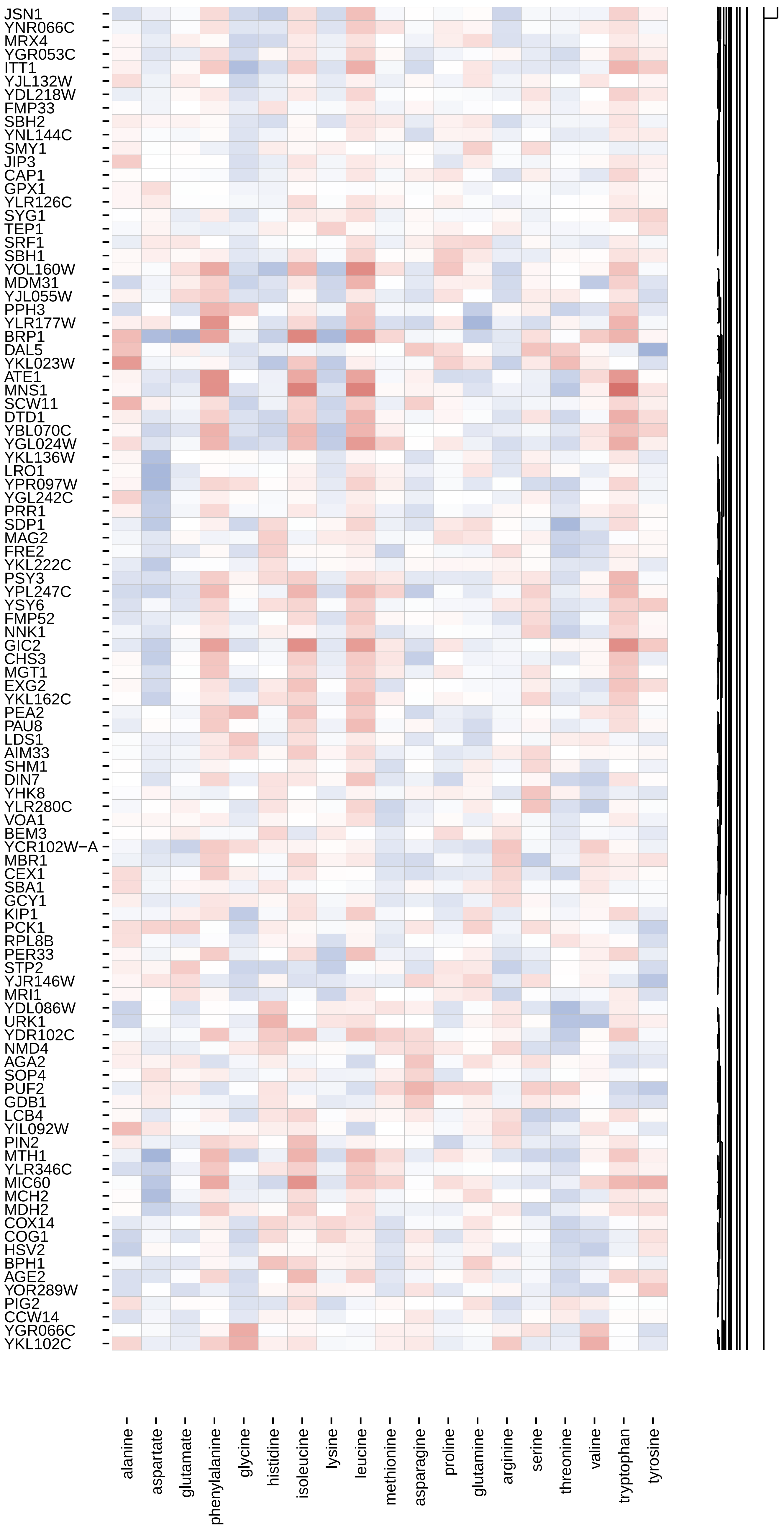
JSN1
YNR066C
MRX4
YGR053C
ITT1
YJL132W
YDL218W
FMP33
SBH2
YNL144C
SMY1
JIP3
CAP1
GPX1
YLR126C
SYG1
TEP1
SRF1
SBH1
YOL160W
MDM31
YJL055W
PPH3
YLR177W
BRP1
DAL5
YKL023W
ATE1
MNS1
SCW11
DTD1
YBL070C
YGL024W
YKL136W
LRO1
YPR097W
YGL242C
PRR1
SDP1
MAG2
FRE2
YKL222C
PSY3
YPL247C
YSY6
FMP52
NNK1
GIC2
CHS3
MGT1
EXG2
YKL162C
PEA2
PAU8
LDS1
AIM33
SHM1
DIN7
YHK8
YLR280C
VOA1
BEM3
YCR102W−A
MBR1
CEX1
SBA1
GCY1
KIP1
PCK1
RPL8B
PER33
STP2
YJR146W
MRI1
YDL086W
URK1
YDR102C
NMD4
AGA2
SOP4
PUF2
GDB1
LCB4
YIL092W
PIN2
MTH1
YLR346C
MIC60
MCH2
MDH2
COX14
COG1
HSV2
BPH1
AGE2
YOR289W
PIG2
CCW14
YGR066C
YKL102C
alanine
aspartate
glutamate
phenylalanine
glycine
histidine
isoleucine
lysine
leucine
methionine
asparagine
proline
glutamine
arginine
serine
threonine
valine
tryptophan
tyrosine

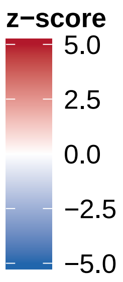
z−score
5.0
2.5
0.0
−2.5
−5.0

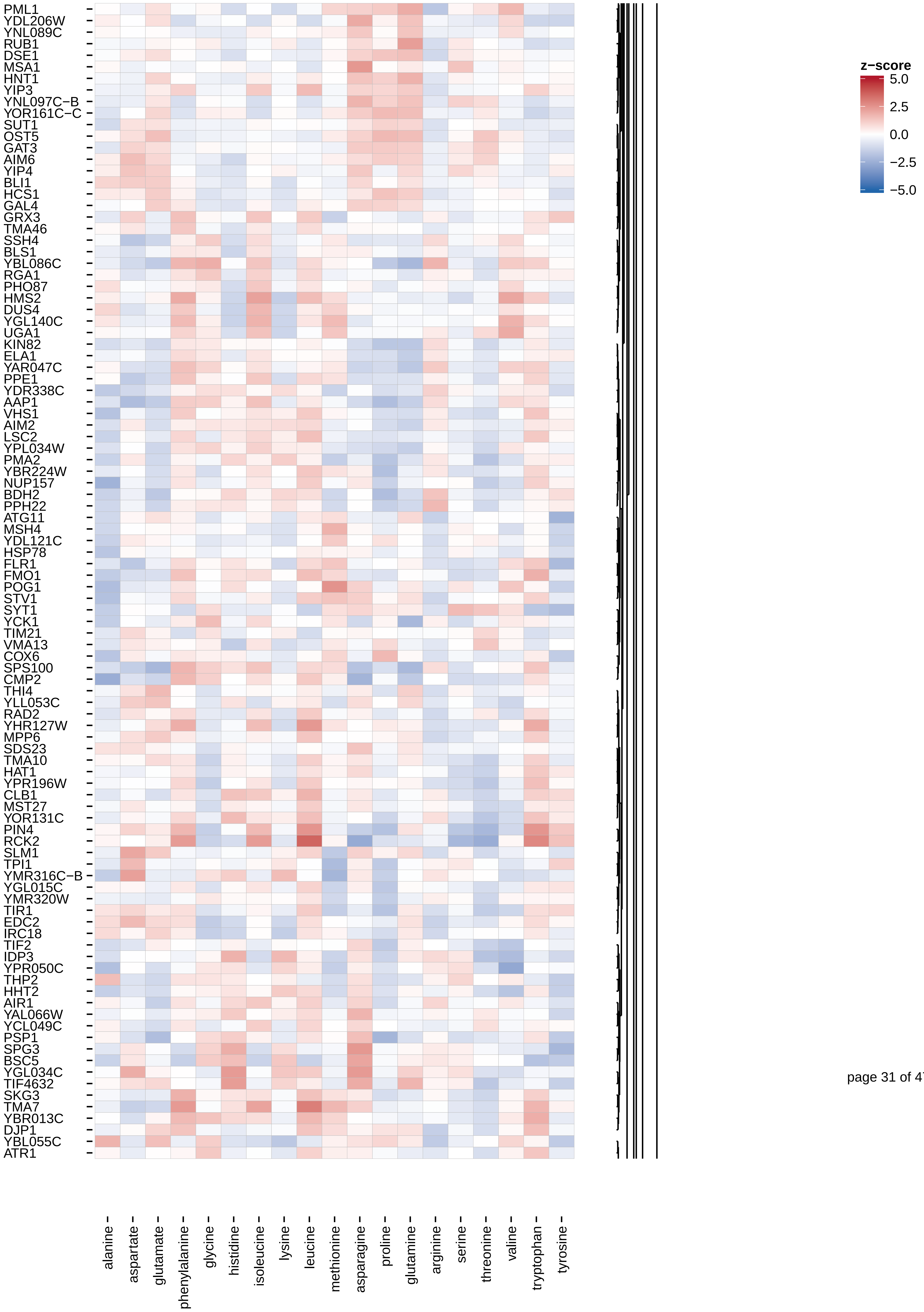
PML1
YDL206W
YNL089C
RUB1
DSE1
MSA1
HNT1
YIP3
YNL097C−B
YOR161C−C
SUT1
OST5
GAT3
AIM6
YIP4
BLI1
HCS1
GAL4
GRX3
TMA46
SSH4
BLS1
YBL086C
RGA1
PHO87
HMS2
DUS4
YGL140C
UGA1
KIN82
ELA1
YAR047C
PPE1
YDR338C
AAP1
VHS1
AIM2
LSC2
YPL034W
PMA2
YBR224W
NUP157
BDH2
PPH22
ATG11
MSH4
YDL121C
HSP78
FLR1
FMO1
POG1
STV1
SYT1
YCK1
TIM21
VMA13
COX6
SPS100
CMP2
THI4
YLL053C
RAD2
YHR127W
MPP6
SDS23
TMA10
HAT1
YPR196W
CLB1
MST27
YOR131C
PIN4
RCK2
SLM1
TPI1
YMR316C−B
YGL015C
YMR320W
TIR1
EDC2
IRC18
TIF2
IDP3
YPR050C
THP2
HHT2
AIR1
YAL066W
YCL049C
PSP1
SPG3
BSC5
YGL034C
TIF4632
SKG3
TMA7
YBR013C
DJP1
YBL055C
ATR1
alanine
aspartate
glutamate
phenylalanine
glycine
histidine
isoleucine
lysine
leucine
methionine
asparagine
proline
glutamine
arginine
serine
threonine
valine
tryptophan
tyrosine
z−score
5.0
2.5
0.0
−2.5
−5.0

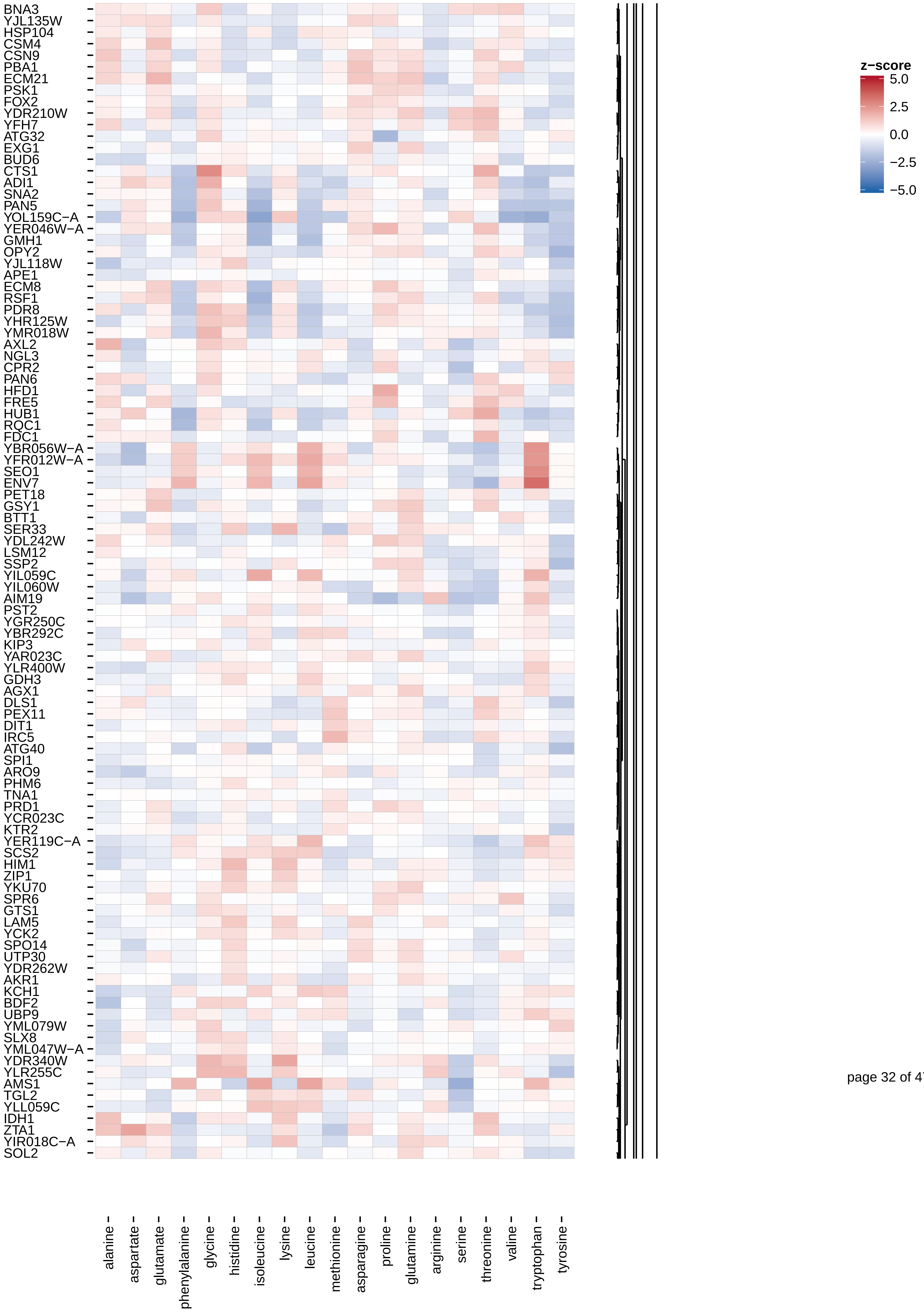
BNA3
YJL135W
HSP104
CSM4
CSN9
PBA1
ECM21
PSK1
FOX2
YDR210W
YFH7
ATG32
EXG1
BUD6
CTS1
ADI1
SNA2
PAN5
YOL159C−A
YER046W−A
GMH1
OPY2
YJL118W
APE1
ECM8
RSF1
PDR8
YHR125W
YMR018W
AXL2
NGL3
CPR2
PAN6
HFD1
FRE5
HUB1
RQC1
FDC1
YBR056W−A
YFR012W−A
SEO1
ENV7
PET18
GSY1
BTT1
SER33
YDL242W
LSM12
SSP2
YIL059C
YIL060W
AIM19
PST2
YGR250C
YBR292C
KIP3
YAR023C
YLR400W
GDH3
AGX1
DLS1
PEX11
DIT1
IRC5
ATG40
SPI1
ARO9
PHM6
TNA1
PRD1
YCR023C
KTR2
YER119C−A
SCS2
HIM1
ZIP1
YKU70
SPR6
GTS1
LAM5
YCK2
SPO14
UTP30
YDR262W
AKR1
KCH1
BDF2
UBP9
YML079W
SLX8
YML047W−A
YDR340W
YLR255C
AMS1
TGL2
YLL059C
IDH1
ZTA1
YIR018C−A
SOL2
alanine
aspartate
glutamate
phenylalanine
glycine
histidine
isoleucine
lysine
leucine
methionine
asparagine
proline
glutamine
arginine
serine
threonine
valine
tryptophan
tyrosine
z−score
5.0
2.5
0.0
−2.5
−5.0

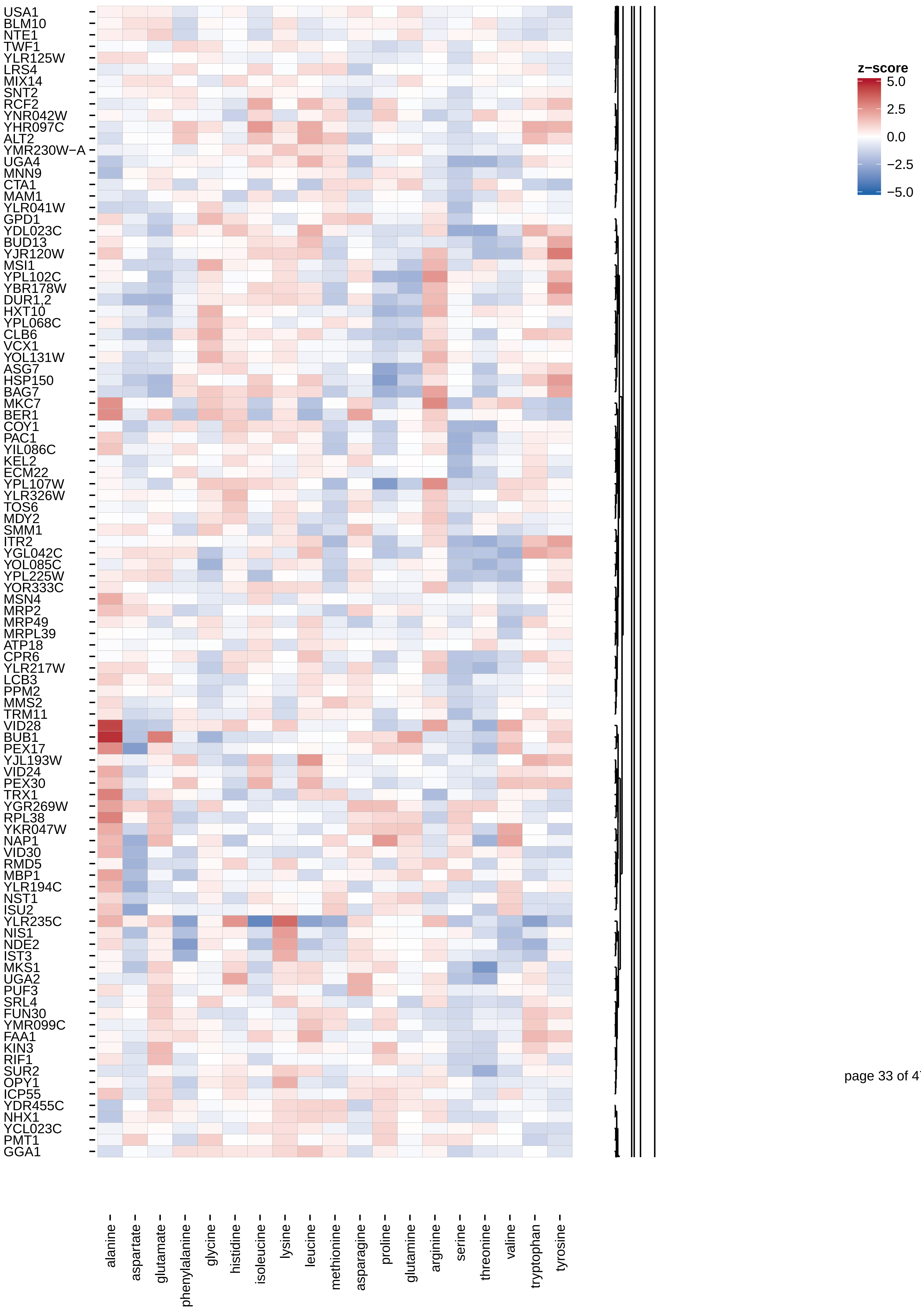
USA1
BLM10
NTE1
TWF1
YLR125W
LRS4
MIX14
SNT2
RCF2
YNR042W
YHR097C
ALT2
YMR230W−A
UGA4
MNN9
CTA1
MAM1
YLR041W
GPD1
YDL023C
BUD13
YJR120W
MSI1
YPL102C
YBR178W
DUR1,2
HXT10
YPL068C
CLB6
VCX1
YOL131W
ASG7
HSP150
BAG7
MKC7
BER1
COY1
PAC1
YIL086C
KEL2
ECM22
YPL107W
YLR326W
TOS6
MDY2
SMM1
ITR2
YGL042C
YOL085C
YPL225W
YOR333C
MSN4
MRP2
MRP49
MRPL39
ATP18
CPR6
YLR217W
LCB3
PPM2
MMS2
TRM11
VID28
BUB1
PEX17
YJL193W
VID24
PEX30
TRX1
YGR269W
RPL38
YKR047W
NAP1
VID30
RMD5
MBP1
YLR194C
NST1
ISU2
YLR235C
NIS1
NDE2
IST3
MKS1
UGA2
PUF3
SRL4
FUN30
YMR099C
FAA1
KIN3
RIF1
SUR2
OPY1
ICP55
YDR455C
NHX1
YCL023C
PMT1
GGA1
alanine
aspartate
glutamate
phenylalanine
glycine
histidine
isoleucine
lysine
leucine
methionine
asparagine
proline
glutamine
arginine
serine
threonine
valine
tryptophan
tyrosine
z−score
5.0
2.5
0.0
−2.5
−5.0

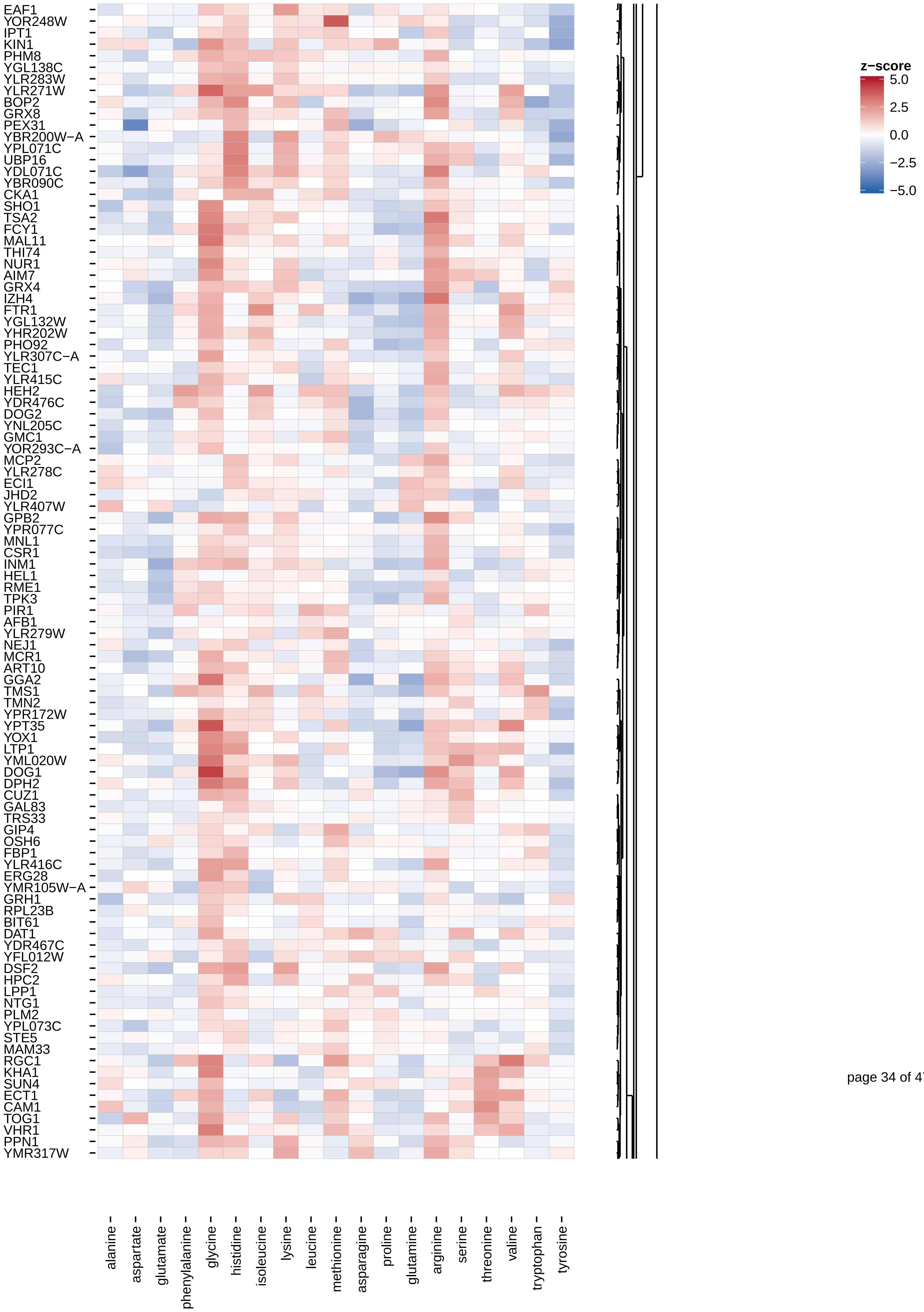

EAF1
YOR248W
IPT1
KIN1
PHM8
YGL138C
YLR283W
YLR271W
BOP2
GRX8
PEX31
YBR200W−A
YPL071C
UBP16
YDL071C
YBR090C
CKA1
SHO1
TSA2
FCY1
MAL11
THI74
NUR1
AIM7
GRX4
IZH4
FTR1
YGL132W
YHR202W
PHO92
YLR307C−A
TEC1
YLR415C
HEH2
YDR476C
DOG2
YNL205C
GMC1
YOR293C−A
MCP2
YLR278C
ECI1
JHD2
YLR407W
GPB2
YPR077C
MNL1
CSR1
INM1
HEL1
RME1
TPK3
PIR1
AFB1
YLR279W
NEJ1
MCR1
ART10
GGA2
TMS1
TMN2
YPR172W
YPT35
YOX1
LTP1
YML020W
DOG1
DPH2
CUZ1
GAL83
TRS33
GIP4
OSH6
FBP1
YLR416C
ERG28
YMR105W−A
GRH1
RPL23B
BIT61
DAT1
YDR467C
YFL012W
DSF2
HPC2
LPP1
NTG1
PLM2
YPL073C
STE5
MAM33
RGC1
KHA1
SUN4
ECT1
CAM1
TOG1
VHR1
PPN1
YMR317W
alanine
aspartate
glutamate
phenylalanine
glycine
histidine
isoleucine
lysine
leucine
methionine
asparagine
proline
glutamine
arginine
serine
threonine
valine
tryptophan
tyrosine
z−score
5.0
2.5
0.0
−2.5
−5.0

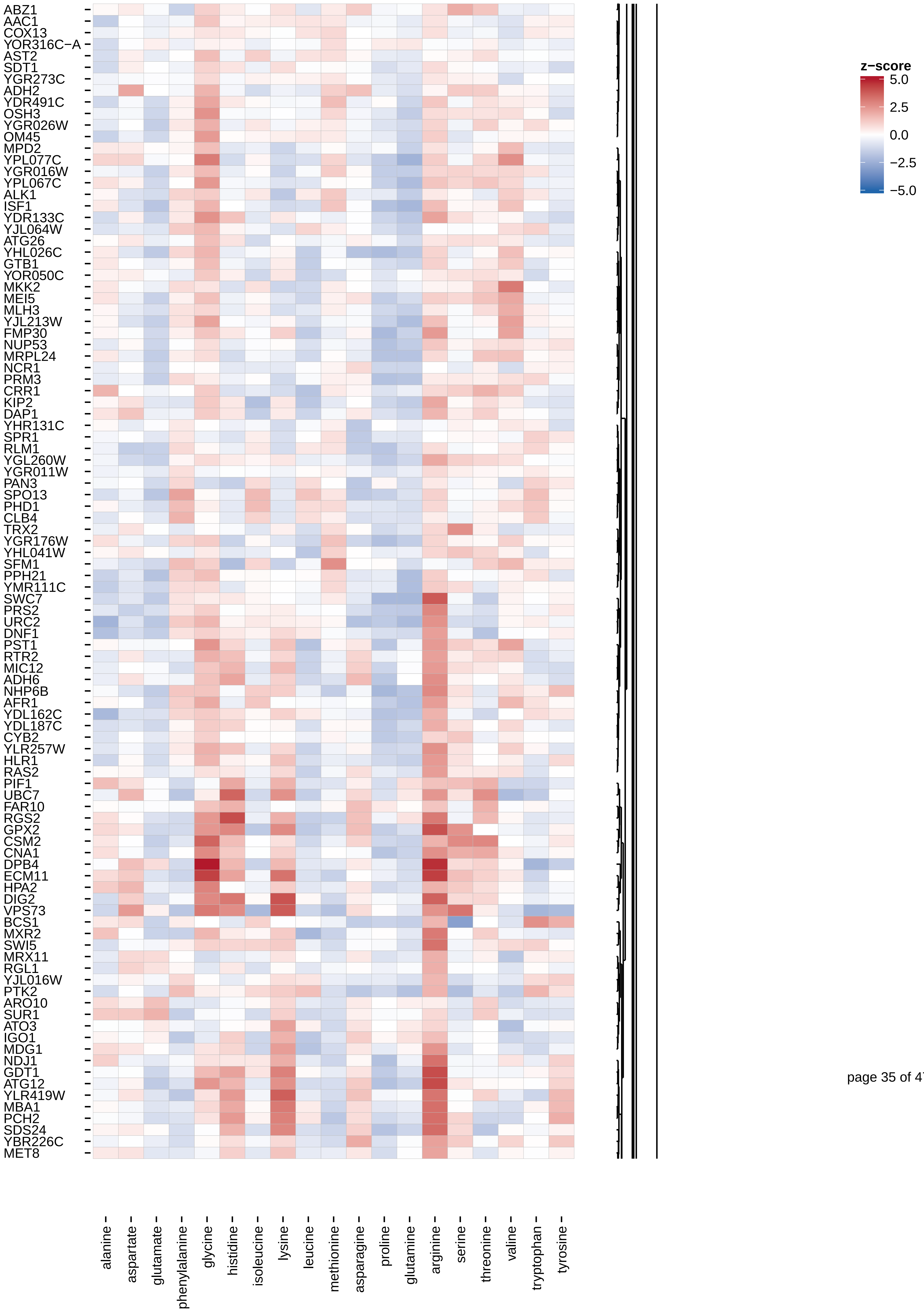
z−score
5.0
2.5
0.0
−2.5
−5.0
ABZ1
AAC1
COX13
YOR316C−A
AST2
SDT1
YGR273C
ADH2
YDR491C
OSH3
YGR026W
OM45
MPD2
YPL077C
YGR016W
YPL067C
ALK1
ISF1
YDR133C
YJL064W
ATG26
YHL026C
GTB1
YOR050C
MKK2
MEI5
MLH3
YJL213W
FMP30
NUP53
MRPL24
NCR1
PRM3
CRR1
KIP2
DAP1
YHR131C
SPR1
RLM1
YGL260W
YGR011W
PAN3
SPO13
PHD1
CLB4
TRX2
YGR176W
YHL041W
SFM1
PPH21
YMR111C
SWC7
PRS2
URC2
DNF1
PST1
RTR2
MIC12
ADH6
NHP6B
AFR1
YDL162C
YDL187C
CYB2
YLR257W
HLR1
RAS2
PIF1
UBC7
FAR10
RGS2
GPX2
CSM2
CNA1
DPB4
ECM11
HPA2
DIG2
VPS73
BCS1
MXR2
SWI5
MRX11
RGL1
YJL016W
PTK2
ARO10
SUR1
ATO3
IGO1
MDG1
NDJ1
GDT1
ATG12
YLR419W
MBA1
PCH2
SDS24
YBR226C
MET8
alanine
aspartate
glutamate
phenylalanine
glycine
histidine
isoleucine
lysine
leucine
methionine
asparagine
proline
glutamine
arginine
serine
threonine
valine
tryptophan
tyrosine

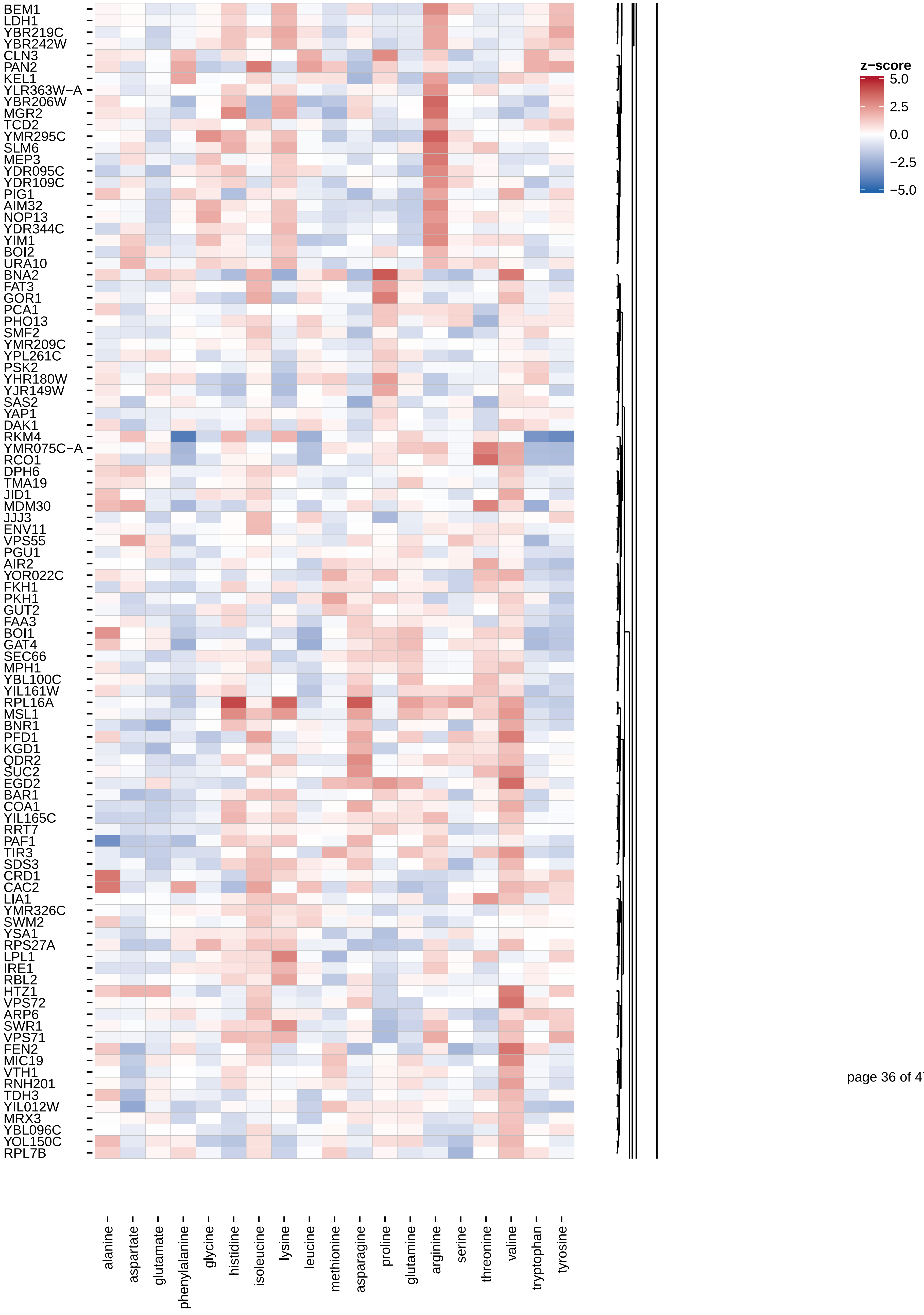
BEM1
LDH1
YBR219C
YBR242W
CLN3
PAN2
KEL1
YLR363W−A
YBR206W
MGR2
TCD2
YMR295C
SLM6
MEP3
YDR095C
YDR109C
PIG1
AIM32
NOP13
YDR344C
YIM1
BOI2
URA10
BNA2
FAT3
GOR1
PCA1
PHO13
SMF2
YMR209C
YPL261C
PSK2
YHR180W
YJR149W
SAS2
YAP1
DAK1
RKM4
YMR075C−A
RCO1
DPH6
TMA19
JID1
MDM30
JJJ3
ENV11
VPS55
PGU1
AIR2
YOR022C
FKH1
PKH1
GUT2
FAA3
BOI1
GAT4
SEC66
MPH1
YBL100C
YIL161W
RPL16A
MSL1
BNR1
PFD1
KGD1
QDR2
SUC2
EGD2
BAR1
COA1
YIL165C
RRT7
PAF1
TIR3
SDS3
CRD1
CAC2
LIA1
YMR326C
SWM2
YSA1
RPS27A
LPL1
IRE1
RBL2
HTZ1
VPS72
ARP6
SWR1
VPS71
FEN2
MIC19
VTH1
RNH201
TDH3
YIL012W
MRX3
YBL096C
YOL150C
RPL7B
alanine
aspartate
glutamate
phenylalanine
glycine
histidine
isoleucine
lysine
leucine
methionine
asparagine
proline
glutamine
arginine
serine
threonine
valine
tryptophan
tyrosine
z−score
5.0
2.5
0.0
−2.5
−5.0

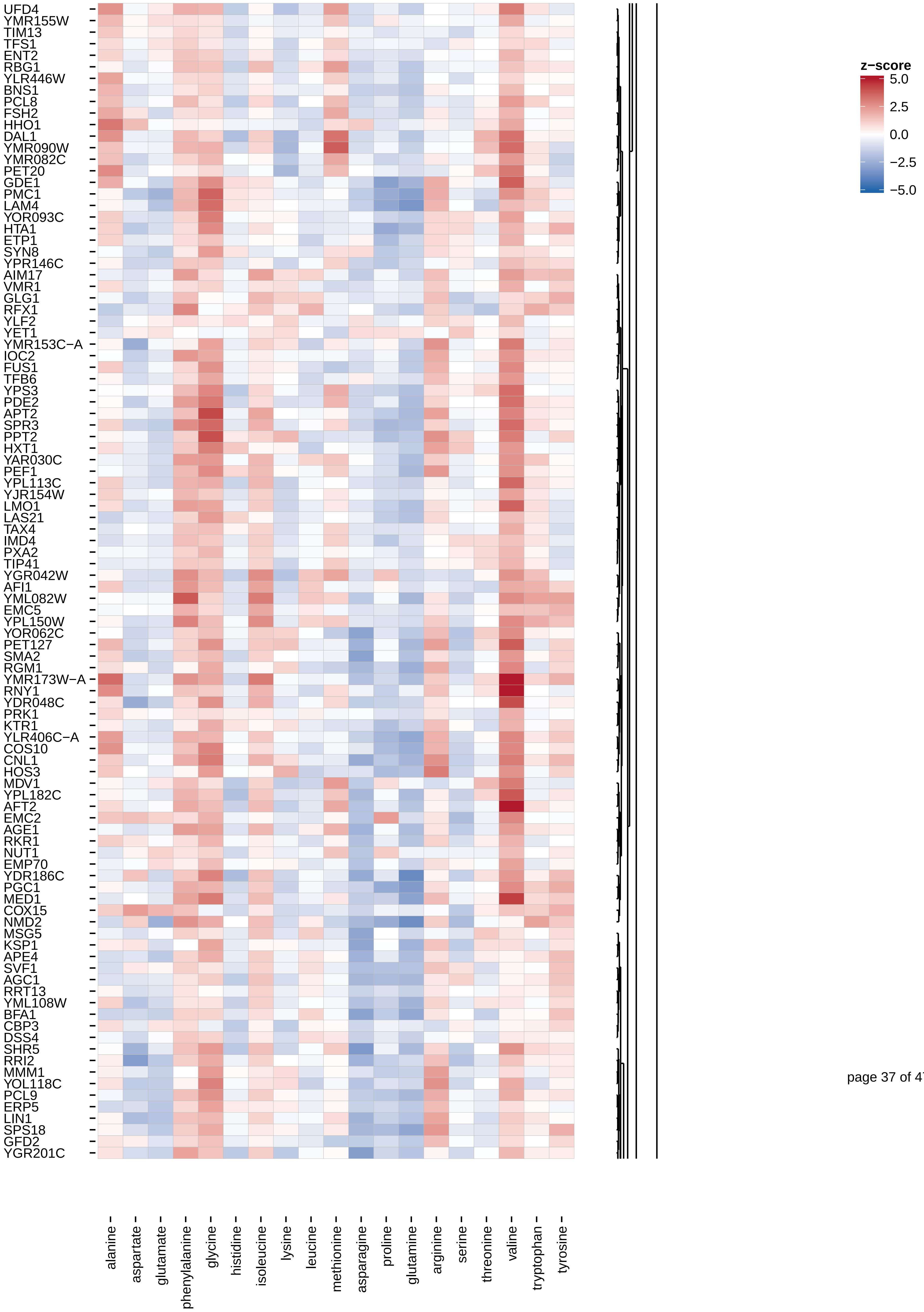

UFD4
YMR155W
TIM13
TFS1
ENT2
RBG1
YLR446W
BNS1
PCL8
FSH2
HHO1
DAL1
YMR090W
YMR082C
PET20
GDE1
PMC1
LAM4
YOR093C
HTA1
ETP1
SYN8
YPR146C
AIM17
VMR1
GLG1
RFX1
YLF2
YET1
YMR153C−A
IOC2
FUS1
TFB6
YPS3
PDE2
APT2
SPR3
PPT2
HXT1
YAR030C
PEF1
YPL113C
YJR154W
LMO1
LAS21
TAX4
IMD4
PXA2
TIP41
YGR042W
AFI1
YML082W
EMC5
YPL150W
YOR062C
PET127
SMA2
RGM1
YMR173W−A
RNY1
YDR048C
PRK1
KTR1
YLR406C−A
COS10
CNL1
HOS3
MDV1
YPL182C
AFT2
EMC2
AGE1
RKR1
NUT1
EMP70
YDR186C
PGC1
MED1
COX15
NMD2
MSG5
KSP1
APE4
SVF1
AGC1
RRT13
YML108W
BFA1
CBP3
DSS4
SHR5
RRI2
MMM1
YOL118C
PCL9
ERP5
LIN1
SPS18
GFD2
YGR201C
alanine
aspartate
glutamate
phenylalanine
glycine
histidine
isoleucine
lysine
leucine
methionine
asparagine
proline
glutamine
arginine
serine
threonine
valine
tryptophan
tyrosine
z−score
5.0
2.5
0.0
−2.5
−5.0

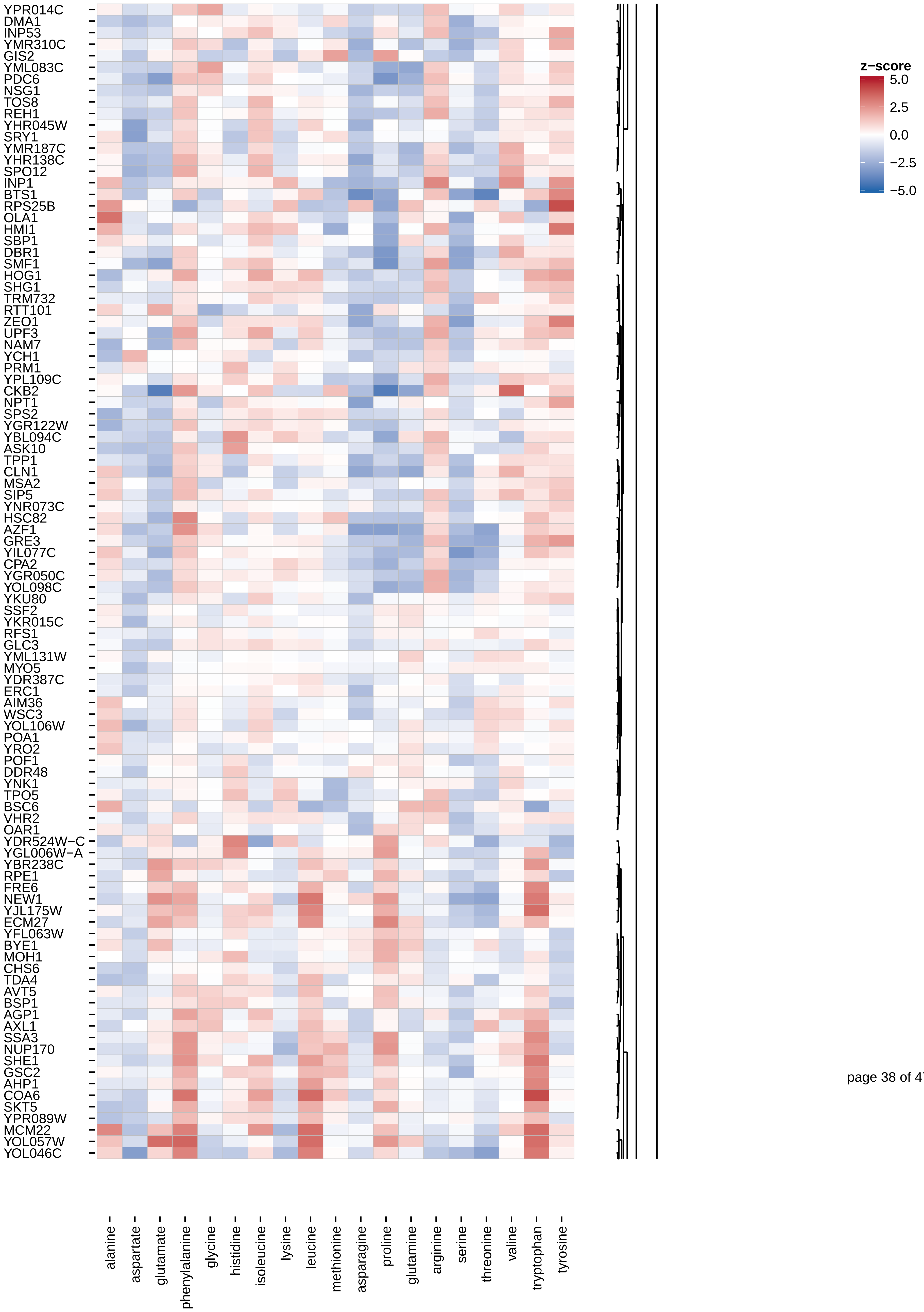
YPR014C
DMA1
INP53
YMR310C
GIS2
YML083C
PDC6
NSG1
TOS8
REH1
YHR045W
SRY1
YMR187C
YHR138C
SPO12
INP1
BTS1
RPS25B
OLA1
HMI1
SBP1
DBR1
SMF1
HOG1
SHG1
TRM732
RTT101
ZEO1
UPF3
NAM7
YCH1
PRM1
YPL109C
CKB2
NPT1
SPS2
YGR122W
YBL094C
ASK10
TPP1
CLN1
MSA2
SIP5
YNR073C
HSC82
AZF1
GRE3
YIL077C
CPA2
YGR050C
YOL098C
YKU80
SSF2
YKR015C
RFS1
GLC3
YML131W
MYO5
YDR387C
ERC1
AIM36
WSC3
YOL106W
POA1
YRO2
POF1
DDR48
YNK1
TPO5
BSC6
VHR2
OAR1
YDR524W−C
YGL006W−A
YBR238C
RPE1
FRE6
NEW1
YJL175W
ECM27
YFL063W
BYE1
MOH1
CHS6
TDA4
AVT5
BSP1
AGP1
AXL1
SSA3
NUP170
SHE1
GSC2
AHP1
COA6
SKT5
YPR089W
MCM22
YOL057W
YOL046C
alanine
aspartate
glutamate
phenylalanine
glycine
histidine
isoleucine
lysine
leucine
methionine
asparagine
proline
glutamine
arginine
serine
threonine
valine
tryptophan
tyrosine
z−score
5.0
2.5
0.0
−2.5
−5.0

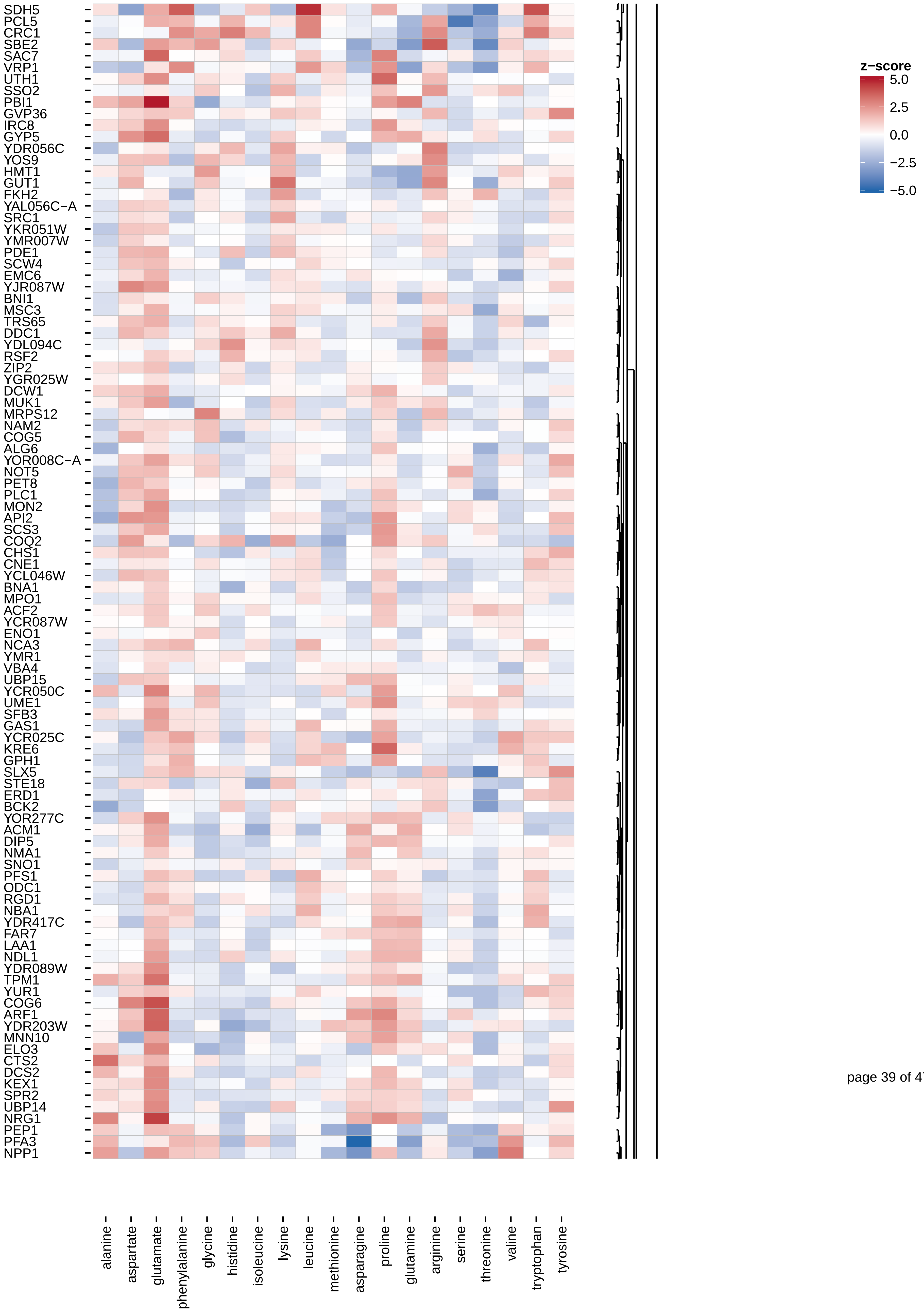
z−score
5.0
2.5
0.0
−2.5
−5.0
SDH5
PCL5
CRC1
SBE2
SAC7
VRP1
UTH1
SSO2
PBI1
GVP36
IRC8
GYP5
YDR056C
YOS9
HMT1
GUT1
FKH2
YAL056C−A
SRC1
YKR051W
YMR007W
PDE1
SCW4
EMC6
YJR087W
BNI1
MSC3
TRS65
DDC1
YDL094C
RSF2
ZIP2
YGR025W
DCW1
MUK1
MRPS12
NAM2
COG5
ALG6
YOR008C−A
NOT5
PET8
PLC1
MON2
API2
SCS3
COQ2
CHS1
CNE1
YCL046W
BNA1
MPO1
ACF2
YCR087W
ENO1
NCA3
YMR1
VBA4
UBP15
YCR050C
UME1
SFB3
GAS1
YCR025C
KRE6
GPH1
SLX5
STE18
ERD1
BCK2
YOR277C
ACM1
DIP5
NMA1
SNO1
PFS1
ODC1
RGD1
NBA1
YDR417C
FAR7
LAA1
NDL1
YDR089W
TPM1
YUR1
COG6
ARF1
YDR203W
MNN10
ELO3
CTS2
DCS2
KEX1
SPR2
UBP14
NRG1
PEP1
PFA3
NPP1
alanine
aspartate
glutamate
phenylalanine
glycine
histidine
isoleucine
lysine
leucine
methionine
asparagine
proline
glutamine
arginine
serine
threonine
valine
tryptophan
tyrosine

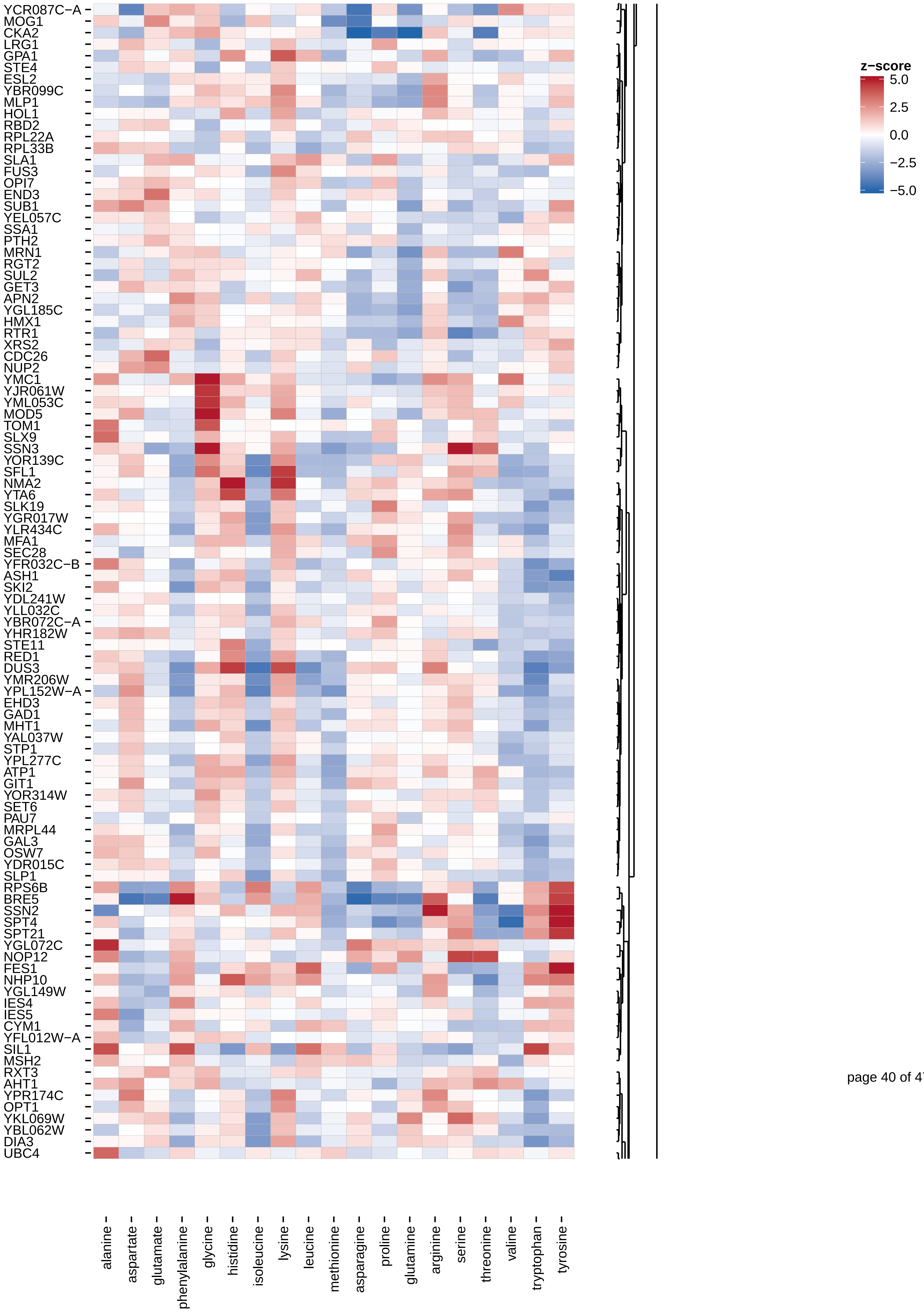

z−score
5.0
2.5
0.0
−2.5
−5.0
YCR087C−A
MOG1
CKA2
LRG1
GPA1
STE4
ESL2
YBR099C
MLP1
HOL1
RBD2
RPL22A
RPL33B
SLA1
FUS3
OPI7
END3
SUB1
YEL057C
SSA1
PTH2
MRN1
RGT2
SUL2
GET3
APN2
YGL185C
HMX1
RTR1
XRS2
CDC26
NUP2
YMC1
YJR061W
YML053C
MOD5
TOM1
SLX9
SSN3
YOR139C
SFL1
NMA2
YTA6
SLK19
YGR017W
YLR434C
MFA1
SEC28
YFR032C−B
ASH1
SKI2
YDL241W
YLL032C
YBR072C−A
YHR182W
STE11
RED1
DUS3
YMR206W
YPL152W−A
EHD3
GAD1
MHT1
YAL037W
STP1
YPL277C
ATP1
GIT1
YOR314W
SET6
PAU7
MRPL44
GAL3
OSW7
YDR015C
SLP1
RPS6B
BRE5
SSN2
SPT4
SPT21
YGL072C
NOP12
FES1
NHP10
YGL149W
IES4
IES5
CYM1
YFL012W−A
SIL1
MSH2
RXT3
AHT1
YPR174C
OPT1
YKL069W
YBL062W
DIA3
UBC4
alanine
aspartate
glutamate
phenylalanine
glycine
histidine
isoleucine
lysine
leucine
methionine
asparagine
proline
glutamine
arginine
serine
threonine
valine
tryptophan
tyrosine

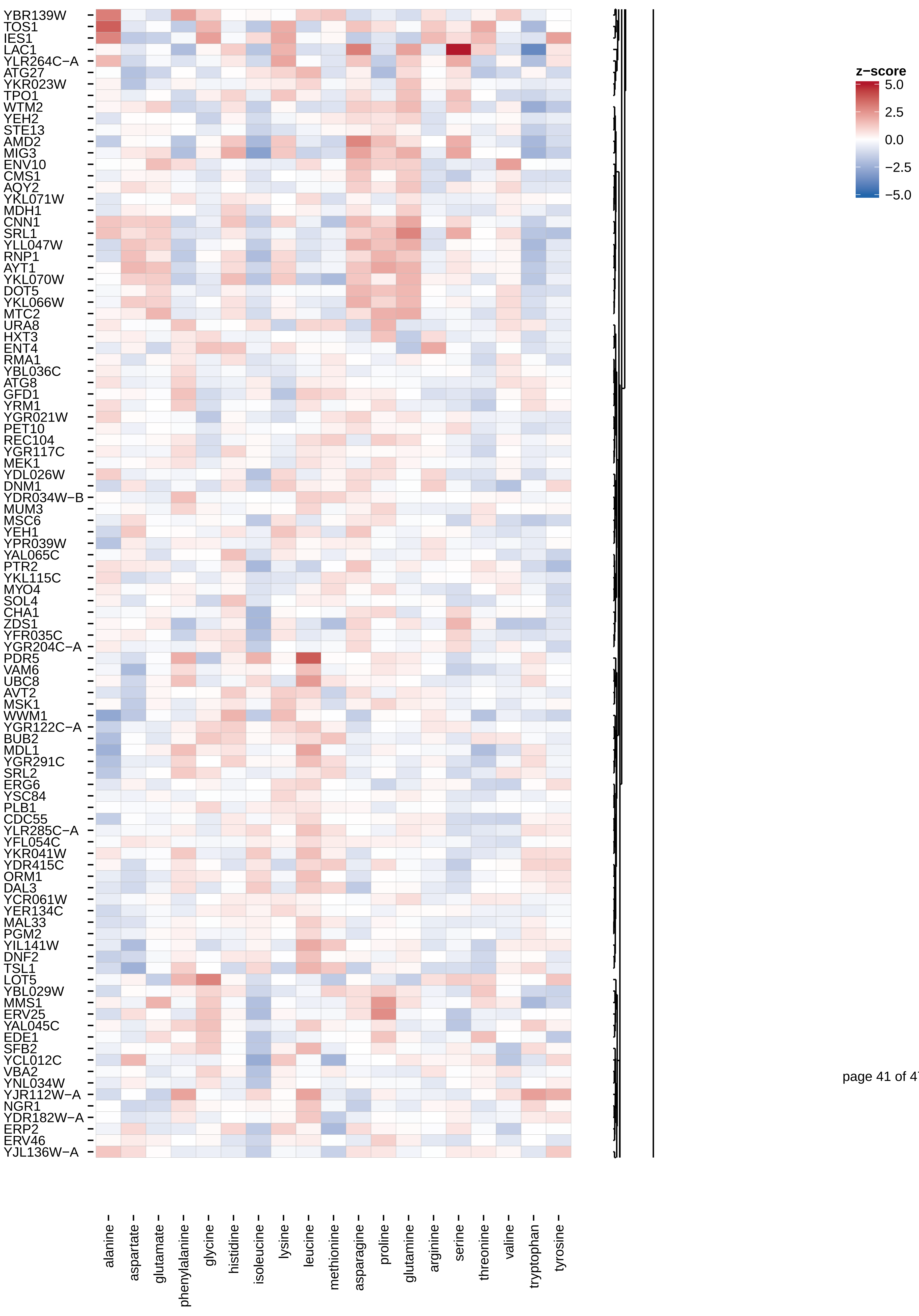
YBR139W
TOS1
IES1
LAC1
YLR264C−A
ATG27
YKR023W
TPO1
WTM2
YEH2
STE13
AMD2
MIG3
ENV10
CMS1
AQY2
YKL071W
MDH1
CNN1
SRL1
YLL047W
RNP1
AYT1
YKL070W
DOT5
YKL066W
MTC2
URA8
HXT3
ENT4
RMA1
YBL036C
ATG8
GFD1
YRM1
YGR021W
PET10
REC104
YGR117C
MEK1
YDL026W
DNM1
YDR034W−B
MUM3
MSC6
YEH1
YPR039W
YAL065C
PTR2
YKL115C
MYO4
SOL4
CHA1
ZDS1
YFR035C
YGR204C−A
PDR5
VAM6
UBC8
AVT2
MSK1
WWM1
YGR122C−A
BUB2
MDL1
YGR291C
SRL2
ERG6
YSC84
PLB1
CDC55
YLR285C−A
YFL054C
YKR041W
YDR415C
ORM1
DAL3
YCR061W
YER134C
MAL33
PGM2
YIL141W
DNF2
TSL1
LOT5
YBL029W
MMS1
ERV25
YAL045C
EDE1
SFB2
YCL012C
VBA2
YNL034W
YJR112W−A
NGR1
YDR182W−A
ERP2
ERV46
YJL136W−A
alanine
aspartate
glutamate
phenylalanine
glycine
histidine
isoleucine
lysine
leucine
methionine
asparagine
proline
glutamine
arginine
serine
threonine
valine
tryptophan
tyrosine
z−score
5.0
2.5
0.0
−2.5
−5.0

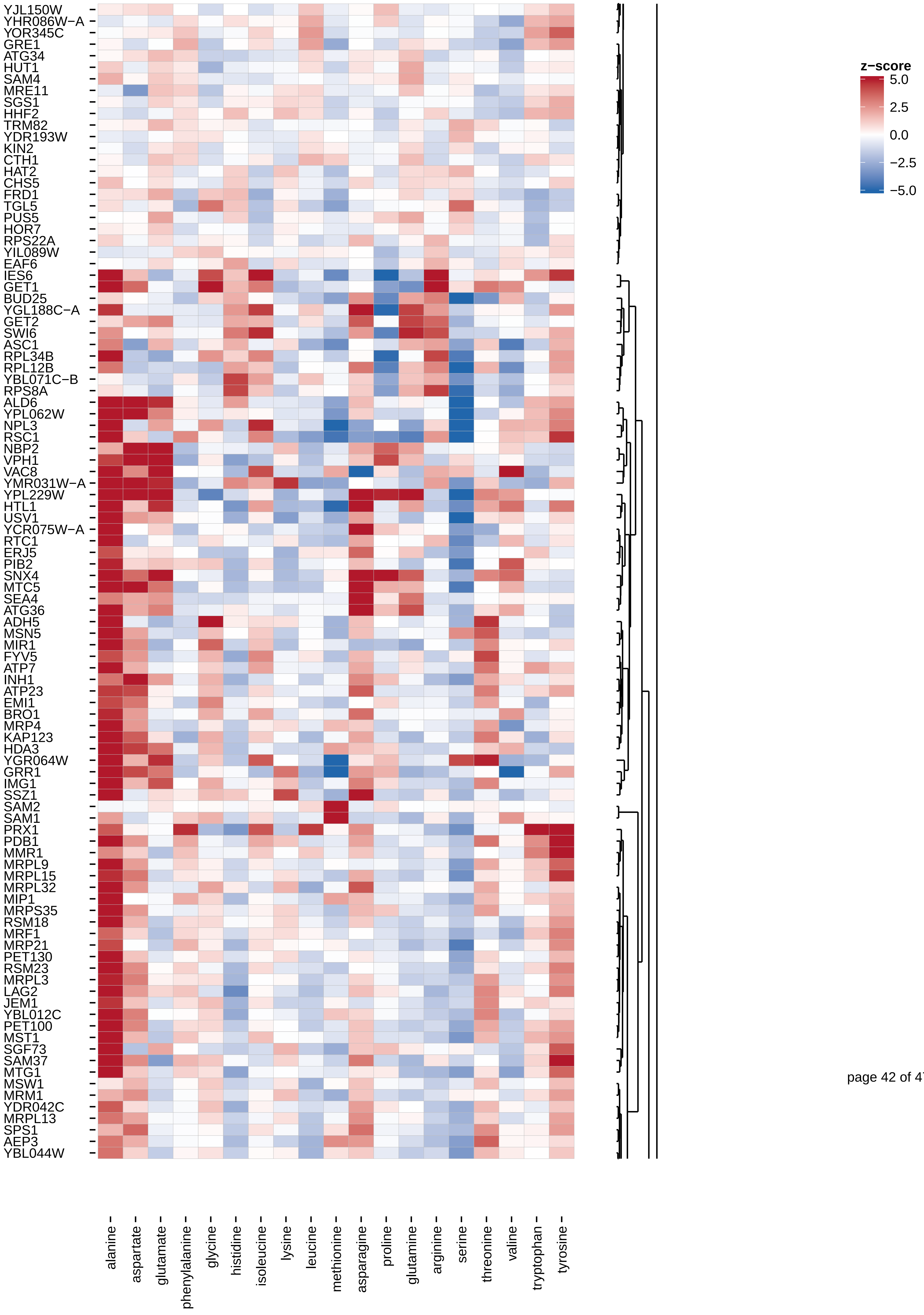

z−score
5.0
2.5
0.0
−2.5
−5.0
YJL150W
YHR086W−A
YOR345C
GRE1
ATG34
HUT1
SAM4
MRE11
SGS1
HHF2
TRM82
YDR193W
KIN2
CTH1
HAT2
CHS5
FRD1
TGL5
PUS5
HOR7
RPS22A
YIL089W
EAF6
IES6
GET1
BUD25
YGL188C−A
GET2
SWI6
ASC1
RPL34B
RPL12B
YBL071C−B
RPS8A
ALD6
YPL062W
NPL3
RSC1
NBP2
VPH1
VAC8
YMR031W−A
YPL229W
HTL1
USV1
YCR075W−A
RTC1
ERJ5
PIB2
SNX4
MTC5
SEA4
ATG36
ADH5
MSN5
MIR1
FYV5
ATP7
INH1
ATP23
EMI1
BRO1
MRP4
KAP123
HDA3
YGR064W
GRR1
IMG1
SSZ1
SAM2
SAM1
PRX1
PDB1
MMR1
MRPL9
MRPL15
MRPL32
MIP1
MRPS35
RSM18
MRF1
MRP21
PET130
RSM23
MRPL3
LAG2
JEM1
YBL012C
PET100
MST1
SGF73
SAM37
MTG1
MSW1
MRM1
YDR042C
MRPL13
SPS1
AEP3
YBL044W
alanine
aspartate
glutamate
phenylalanine
glycine
histidine
isoleucine
lysine
leucine
methionine
asparagine
proline
glutamine
arginine
serine
threonine
valine
tryptophan
tyrosine

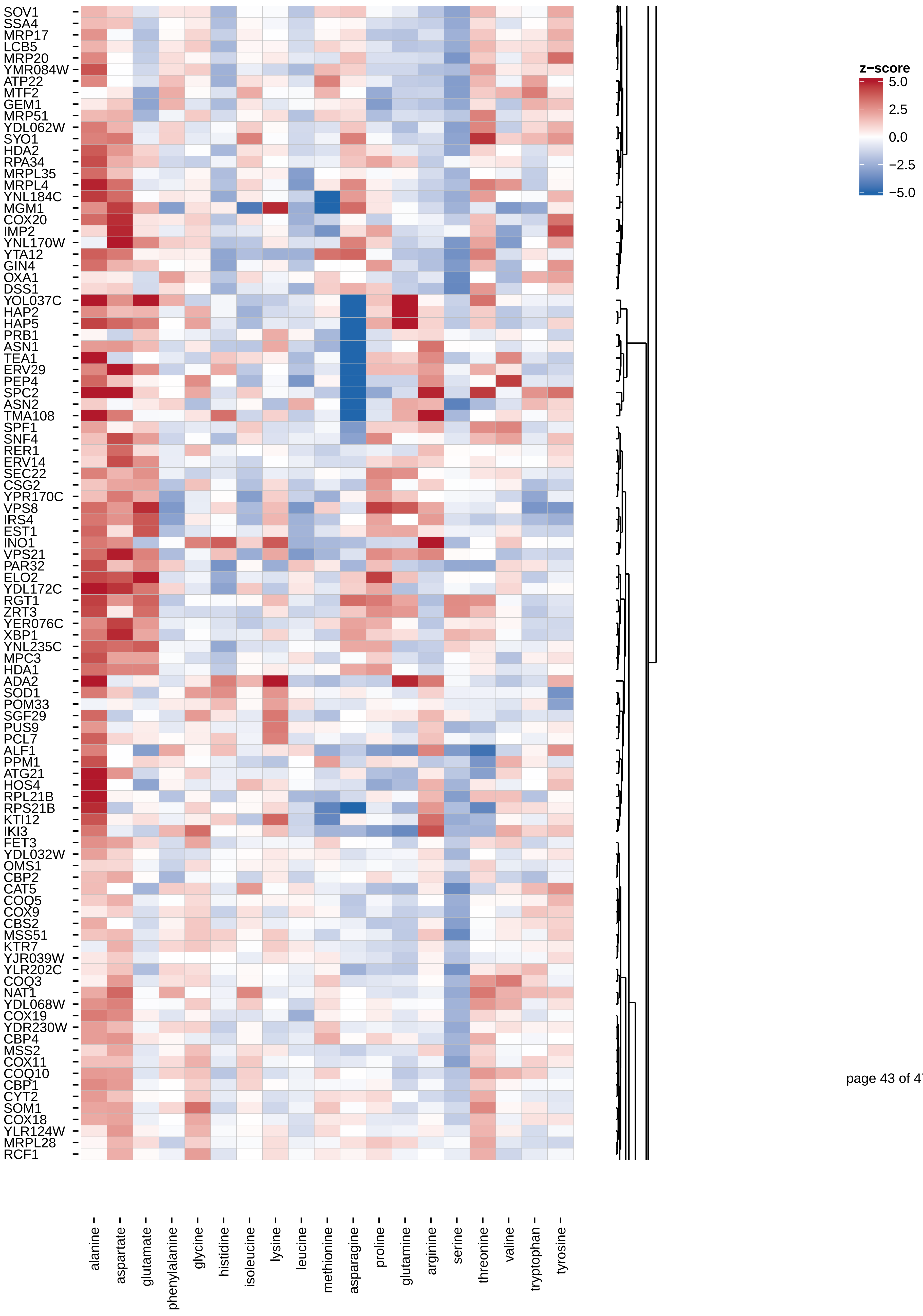
z−score
5.0
2.5
0.0
−2.5
−5.0
SOV1
SSA4
MRP17
LCB5
MRP20
YMR084W
ATP22
MTF2
GEM1
MRP51
YDL062W
SYO1
HDA2
RPA34
MRPL35
MRPL4
YNL184C
MGM1
COX20
IMP2
YNL170W
YTA12
GIN4
OXA1
DSS1
YOL037C
HAP2
HAP5
PRB1
ASN1
TEA1
ERV29
PEP4
SPC2
ASN2
TMA108
SPF1
SNF4
RER1
ERV14
SEC22
CSG2
YPR170C
VPS8
IRS4
EST1
INO1
VPS21
PAR32
ELO2
YDL172C
RGT1
ZRT3
YER076C
XBP1
YNL235C
MPC3
HDA1
ADA2
SOD1
POM33
SGF29
PUS9
PCL7
ALF1
PPM1
ATG21
HOS4
RPL21B
RPS21B
KTI12
IKI3
FET3
YDL032W
OMS1
CBP2
CAT5
COQ5
COX9
CBS2
MSS51
KTR7
YJR039W
YLR202C
COQ3
NAT1
YDL068W
COX19
YDR230W
CBP4
MSS2
COX11
COQ10
CBP1
CYT2
SOM1
COX18
YLR124W
MRPL28
RCF1
alanine
aspartate
glutamate
phenylalanine
glycine
histidine
isoleucine
lysine
leucine
methionine
asparagine
proline
glutamine
arginine
serine
threonine
valine
tryptophan
tyrosine

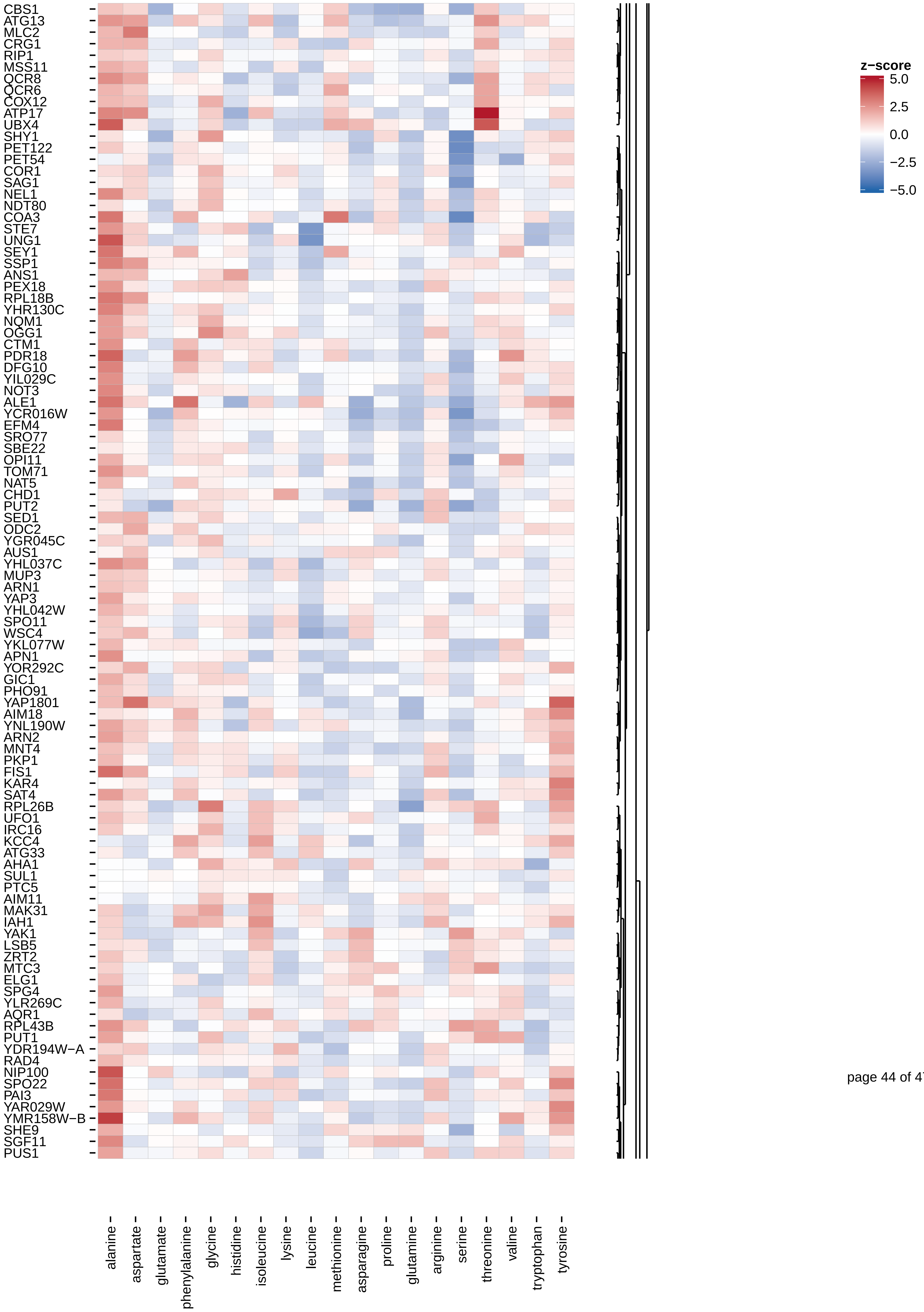

z−score
5.0
2.5
0.0
−2.5
−5.0
CBS1
ATG13
MLC2
CRG1
RIP1
MSS11
QCR8
QCR6
COX12
ATP17
UBX4
SHY1
PET122
PET54
COR1
SAG1
NEL1
NDT80
COA3
STE7
UNG1
SEY1
SSP1
ANS1
PEX18
RPL18B
YHR130C
NQM1
OGG1
CTM1
PDR18
DFG10
YIL029C
NOT3
ALE1
YCR016W
EFM4
SRO77
SBE22
OPI11
TOM71
NAT5
CHD1
PUT2
SED1
ODC2
YGR045C
AUS1
YHL037C
MUP3
ARN1
YAP3
YHL042W
SPO11
WSC4
YKL077W
APN1
YOR292C
GIC1
PHO91
YAP1801
AIM18
YNL190W
ARN2
MNT4
PKP1
FIS1
KAR4
SAT4
RPL26B
UFO1
IRC16
KCC4
ATG33
AHA1
SUL1
PTC5
AIM11
MAK31
IAH1
YAK1
LSB5
ZRT2
MTC3
ELG1
SPG4
YLR269C
AQR1
RPL43B
PUT1
YDR194W−A
RAD4
NIP100
SPO22
PAI3
YAR029W
YMR158W−B
SHE9
SGF11
PUS1
alanine
aspartate
glutamate
phenylalanine
glycine
histidine
isoleucine
lysine
leucine
methionine
asparagine
proline
glutamine
arginine
serine
threonine
valine
tryptophan
tyrosine

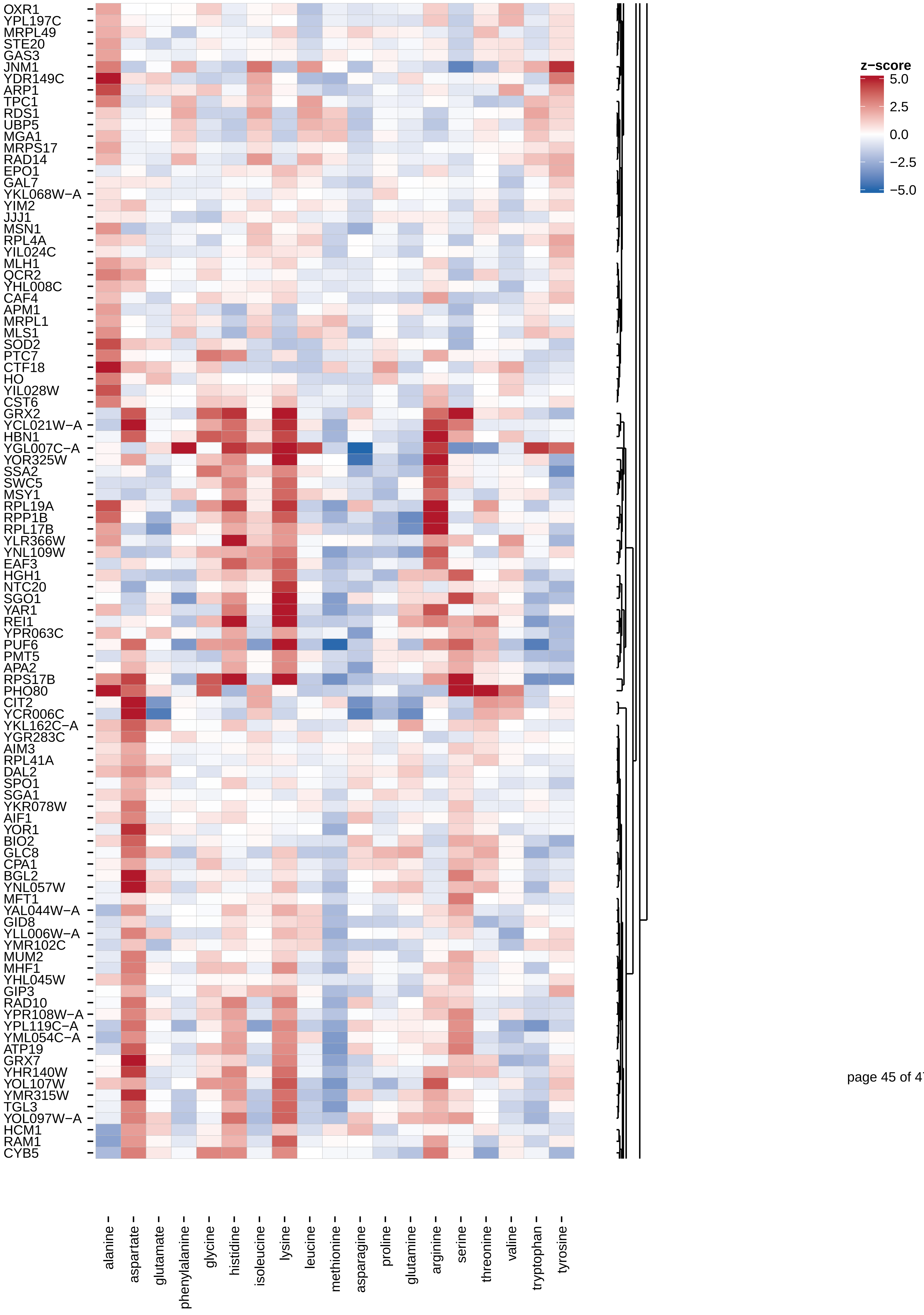
OXR1
YPL197C
MRPL49
STE20
GAS3
JNM1
YDR149C
ARP1
TPC1
RDS1
UBP5
MGA1
MRPS17
RAD14
EPO1
GAL7
YKL068W−A
YIM2
JJJ1
MSN1
RPL4A
YIL024C
MLH1
QCR2
YHL008C
CAF4
APM1
MRPL1
MLS1
SOD2
PTC7
CTF18
HO
YIL028W
CST6
GRX2
YCL021W−A
HBN1
YGL007C−A
YOR325W
SSA2
SWC5
MSY1
RPL19A
RPP1B
RPL17B
YLR366W
YNL109W
EAF3
HGH1
NTC20
SGO1
YAR1
REI1
YPR063C
PUF6
PMT5
APA2
RPS17B
PHO80
CIT2
YCR006C
YKL162C−A
YGR283C
AIM3
RPL41A
DAL2
SPO1
SGA1
YKR078W
AIF1
YOR1
BIO2
GLC8
CPA1
BGL2
YNL057W
MFT1
YAL044W−A
GID8
YLL006W−A
YMR102C
MUM2
MHF1
YHL045W
GIP3
RAD10
YPR108W−A
YPL119C−A
YML054C−A
ATP19
GRX7
YHR140W
YOL107W
YMR315W
TGL3
YOL097W−A
HCM1
RAM1
CYB5
alanine
aspartate
glutamate
phenylalanine
glycine
histidine
isoleucine
lysine
leucine
methionine
asparagine
proline
glutamine
arginine
serine
threonine
valine
tryptophan
tyrosine
z−score
5.0
2.5
0.0
−2.5
−5.0

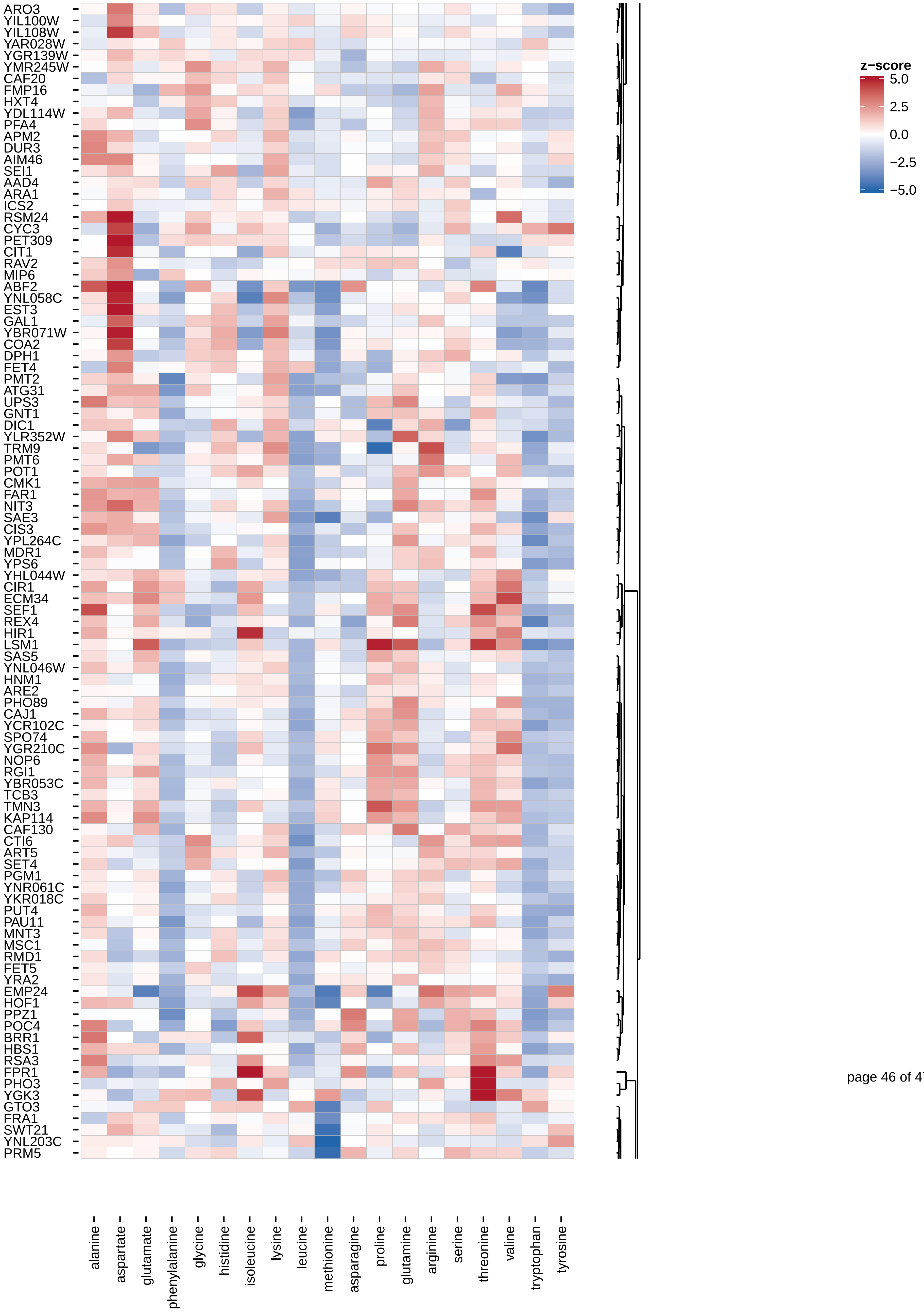
ARO3
YIL100W
YIL108W
YAR028W
YGR139W
YMR245W
CAF20
FMP16
HXT4
YDL114W
PFA4
APM2
DUR3
AIM46
SEI1
AAD4
ARA1
ICS2
RSM24
CYC3
PET309
CIT1
RAV2
MIP6
ABF2
YNL058C
EST3
GAL1
YBR071W
COA2
DPH1
FET4
PMT2
ATG31
UPS3
GNT1
DIC1
YLR352W
TRM9
PMT6
POT1
CMK1
FAR1
NIT3
SAE3
CIS3
YPL264C
MDR1
YPS6
YHL044W
CIR1
ECM34
SEF1
REX4
HIR1
LSM1
SAS5
YNL046W
HNM1
ARE2
PHO89
CAJ1
YCR102C
SPO74
YGR210C
NOP6
RGI1
YBR053C
TCB3
TMN3
KAP114
CAF130
CTI6
ART5
SET4
PGM1
YNR061C
YKR018C
PUT4
PAU11
MNT3
MSC1
RMD1
FET5
YRA2
EMP24
HOF1
PPZ1
POC4
BRR1
HBS1
RSA3
FPR1
PHO3
YGK3
GTO3
FRA1
SWT21
YNL203C
PRM5
alanine
aspartate
glutamate
phenylalanine
glycine
histidine
isoleucine
lysine
leucine
methionine
asparagine
proline
glutamine
arginine
serine
threonine
valine
tryptophan
tyrosine
z−score
5.0
2.5
0.0
−2.5
−5.0

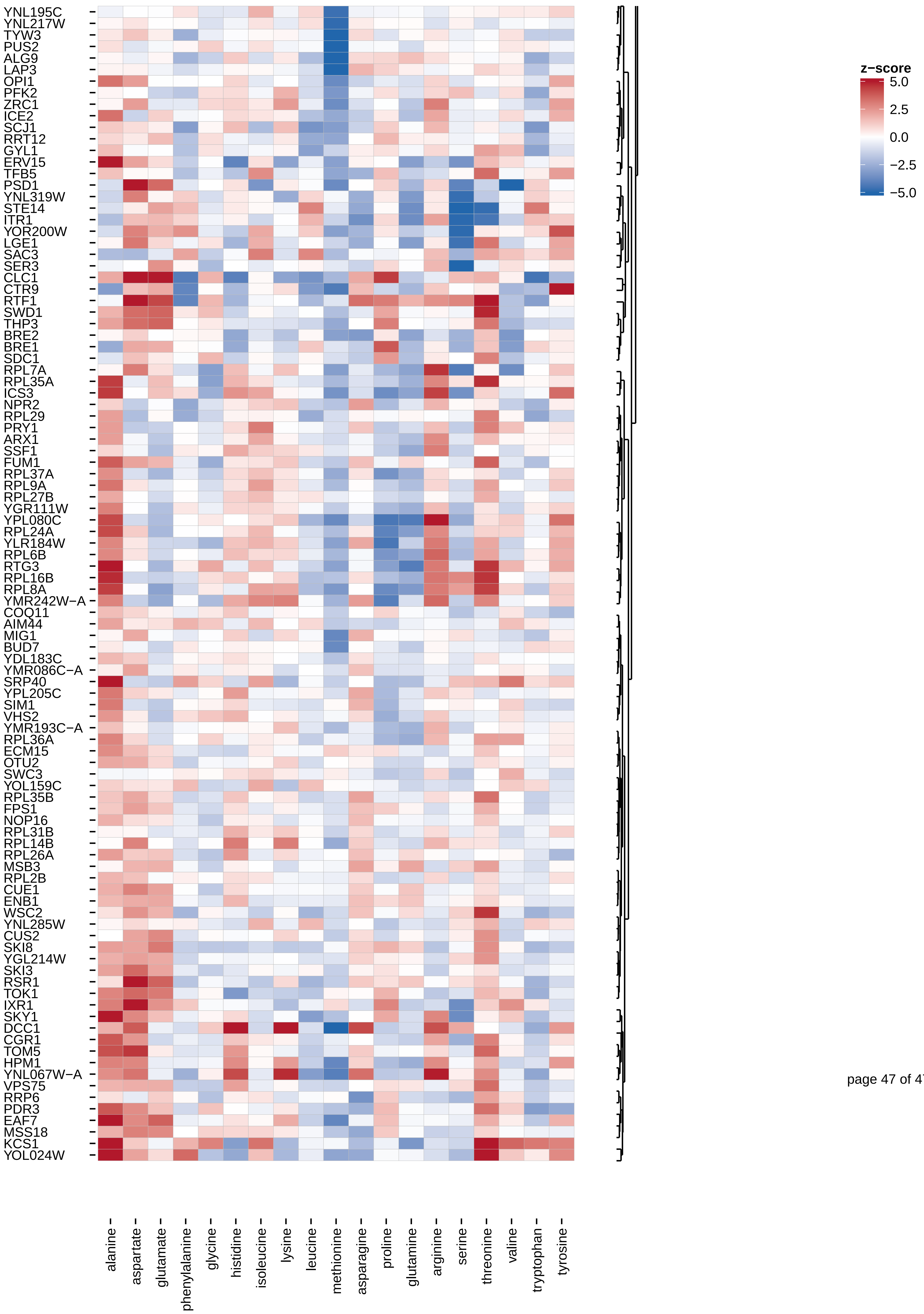

YNL195C
YNL217W
TYW3
PUS2
ALG9
LAP3
OPI1
PFK2
ZRC1
ICE2
SCJ1
RRT12
GYL1
ERV15
TFB5
PSD1
YNL319W
STE14
ITR1
YOR200W
LGE1
SAC3
SER3
CLC1
CTR9
RTF1
SWD1
THP3
BRE2
BRE1
SDC1
RPL7A
RPL35A
ICS3
NPR2
RPL29
PRY1
ARX1
SSF1
FUM1
RPL37A
RPL9A
RPL27B
YGR111W
YPL080C
RPL24A
YLR184W
RPL6B
RTG3
RPL16B
RPL8A
YMR242W−A
COQ11
AIM44
MIG1
BUD7
YDL183C
YMR086C−A
SRP40
YPL205C
SIM1
VHS2
YMR193C−A
RPL36A
ECM15
OTU2
SWC3
YOL159C
RPL35B
FPS1
NOP16
RPL31B
RPL14B
RPL26A
MSB3
RPL2B
CUE1
ENB1
WSC2
YNL285W
CUS2
SKI8
YGL214W
SKI3
RSR1
TOK1
IXR1
SKY1
DCC1
CGR1
TOM5
HPM1
YNL067W−A
VPS75
RRP6
PDR3
EAF7
MSS18
KCS1
YOL024W
alanine
aspartate
glutamate
phenylalanine
glycine
histidine
isoleucine
lysine
leucine
methionine
asparagine
proline
glutamine
arginine
serine
threonine
valine
tryptophan
tyrosine
z−score
5.0
2.5
0.0
−2.5
−5.0

page 47 of 47

280 similarity clusters informative of gene function

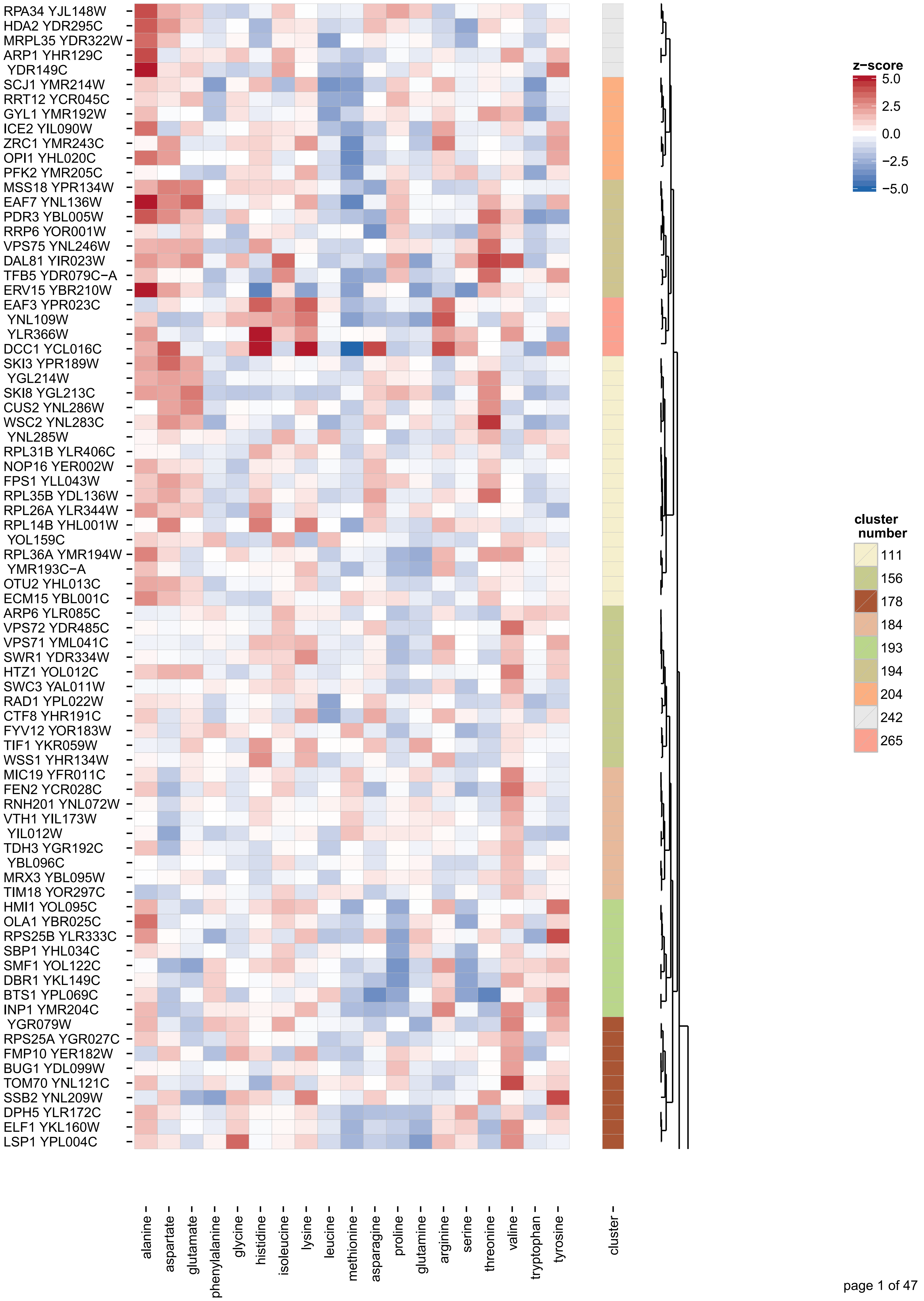

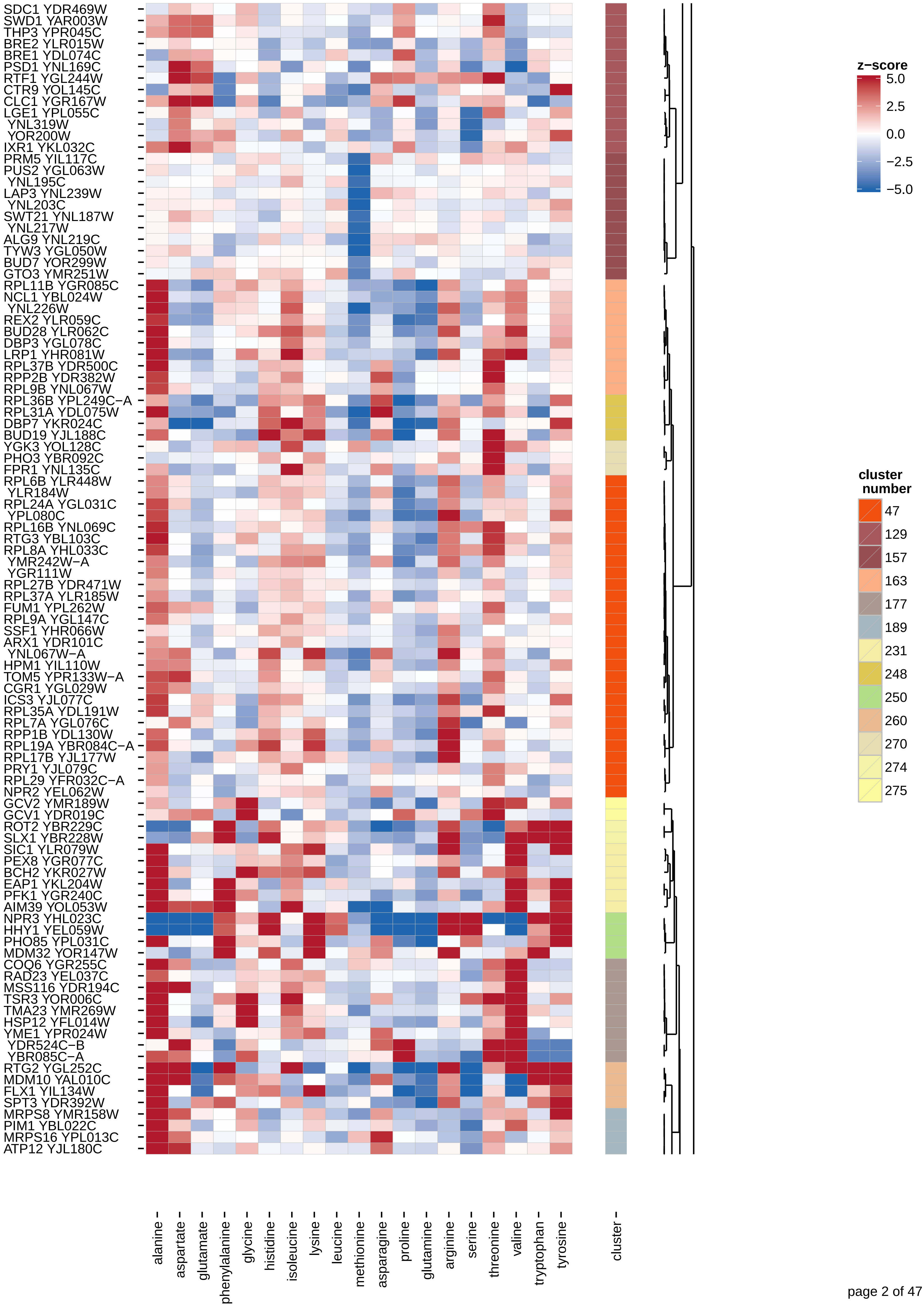
SDC1 YDR469W
SWD1 YAR003W
THP3 YPR045C
BRE2 YLR015W
BRE1 YDL074C
PSD1 YNL169C
RTF1 YGL244W
CTR9 YOL145C
CLC1 YGR167W
LGE1 YPL055C
YNL319W
YOR200W
IXR1 YKL032C
PRM5 YIL117C
PUS2 YGL063W
YNL195C
LAP3 YNL239W
YNL203C
SWT21 YNL187W
YNL217W
ALG9 YNL219C
TYW3 YGL050W
BUD7 YOR299W
GTO3 YMR251W
RPL11B YGR085C
NCL1 YBL024W
YNL226W
REX2 YLR059C
BUD28 YLR062C
DBP3 YGL078C
LRP1 YHR081W
RPL37B YDR500C
RPP2B YDR382W
RPL9B YNL067W
RPL36B YPL249C−A
RPL31A YDL075W
DBP7 YKR024C
BUD19 YJL188C
YGK3 YOL128C
PHO3 YBR092C
FPR1 YNL135C
RPL6B YLR448W
YLR184W
RPL24A YGL031C
YPL080C
RPL16B YNL069C
RTG3 YBL103C
RPL8A YHL033C
YMR242W−A
YGR111W
RPL27B YDR471W
RPL37A YLR185W
FUM1 YPL262W
RPL9A YGL147C
SSF1 YHR066W
ARX1 YDR101C
YNL067W−A
HPM1 YIL110W
TOM5 YPR133W−A
CGR1 YGL029W
ICS3 YJL077C
RPL35A YDL191W
RPL7A YGL076C
RPP1B YDL130W
RPL19A YBR084C−A
RPL17B YJL177W
PRY1 YJL079C
RPL29 YFR032C−A
NPR2 YEL062W
GCV2 YMR189W
GCV1 YDR019C
ROT2 YBR229C
SLX1 YBR228W
SIC1 YLR079W
PEX8 YGR077C
BCH2 YKR027W
EAP1 YKL204W
PFK1 YGR240C
AIM39 YOL053W
NPR3 YHL023C
HHY1 YEL059W
PHO85 YPL031C
MDM32 YOR147W
COQ6 YGR255C
RAD23 YEL037C
MSS116 YDR194C
TSR3 YOR006C
TMA23 YMR269W
HSP12 YFL014W
YME1 YPR024W
YDR524C−B
YBR085C−A
RTG2 YGL252C
MDM10 YAL010C
FLX1 YIL134W
SPT3 YDR392W
MRPS8 YMR158W
PIM1 YBL022C
MRPS16 YPL013C
ATP12 YJL180C
alanine
aspartate
glutamate
phenylalanine
glycine
histidine
isoleucine
lysine
leucine
methionine
asparagine
proline
glutamine
arginine
serine
threonine
valine
tryptophan
tyrosine
cluster
z−score
5.0
2.5
0.0
−2.5
−5.0
cluster number
47
129
157
163
177
189
231
248
250
260
270
274
275

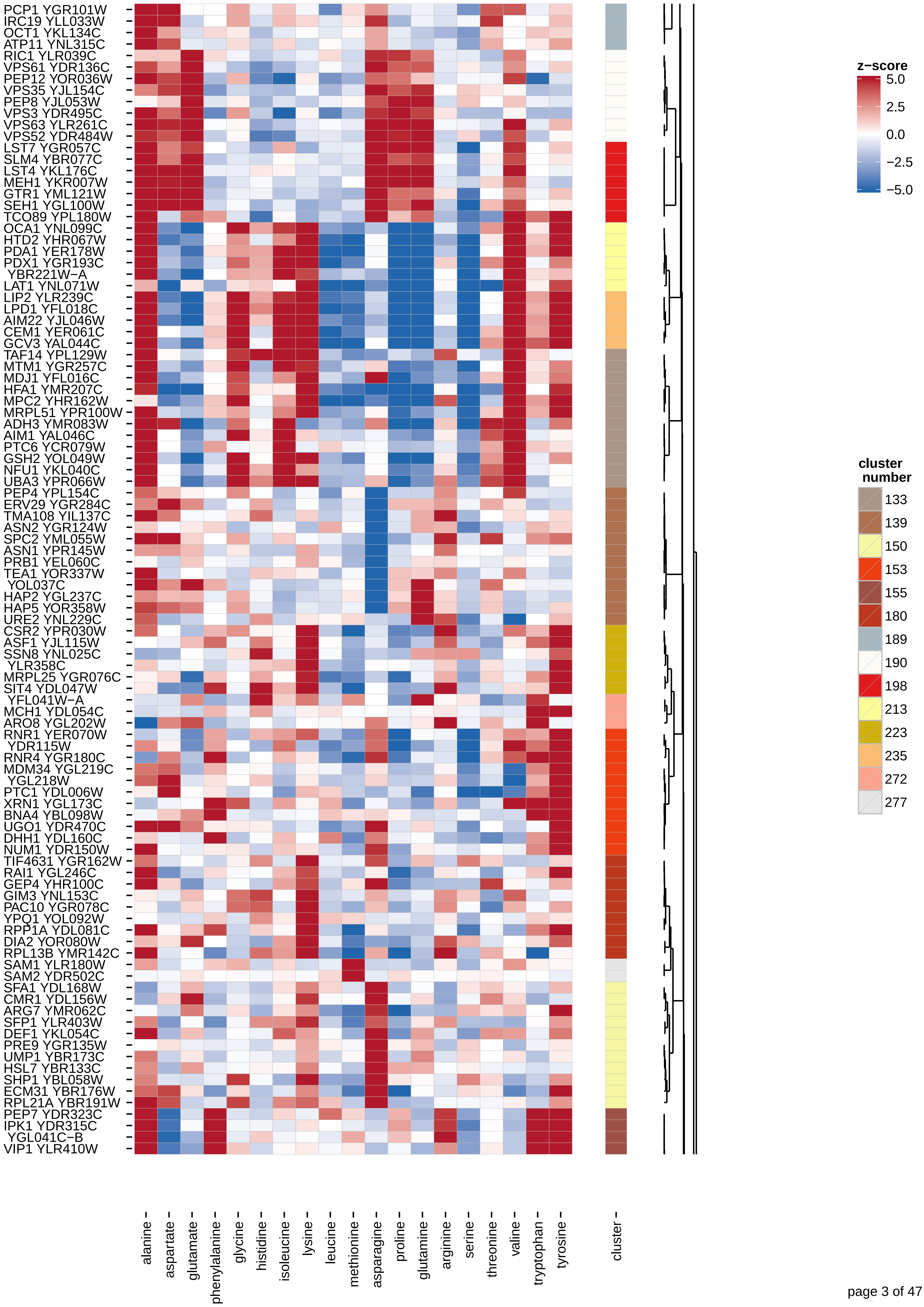

PCP1 YGR101W
IRC19 YLL033W
OCT1 YKL134C
ATP11 YNL315C
RIC1 YLR039C
VPS61 YDR136C
PEP12 YOR036W
VPS35 YJL154C
PEP8 YJL053W
VPS3 YDR495C
VPS63 YLR261C
VPS52 YDR484W
LST7 YGR057C
SLM4 YBR077C
LST4 YKL176C
MEH1 YKR007W
GTR1 YML121W
SEH1 YGL100W
TCO89 YPL180W
OCA1 YNL099C
HTD2 YHR067W
PDA1 YER178W
PDX1 YGR193C
YBR221W−A
LAT1 YNL071W
LIP2 YLR239C
LPD1 YFL018C
AIM22 YJL046W
CEM1 YER061C
GCV3 YAL044C
TAF14 YPL129W
MTM1 YGR257C
MDJ1 YFL016C
HFA1 YMR207C
MPC2 YHR162W
MRPL51 YPR100W
ADH3 YMR083W
AIM1 YAL046C
PTC6 YCR079W
GSH2 YOL049W
NFU1 YKL040C
UBA3 YPR066W
PEP4 YPL154C
ERV29 YGR284C
TMA108 YIL137C
ASN2 YGR124W
SPC2 YML055W
ASN1 YPR145W
PRB1 YEL060C
TEA1 YOR337W
YOL037C
HAP2 YGL237C
HAP5 YOR358W
URE2 YNL229C
CSR2 YPR030W
ASF1 YJL115W
SSN8 YNL025C
YLR358C
MRPL25 YGR076C
SIT4 YDL047W
YFL041W−A
MCH1 YDL054C
ARO8 YGL202W
RNR1 YER070W
YDR115W
RNR4 YGR180C
MDM34 YGL219C
YGL218W
PTC1 YDL006W
XRN1 YGL173C
BNA4 YBL098W
UGO1 YDR470C
DHH1 YDL160C
NUM1 YDR150W
TIF4631 YGR162W
RAI1 YGL246C
GEP4 YHR100C
GIM3 YNL153C
PAC10 YGR078C
YPQ1 YOL092W
RPP1A YDL081C
DIA2 YOR080W
RPL13B YMR142C
SAM1 YLR180W
SAM2 YDR502C
SFA1 YDL168W
CMR1 YDL156W
ARG7 YMR062C
SFP1 YLR403W
DEF1 YKL054C
PRE9 YGR135W
UMP1 YBR173C
HSL7 YBR133C
SHP1 YBL058W
ECM31 YBR176W
RPL21A YBR191W
PEP7 YDR323C
IPK1 YDR315C
YGL041C−B
VIP1 YLR410W
alanine
aspartate
glutamate
phenylalanine
glycine
histidine
isoleucine
lysine
leucine
methionine
asparagine
proline
glutamine
arginine
serine
threonine
valine
tryptophan
tyrosine
cluster
z−score
5.0
2.5
0.0
−2.5
−5.0
cluster number
133
139
150
153
155
180
189
190
198
213
223
235
272
277

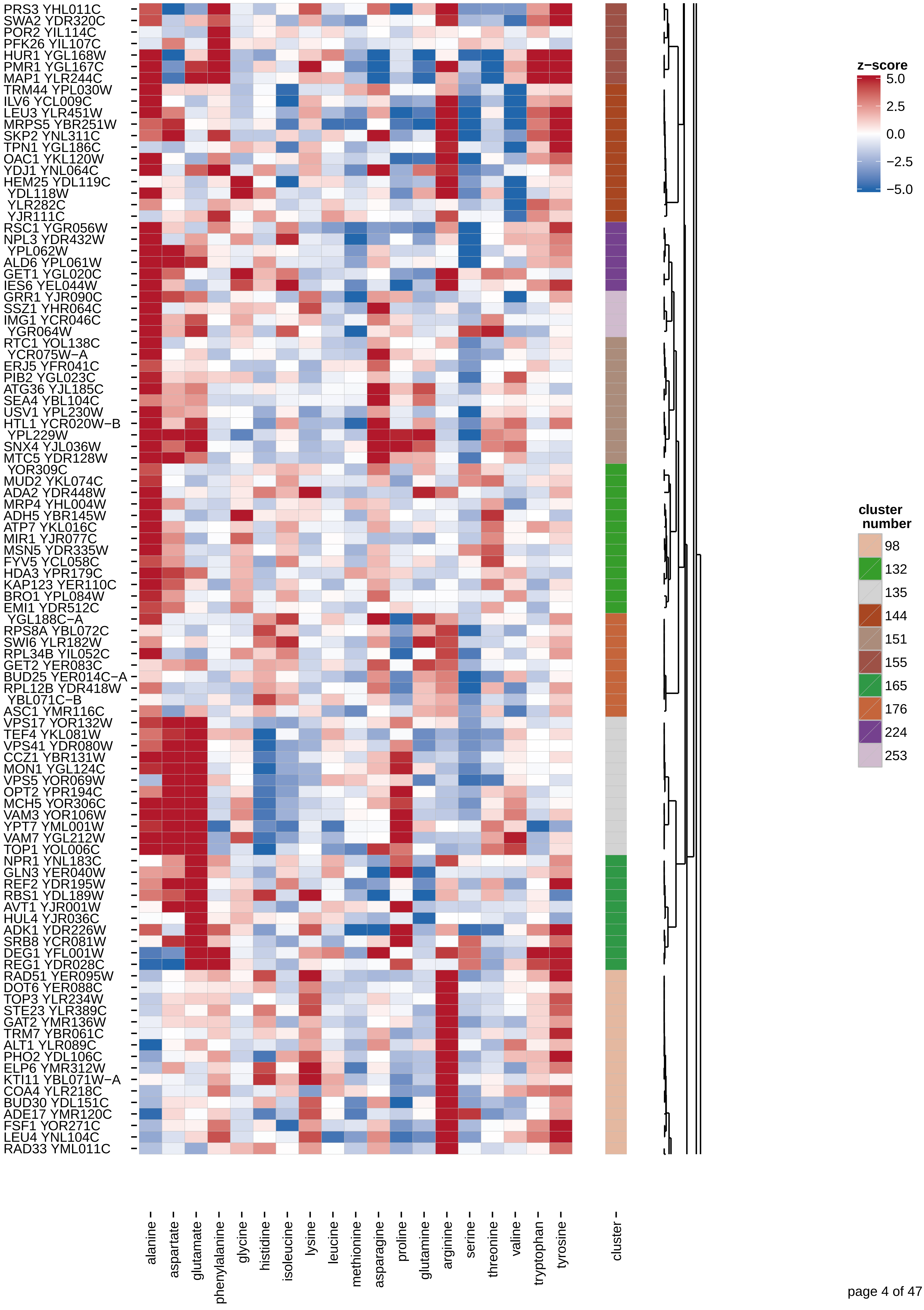

PRS3 YHL011C
SWA2 YDR320C
POR2 YIL114C
PFK26 YIL107C
HUR1 YGL168W
PMR1 YGL167C
MAP1 YLR244C
TRM44 YPL030W
ILV6 YCL009C
LEU3 YLR451W
MRPS5 YBR251W
SKP2 YNL311C
TPN1 YGL186C
OAC1 YKL120W
YDJ1 YNL064C
HEM25 YDL119C
YDL118W
YLR282C
YJR111C
RSC1 YGR056W
NPL3 YDR432W
YPL062W
ALD6 YPL061W
GET1 YGL020C
IES6 YEL044W
GRR1 YJR090C
SSZ1 YHR064C
IMG1 YCR046C
YGR064W
RTC1 YOL138C
YCR075W−A
ERJ5 YFR041C
PIB2 YGL023C
ATG36 YJL185C
SEA4 YBL104C
USV1 YPL230W
HTL1 YCR020W−B
YPL229W
SNX4 YJL036W
MTC5 YDR128W
YOR309C
MUD2 YKL074C
ADA2 YDR448W
MRP4 YHL004W
ADH5 YBR145W
ATP7 YKL016C
MIR1 YJR077C
MSN5 YDR335W
FYV5 YCL058C
HDA3 YPR179C
KAP123 YER110C
BRO1 YPL084W
EMI1 YDR512C
YGL188C−A
RPS8A YBL072C
SWI6 YLR182W
RPL34B YIL052C
GET2 YER083C
BUD25 YER014C−A
RPL12B YDR418W
YBL071C−B
ASC1 YMR116C
VPS17 YOR132W
TEF4 YKL081W
VPS41 YDR080W
CCZ1 YBR131W
MON1 YGL124C
VPS5 YOR069W
OPT2 YPR194C
MCH5 YOR306C
VAM3 YOR106W
YPT7 YML001W
VAM7 YGL212W
TOP1 YOL006C
NPR1 YNL183C
GLN3 YER040W
REF2 YDR195W
RBS1 YDL189W
AVT1 YJR001W
HUL4 YJR036C
ADK1 YDR226W
SRB8 YCR081W
DEG1 YFL001W
REG1 YDR028C
RAD51 YER095W
DOT6 YER088C
TOP3 YLR234W
STE23 YLR389C
GAT2 YMR136W
TRM7 YBR061C
ALT1 YLR089C
PHO2 YDL106C
ELP6 YMR312W
KTI11 YBL071W−A
COA4 YLR218C
BUD30 YDL151C
ADE17 YMR120C
FSF1 YOR271C
LEU4 YNL104C
RAD33 YML011C
alanine
aspartate
glutamate
phenylalanine
glycine
histidine
isoleucine
lysine
leucine
methionine
asparagine
proline
glutamine
arginine
serine
threonine
valine
tryptophan
tyrosine
cluster
z−score
5.0
2.5
0.0
−2.5
−5.0
cluster number
98
132
135
144
151
155
165
176
224
253

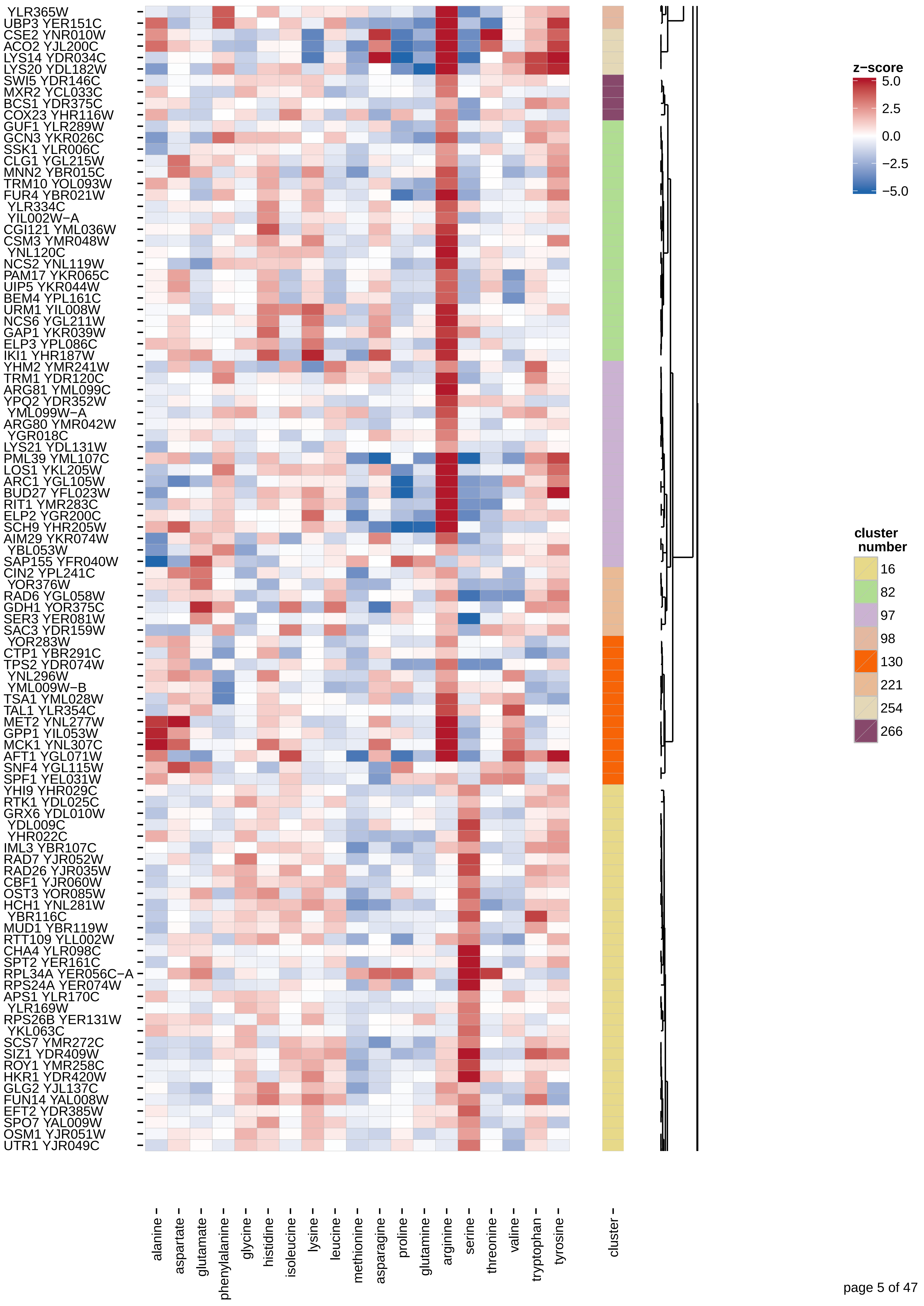
YLR365W
UBP3 YER151C
CSE2 YNR010W
ACO2 YJL200C
LYS14 YDR034C
LYS20 YDL182W
SWI5 YDR146C
MXR2 YCL033C
BCS1 YDR375C
COX23 YHR116W
GUF1 YLR289W
GCN3 YKR026C
SSK1 YLR006C
CLG1 YGL215W
MNN2 YBR015C
TRM10 YOL093W
FUR4 YBR021W
YLR334C
YIL002W−A
CGI121 YML036W
CSM3 YMR048W
YNL120C
NCS2 YNL119W
PAM17 YKR065C
UIP5 YKR044W
BEM4 YPL161C
URM1 YIL008W
NCS6 YGL211W
GAP1 YKR039W
ELP3 YPL086C
IKI1 YHR187W
YHM2 YMR241W
TRM1 YDR120C
ARG81 YML099C
YPQ2 YDR352W
YML099W−A
ARG80 YMR042W
YGR018C
LYS21 YDL131W
PML39 YML107C
LOS1 YKL205W
ARC1 YGL105W
BUD27 YFL023W
RIT1 YMR283C
ELP2 YGR200C
SCH9 YHR205W
AIM29 YKR074W
YBL053W
SAP155 YFR040W
CIN2 YPL241C
YOR376W
RAD6 YGL058W
GDH1 YOR375C
SER3 YER081W
SAC3 YDR159W
YOR283W
CTP1 YBR291C
TPS2 YDR074W
YNL296W
YML009W−B
TSA1 YML028W
TAL1 YLR354C
MET2 YNL277W
GPP1 YIL053W
MCK1 YNL307C
AFT1 YGL071W
SNF4 YGL115W
SPF1 YEL031W
YHI9 YHR029C
RTK1 YDL025C
GRX6 YDL010W
YDL009C
YHR022C
IML3 YBR107C
RAD7 YJR052W
RAD26 YJR035W
CBF1 YJR060W
OST3 YOR085W
HCH1 YNL281W
YBR116C
MUD1 YBR119W
RTT109 YLL002W
CHA4 YLR098C
SPT2 YER161C
RPL34A YER056C−A
RPS24A YER074W
APS1 YLR170C
YLR169W
RPS26B YER131W
YKL063C
SCS7 YMR272C
SIZ1 YDR409W
ROY1 YMR258C
HKR1 YDR420W
GLG2 YJL137C
FUN14 YAL008W
EFT2 YDR385W
SPO7 YAL009W
OSM1 YJR051W
UTR1 YJR049C
alanine
aspartate
glutamate
phenylalanine
glycine
histidine
isoleucine
lysine
leucine
methionine
asparagine
proline
glutamine
arginine
serine
threonine
valine
tryptophan
tyrosine
cluster
z−score
5.0
2.5
0.0
−2.5
−5.0
cluster
number
16
82
97
98
130
221
254
266

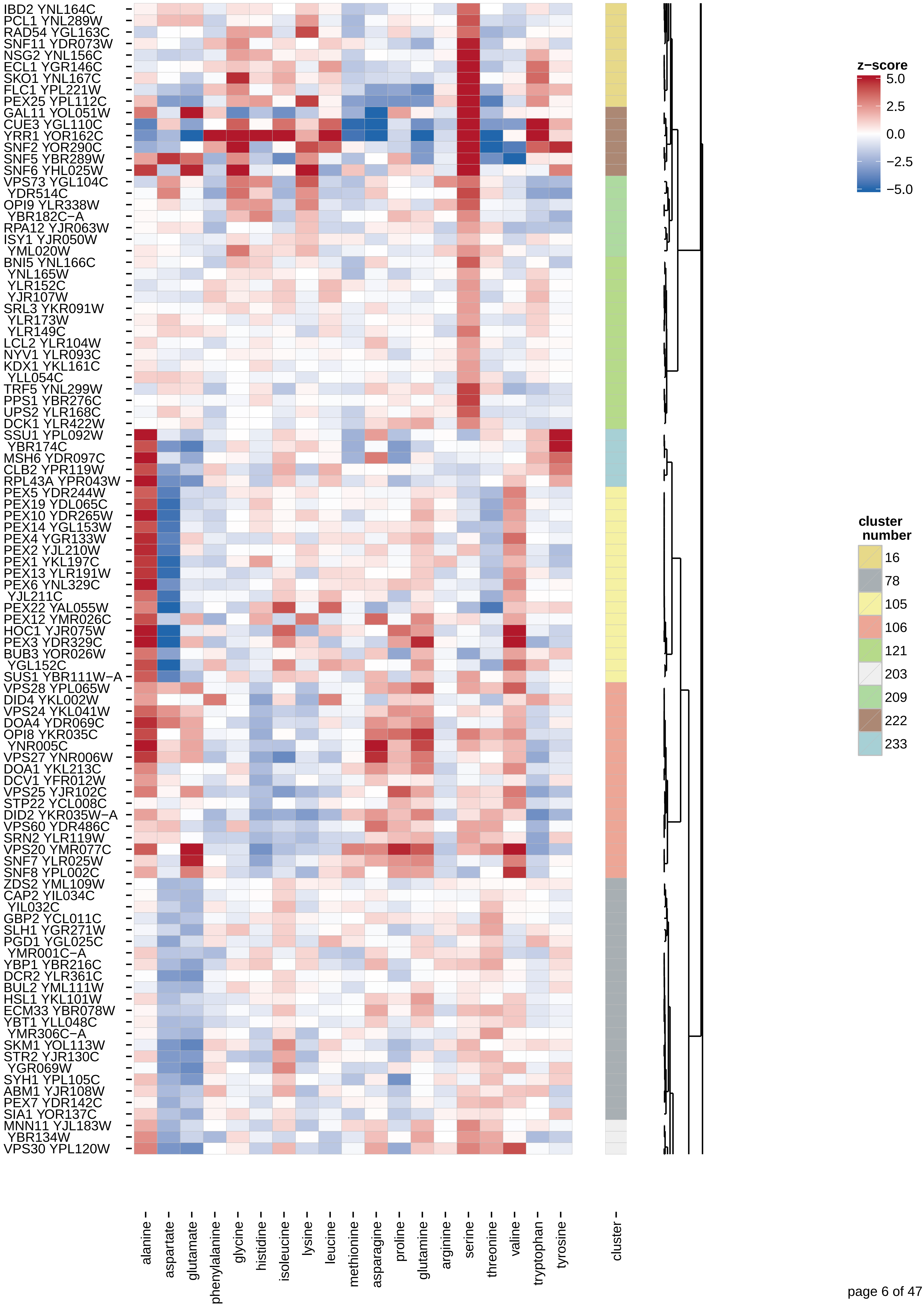
IBD2 YNL164C
PCL1 YNL289W
RAD54 YGL163C
SNF11 YDR073W
NSG2 YNL156C
ECL1 YGR146C
SKO1 YNL167C
FLC1 YPL221W
PEX25 YPL112C
GAL11 YOL051W
CUE3 YGL110C
YRR1 YOR162C
SNF2 YOR290C
SNF5 YBR289W
SNF6 YHL025W
VPS73 YGL104C
YDR514C
OPI9 YLR338W
YBR182C−A
RPA12 YJR063W
ISY1 YJR050W
YML020W
BNI5 YNL166C
YNL165W
YLR152C
YJR107W
SRL3 YKR091W
YLR173W
YLR149C
LCL2 YLR104W
NYV1 YLR093C
KDX1 YKL161C
YLL054C
TRF5 YNL299W
PPS1 YBR276C
UPS2 YLR168C
DCK1 YLR422W
SSU1 YPL092W
YBR174C
MSH6 YDR097C
CLB2 YPR119W
RPL43A YPR043W
PEX5 YDR244W
PEX19 YDL065C
PEX10 YDR265W
PEX14 YGL153W
PEX4 YGR133W
PEX2 YJL210W
PEX1 YKL197C
PEX13 YLR191W
PEX6 YNL329C
YJL211C
PEX22 YAL055W
PEX12 YMR026C
HOC1 YJR075W
PEX3 YDR329C
BUB3 YOR026W
YGL152C
SUS1 YBR111W−A
VPS28 YPL065W
DID4 YKL002W
VPS24 YKL041W
DOA4 YDR069C
OPI8 YKR035C
YNR005C
VPS27 YNR006W
DOA1 YKL213C
DCV1 YFR012W
VPS25 YJR102C
STP22 YCL008C
DID2 YKR035W−A
VPS60 YDR486C
SRN2 YLR119W
VPS20 YMR077C
SNF7 YLR025W
SNF8 YPL002C
ZDS2 YML109W
CAP2 YIL034C
YIL032C
GBP2 YCL011C
SLH1 YGR271W
PGD1 YGL025C
YMR001C−A
YBP1 YBR216C
DCR2 YLR361C
BUL2 YML111W
HSL1 YKL101W
ECM33 YBR078W
YBT1 YLL048C
YMR306C−A
SKM1 YOL113W
STR2 YJR130C
YGR069W
SYH1 YPL105C
ABM1 YJR108W
PEX7 YDR142C
SIA1 YOR137C
MNN11 YJL183W
YBR134W
VPS30 YPL120W
alanine
aspartate
glutamate
phenylalanine
glycine
histidine
isoleucine
lysine
leucine
methionine
asparagine
proline
glutamine
arginine
serine
threonine
valine
tryptophan
tyrosine
cluster
z−score
5.0
2.5
0.0
−2.5
−5.0
cluster number
16
78
105
106
121
203
209
222
233

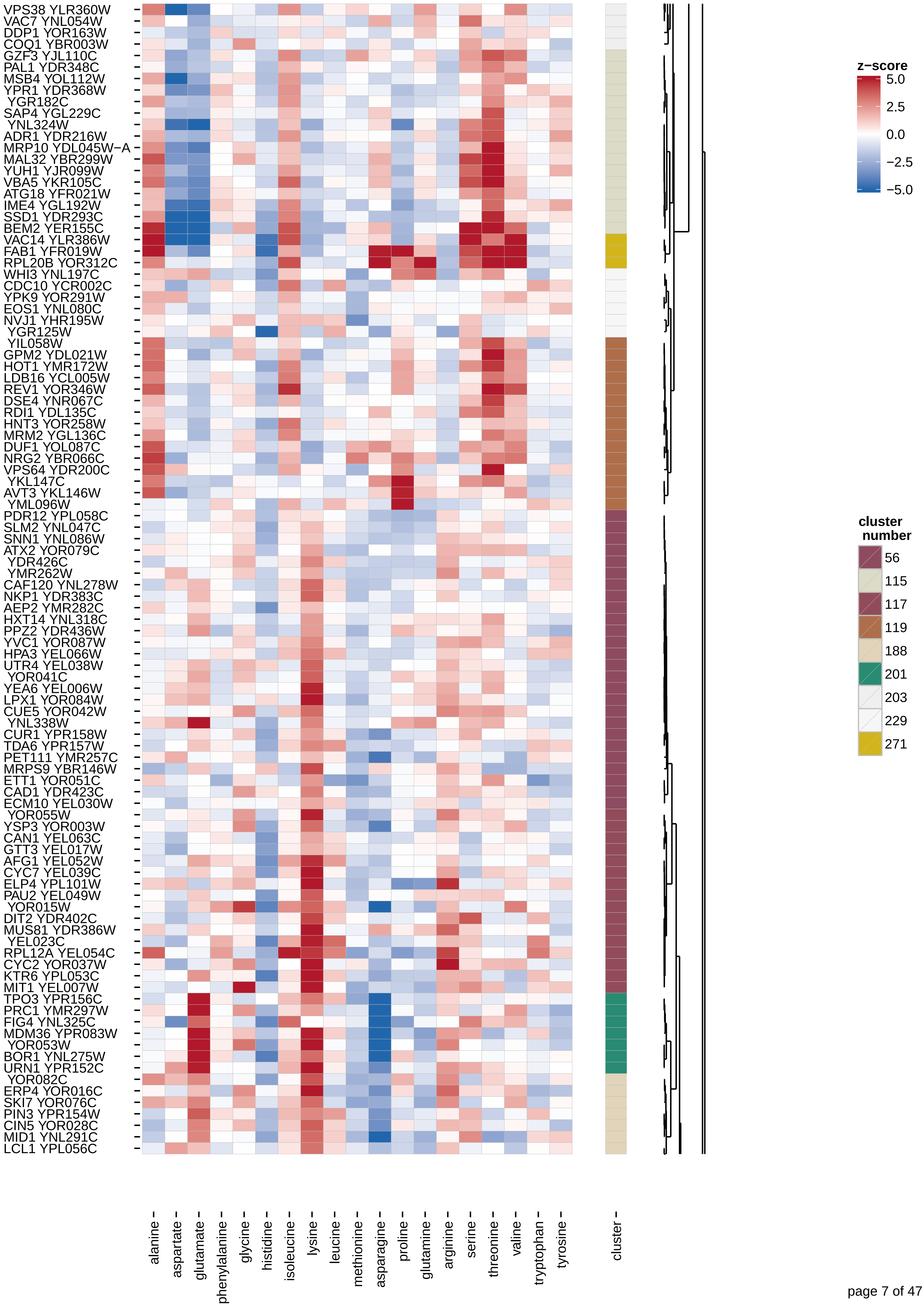
VPS38 YLR360W
VAC7 YNL054W
DDP1 YOR163W
COQ1 YBR003W
GZF3 YJL110C
PAL1 YDR348C
MSB4 YOL112W
YPR1 YDR368W
YGR182C
SAP4 YGL229C
YNL324W
ADR1 YDR216W
MRP10 YDL045W−A
MAL32 YBR299W
YUH1 YJR099W
VBA5 YKR105C
ATG18 YFR021W
IME4 YGL192W
SSD1 YDR293C
BEM2 YER155C
VAC14 YLR386W
FAB1 YFR019W
RPL20B YOR312C
WHI3 YNL197C
CDC10 YCR002C
YPK9 YOR291W
EOS1 YNL080C
NVJ1 YHR195W
YGR125W
YIL058W
GPM2 YDL021W
HOT1 YMR172W
LDB16 YCL005W
REV1 YOR346W
DSE4 YNR067C
RDI1 YDL135C
HNT3 YOR258W
MRM2 YGL136C
DUF1 YOL087C
NRG2 YBR066C
VPS64 YDR200C
YKL147C
AVT3 YKL146W
YML096W
PDR12 YPL058C
SLM2 YNL047C
SNN1 YNL086W
ATX2 YOR079C
YDR426C
YMR262W
CAF120 YNL278W
NKP1 YDR383C
AEP2 YMR282C
HXT14 YNL318C
PPZ2 YDR436W
YVC1 YOR087W
HPA3 YEL066W
UTR4 YEL038W
YOR041C
YEA6 YEL006W
LPX1 YOR084W
CUE5 YOR042W
YNL338W
CUR1 YPR158W
TDA6 YPR157W
PET111 YMR257C
MRPS9 YBR146W
ETT1 YOR051C
CAD1 YDR423C
ECM10 YEL030W
YOR055W
YSP3 YOR003W
CAN1 YEL063C
GTT3 YEL017W
AFG1 YEL052W
CYC7 YEL039C
ELP4 YPL101W
PAU2 YEL049W
YOR015W
DIT2 YDR402C
MUS81 YDR386W
YEL023C
RPL12A YEL054C
CYC2 YOR037W
KTR6 YPL053C
MIT1 YEL007W
TPO3 YPR156C
PRC1 YMR297W
FIG4 YNL325C
MDM36 YPR083W
YOR053W
BOR1 YNL275W
URN1 YPR152C
YOR082C
ERP4 YOR016C
SKI7 YOR076C
PIN3 YPR154W
CIN5 YOR028C
MID1 YNL291C
LCL1 YPL056C
alanine
aspartate
glutamate
phenylalanine
glycine
histidine
isoleucine
lysine
leucine
methionine
asparagine
proline
glutamine
arginine
serine
threonine
valine
tryptophan
tyrosine
cluster
z−score
5.0
2.5
0.0
−2.5
−5.0
cluster number
56
115
117
119
188
201
203
229
271

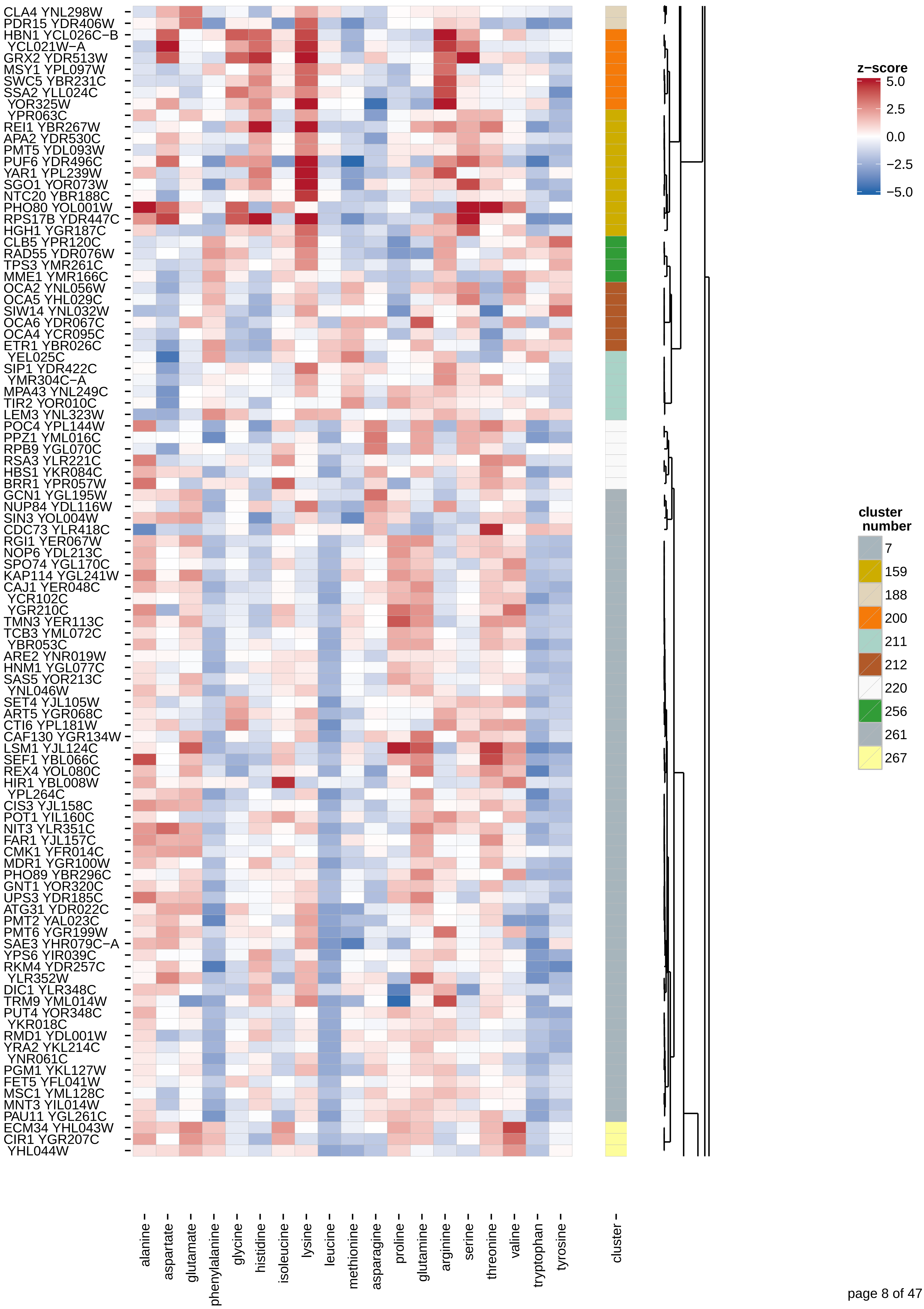
CLA4 YNL298W
PDR15 YDR406W
HBN1 YCL026C−B
YCL021W−A
GRX2 YDR513W
MSY1 YPL097W
SWC5 YBR231C
SSA2 YLL024C
YOR325W
YPR063C
REI1 YBR267W
APA2 YDR530C
PMT5 YDL093W
PUF6 YDR496C
YAR1 YPL239W
SGO1 YOR073W
NTC20 YBR188C
PHO80 YOL001W
RPS17B YDR447C
HGH1 YGR187C
CLB5 YPR120C
RAD55 YDR076W
TPS3 YMR261C
MME1 YMR166C
OCA2 YNL056W
OCA5 YHL029C
SIW14 YNL032W
OCA6 YDR067C
OCA4 YCR095C
ETR1 YBR026C
YEL025C
SIP1 YDR422C
YMR304C−A
MPA43 YNL249C
TIR2 YOR010C
LEM3 YNL323W
POC4 YPL144W
PPZ1 YML016C
RPB9 YGL070C
RSA3 YLR221C
HBS1 YKR084C
BRR1 YPR057W
GCN1 YGL195W
NUP84 YDL116W
SIN3 YOL004W
CDC73 YLR418C
RGI1 YER067W
NOP6 YDL213C
SPO74 YGL170C
KAP114 YGL241W
CAJ1 YER048C
YCR102C
YGR210C
TMN3 YER113C
TCB3 YML072C
YBR053C
ARE2 YNR019W
HNM1 YGL077C
SAS5 YOR213C
YNL046W
SET4 YJL105W
ART5 YGR068C
CTI6 YPL181W
CAF130 YGR134W
LSM1 YJL124C
SEF1 YBL066C
REX4 YOL080C
HIR1 YBL008W
YPL264C
CIS3 YJL158C
POT1 YIL160C
NIT3 YLR351C
FAR1 YJL157C
CMK1 YFR014C
MDR1 YGR100W
PHO89 YBR296C
GNT1 YOR320C
UPS3 YDR185C
ATG31 YDR022C
PMT2 YAL023C
PMT6 YGR199W
SAE3 YHR079C−A
YPS6 YIR039C
RKM4 YDR257C
YLR352W
DIC1 YLR348C
TRM9 YML014W
PUT4 YOR348C
YKR018C
RMD1 YDL001W
YRA2 YKL214C
YNR061C
PGM1 YKL127W
FET5 YFL041W
MSC1 YML128C
MNT3 YIL014W
PAU11 YGL261C
ECM34 YHL043W
CIR1 YGR207C
YHL044W
alanine
aspartate
glutamate
phenylalanine
glycine
histidine
isoleucine
lysine
leucine
methionine
asparagine
proline
glutamine
arginine
serine
threonine
valine
tryptophan
tyrosine
cluster
z−score
5.0
2.5
0.0
−2.5
−5.0
cluster number
7
159
188
200
211
212
220
256
261
267

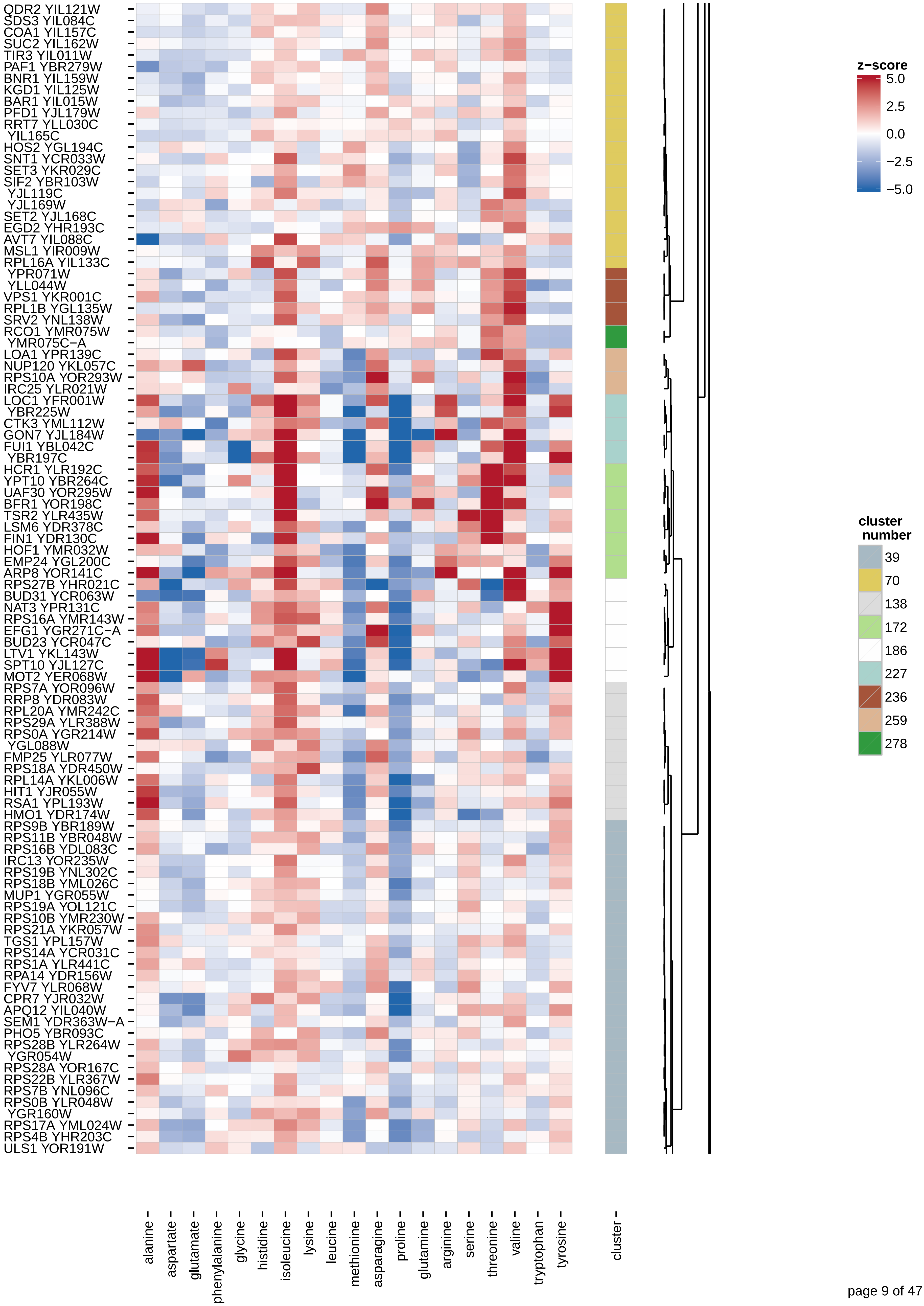
QDR2 YIL121W
SDS3 YIL084C
COA1 YIL157C
SUC2 YIL162W
TIR3 YIL011W
PAF1 YBR279W
BNR1 YIL159W
KGD1 YIL125W
BAR1 YIL015W
PFD1 YJL179W
RRT7 YLL030C
YIL165C
HOS2 YGL194C
SNT1 YCR033W
SET3 YKR029C
SIF2 YBR103W
YJL119C
YJL169W
SET2 YJL168C
EGD2 YHR193C
AVT7 YIL088C
MSL1 YIR009W
RPL16A YIL133C
YPR071W
YLL044W
VPS1 YKR001C
RPL1B YGL135W
SRV2 YNL138W
RCO1 YMR075W
YMR075C−A
LOA1 YPR139C
NUP120 YKL057C
RPS10A YOR293W
IRC25 YLR021W
LOC1 YFR001W
YBR225W
CTK3 YML112W
GON7 YJL184W
FUI1 YBL042C
YBR197C
HCR1 YLR192C
YPT10 YBR264C
UAF30 YOR295W
BFR1 YOR198C
TSR2 YLR435W
LSM6 YDR378C
FIN1 YDR130C
HOF1 YMR032W
EMP24 YGL200C
ARP8 YOR141C
RPS27B YHR021C
BUD31 YCR063W
NAT3 YPR131C
RPS16A YMR143W
EFG1 YGR271C−A
BUD23 YCR047C
LTV1 YKL143W
SPT10 YJL127C
MOT2 YER068W
RPS7A YOR096W
RRP8 YDR083W
RPL20A YMR242C
RPS29A YLR388W
RPS0A YGR214W
YGL088W
FMP25 YLR077W
RPS18A YDR450W
RPL14A YKL006W
HIT1 YJR055W
RSA1 YPL193W
HMO1 YDR174W
RPS9B YBR189W
RPS11B YBR048W
RPS16B YDL083C
IRC13 YOR235W
RPS19B YNL302C
RPS18B YML026C
MUP1 YGR055W
RPS19A YOL121C
RPS10B YMR230W
RPS21A YKR057W
TGS1 YPL157W
RPS14A YCR031C
RPS1A YLR441C
RPA14 YDR156W
FYV7 YLR068W
CPR7 YJR032W
APQ12 YIL040W
SEM1 YDR363W−A
PHO5 YBR093C
RPS28B YLR264W
YGR054W
RPS28A YOR167C
RPS22B YLR367W
RPS7B YNL096C
RPS0B YLR048W
YGR160W
RPS17A YML024W
RPS4B YHR203C
ULS1 YOR191W
alanine
aspartate
glutamate
phenylalanine
glycine
histidine
isoleucine
lysine
leucine
methionine
asparagine
proline
glutamine
arginine
serine
threonine
valine
tryptophan
tyrosine
cluster
z−score
5.0
2.5
0.0
−2.5
−5.0
cluster number
39
70
138
172
186
227
236
259
278

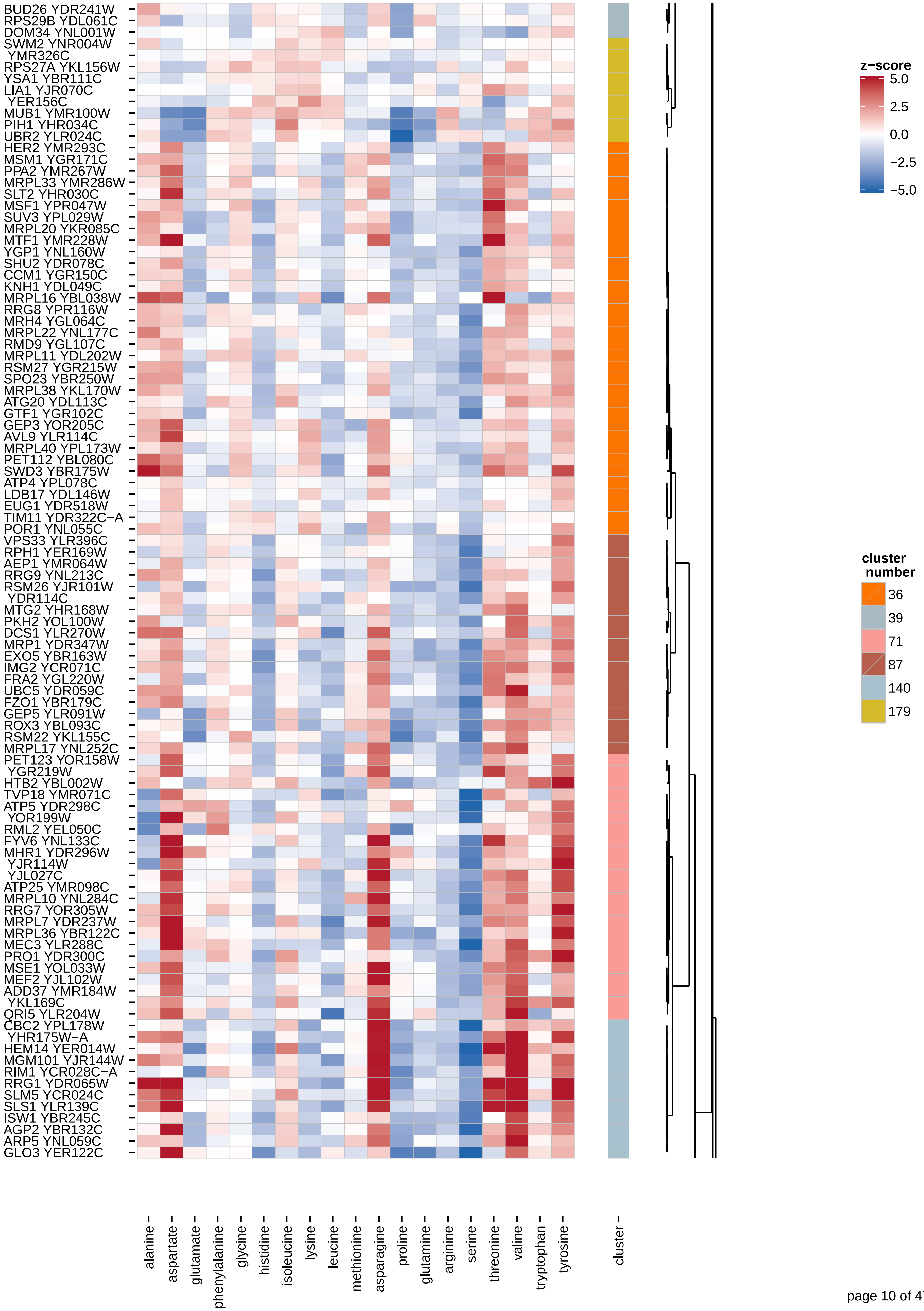
BUD26 YDR241W
RPS29B YDL061C
DOM34 YNL001W
SWM2 YNR004W
YMR326C
RPS27A YKL156W
YSA1 YBR111C
LIA1 YJR070C
YER156C
MUB1 YMR100W
PIH1 YHR034C
UBR2 YLR024C
HER2 YMR293C
MSM1 YGR171C
PPA2 YMR267W
MRPL33 YMR286W
SLT2 YHR030C
MSF1 YPR047W
SUV3 YPL029W
MRPL20 YKR085C
MTF1 YMR228W
YGP1 YNL160W
SHU2 YDR078C
CCM1 YGR150C
KNH1 YDL049C
MRPL16 YBL038W
RRG8 YPR116W
MRH4 YGL064C
MRPL22 YNL177C
RMD9 YGL107C
MRPL11 YDL202W
RSM27 YGR215W
SPO23 YBR250W
MRPL38 YKL170W
ATG20 YDL113C
GTF1 YGR102C
GEP3 YOR205C
AVL9 YLR114C
MRPL40 YPL173W
PET112 YBL080C
SWD3 YBR175W
ATP4 YPL078C
LDB17 YDL146W
EUG1 YDR518W
TIM11 YDR322C−A
POR1 YNL055C
VPS33 YLR396C
RPH1 YER169W
AEP1 YMR064W
RRG9 YNL213C
RSM26 YJR101W
YDR114C
MTG2 YHR168W
PKH2 YOL100W
DCS1 YLR270W
MRP1 YDR347W
EXO5 YBR163W
IMG2 YCR071C
FRA2 YGL220W
UBC5 YDR059C
FZO1 YBR179C
GEP5 YLR091W
ROX3 YBL093C
RSM22 YKL155C
MRPL17 YNL252C
PET123 YOR158W
YGR219W
HTB2 YBL002W
TVP18 YMR071C
ATP5 YDR298C
YOR199W
RML2 YEL050C
FYV6 YNL133C
MHR1 YDR296W
YJR114W
YJL027C
ATP25 YMR098C
MRPL10 YNL284C
RRG7 YOR305W
MRPL7 YDR237W
MRPL36 YBR122C
MEC3 YLR288C
PRO1 YDR300C
MSE1 YOL033W
MEF2 YJL102W
ADD37 YMR184W
YKL169C
QRI5 YLR204W
CBC2 YPL178W
YHR175W−A
HEM14 YER014W
MGM101 YJR144W
RIM1 YCR028C−A
RRG1 YDR065W
SLM5 YCR024C
SLS1 YLR139C
ISW1 YBR245C
AGP2 YBR132C
ARP5 YNL059C
GLO3 YER122C
alanine
aspartate
glutamate
phenylalanine
glycine
histidine
isoleucine
lysine
leucine
methionine
asparagine
proline
glutamine
arginine
serine
threonine
valine
tryptophan
tyrosine
cluster
z−score
5.0
2.5
0.0
−2.5
−5.0
cluster number
36
39
71
87
140
179

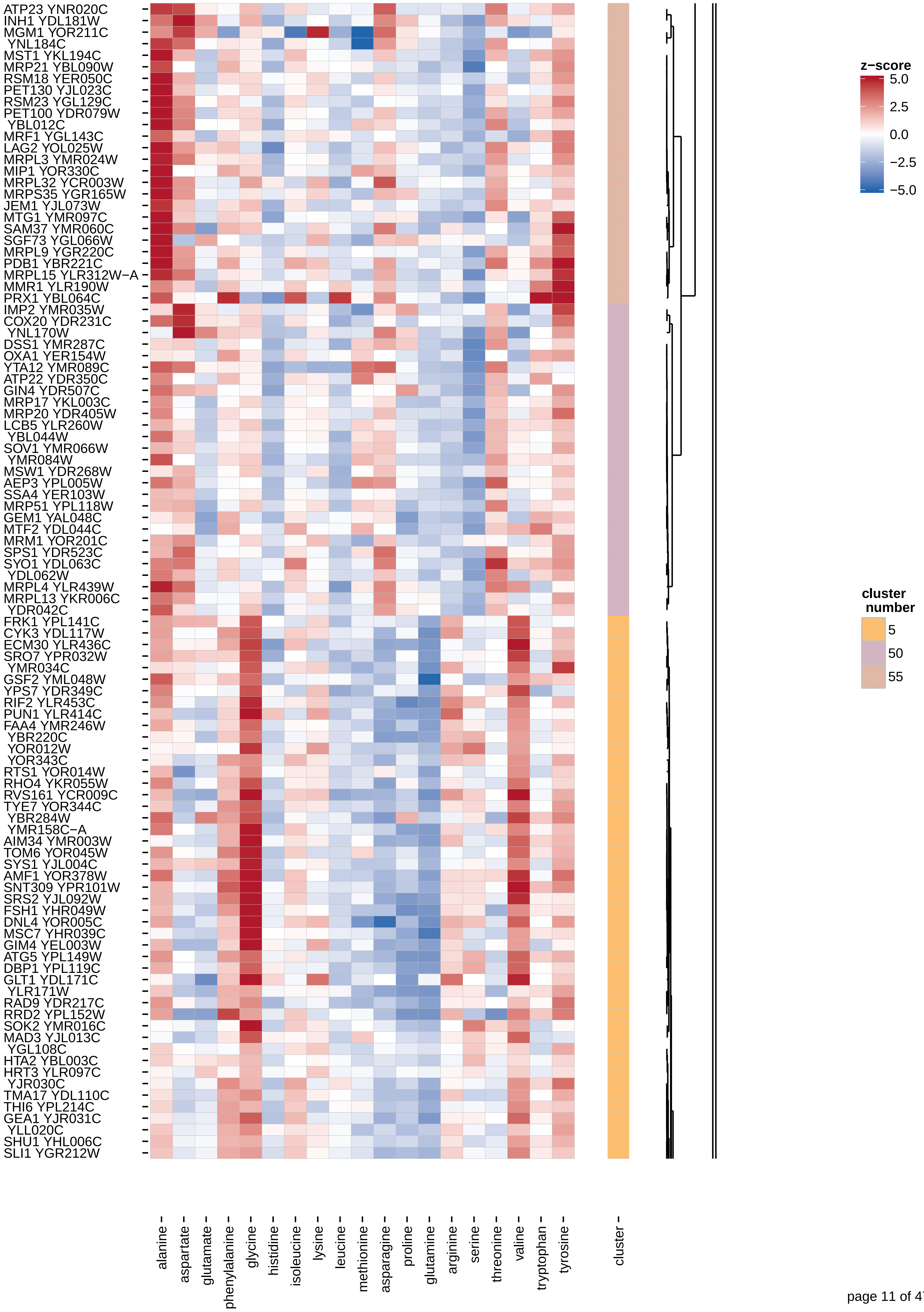
ATP23 YNR020C
INH1 YDL181W
MGM1 YOR211C
YNL184C
MST1 YKL194C
MRP21 YBL090W
RSM18 YER050C
PET130 YJL023C
RSM23 YGL129C
PET100 YDR079W
YBL012C
MRF1 YGL143C
LAG2 YOL025W
MRPL3 YMR024W
MIP1 YOR330C
MRPL32 YCR003W
MRPS35 YGR165W
JEM1 YJL073W
MTG1 YMR097C
SAM37 YMR060C
SGF73 YGL066W
MRPL9 YGR220C
PDB1 YBR221C
MRPL15 YLR312W−A
MMR1 YLR190W
PRX1 YBL064C
IMP2 YMR035W
COX20 YDR231C
YNL170W
DSS1 YMR287C
OXA1 YER154W
YTA12 YMR089C
ATP22 YDR350C
GIN4 YDR507C
MRP17 YKL003C
MRP20 YDR405W
LCB5 YLR260W
YBL044W
SOV1 YMR066W
YMR084W
MSW1 YDR268W
AEP3 YPL005W
SSA4 YER103W
MRP51 YPL118W
GEM1 YAL048C
MTF2 YDL044C
MRM1 YOR201C
SPS1 YDR523C
SYO1 YDL063C
YDL062W
MRPL4 YLR439W
MRPL13 YKR006C
YDR042C
FRK1 YPL141C
CYK3 YDL117W
ECM30 YLR436C
SRO7 YPR032W
YMR034C
GSF2 YML048W
YPS7 YDR349C
RIF2 YLR453C
PUN1 YLR414C
FAA4 YMR246W
YBR220C
YOR012W
YOR343C
RTS1 YOR014W
RHO4 YKR055W
RVS161 YCR009C
TYE7 YOR344C
YBR284W
YMR158C−A
AIM34 YMR003W
TOM6 YOR045W
SYS1 YJL004C
AMF1 YOR378W
SNT309 YPR101W
SRS2 YJL092W
FSH1 YHR049W
DNL4 YOR005C
MSC7 YHR039C
GIM4 YEL003W
ATG5 YPL149W
DBP1 YPL119C
GLT1 YDL171C
YLR171W
RAD9 YDR217C
RRD2 YPL152W
SOK2 YMR016C
MAD3 YJL013C
YGL108C
HTA2 YBL003C
HRT3 YLR097C
YJR030C
TMA17 YDL110C
THI6 YPL214C
GEA1 YJR031C
YLL020C
SHU1 YHL006C
SLI1 YGR212W
alanine
aspartate
glutamate
phenylalanine
glycine
histidine
isoleucine
lysine
leucine
methionine
asparagine
proline
glutamine
arginine
serine
threonine
valine
tryptophan
tyrosine
cluster
z−score
5.0
2.5
0.0
−2.5
−5.0
cluster
number
5
50
55

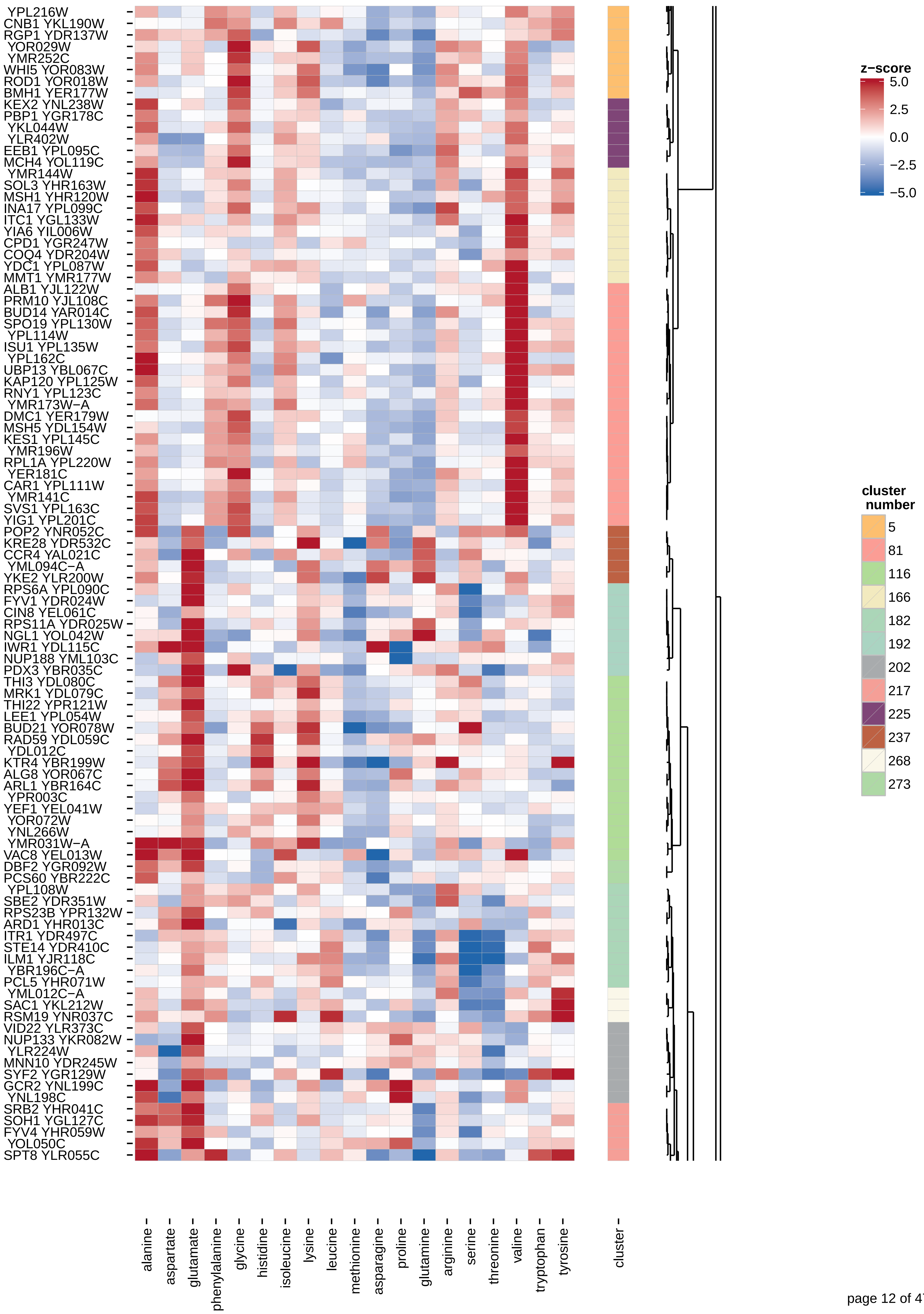

YPL216W
CNB1 YKL190W
RGP1 YDR137W
YOR029W
YMR252C
WHI5 YOR083W
ROD1 YOR018W
BMH1 YER177W
KEX2 YNL238W
PBP1 YGR178C
YKL044W
YLR402W
EEB1 YPL095C
MCH4 YOL119C
YMR144W
SOL3 YHR163W
MSH1 YHR120W
INA17 YPL099C
ITC1 YGL133W
YIA6 YIL006W
CPD1 YGR247W
COQ4 YDR204W
YDC1 YPL087W
MMT1 YMR177W
ALB1 YJL122W
PRM10 YJL108C
BUD14 YAR014C
SPO19 YPL130W
YPL114W
ISU1 YPL135W
YPL162C
UBP13 YBL067C
KAP120 YPL125W
RNY1 YPL123C
YMR173W−A
DMC1 YER179W
MSH5 YDL154W
KES1 YPL145C
YMR196W
RPL1A YPL220W
YER181C
CAR1 YPL111W
YMR141C
SVS1 YPL163C
YIG1 YPL201C
POP2 YNR052C
KRE28 YDR532C
CCR4 YAL021C
YML094C−A
YKE2 YLR200W
RPS6A YPL090C
FYV1 YDR024W
CIN8 YEL061C
RPS11A YDR025W
NGL1 YOL042W
IWR1 YDL115C
NUP188 YML103C
PDX3 YBR035C
THI3 YDL080C
MRK1 YDL079C
THI22 YPR121W
LEE1 YPL054W
BUD21 YOR078W
RAD59 YDL059C
YDL012C
KTR4 YBR199W
ALG8 YOR067C
ARL1 YBR164C
YPR003C
YEF1 YEL041W
YOR072W
YNL266W
YMR031W−A
VAC8 YEL013W
DBF2 YGR092W
PCS60 YBR222C
YPL108W
SBE2 YDR351W
RPS23B YPR132W
ARD1 YHR013C
ITR1 YDR497C
STE14 YDR410C
ILM1 YJR118C
YBR196C−A
PCL5 YHR071W
YML012C−A
SAC1 YKL212W
RSM19 YNR037C
VID22 YLR373C
NUP133 YKR082W
YLR224W
MNN10 YDR245W
SYF2 YGR129W
GCR2 YNL199C
YNL198C
SRB2 YHR041C
SOH1 YGL127C
FYV4 YHR059W
YOL050C
SPT8 YLR055C
alanine
aspartate
glutamate
phenylalanine
glycine
histidine
isoleucine
lysine
leucine
methionine
asparagine
proline
glutamine
arginine
serine
threonine
valine
tryptophan
tyrosine
cluster
z−score
5.0
2.5
0.0
−2.5
−5.0
cluster number
5
81
116
166
182
192
202
217
225
237
268
273

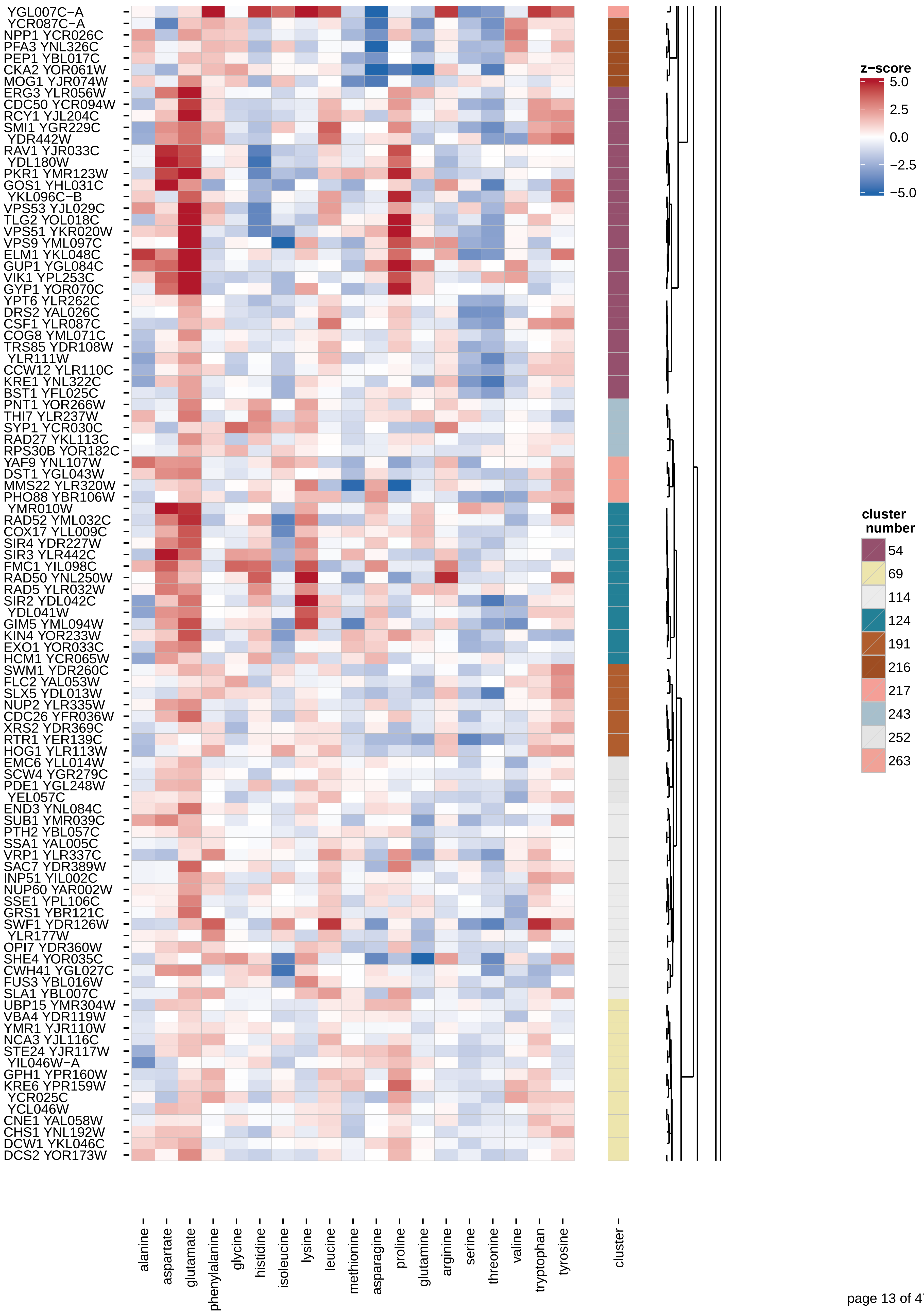
YGL007C−A
YCR087C−A
NPP1 YCR026C
PFA3 YNL326C
PEP1 YBL017C
CKA2 YOR061W
MOG1 YJR074W
ERG3 YLR056W
CDC50 YCR094W
RCY1 YJL204C
SMI1 YGR229C
YDR442W
RAV1 YJR033C
YDL180W
PKR1 YMR123W
GOS1 YHL031C
YKL096C−B
VPS53 YJL029C
TLG2 YOL018C
VPS51 YKR020W
VPS9 YML097C
ELM1 YKL048C
GUP1 YGL084C
VIK1 YPL253C
GYP1 YOR070C
YPT6 YLR262C
DRS2 YAL026C
CSF1 YLR087C
COG8 YML071C
TRS85 YDR108W
YLR111W
CCW12 YLR110C
KRE1 YNL322C
BST1 YFL025C
PNT1 YOR266W
THI7 YLR237W
SYP1 YCR030C
RAD27 YKL113C
RPS30B YOR182C
YAF9 YNL107W
DST1 YGL043W
MMS22 YLR320W
PHO88 YBR106W
YMR010W
RAD52 YML032C
COX17 YLL009C
SIR4 YDR227W
SIR3 YLR442C
FMC1 YIL098C
RAD50 YNL250W
RAD5 YLR032W
SIR2 YDL042C
YDL041W
GIM5 YML094W
KIN4 YOR233W
EXO1 YOR033C
HCM1 YCR065W
SWM1 YDR260C
FLC2 YAL053W
SLX5 YDL013W
NUP2 YLR335W
CDC26 YFR036W
XRS2 YDR369C
RTR1 YER139C
HOG1 YLR113W
EMC6 YLL014W
SCW4 YGR279C
PDE1 YGL248W
YEL057C
END3 YNL084C
SUB1 YMR039C
PTH2 YBL057C
SSA1 YAL005C
VRP1 YLR337C
SAC7 YDR389W
INP51 YIL002C
NUP60 YAR002W
SSE1 YPL106C
GRS1 YBR121C
SWF1 YDR126W
YLR177W
OPI7 YDR360W
SHE4 YOR035C
CWH41 YGL027C
FUS3 YBL016W
SLA1 YBL007C
UBP15 YMR304W
VBA4 YDR119W
YMR1 YJR110W
NCA3 YJL116C
STE24 YJR117W
YIL046W−A
GPH1 YPR160W
KRE6 YPR159W
YCR025C
YCL046W
CNE1 YAL058W
CHS1 YNL192W
DCW1 YKL046C
DCS2 YOR173W
alanine
aspartate
glutamate
phenylalanine
glycine
histidine
isoleucine
lysine
leucine
methionine
asparagine
proline
glutamine
arginine
serine
threonine
valine
tryptophan
tyrosine
cluster
z−score
5.0
2.5
0.0
−2.5
−5.0
cluster
number
54
69
114
124
191
216
217
243
252
263

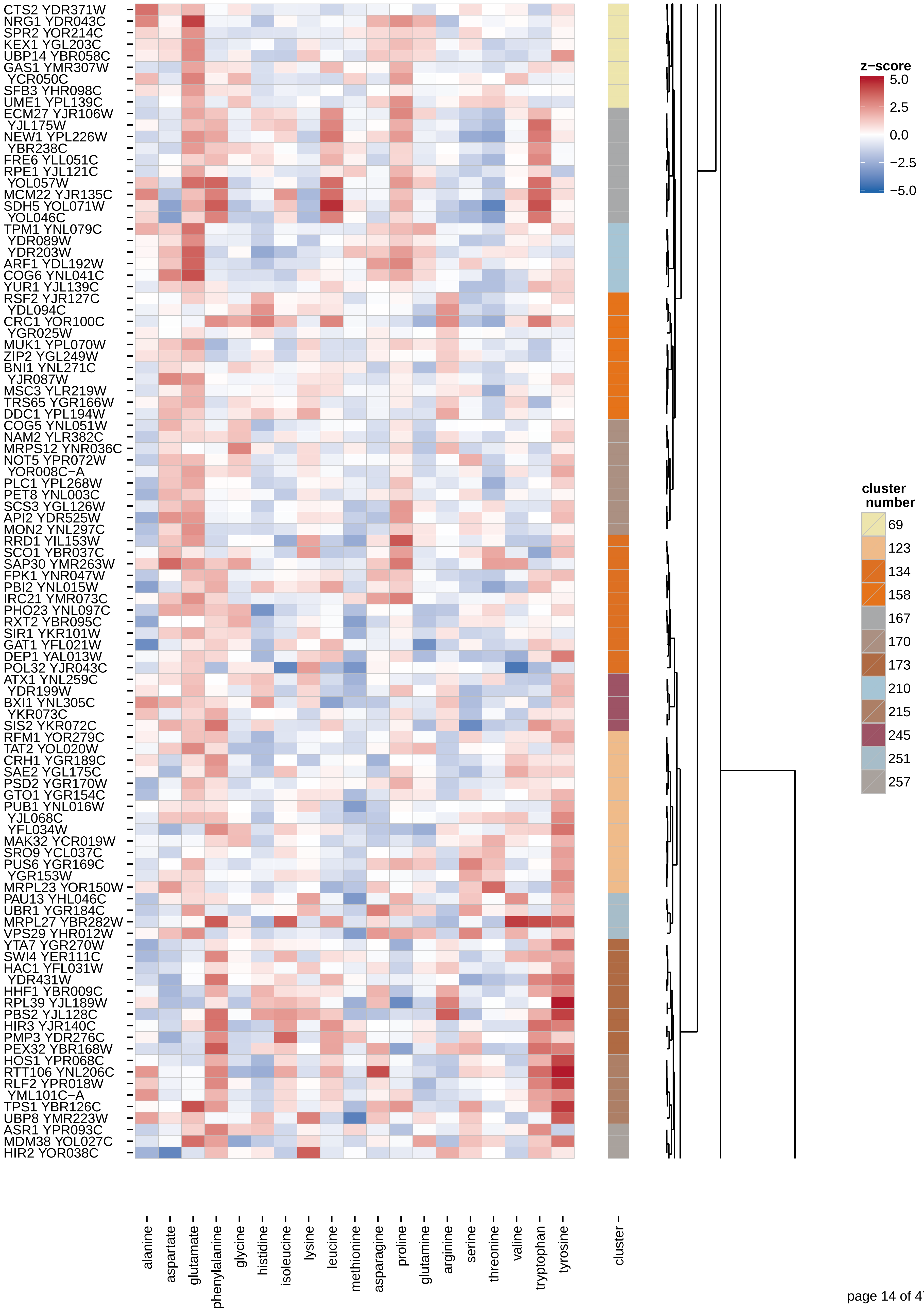
CTS2 YDR371W
NRG1 YDR043C
SPR2 YOR214C
KEX1 YGL203C
UBP14 YBR058C
GAS1 YMR307W
YCR050C
SFB3 YHR098C
UME1 YPL139C
ECM27 YJR106W
YJL175W
NEW1 YPL226W
YBR238C
FRE6 YLL051C
RPE1 YJL121C
YOL057W
MCM22 YJR135C
SDH5 YOL071W
YOL046C
TPM1 YNL079C
YDR089W
YDR203W
ARF1 YDL192W
COG6 YNL041C
YUR1 YJL139C
RSF2 YJR127C
YDL094C
CRC1 YOR100C
YGR025W
MUK1 YPL070W
ZIP2 YGL249W
BNI1 YNL271C
YJR087W
MSC3 YLR219W
TRS65 YGR166W
DDC1 YPL194W
COG5 YNL051W
NAM2 YLR382C
MRPS12 YNR036C
NOT5 YPR072W
YOR008C−A
PLC1 YPL268W
PET8 YNL003C
SCS3 YGL126W
API2 YDR525W
MON2 YNL297C
RRD1 YIL153W
SCO1 YBR037C
SAP30 YMR263W
FPK1 YNR047W
PBI2 YNL015W
IRC21 YMR073C
PHO23 YNL097C
RXT2 YBR095C
SIR1 YKR101W
GAT1 YFL021W
DEP1 YAL013W
POL32 YJR043C
ATX1 YNL259C
YDR199W
BXI1 YNL305C
YKR073C
SIS2 YKR072C
RFM1 YOR279C
TAT2 YOL020W
CRH1 YGR189C
SAE2 YGL175C
PSD2 YGR170W
GTO1 YGR154C
PUB1 YNL016W
YJL068C
YFL034W
MAK32 YCR019W
SRO9 YCL037C
PUS6 YGR169C
YGR153W
MRPL23 YOR150W
PAU13 YHL046C
UBR1 YGR184C
MRPL27 YBR282W
VPS29 YHR012W
YTA7 YGR270W
SWI4 YER111C
HAC1 YFL031W
YDR431W
HHF1 YBR009C
RPL39 YJL189W
PBS2 YJL128C
HIR3 YJR140C
PMP3 YDR276C
PEX32 YBR168W
HOS1 YPR068C
RTT106 YNL206C
RLF2 YPR018W
YML101C−A
TPS1 YBR126C
UBP8 YMR223W
ASR1 YPR093C
MDM38 YOL027C
HIR2 YOR038C
alanine
aspartate
glutamate
phenylalanine
glycine
histidine
isoleucine
lysine
leucine
methionine
asparagine
proline
glutamine
arginine
serine
threonine
valine
tryptophan
tyrosine
cluster
z−score
5.0
2.5
0.0
−2.5
−5.0
cluster
number
69
123
134
158
167
170
173
210
215
245
251
257

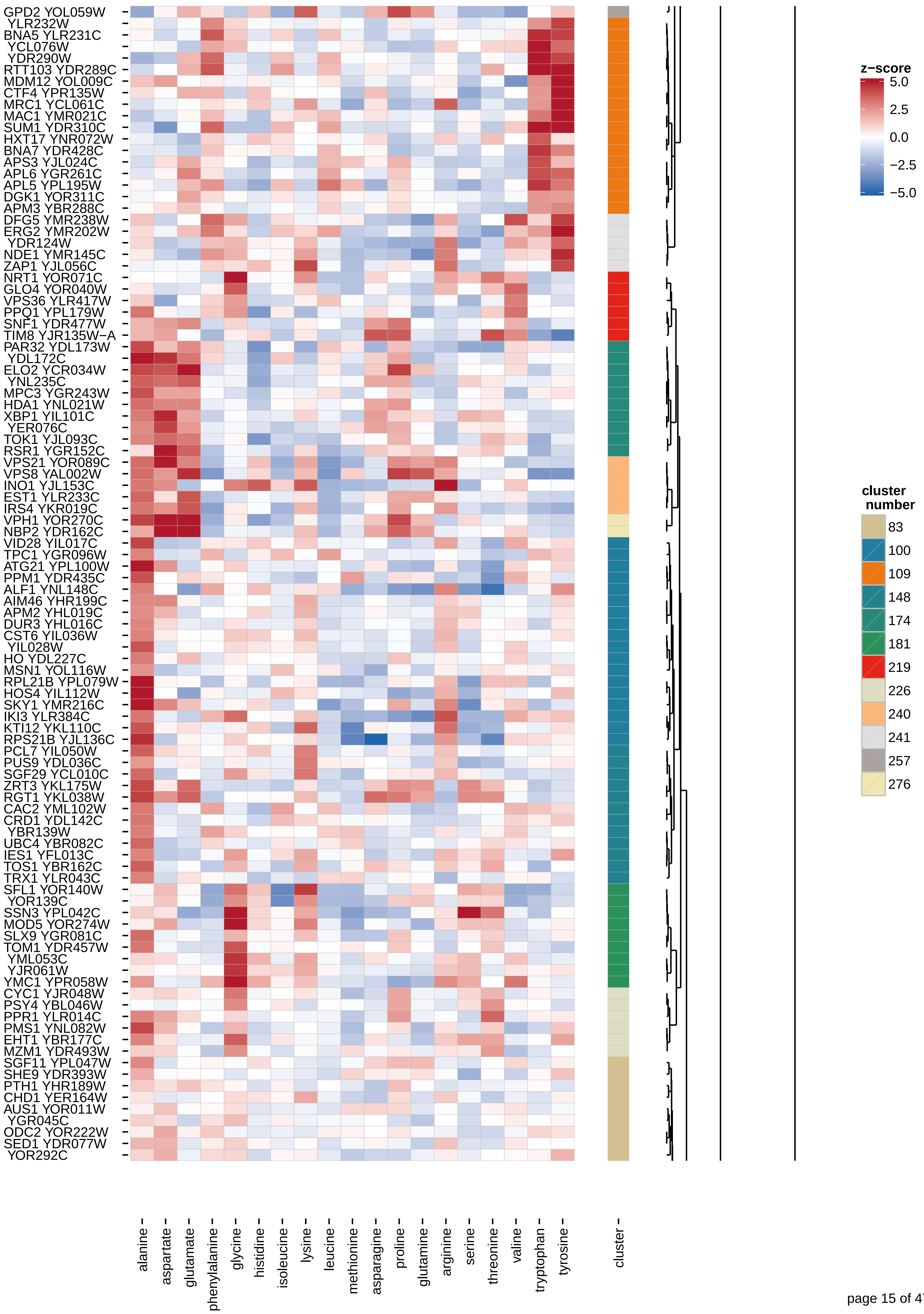
GPD2 YOL059W
YLR232W
BNA5 YLR231C
YCL076W
YDR290W
RTT103 YDR289C
MDM12 YOL009C
CTF4 YPR135W
MRC1 YCL061C
MAC1 YMR021C
SUM1 YDR310C
HXT17 YNR072W
BNA7 YDR428C
APS3 YJL024C
APL6 YGR261C
APL5 YPL195W
DGK1 YOR311C
APM3 YBR288C
DFG5 YMR238W
ERG2 YMR202W
YDR124W
NDE1 YMR145C
ZAP1 YJL056C
NRT1 YOR071C
GLO4 YOR040W
VPS36 YLR417W
PPQ1 YPL179W
SNF1 YDR477W
TIM8 YJR135W−A
PAR32 YDL173W
YDL172C
ELO2 YCR034W
YNL235C
MPC3 YGR243W
HDA1 YNL021W
XBP1 YIL101C
YER076C
TOK1 YJL093C
RSR1 YGR152C
VPS21 YOR089C
VPS8 YAL002W
INO1 YJL153C
EST1 YLR233C
IRS4 YKR019C
VPH1 YOR270C
NBP2 YDR162C
VID28 YIL017C
TPC1 YGR096W
ATG21 YPL100W
PPM1 YDR435C
ALF1 YNL148C
AIM46 YHR199C
APM2 YHL019C
DUR3 YHL016C
CST6 YIL036W
YIL028W
HO YDL227C
MSN1 YOL116W
RPL21B YPL079W
HOS4 YIL112W
SKY1 YMR216C
IKI3 YLR384C
KTI12 YKL110C
RPS21B YJL136C
PCL7 YIL050W
PUS9 YDL036C
SGF29 YCL010C
ZRT3 YKL175W
RGT1 YKL038W
CAC2 YML102W
CRD1 YDL142C
YBR139W
UBC4 YBR082C
IES1 YFL013C
TOS1 YBR162C
TRX1 YLR043C
SFL1 YOR140W
YOR139C
SSN3 YPL042C
MOD5 YOR274W
SLX9 YGR081C
TOM1 YDR457W
YML053C
YJR061W
YMC1 YPR058W
CYC1 YJR048W
PSY4 YBL046W
PPR1 YLR014C
PMS1 YNL082W
EHT1 YBR177C
MZM1 YDR493W
SGF11 YPL047W
SHE9 YDR393W
PTH1 YHR189W
CHD1 YER164W
AUS1 YOR011W
YGR045C
ODC2 YOR222W
SED1 YDR077W
YOR292C
alanine
aspartate
glutamate
phenylalanine
glycine
histidine
isoleucine
lysine
leucine
methionine
asparagine
proline
glutamine
arginine
serine
threonine
valine
tryptophan
tyrosine
cluster
z−score
5.0
2.5
0.0
−2.5
−5.0
cluster number
83
100
109
148
174
181
219
226
240
241
257
276

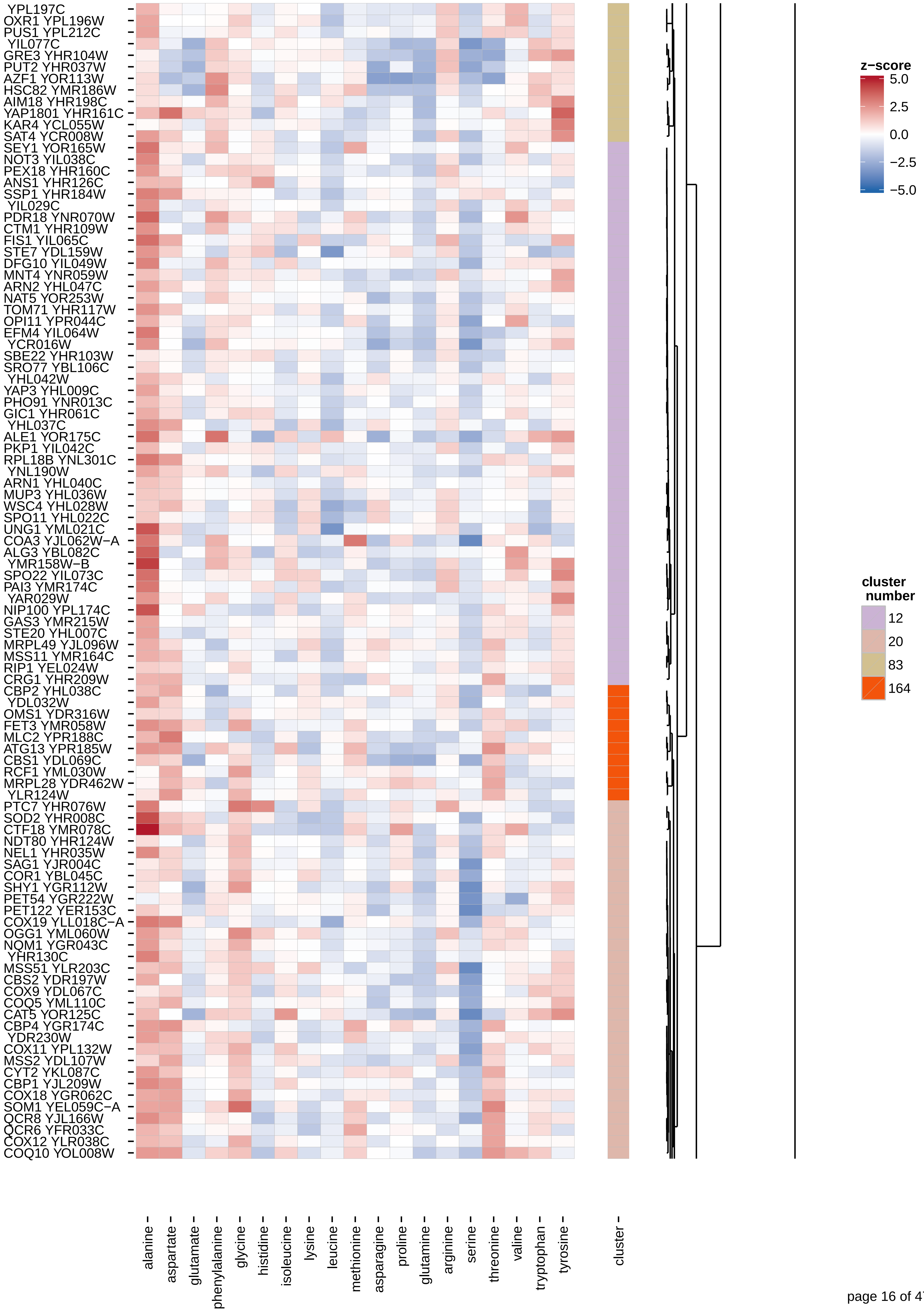
YPL197C
OXR1 YPL196W
PUS1 YPL212C
YIL077C
GRE3 YHR104W
PUT2 YHR037W
AZF1 YOR113W
HSC82 YMR186W
AIM18 YHR198C
YAP1801 YHR161C
KAR4 YCL055W
SAT4 YCR008W
SEY1 YOR165W
NOT3 YIL038C
PEX18 YHR160C
ANS1 YHR126C
SSP1 YHR184W
YIL029C
PDR18 YNR070W
CTM1 YHR109W
FIS1 YIL065C
STE7 YDL159W
DFG10 YIL049W
MNT4 YNR059W
ARN2 YHL047C
NAT5 YOR253W
TOM71 YHR117W
OPI11 YPR044C
EFM4 YIL064W
YCR016W
SBE22 YHR103W
SRO77 YBL106C
YHL042W
YAP3 YHL009C
PHO91 YNR013C
GIC1 YHR061C
YHL037C
ALE1 YOR175C
PKP1 YIL042C
RPL18B YNL301C
YNL190W
ARN1 YHL040C
MUP3 YHL036W
WSC4 YHL028W
SPO11 YHL022C
UNG1 YML021C
COA3 YJL062W−A
ALG3 YBL082C
YMR158W−B
SPO22 YIL073C
PAI3 YMR174C
YAR029W
NIP100 YPL174C
GAS3 YMR215W
STE20 YHL007C
MRPL49 YJL096W
MSS11 YMR164C
RIP1 YEL024W
CRG1 YHR209W
CBP2 YHL038C
YDL032W
OMS1 YDR316W
FET3 YMR058W
MLC2 YPR188C
ATG13 YPR185W
CBS1 YDL069C
RCF1 YML030W
MRPL28 YDR462W
YLR124W
PTC7 YHR076W
SOD2 YHR008C
CTF18 YMR078C
NDT80 YHR124W
NEL1 YHR035W
SAG1 YJR004C
COR1 YBL045C
SHY1 YGR112W
PET54 YGR222W
PET122 YER153C
COX19 YLL018C−A
OGG1 YML060W
NQM1 YGR043C
YHR130C
MSS51 YLR203C
CBS2 YDR197W
COX9 YDL067C
COQ5 YML110C
CAT5 YOR125C
CBP4 YGR174C
YDR230W
COX11 YPL132W
MSS2 YDL107W
CYT2 YKL087C
CBP1 YJL209W
COX18 YGR062C
SOM1 YEL059C−A
QCR8 YJL166W
QCR6 YFR033C
COX12 YLR038C
COQ10 YOL008W
alanine
aspartate
glutamate
phenylalanine
glycine
histidine
isoleucine
lysine
leucine
methionine
asparagine
proline
glutamine
arginine
serine
threonine
valine
tryptophan
tyrosine
cluster
z−score
5.0
2.5
0.0
−2.5
−5.0
cluster number
12
20
83
164

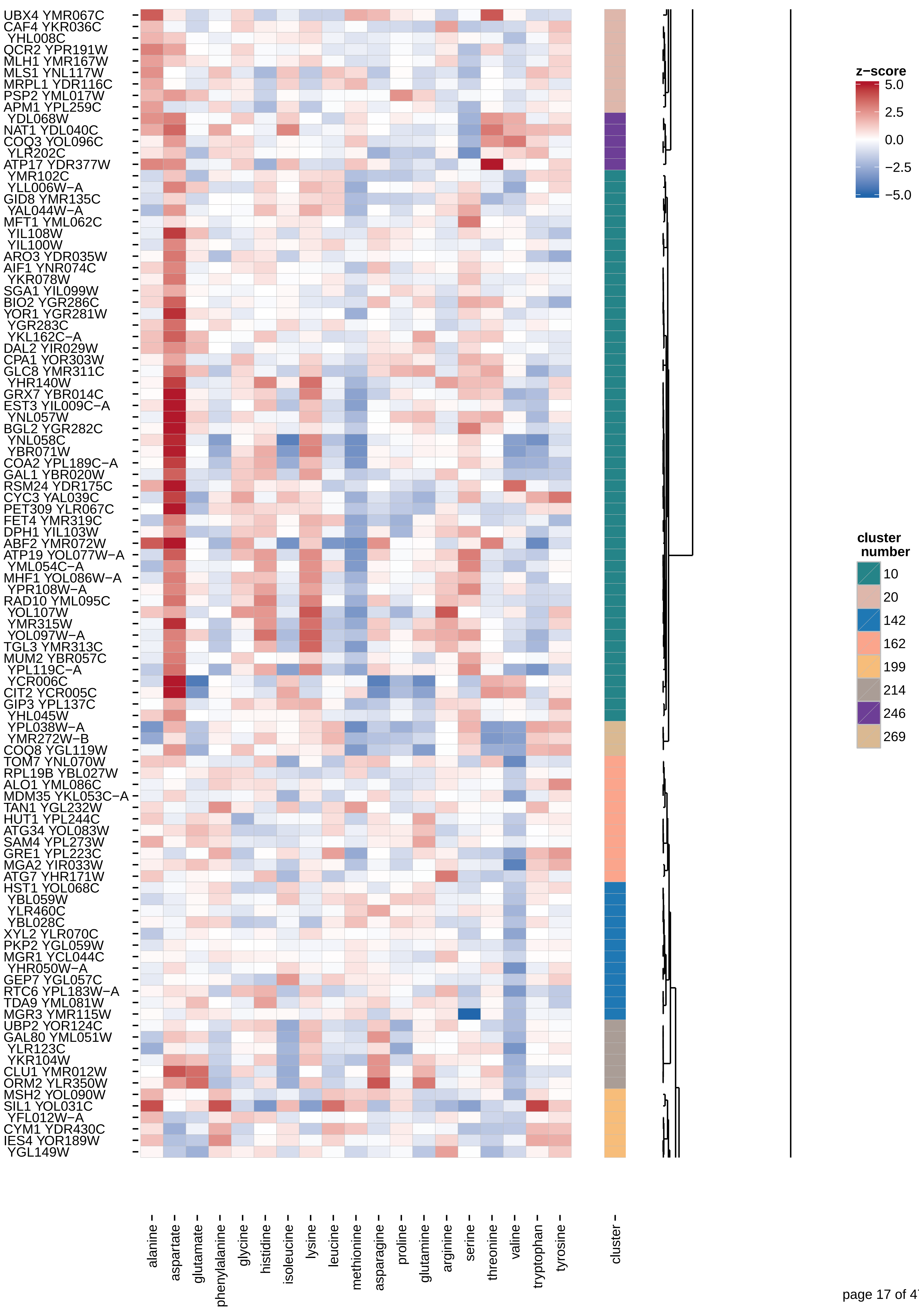
UBX4 YMR067C
CAF4 YKR036C
YHL008C
QCR2 YPR191W
MLH1 YMR167W
MLS1 YNL117W
MRPL1 YDR116C
PSP2 YML017W
APM1 YPL259C
YDL068W
NAT1 YDL040C
COQ3 YOL096C
YLR202C
ATP17 YDR377W
YMR102C
YLL006W−A
GID8 YMR135C
YAL044W−A
MFT1 YML062C
YIL108W
YIL100W
ARO3 YDR035W
AIF1 YNR074C
YKR078W
SGA1 YIL099W
BIO2 YGR286C
YOR1 YGR281W
YGR283C
YKL162C−A
DAL2 YIR029W
CPA1 YOR303W
GLC8 YMR311C
YHR140W
GRX7 YBR014C
EST3 YIL009C−A
YNL057W
BGL2 YGR282C
YNL058C
YBR071W
COA2 YPL189C−A
GAL1 YBR020W
RSM24 YDR175C
CYC3 YAL039C
PET309 YLR067C
FET4 YMR319C
DPH1 YIL103W
ABF2 YMR072W
ATP19 YOL077W−A
YML054C−A
MHF1 YOL086W−A
YPR108W−A
RAD10 YML095C
YOL107W
YMR315W
YOL097W−A
TGL3 YMR313C
MUM2 YBR057C
YPL119C−A
YCR006C
CIT2 YCR005C
GIP3 YPL137C
YHL045W
YPL038W−A
YMR272W−B
COQ8 YGL119W
TOM7 YNL070W
RPL19B YBL027W
ALO1 YML086C
MDM35 YKL053C−A
TAN1 YGL232W
HUT1 YPL244C
ATG34 YOL083W
SAM4 YPL273W
GRE1 YPL223C
MGA2 YIR033W
ATG7 YHR171W
HST1 YOL068C
YBL059W
YLR460C
YBL028C
XYL2 YLR070C
PKP2 YGL059W
MGR1 YCL044C
YHR050W−A
GEP7 YGL057C
RTC6 YPL183W−A
TDA9 YML081W
MGR3 YMR115W
UBP2 YOR124C
GAL80 YML051W
YLR123C
YKR104W
CLU1 YMR012W
ORM2 YLR350W
MSH2 YOL090W
SIL1 YOL031C
YFL012W−A
CYM1 YDR430C
IES4 YOR189W
YGL149W
alanine
aspartate
glutamate
phenylalanine
glycine
histidine
isoleucine
lysine
leucine
methionine
asparagine
proline
glutamine
arginine
serine
threonine
valine
tryptophan
tyrosine
cluster
z−score
5.0
2.5
0.0
−2.5
−5.0
cluster number
10
20
142
162
199
214
246
269

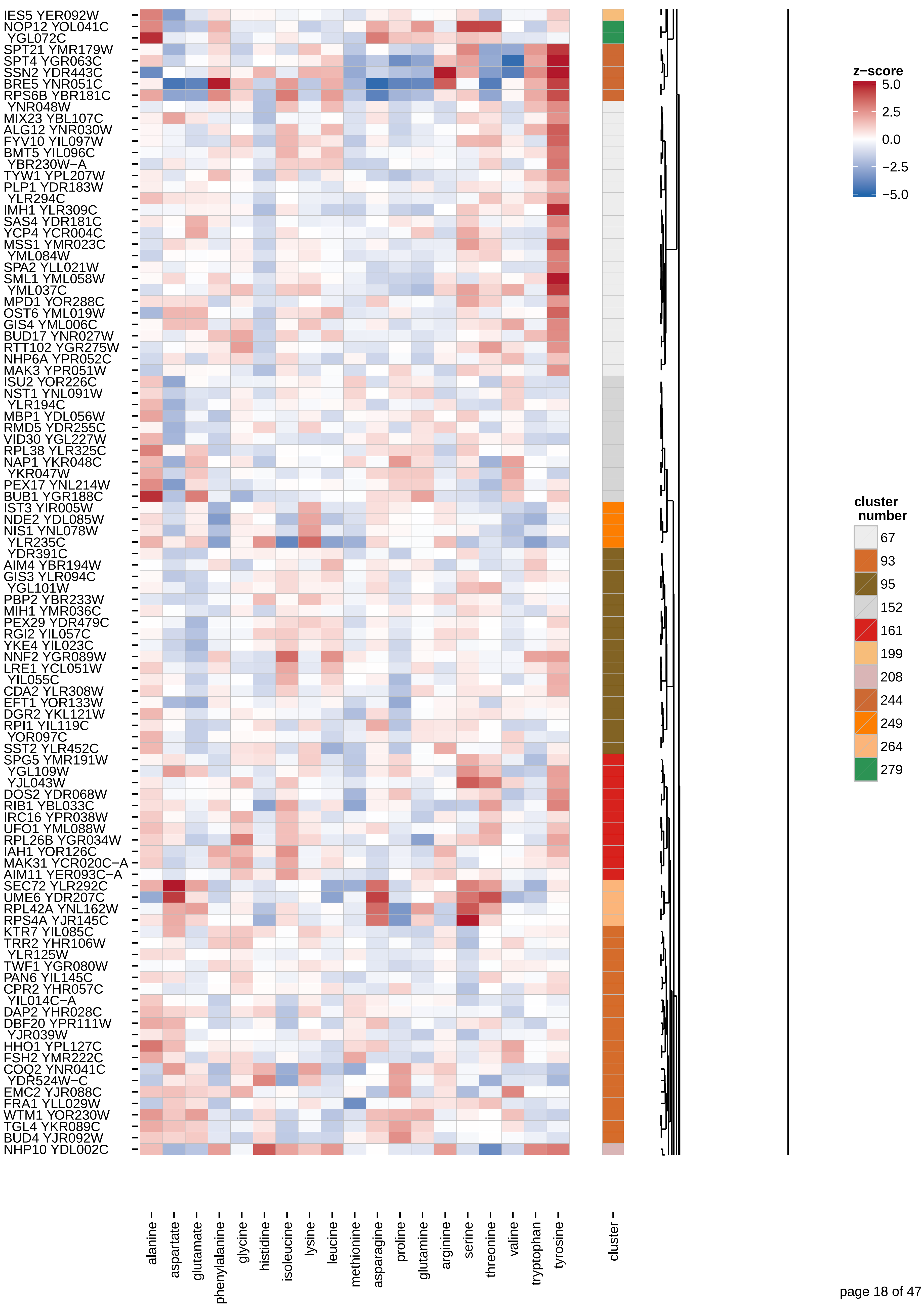

IES5 YER092W
NOP12 YOL041C
YGL072C
SPT21 YMR179W
SPT4 YGR063C
SSN2 YDR443C
BRE5 YNR051C
RPS6B YBR181C
YNR048W
MIX23 YBL107C
ALG12 YNR030W
FYV10 YIL097W
BMT5 YIL096C
YBR230W−A
TYW1 YPL207W
PLP1 YDR183W
YLR294C
IMH1 YLR309C
SAS4 YDR181C
YCP4 YCR004C
MSS1 YMR023C
YML084W
SPA2 YLL021W
SML1 YML058W
YML037C
MPD1 YOR288C
OST6 YML019W
GIS4 YML006C
BUD17 YNR027W
RTT102 YGR275W
NHP6A YPR052C
MAK3 YPR051W
ISU2 YOR226C
NST1 YNL091W
YLR194C
MBP1 YDL056W
RMD5 YDR255C
VID30 YGL227W
RPL38 YLR325C
NAP1 YKR048C
YKR047W
PEX17 YNL214W
BUB1 YGR188C
IST3 YIR005W
NDE2 YDL085W
NIS1 YNL078W
YLR235C
YDR391C
AIM4 YBR194W
GIS3 YLR094C
YGL101W
PBP2 YBR233W
MIH1 YMR036C
PEX29 YDR479C
RGI2 YIL057C
YKE4 YIL023C
NNF2 YGR089W
LRE1 YCL051W
YIL055C
CDA2 YLR308W
EFT1 YOR133W
DGR2 YKL121W
RPI1 YIL119C
YOR097C
SST2 YLR452C
SPG5 YMR191W
YGL109W
YJL043W
DOS2 YDR068W
RIB1 YBL033C
IRC16 YPR038W
UFO1 YML088W
RPL26B YGR034W
IAH1 YOR126C
MAK31 YCR020C−A
AIM11 YER093C−A
SEC72 YLR292C
UME6 YDR207C
RPL42A YNL162W
RPS4A YJR145C
KTR7 YIL085C
TRR2 YHR106W
YLR125W
TWF1 YGR080W
PAN6 YIL145C
CPR2 YHR057C
YIL014C−A
DAP2 YHR028C
DBF20 YPR111W
YJR039W
HHO1 YPL127C
FSH2 YMR222C
COQ2 YNR041C
YDR524W−C
EMC2 YJR088C
FRA1 YLL029W
WTM1 YOR230W
TGL4 YKR089C
BUD4 YJR092W
NHP10 YDL002C
alanine
aspartate
glutamate
phenylalanine
glycine
histidine
isoleucine
lysine
leucine
methionine
asparagine
proline
glutamine
arginine
serine
threonine
valine
tryptophan
tyrosine
cluster
z−score
5.0
2.5
0.0
−2.5
−5.0
cluster number
67
93
95
152
161
199
208
244
249
264
279

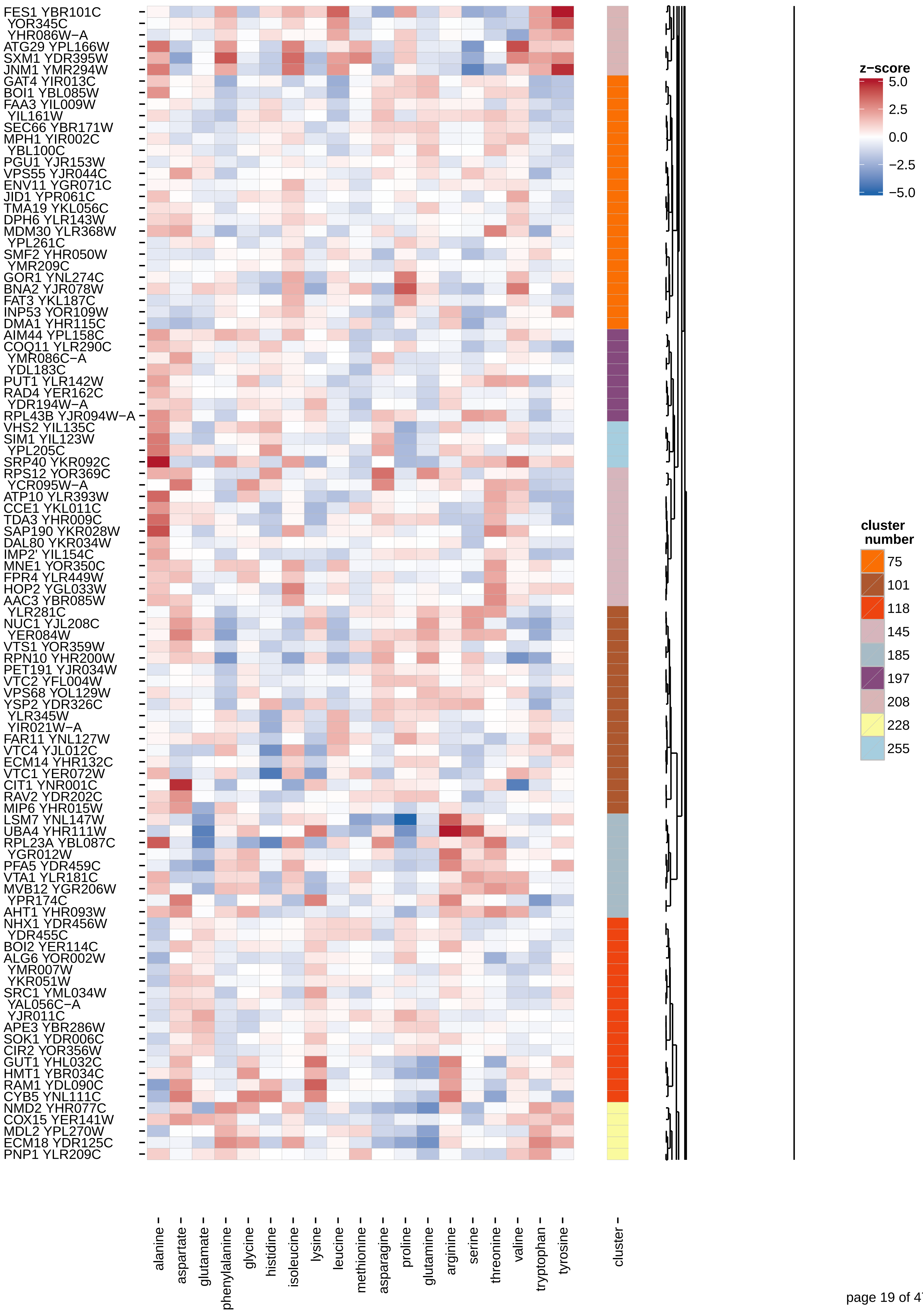
FES1 YBR101C
YOR345C
YHR086W−A
ATG29 YPL166W
SXM1 YDR395W
JNM1 YMR294W
GAT4 YIR013C
BOI1 YBL085W
FAA3 YIL009W
YIL161W
SEC66 YBR171W
MPH1 YIR002C
YBL100C
PGU1 YJR153W
VPS55 YJR044C
ENV11 YGR071C
JID1 YPR061C
TMA19 YKL056C
DPH6 YLR143W
MDM30 YLR368W
YPL261C
SMF2 YHR050W
YMR209C
GOR1 YNL274C
BNA2 YJR078W
FAT3 YKL187C
INP53 YOR109W
DMA1 YHR115C
AIM44 YPL158C
COQ11 YLR290C
YMR086C−A
YDL183C
PUT1 YLR142W
RAD4 YER162C
YDR194W−A
RPL43B YJR094W−A
VHS2 YIL135C
SIM1 YIL123W
YPL205C
SRP40 YKR092C
RPS12 YOR369C
YCR095W−A
ATP10 YLR393W
CCE1 YKL011C
TDA3 YHR009C
SAP190 YKR028W
DAL80 YKR034W
IMP2' YIL154C
MNE1 YOR350C
FPR4 YLR449W
HOP2 YGL033W
AAC3 YBR085W
YLR281C
NUC1 YJL208C
YER084W
VTS1 YOR359W
RPN10 YHR200W
PET191 YJR034W
VTC2 YFL004W
VPS68 YOL129W
YSP2 YDR326C
YLR345W
YIR021W−A
FAR11 YNL127W
VTC4 YJL012C
ECM14 YHR132C
VTC1 YER072W
CIT1 YNR001C
RAV2 YDR202C
MIP6 YHR015W
LSM7 YNL147W
UBA4 YHR111W
RPL23A YBL087C
YGR012W
PFA5 YDR459C
VTA1 YLR181C
MVB12 YGR206W
YPR174C
AHT1 YHR093W
NHX1 YDR456W
YDR455C
BOI2 YER114C
ALG6 YOR002W
YMR007W
YKR051W
SRC1 YML034W
YAL056C−A
YJR011C
APE3 YBR286W
SOK1 YDR006C
CIR2 YOR356W
GUT1 YHL032C
HMT1 YBR034C
RAM1 YDL090C
CYB5 YNL111C
NMD2 YHR077C
COX15 YER141W
MDL2 YPL270W
ECM18 YDR125C
PNP1 YLR209C
alanine
aspartate
glutamate
phenylalanine
glycine
histidine
isoleucine
lysine
leucine
methionine
asparagine
proline
glutamine
arginine
serine
threonine
valine
tryptophan
tyrosine
cluster
z−score
5.0
2.5
0.0
−2.5
−5.0
cluster number
75
101
118
145
185
197
208
228
255

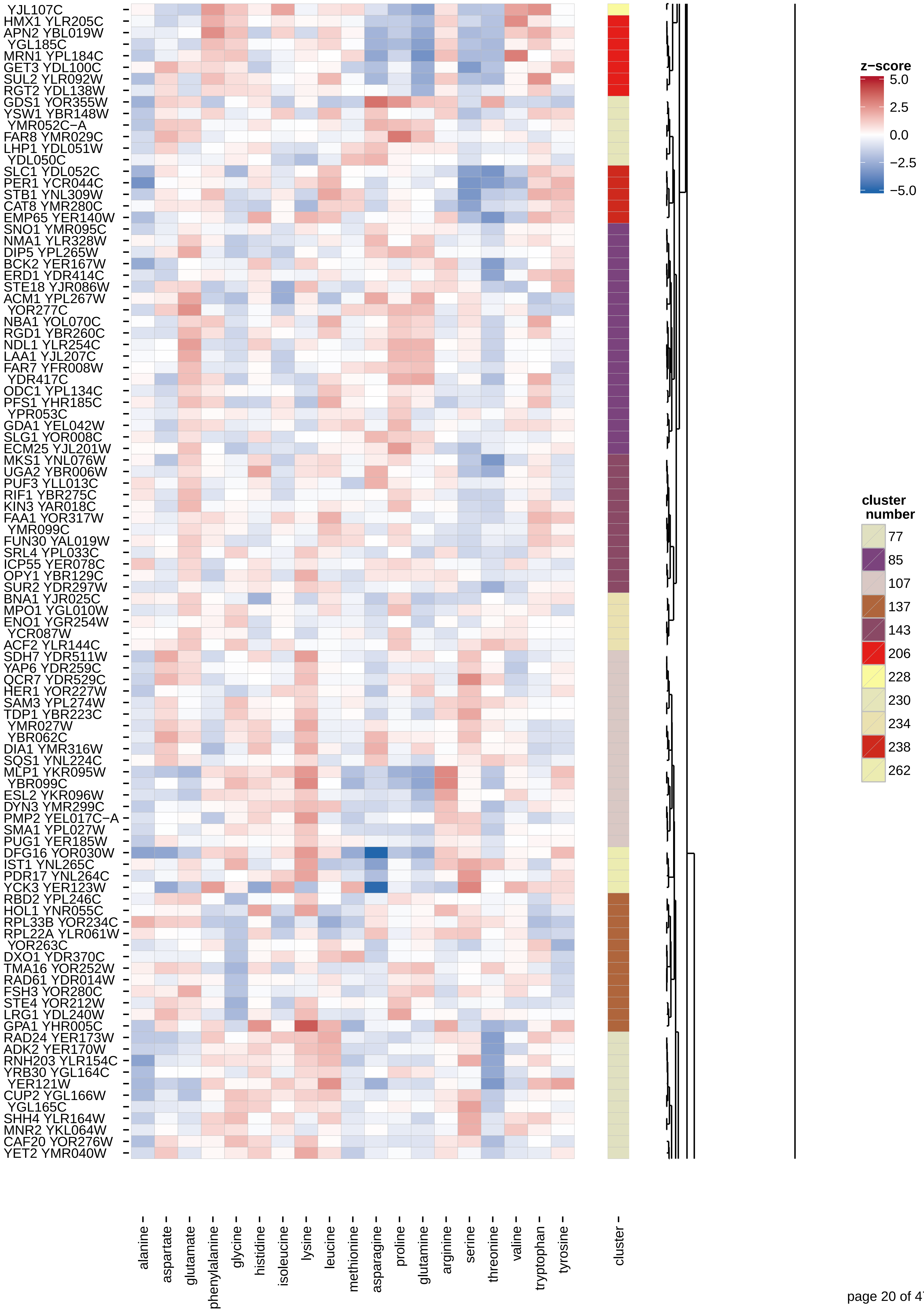
YJL107C
HMX1 YLR205C
APN2 YBL019W
YGL185C
MRN1 YPL184C
GET3 YDL100C
SUL2 YLR092W
RGT2 YDL138W
GDS1 YOR355W
YSW1 YBR148W
YMR052C−A
FAR8 YMR029C
LHP1 YDL051W
YDL050C
SLC1 YDL052C
PER1 YCR044C
STB1 YNL309W
CAT8 YMR280C
EMP65 YER140W
SNO1 YMR095C
NMA1 YLR328W
DIP5 YPL265W
BCK2 YER167W
ERD1 YDR414C
STE18 YJR086W
ACM1 YPL267W
YOR277C
NBA1 YOL070C
RGD1 YBR260C
NDL1 YLR254C
LAA1 YJL207C
FAR7 YFR008W
YDR417C
ODC1 YPL134C
PFS1 YHR185C
YPR053C
GDA1 YEL042W
SLG1 YOR008C
ECM25 YJL201W
MKS1 YNL076W
UGA2 YBR006W
PUF3 YLL013C
RIF1 YBR275C
KIN3 YAR018C
FAA1 YOR317W
YMR099C
FUN30 YAL019W
SRL4 YPL033C
ICP55 YER078C
OPY1 YBR129C
SUR2 YDR297W
BNA1 YJR025C
MPO1 YGL010W
ENO1 YGR254W
YCR087W
ACF2 YLR144C
SDH7 YDR511W
YAP6 YDR259C
QCR7 YDR529C
HER1 YOR227W
SAM3 YPL274W
TDP1 YBR223C
YMR027W
YBR062C
DIA1 YMR316W
SQS1 YNL224C
MLP1 YKR095W
YBR099C
ESL2 YKR096W
DYN3 YMR299C
PMP2 YEL017C−A
SMA1 YPL027W
PUG1 YER185W
DFG16 YOR030W
IST1 YNL265C
PDR17 YNL264C
YCK3 YER123W
RBD2 YPL246C
HOL1 YNR055C
RPL33B YOR234C
RPL22A YLR061W
YOR263C
DXO1 YDR370C
TMA16 YOR252W
RAD61 YDR014W
FSH3 YOR280C
STE4 YOR212W
LRG1 YDL240W
GPA1 YHR005C
RAD24 YER173W
ADK2 YER170W
RNH203 YLR154C
YRB30 YGL164C
YER121W
CUP2 YGL166W
YGL165C
SHH4 YLR164W
MNR2 YKL064W
CAF20 YOR276W
YET2 YMR040W
alanine
aspartate
glutamate
phenylalanine
glycine
histidine
isoleucine
lysine
leucine
methionine
asparagine
proline
glutamine
arginine
serine
threonine
valine
tryptophan
tyrosine
cluster
z−score
5.0
2.5
0.0
−2.5
−5.0
cluster
number
77
85
107
137
143
206
228
230
234
238
262

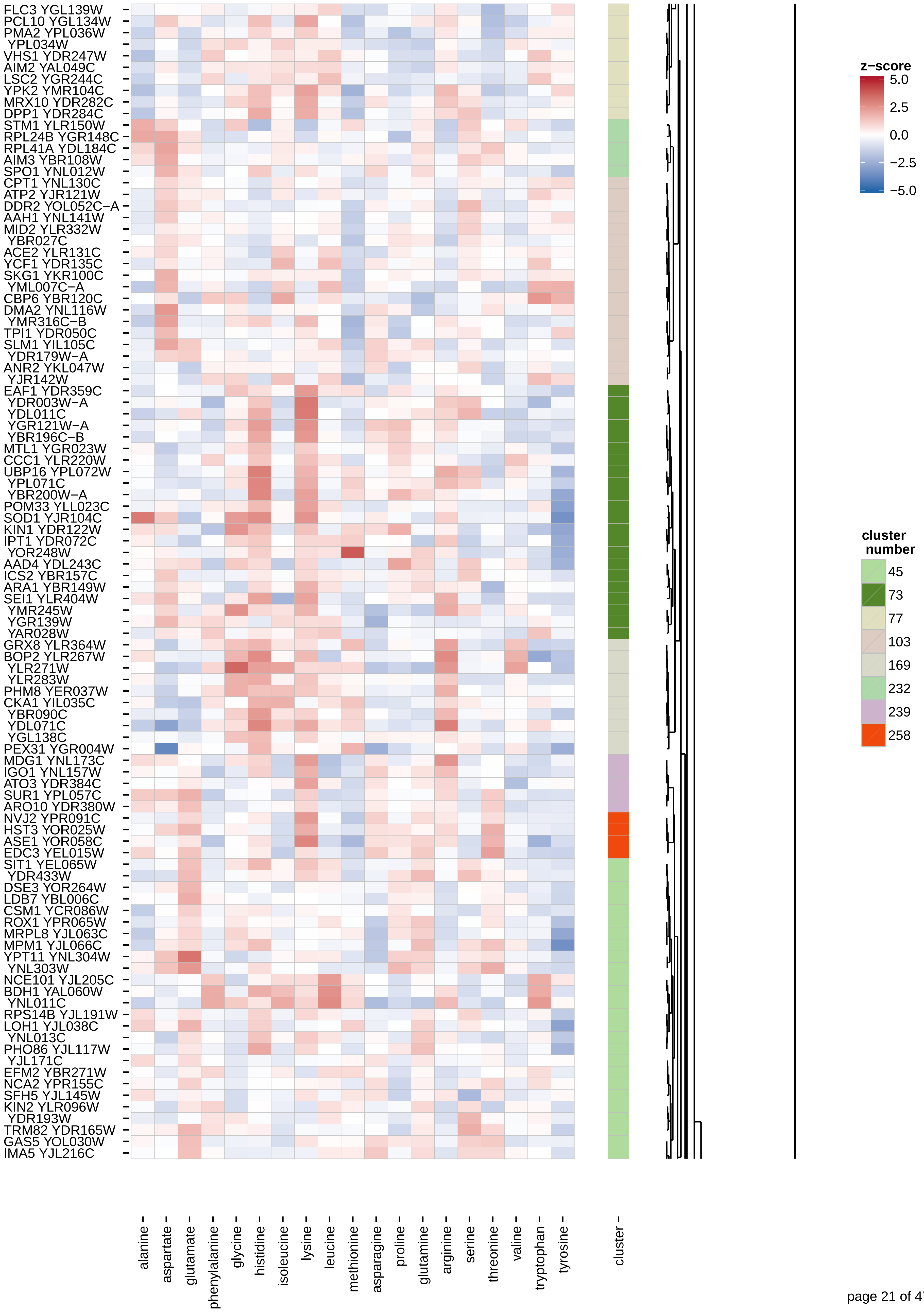

FLC3 YGL139W
PCL10 YGL134W
PMA2 YPL036W
YPL034W
VHS1 YDR247W
AIM2 YAL049C
LSC2 YGR244C
YPK2 YMR104C
MRX10 YDR282C
DPP1 YDR284C
STM1 YLR150W
RPL24B YGR148C
RPL41A YDL184C
AIM3 YBR108W
SPO1 YNL012W
CPT1 YNL130C
ATP2 YJR121W
DDR2 YOL052C−A
AAH1 YNL141W
MID2 YLR332W
YBR027C
ACE2 YLR131C
YCF1 YDR135C
SKG1 YKR100C
YML007C−A
CBP6 YBR120C
DMA2 YNL116W
YMR316C−B
TPI1 YDR050C
SLM1 YIL105C
YDR179W−A
ANR2 YKL047W
YJR142W
EAF1 YDR359C
YDR003W−A
YDL011C
YGR121W−A
YBR196C−B
MTL1 YGR023W
CCC1 YLR220W
UBP16 YPL072W
YPL071C
YBR200W−A
POM33 YLL023C
SOD1 YJR104C
KIN1 YDR122W
IPT1 YDR072C
YOR248W
AAD4 YDL243C
ICS2 YBR157C
ARA1 YBR149W
SEI1 YLR404W
YMR245W
YGR139W
YAR028W
GRX8 YLR364W
BOP2 YLR267W
YLR271W
YLR283W
PHM8 YER037W
CKA1 YIL035C
YBR090C
YDL071C
YGL138C
PEX31 YGR004W
MDG1 YNL173C
IGO1 YNL157W
ATO3 YDR384C
SUR1 YPL057C
ARO10 YDR380W
NVJ2 YPR091C
HST3 YOR025W
ASE1 YOR058C
EDC3 YEL015W
SIT1 YEL065W
YDR433W
DSE3 YOR264W
LDB7 YBL006C
CSM1 YCR086W
ROX1 YPR065W
MRPL8 YJL063C
MPM1 YJL066C
YPT11 YNL304W
YNL303W
NCE101 YJL205C
BDH1 YAL060W
YNL011C
RPS14B YJL191W
LOH1 YJL038C
YNL013C
PHO86 YJL117W
YJL171C
EFM2 YBR271W
NCA2 YPR155C
SFH5 YJL145W
KIN2 YLR096W
YDR193W
TRM82 YDR165W
GAS5 YOL030W
IMA5 YJL216C
alanine
aspartate
glutamate
phenylalanine
glycine
histidine
isoleucine
lysine
leucine
methionine
asparagine
proline
glutamine
arginine
serine
threonine
valine
tryptophan
tyrosine
cluster
z−score
5.0
2.5
0.0
−2.5
−5.0
cluster number
45
73
77
103
169
232
239
258

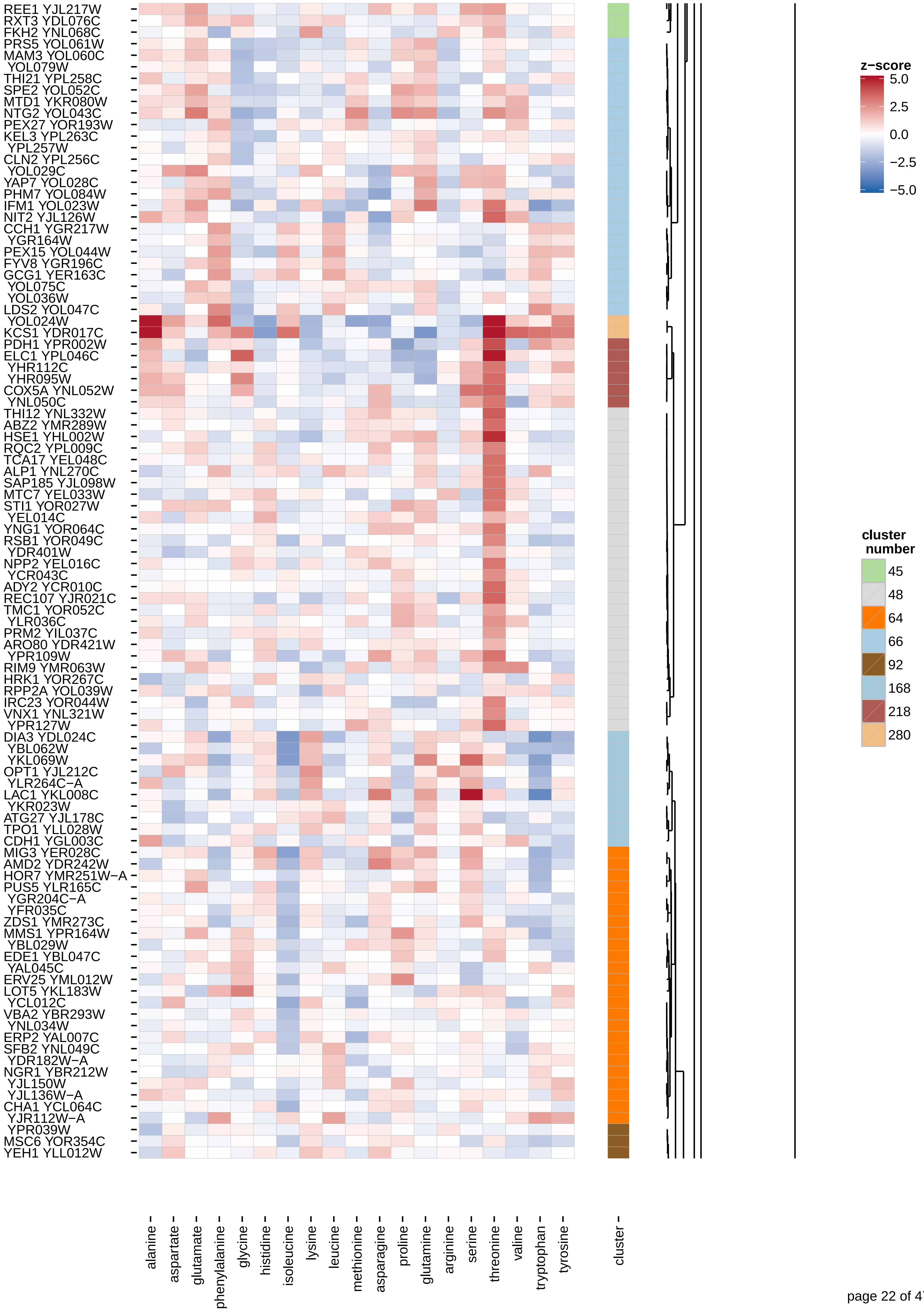
REE1 YJL217W
RXT3 YDL076C
FKH2 YNL068C
PRS5 YOL061W
MAM3 YOL060C
YOL079W
THI21 YPL258C
SPE2 YOL052C
MTD1 YKR080W
NTG2 YOL043C
PEX27 YOR193W
KEL3 YPL263C
YPL257W
CLN2 YPL256C
YOL029C
YAP7 YOL028C
PHM7 YOL084W
IFM1 YOL023W
NIT2 YJL126W
CCH1 YGR217W
YGR164W
PEX15 YOL044W
FYV8 YGR196C
GCG1 YER163C
YOL075C
YOL036W
LDS2 YOL047C
YOL024W
KCS1 YDR017C
PDH1 YPR002W
ELC1 YPL046C
YHR112C
YHR095W
COX5A YNL052W
YNL050C
THI12 YNL332W
ABZ2 YMR289W
HSE1 YHL002W
RQC2 YPL009C
TCA17 YEL048C
ALP1 YNL270C
SAP185 YJL098W
MTC7 YEL033W
STI1 YOR027W
YEL014C
YNG1 YOR064C
RSB1 YOR049C
YDR401W
NPP2 YEL016C
YCR043C
ADY2 YCR010C
REC107 YJR021C
TMC1 YOR052C
YLR036C
PRM2 YIL037C
ARO80 YDR421W
YPR109W
RIM9 YMR063W
HRK1 YOR267C
RPP2A YOL039W
IRC23 YOR044W
VNX1 YNL321W
YPR127W
DIA3 YDL024C
YBL062W
YKL069W
OPT1 YJL212C
YLR264C−A
LAC1 YKL008C
YKR023W
ATG27 YJL178C
TPO1 YLL028W
CDH1 YGL003C
MIG3 YER028C
AMD2 YDR242W
HOR7 YMR251W−A
PUS5 YLR165C
YGR204C−A
YFR035C
ZDS1 YMR273C
MMS1 YPR164W
YBL029W
EDE1 YBL047C
YAL045C
ERV25 YML012W
LOT5 YKL183W
YCL012C
VBA2 YBR293W
YNL034W
ERP2 YAL007C
SFB2 YNL049C
YDR182W−A
NGR1 YBR212W
YJL150W
YJL136W−A
CHA1 YCL064C
YJR112W−A
YPR039W
MSC6 YOR354C
YEH1 YLL012W
alanine
aspartate
glutamate
phenylalanine
glycine
histidine
isoleucine
lysine
leucine
methionine
asparagine
proline
glutamine
arginine
serine
threonine
valine
tryptophan
tyrosine
cluster
z−score
5.0
2.5
0.0
−2.5
−5.0
cluster
number
45
48
64
66
92
168
218
280

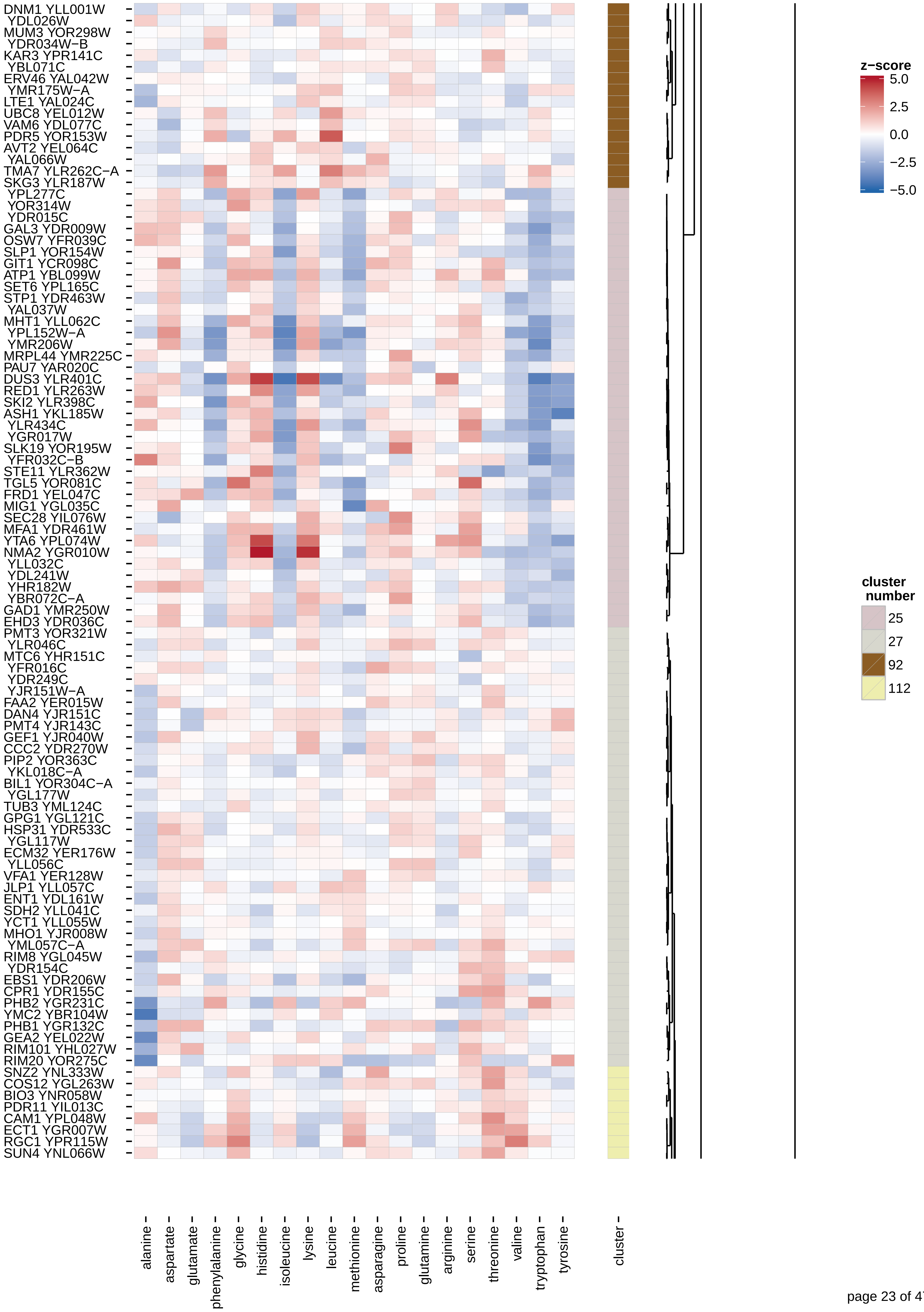
DNM1 YLL001W
YDL026W
MUM3 YOR298W
YDR034W−B
KAR3 YPR141C
YBL071C
ERV46 YAL042W
YMR175W−A
LTE1 YAL024C
UBC8 YEL012W
VAM6 YDL077C
PDR5 YOR153W
AVT2 YEL064C
YAL066W
TMA7 YLR262C−A
SKG3 YLR187W
YPL277C
YOR314W
YDR015C
GAL3 YDR009W
OSW7 YFR039C
SLP1 YOR154W
GIT1 YCR098C
ATP1 YBL099W
SET6 YPL165C
STP1 YDR463W
YAL037W
MHT1 YLL062C
YPL152W−A
YMR206W
MRPL44 YMR225C
PAU7 YAR020C
DUS3 YLR401C
RED1 YLR263W
SKI2 YLR398C
ASH1 YKL185W
YLR434C
YGR017W
SLK19 YOR195W
YFR032C−B
STE11 YLR362W
TGL5 YOR081C
FRD1 YEL047C
MIG1 YGL035C
SEC28 YIL076W
MFA1 YDR461W
YTA6 YPL074W
NMA2 YGR010W
YLL032C
YDL241W
YHR182W
YBR072C−A
GAD1 YMR250W
EHD3 YDR036C
PMT3 YOR321W
YLR046C
MTC6 YHR151C
YFR016C
YDR249C
YJR151W−A
FAA2 YER015W
DAN4 YJR151C
PMT4 YJR143C
GEF1 YJR040W
CCC2 YDR270W
PIP2 YOR363C
YKL018C−A
BIL1 YOR304C−A
YGL177W
TUB3 YML124C
GPG1 YGL121C
HSP31 YDR533C
YGL117W
ECM32 YER176W
YLL056C
VFA1 YER128W
JLP1 YLL057C
ENT1 YDL161W
SDH2 YLL041C
YCT1 YLL055W
MHO1 YJR008W
YML057C−A
RIM8 YGL045W
YDR154C
EBS1 YDR206W
CPR1 YDR155C
PHB2 YGR231C
YMC2 YBR104W
PHB1 YGR132C
GEA2 YEL022W
RIM101 YHL027W
RIM20 YOR275C
SNZ2 YNL333W
COS12 YGL263W
BIO3 YNR058W
PDR11 YIL013C
CAM1 YPL048W
ECT1 YGR007W
RGC1 YPR115W
SUN4 YNL066W
alanine
aspartate
glutamate
phenylalanine
glycine
histidine
isoleucine
lysine
leucine
methionine
asparagine
proline
glutamine
arginine
serine
threonine
valine
tryptophan
tyrosine
cluster
z−score
5.0
2.5
0.0
−2.5
−5.0
cluster number
25
27
92
112

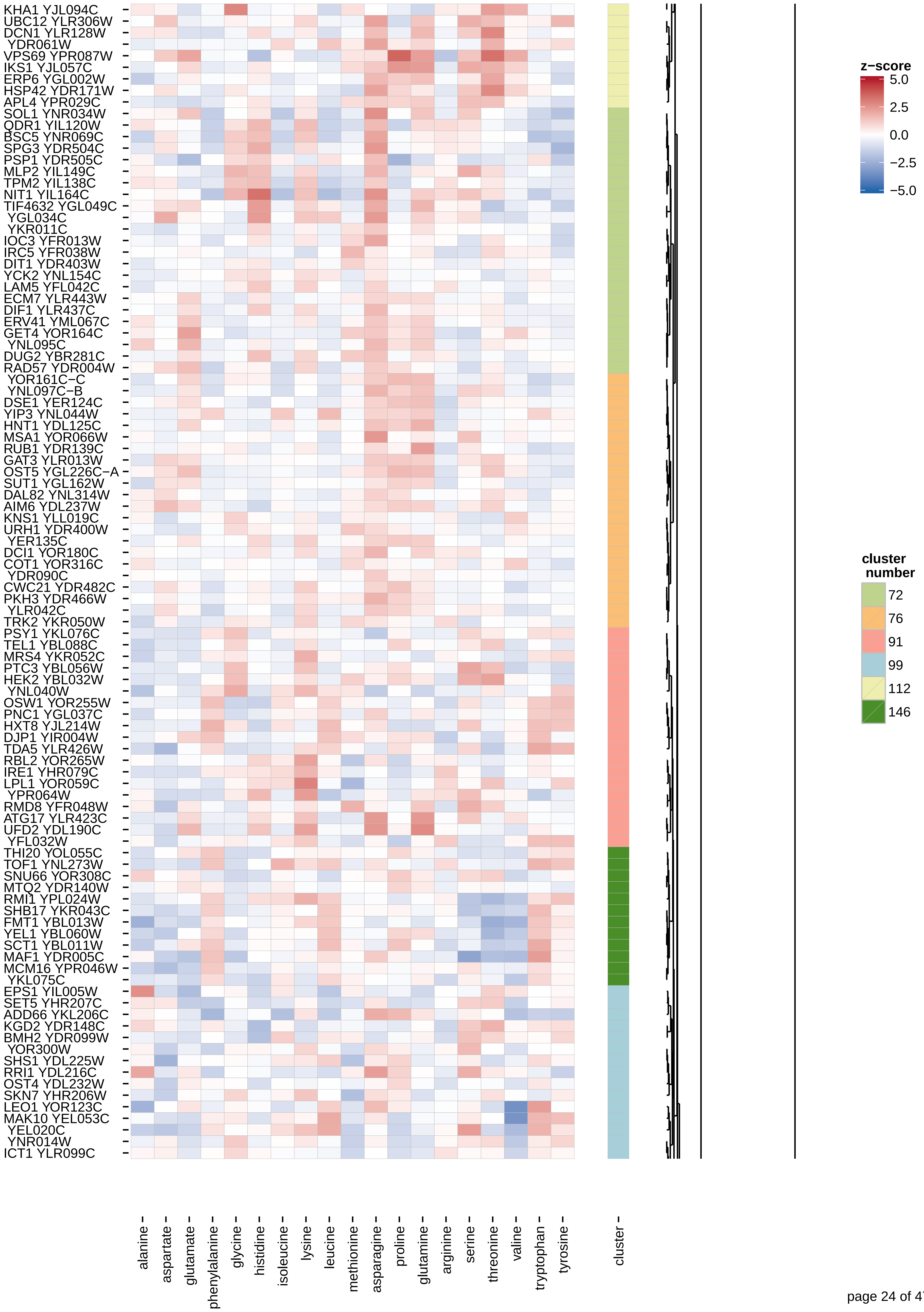
KHA1 YJL094C
UBC12 YLR306W
DCN1 YLR128W
YDR061W
VPS69 YPR087W
IKS1 YJL057C
ERP6 YGL002W
HSP42 YDR171W
APL4 YPR029C
SOL1 YNR034W
QDR1 YIL120W
BSC5 YNR069C
SPG3 YDR504C
PSP1 YDR505C
MLP2 YIL149C
TPM2 YIL138C
NIT1 YIL164C
TIF4632 YGL049C
YGL034C
YKR011C
IOC3 YFR013W
IRC5 YFR038W
DIT1 YDR403W
YCK2 YNL154C
LAM5 YFL042C
ECM7 YLR443W
DIF1 YLR437C
ERV41 YML067C
GET4 YOR164C
YNL095C
DUG2 YBR281C
RAD57 YDR004W
YOR161C−C
YNL097C−B
DSE1 YER124C
YIP3 YNL044W
HNT1 YDL125C
MSA1 YOR066W
RUB1 YDR139C
GAT3 YLR013W
OST5 YGL226C−A
SUT1 YGL162W
DAL82 YNL314W
AIM6 YDL237W
KNS1 YLL019C
URH1 YDR400W
YER135C
DCI1 YOR180C
COT1 YOR316C
YDR090C
CWC21 YDR482C
PKH3 YDR466W
YLR042C
TRK2 YKR050W
PSY1 YKL076C
TEL1 YBL088C
MRS4 YKR052C
PTC3 YBL056W
HEK2 YBL032W
YNL040W
OSW1 YOR255W
PNC1 YGL037C
HXT8 YJL214W
DJP1 YIR004W
TDA5 YLR426W
RBL2 YOR265W
IRE1 YHR079C
LPL1 YOR059C
YPR064W
RMD8 YFR048W
ATG17 YLR423C
UFD2 YDL190C
YFL032W
THI20 YOL055C
TOF1 YNL273W
SNU66 YOR308C
MTQ2 YDR140W
RMI1 YPL024W
SHB17 YKR043C
FMT1 YBL013W
YEL1 YBL060W
SCT1 YBL011W
MAF1 YDR005C
MCM16 YPR046W
YKL075C
EPS1 YIL005W
SET5 YHR207C
ADD66 YKL206C
KGD2 YDR148C
BMH2 YDR099W
YOR300W
SHS1 YDL225W
RRI1 YDL216C
OST4 YDL232W
SKN7 YHR206W
LEO1 YOR123C
MAK10 YEL053C
YEL020C
YNR014W
ICT1 YLR099C
alanine
aspartate
glutamate
phenylalanine
glycine
histidine
isoleucine
lysine
leucine
methionine
asparagine
proline
glutamine
arginine
serine
threonine
valine
tryptophan
tyrosine
cluster
z−score
5.0
2.5
0.0
−2.5
−5.0
cluster
number
72
76
91
99
112
146

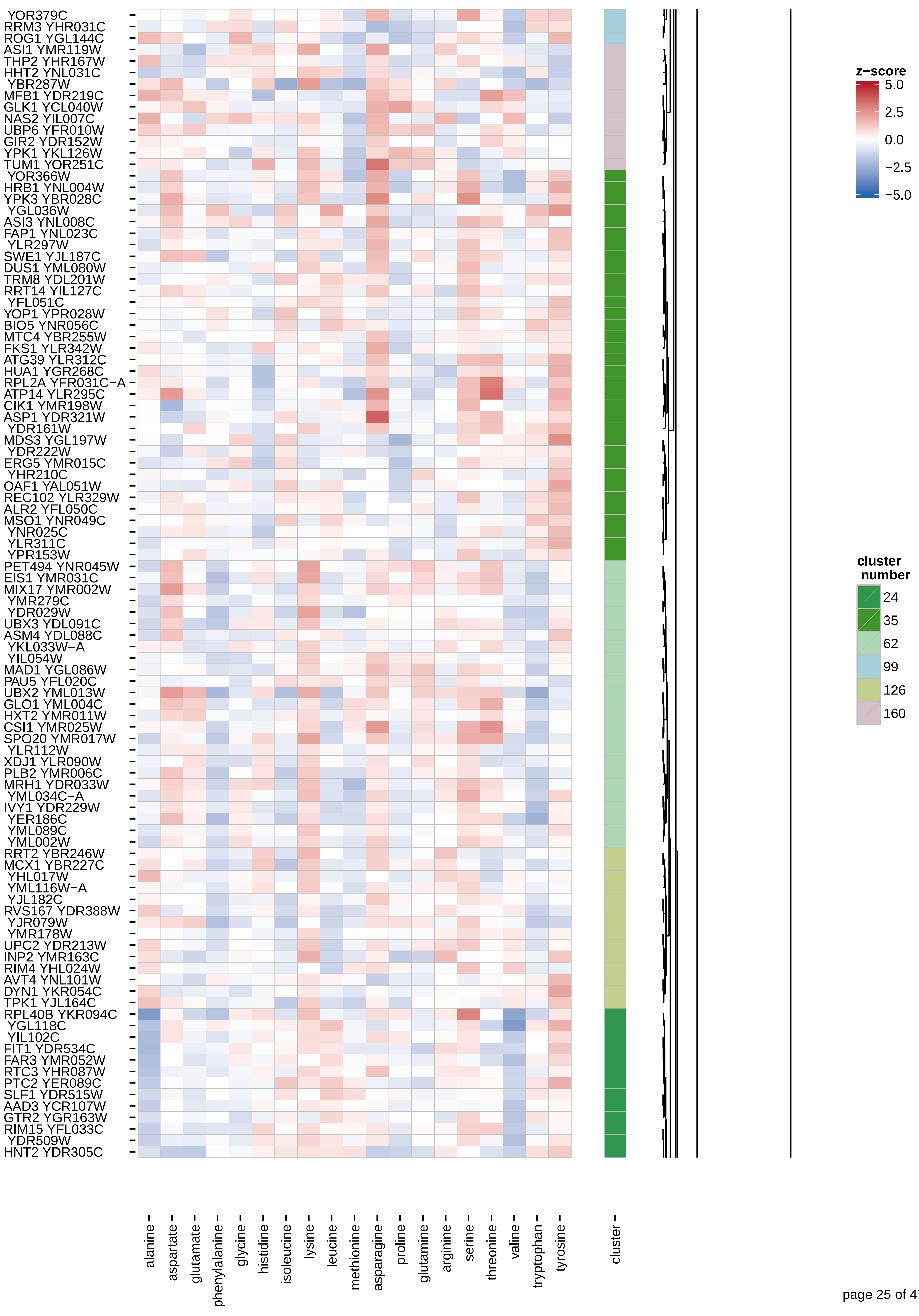
YOR379C
RRM3 YHR031C
ROG1 YGL144C
ASI1 YMR119W
THP2 YHR167W
HHT2 YNL031C
YBR287W
MFB1 YDR219C
GLK1 YCL040W
NAS2 YIL007C
UBP6 YFR010W
GIR2 YDR152W
YPK1 YKL126W
TUM1 YOR251C
YOR366W
HRB1 YNL004W
YPK3 YBR028C
YGL036W
ASI3 YNL008C
FAP1 YNL023C
YLR297W
SWE1 YJL187C
DUS1 YML080W
TRM8 YDL201W
RRT14 YIL127C
YFL051C
YOP1 YPR028W
BIO5 YNR056C
MTC4 YBR255W
FKS1 YLR342W
ATG39 YLR312C
HUA1 YGR268C
RPL2A YFR031C−A
ATP14 YLR295C
CIK1 YMR198W
ASP1 YDR321W
YDR161W
MDS3 YGL197W
YDR222W
ERG5 YMR015C
YHR210C
OAF1 YAL051W
REC102 YLR329W
ALR2 YFL050C
MSO1 YNR049C
YNR025C
YLR311C
YPR153W
PET494 YNR045W
EIS1 YMR031C
MIX17 YMR002W
YMR279C
YDR029W
UBX3 YDL091C
ASM4 YDL088C
YKL033W−A
YIL054W
MAD1 YGL086W
PAU5 YFL020C
UBX2 YML013W
GLO1 YML004C
HXT2 YMR011W
CSI1 YMR025W
SPO20 YMR017W
YLR112W
XDJ1 YLR090W
PLB2 YMR006C
MRH1 YDR033W
YML034C−A
IVY1 YDR229W
YER186C
YML089C
YML002W
RRT2 YBR246W
MCX1 YBR227C
YHL017W
YML116W−A
YJL182C
RVS167 YDR388W
YJR079W
YMR178W
UPC2 YDR213W
INP2 YMR163C
RIM4 YHL024W
AVT4 YNL101W
DYN1 YKR054C
TPK1 YJL164C
RPL40B YKR094C
YGL118C
YIL102C
FIT1 YDR534C
FAR3 YMR052W
RTC3 YHR087W
PTC2 YER089C
SLF1 YDR515W
AAD3 YCR107W
GTR2 YGR163W
RIM15 YFL033C
YDR509W
HNT2 YDR305C
alanine
aspartate
glutamate
phenylalanine
glycine
histidine
isoleucine
lysine
leucine
methionine
asparagine
proline
glutamine
arginine
serine
threonine
valine
tryptophan
tyrosine
cluster
z−score
5.0
2.5
0.0
−2.5
−5.0
cluster number
24
35
62
99
126
160

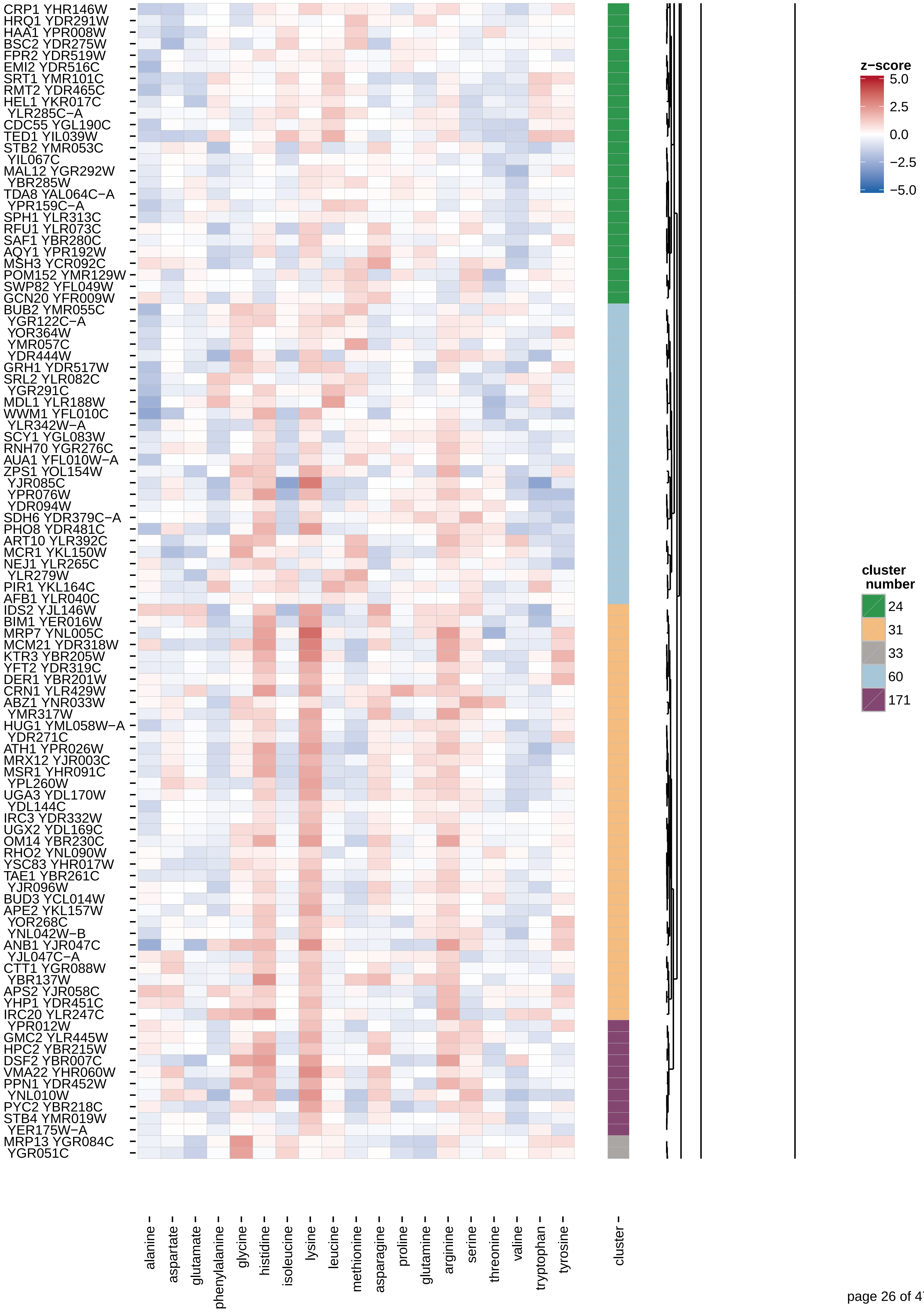

CRP1 YHR146W
HRQ1 YDR291W
HAA1 YPR008W
BSC2 YDR275W
FPR2 YDR519W
EMI2 YDR516C
SRT1 YMR101C
RMT2 YDR465C
HEL1 YKR017C
YLR285C−A
CDC55 YGL190C
TED1 YIL039W
STB2 YMR053C
YIL067C
MAL12 YGR292W
YBR285W
TDA8 YAL064C−A
YPR159C−A
SPH1 YLR313C
RFU1 YLR073C
SAF1 YBR280C
AQY1 YPR192W
MSH3 YCR092C
POM152 YMR129W
SWP82 YFL049W
GCN20 YFR009W
BUB2 YMR055C
YGR122C−A
YOR364W
YMR057C
YDR444W
GRH1 YDR517W
SRL2 YLR082C
YGR291C
MDL1 YLR188W
WWM1 YFL010C
YLR342W−A
SCY1 YGL083W
RNH70 YGR276C
AUA1 YFL010W−A
ZPS1 YOL154W
YJR085C
YPR076W
YDR094W
SDH6 YDR379C−A
PHO8 YDR481C
ART10 YLR392C
MCR1 YKL150W
NEJ1 YLR265C
YLR279W
PIR1 YKL164C
AFB1 YLR040C
IDS2 YJL146W
BIM1 YER016W
MRP7 YNL005C
MCM21 YDR318W
KTR3 YBR205W
YFT2 YDR319C
DER1 YBR201W
CRN1 YLR429W
ABZ1 YNR033W
YMR317W
HUG1 YML058W−A
YDR271C
ATH1 YPR026W
MRX12 YJR003C
MSR1 YHR091C
YPL260W
UGA3 YDL170W
YDL144C
IRC3 YDR332W
UGX2 YDL169C
OM14 YBR230C
RHO2 YNL090W
YSC83 YHR017W
TAE1 YBR261C
YJR096W
BUD3 YCL014W
APE2 YKL157W
YOR268C
YNL042W−B
ANB1 YJR047C
YJL047C−A
CTT1 YGR088W
YBR137W
APS2 YJR058C
YHP1 YDR451C
IRC20 YLR247C
YPR012W
GMC2 YLR445W
HPC2 YBR215W
DSF2 YBR007C
VMA22 YHR060W
PPN1 YDR452W
YNL010W
PYC2 YBR218C
STB4 YMR019W
YER175W−A
MRP13 YGR084C
YGR051C
alanine
aspartate
glutamate
phenylalanine
glycine
histidine
isoleucine
lysine
leucine
methionine
asparagine
proline
glutamine
arginine
serine
threonine
valine
tryptophan
tyrosine
cluster
z−score
5.0
2.5
0.0
−2.5
−5.0
cluster number
24
31
33
60
171

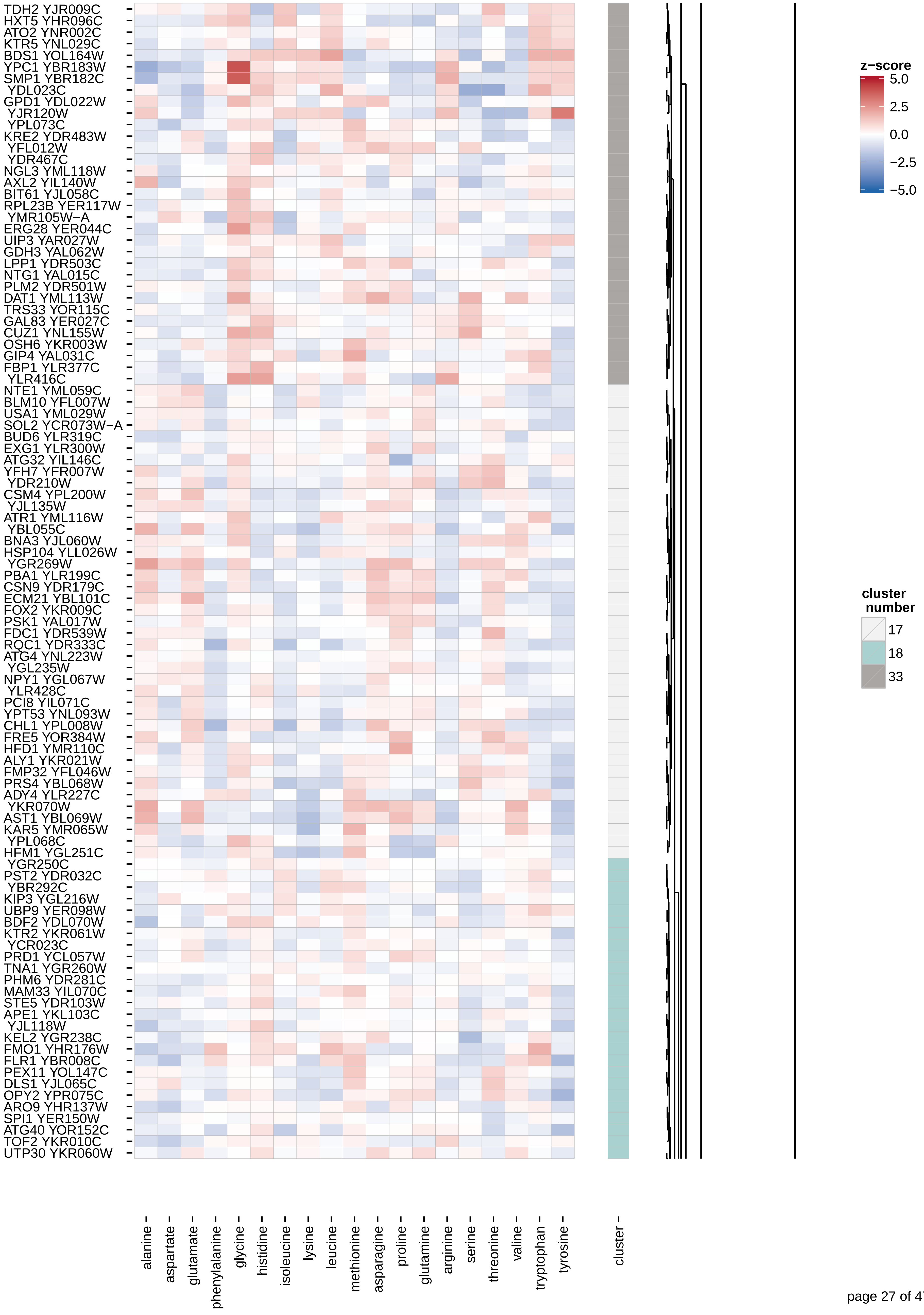
TDH2 YJR009C
HXT5 YHR096C
ATO2 YNR002C
KTR5 YNL029C
BDS1 YOL164W
YPC1 YBR183W
SMP1 YBR182C
YDL023C
GPD1 YDL022W
YJR120W
YPL073C
KRE2 YDR483W
YFL012W
YDR467C
NGL3 YML118W
AXL2 YIL140W
BIT61 YJL058C
RPL23B YER117W
YMR105W−A
ERG28 YER044C
UIP3 YAR027W
GDH3 YAL062W
LPP1 YDR503C
NTG1 YAL015C
PLM2 YDR501W
DAT1 YML113W
TRS33 YOR115C
GAL83 YER027C
CUZ1 YNL155W
OSH6 YKR003W
GIP4 YAL031C
FBP1 YLR377C
YLR416C
NTE1 YML059C
BLM10 YFL007W
USA1 YML029W
SOL2 YCR073W−A
BUD6 YLR319C
EXG1 YLR300W
ATG32 YIL146C
YFH7 YFR007W
YDR210W
CSM4 YPL200W
YJL135W
ATR1 YML116W
YBL055C
BNA3 YJL060W
HSP104 YLL026W
YGR269W
PBA1 YLR199C
CSN9 YDR179C
ECM21 YBL101C
FOX2 YKR009C
PSK1 YAL017W
FDC1 YDR539W
RQC1 YDR333C
ATG4 YNL223W
YGL235W
NPY1 YGL067W
YLR428C
PCI8 YIL071C
YPT53 YNL093W
CHL1 YPL008W
FRE5 YOR384W
HFD1 YMR110C
ALY1 YKR021W
FMP32 YFL046W
PRS4 YBL068W
ADY4 YLR227C
YKR070W
AST1 YBL069W
KAR5 YMR065W
YPL068C
HFM1 YGL251C
YGR250C
PST2 YDR032C
YBR292C
KIP3 YGL216W
UBP9 YER098W
BDF2 YDL070W
KTR2 YKR061W
YCR023C
PRD1 YCL057W
TNA1 YGR260W
PHM6 YDR281C
MAM33 YIL070C
STE5 YDR103W
APE1 YKL103C
YJL118W
KEL2 YGR238C
FMO1 YHR176W
FLR1 YBR008C
PEX11 YOL147C
DLS1 YJL065C
OPY2 YPR075C
ARO9 YHR137W
SPI1 YER150W
ATG40 YOR152C
TOF2 YKR010C
UTP30 YKR060W
alanine
aspartate
glutamate
phenylalanine
glycine
histidine
isoleucine
lysine
leucine
methionine
asparagine
proline
glutamine
arginine
serine
threonine
valine
tryptophan
tyrosine
cluster
z−score
5.0
2.5
0.0
−2.5
−5.0
cluster number
17
18
33

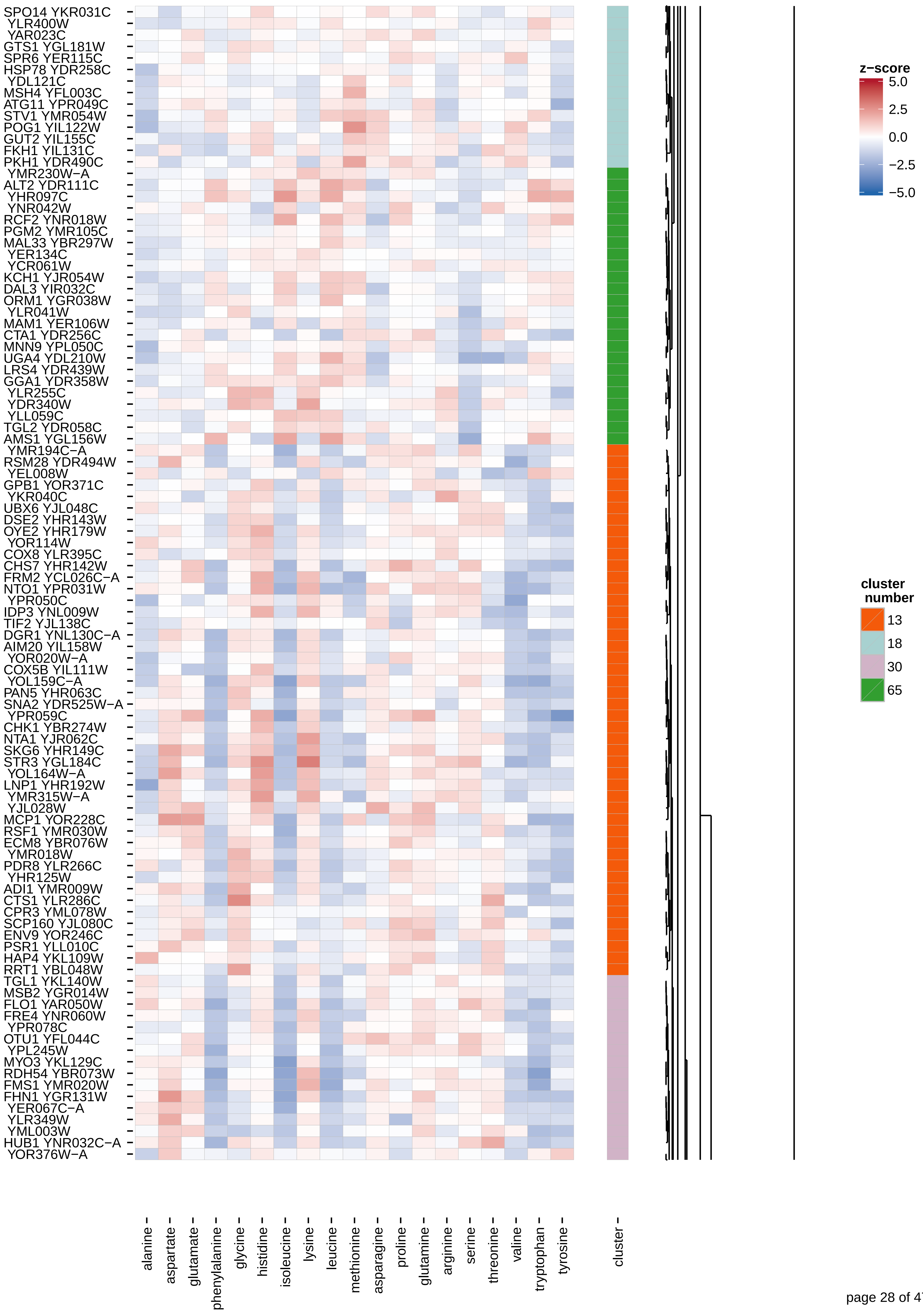

SPO14 YKR031C
YLR400W
YAR023C
GTS1 YGL181W
SPR6 YER115C
HSP78 YDR258C
YDL121C
MSH4 YFL003C
ATG11 YPR049C
STV1 YMR054W
POG1 YIL122W
GUT2 YIL155C
FKH1 YIL131C
PKH1 YDR490C
YMR230W−A
ALT2 YDR111C
YHR097C
YNR042W
RCF2 YNR018W
PGM2 YMR105C
MAL33 YBR297W
YER134C
YCR061W
KCH1 YJR054W
DAL3 YIR032C
ORM1 YGR038W
YLR041W
MAM1 YER106W
CTA1 YDR256C
MNN9 YPL050C
UGA4 YDL210W
LRS4 YDR439W
GGA1 YDR358W
YLR255C
YDR340W
YLL059C
TGL2 YDR058C
AMS1 YGL156W
YMR194C−A
RSM28 YDR494W
YEL008W
GPB1 YOR371C
YKR040C
UBX6 YJL048C
DSE2 YHR143W
OYE2 YHR179W
YOR114W
COX8 YLR395C
CHS7 YHR142W
FRM2 YCL026C−A
NTO1 YPR031W
YPR050C
IDP3 YNL009W
TIF2 YJL138C
DGR1 YNL130C−A
AIM20 YIL158W
YOR020W−A
COX5B YIL111W
YOL159C−A
PAN5 YHR063C
SNA2 YDR525W−A
YPR059C
CHK1 YBR274W
NTA1 YJR062C
SKG6 YHR149C
STR3 YGL184C
YOL164W−A
LNP1 YHR192W
YMR315W−A
YJL028W
MCP1 YOR228C
RSF1 YMR030W
ECM8 YBR076W
YMR018W
PDR8 YLR266C
YHR125W
ADI1 YMR009W
CTS1 YLR286C
CPR3 YML078W
SCP160 YJL080C
ENV9 YOR246C
PSR1 YLL010C
HAP4 YKL109W
RRT1 YBL048W
TGL1 YKL140W
MSB2 YGR014W
FLO1 YAR050W
FRE4 YNR060W
YPR078C
OTU1 YFL044C
YPL245W
MYO3 YKL129C
RDH54 YBR073W
FMS1 YMR020W
FHN1 YGR131W
YER067C−A
YLR349W
YML003W
HUB1 YNR032C−A
YOR376W−A
alanine
aspartate
glutamate
phenylalanine
glycine
histidine
isoleucine
lysine
leucine
methionine
asparagine
proline
glutamine
arginine
serine
threonine
valine
tryptophan
tyrosine
cluster
z−score
5.0
2.5
0.0
−2.5
−5.0
cluster number
13
18
30
65

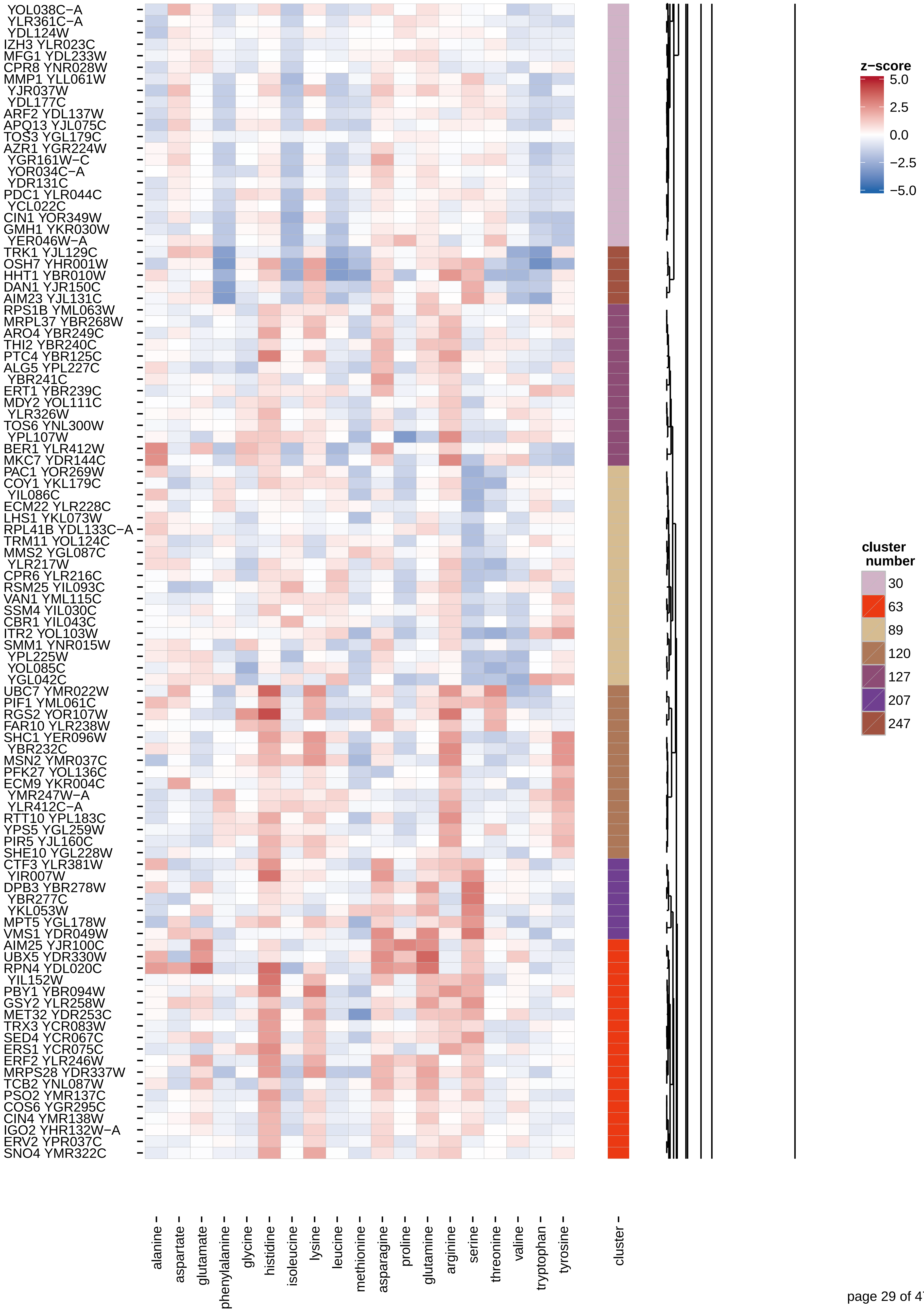

YOL038C−A
YLR361C−A
YDL124W
IZH3 YLR023C
MFG1 YDL233W
CPR8 YNR028W
MMP1 YLL061W
YJR037W
YDL177C
ARF2 YDL137W
APQ13 YJL075C
TOS3 YGL179C
AZR1 YGR224W
YGR161W−C
YOR034C−A
YDR131C
PDC1 YLR044C
YCL022C
CIN1 YOR349W
GMH1 YKR030W
YER046W−A
TRK1 YJL129C
OSH7 YHR001W
HHT1 YBR010W
DAN1 YJR150C
AIM23 YJL131C
RPS1B YML063W
MRPL37 YBR268W
ARO4 YBR249C
THI2 YBR240C
PTC4 YBR125C
ALG5 YPL227C
YBR241C
ERT1 YBR239C
MDY2 YOL111C
YLR326W
TOS6 YNL300W
YPL107W
BER1 YLR412W
MKC7 YDR144C
PAC1 YOR269W
COY1 YKL179C
YIL086C
ECM22 YLR228C
LHS1 YKL073W
RPL41B YDL133C−A
TRM11 YOL124C
MMS2 YGL087C
YLR217W
CPR6 YLR216C
RSM25 YIL093C
VAN1 YML115C
SSM4 YIL030C
CBR1 YIL043C
ITR2 YOL103W
SMM1 YNR015W
YPL225W
YOL085C
YGL042C
UBC7 YMR022W
PIF1 YML061C
RGS2 YOR107W
FAR10 YLR238W
SHC1 YER096W
YBR232C
MSN2 YMR037C
PFK27 YOL136C
ECM9 YKR004C
YMR247W−A
YLR412C−A
RTT10 YPL183C
YPS5 YGL259W
PIR5 YJL160C
SHE10 YGL228W
CTF3 YLR381W
YIR007W
DPB3 YBR278W
YBR277C
YKL053W
MPT5 YGL178W
VMS1 YDR049W
AIM25 YJR100C
UBX5 YDR330W
RPN4 YDL020C
YIL152W
PBY1 YBR094W
GSY2 YLR258W
MET32 YDR253C
TRX3 YCR083W
SED4 YCR067C
ERS1 YCR075C
ERF2 YLR246W
MRPS28 YDR337W
TCB2 YNL087W
PSO2 YMR137C
COS6 YGR295C
CIN4 YMR138W
IGO2 YHR132W−A
ERV2 YPR037C
SNO4 YMR322C
alanine
aspartate
glutamate
phenylalanine
glycine
histidine
isoleucine
lysine
leucine
methionine
asparagine
proline
glutamine
arginine
serine
threonine
valine
tryptophan
tyrosine
cluster
z−score
5.0
2.5
0.0
−2.5
−5.0
cluster
number
30
63
89
120
127
207
247

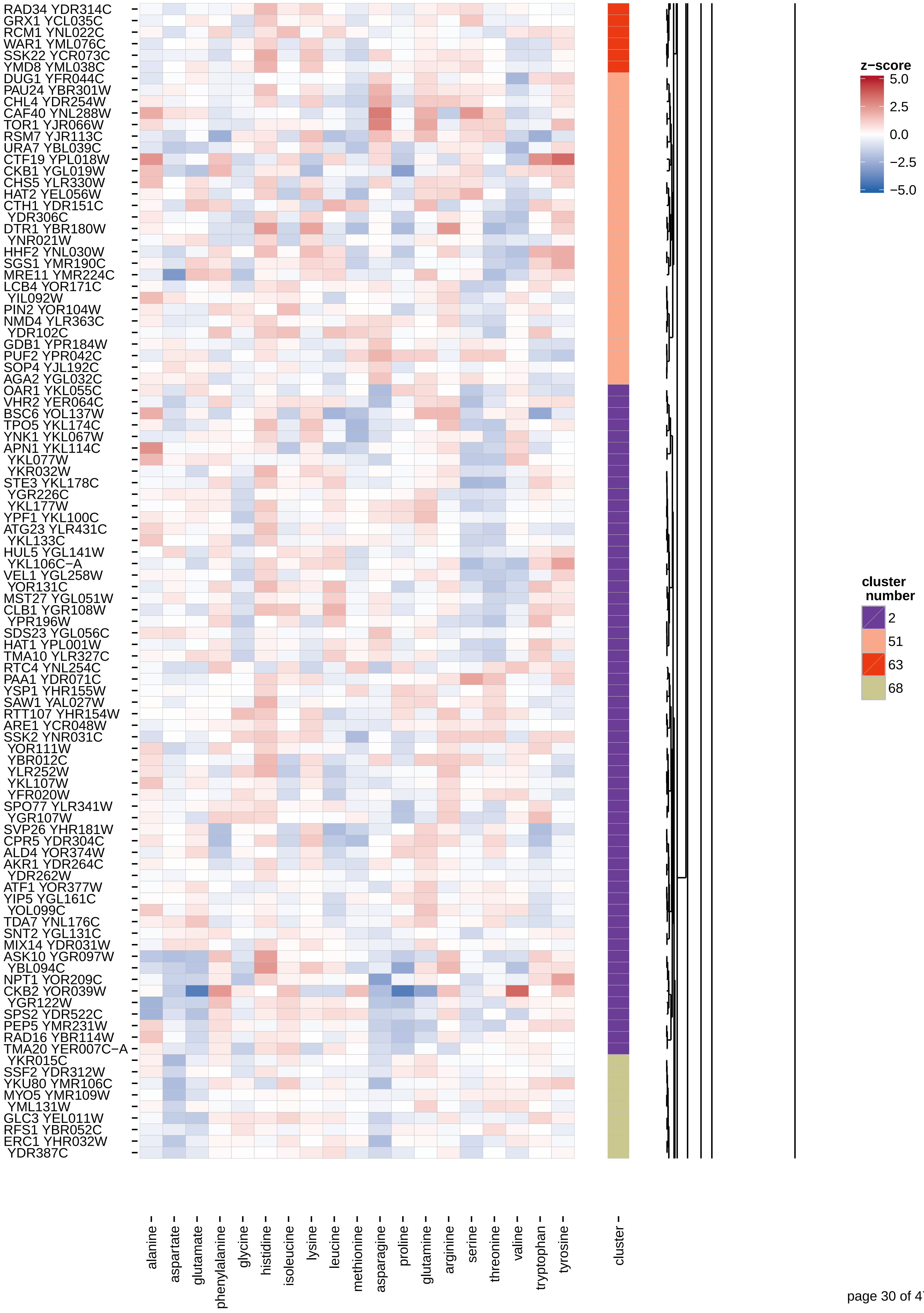
RAD34 YDR314C
GRX1 YCL035C
RCM1 YNL022C
WAR1 YML076C
SSK22 YCR073C
YMD8 YML038C
DUG1 YFR044C
PAU24 YBR301W
CHL4 YDR254W
CAF40 YNL288W
TOR1 YJR066W
RSM7 YJR113C
URA7 YBL039C
CTF19 YPL018W
CKB1 YGL019W
CHS5 YLR330W
HAT2 YEL056W
CTH1 YDR151C
YDR306C
DTR1 YBR180W
YNR021W
HHF2 YNL030W
SGS1 YMR190C
MRE11 YMR224C
LCB4 YOR171C
YIL092W
PIN2 YOR104W
NMD4 YLR363C
YDR102C
GDB1 YPR184W
PUF2 YPR042C
SOP4 YJL192C
AGA2 YGL032C
OAR1 YKL055C
VHR2 YER064C
BSC6 YOL137W
TPO5 YKL174C
YNK1 YKL067W
APN1 YKL114C
YKL077W
YKR032W
STE3 YKL178C
YGR226C
YKL177W
YPF1 YKL100C
ATG23 YLR431C
YKL133C
HUL5 YGL141W
YKL106C−A
VEL1 YGL258W
YOR131C
MST27 YGL051W
CLB1 YGR108W
YPR196W
SDS23 YGL056C
HAT1 YPL001W
TMA10 YLR327C
RTC4 YNL254C
PAA1 YDR071C
YSP1 YHR155W
SAW1 YAL027W
RTT107 YHR154W
ARE1 YCR048W
SSK2 YNR031C
YOR111W
YBR012C
YLR252W
YKL107W
YFR020W
SPO77 YLR341W
YGR107W
SVP26 YHR181W
CPR5 YDR304C
ALD4 YOR374W
AKR1 YDR264C
YDR262W
ATF1 YOR377W
YIP5 YGL161C
YOL099C
TDA7 YNL176C
SNT2 YGL131C
MIX14 YDR031W
ASK10 YGR097W
YBL094C
NPT1 YOR209C
CKB2 YOR039W
YGR122W
SPS2 YDR522C
PEP5 YMR231W
RAD16 YBR114W
TMA20 YER007C−A
YKR015C
SSF2 YDR312W
YKU80 YMR106C
MYO5 YMR109W
YML131W
GLC3 YEL011W
RFS1 YBR052C
ERC1 YHR032W
YDR387C
alanine
aspartate
glutamate
phenylalanine
glycine
histidine
isoleucine
lysine
leucine
methionine
asparagine
proline
glutamine
arginine
serine
threonine
valine
tryptophan
tyrosine
cluster
z−score
5.0
2.5
0.0
−2.5
−5.0
cluster number
2
51
63
68

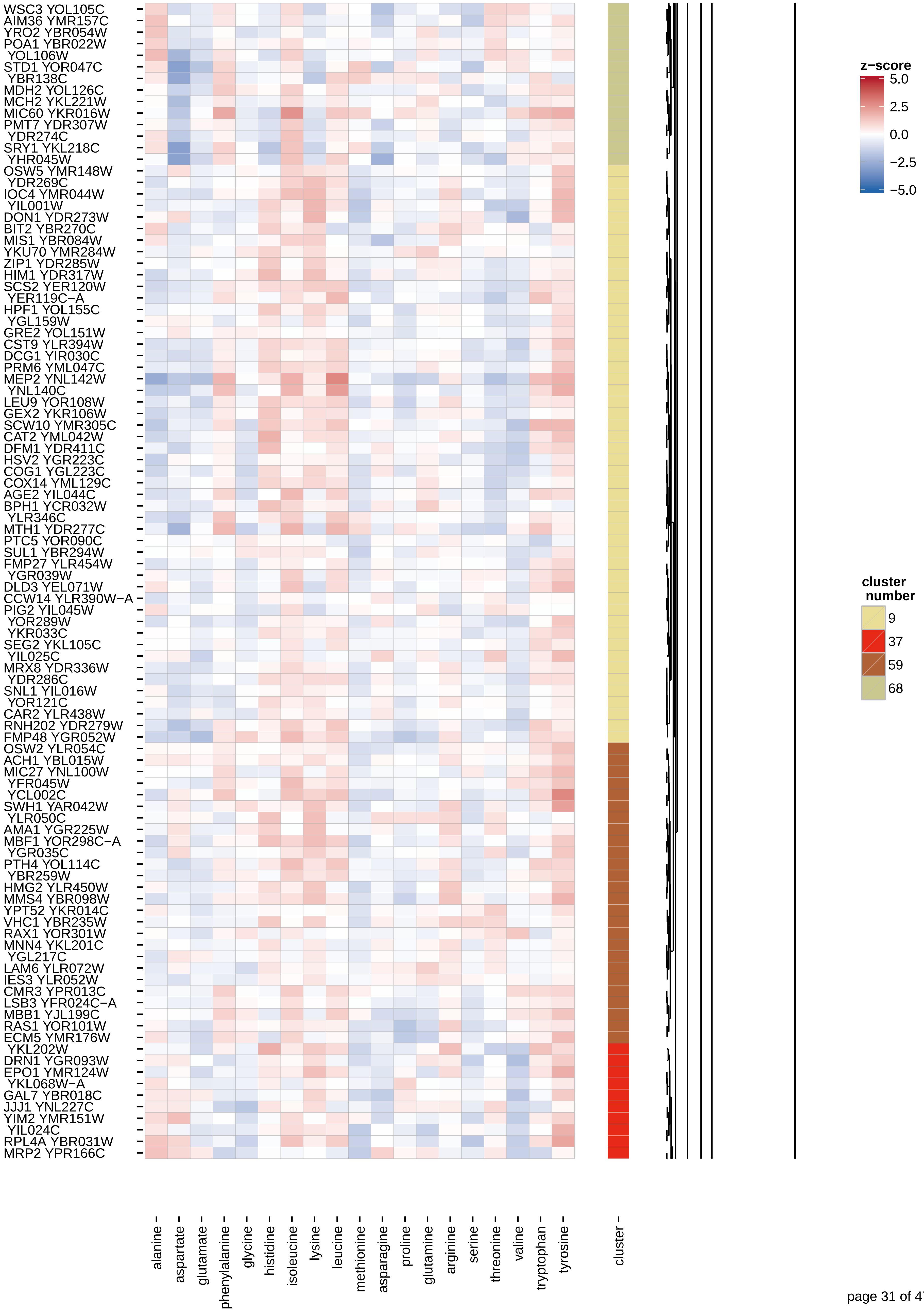
WSC3 YOL105C
AIM36 YMR157C
YRO2 YBR054W
POA1 YBR022W
YOL106W
STD1 YOR047C
YBR138C
MDH2 YOL126C
MCH2 YKL221W
MIC60 YKR016W
PMT7 YDR307W
YDR274C
SRY1 YKL218C
YHR045W
OSW5 YMR148W
YDR269C
IOC4 YMR044W
YIL001W
DON1 YDR273W
BIT2 YBR270C
MIS1 YBR084W
YKU70 YMR284W
ZIP1 YDR285W
HIM1 YDR317W
SCS2 YER120W
YER119C−A
HPF1 YOL155C
YGL159W
GRE2 YOL151W
CST9 YLR394W
DCG1 YIR030C
PRM6 YML047C
MEP2 YNL142W
YNL140C
LEU9 YOR108W
GEX2 YKR106W
SCW10 YMR305C
CAT2 YML042W
DFM1 YDR411C
HSV2 YGR223C
COG1 YGL223C
COX14 YML129C
AGE2 YIL044C
BPH1 YCR032W
YLR346C
MTH1 YDR277C
PTC5 YOR090C
SUL1 YBR294W
FMP27 YLR454W
YGR039W
DLD3 YEL071W
CCW14 YLR390W−A
PIG2 YIL045W
YOR289W
YKR033C
SEG2 YKL105C
YIL025C
MRX8 YDR336W
YDR286C
SNL1 YIL016W
YOR121C
CAR2 YLR438W
RNH202 YDR279W
FMP48 YGR052W
OSW2 YLR054C
ACH1 YBL015W
MIC27 YNL100W
YFR045W
YCL002C
SWH1 YAR042W
YLR050C
AMA1 YGR225W
MBF1 YOR298C−A
YGR035C
PTH4 YOL114C
YBR259W
HMG2 YLR450W
MMS4 YBR098W
YPT52 YKR014C
VHC1 YBR235W
RAX1 YOR301W
MNN4 YKL201C
YGL217C
LAM6 YLR072W
IES3 YLR052W
CMR3 YPR013C
LSB3 YFR024C−A
MBB1 YJL199C
RAS1 YOR101W
ECM5 YMR176W
YKL202W
DRN1 YGR093W
EPO1 YMR124W
YKL068W−A
GAL7 YBR018C
JJJ1 YNL227C
YIM2 YMR151W
YIL024C
RPL4A YBR031W
MRP2 YPR166C
alanine
aspartate
glutamate
phenylalanine
glycine
histidine
isoleucine
lysine
leucine
methionine
asparagine
proline
glutamine
arginine
serine
threonine
valine
tryptophan
tyrosine
cluster
z−score
5.0
2.5
0.0
−2.5
−5.0
cluster number
9
37
59
68

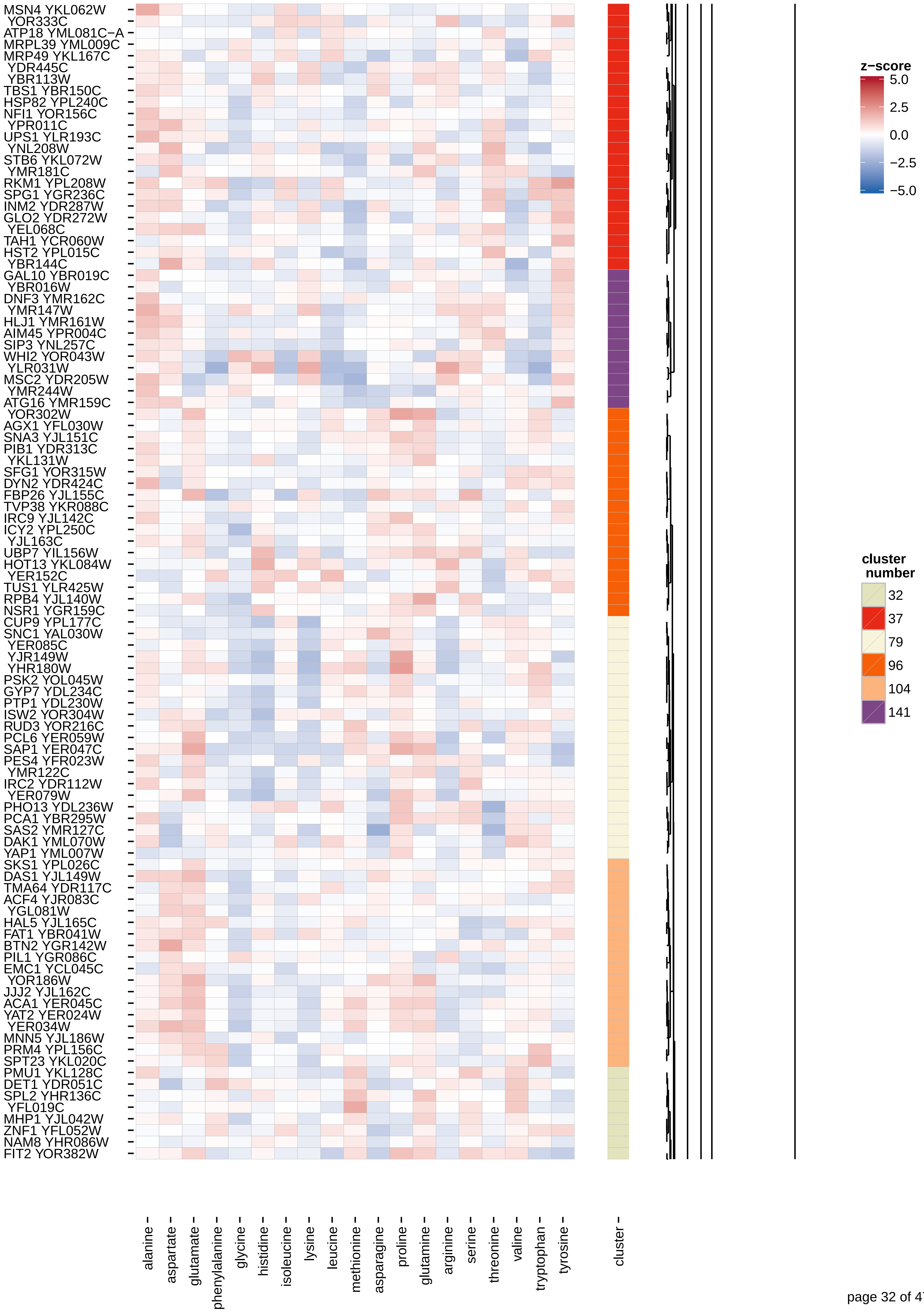
MSN4 YKL062W
YOR333C
ATP18 YML081C−A
MRPL39 YML009C
MRP49 YKL167C
YDR445C
YBR113W
TBS1 YBR150C
HSP82 YPL240C
NFI1 YOR156C
YPR011C
UPS1 YLR193C
YNL208W
STB6 YKL072W
YMR181C
RKM1 YPL208W
SPG1 YGR236C
INM2 YDR287W
GLO2 YDR272W
YEL068C
TAH1 YCR060W
HST2 YPL015C
YBR144C
GAL10 YBR019C
YBR016W
DNF3 YMR162C
YMR147W
HLJ1 YMR161W
AIM45 YPR004C
SIP3 YNL257C
WHI2 YOR043W
YLR031W
MSC2 YDR205W
YMR244W
ATG16 YMR159C
YOR302W
AGX1 YFL030W
SNA3 YJL151C
PIB1 YDR313C
YKL131W
SFG1 YOR315W
DYN2 YDR424C
FBP26 YJL155C
TVP38 YKR088C
IRC9 YJL142C
ICY2 YPL250C
YJL163C
UBP7 YIL156W
HOT13 YKL084W
YER152C
TUS1 YLR425W
RPB4 YJL140W
NSR1 YGR159C
CUP9 YPL177C
SNC1 YAL030W
YER085C
YJR149W
YHR180W
PSK2 YOL045W
GYP7 YDL234C
PTP1 YDL230W
ISW2 YOR304W
RUD3 YOR216C
PCL6 YER059W
SAP1 YER047C
PES4 YFR023W
YMR122C
IRC2 YDR112W
YER079W
PHO13 YDL236W
PCA1 YBR295W
SAS2 YMR127C
DAK1 YML070W
YAP1 YML007W
SKS1 YPL026C
DAS1 YJL149W
TMA64 YDR117C
ACF4 YJR083C
YGL081W
HAL5 YJL165C
FAT1 YBR041W
BTN2 YGR142W
PIL1 YGR086C
EMC1 YCL045C
YOR186W
JJJ2 YJL162C
ACA1 YER045C
YAT2 YER024W
YER034W
MNN5 YJL186W
PRM4 YPL156C
SPT23 YKL020C
PMU1 YKL128C
DET1 YDR051C
SPL2 YHR136C
YFL019C
MHP1 YJL042W
ZNF1 YFL052W
NAM8 YHR086W
FIT2 YOR382W
alanine
aspartate
glutamate
phenylalanine
glycine
histidine
isoleucine
lysine
leucine
methionine
asparagine
proline
glutamine
arginine
serine
threonine
valine
tryptophan
tyrosine
cluster
z−score
5.0
2.5
0.0
−2.5
−5.0
cluster number
32
37
79
96
104
141

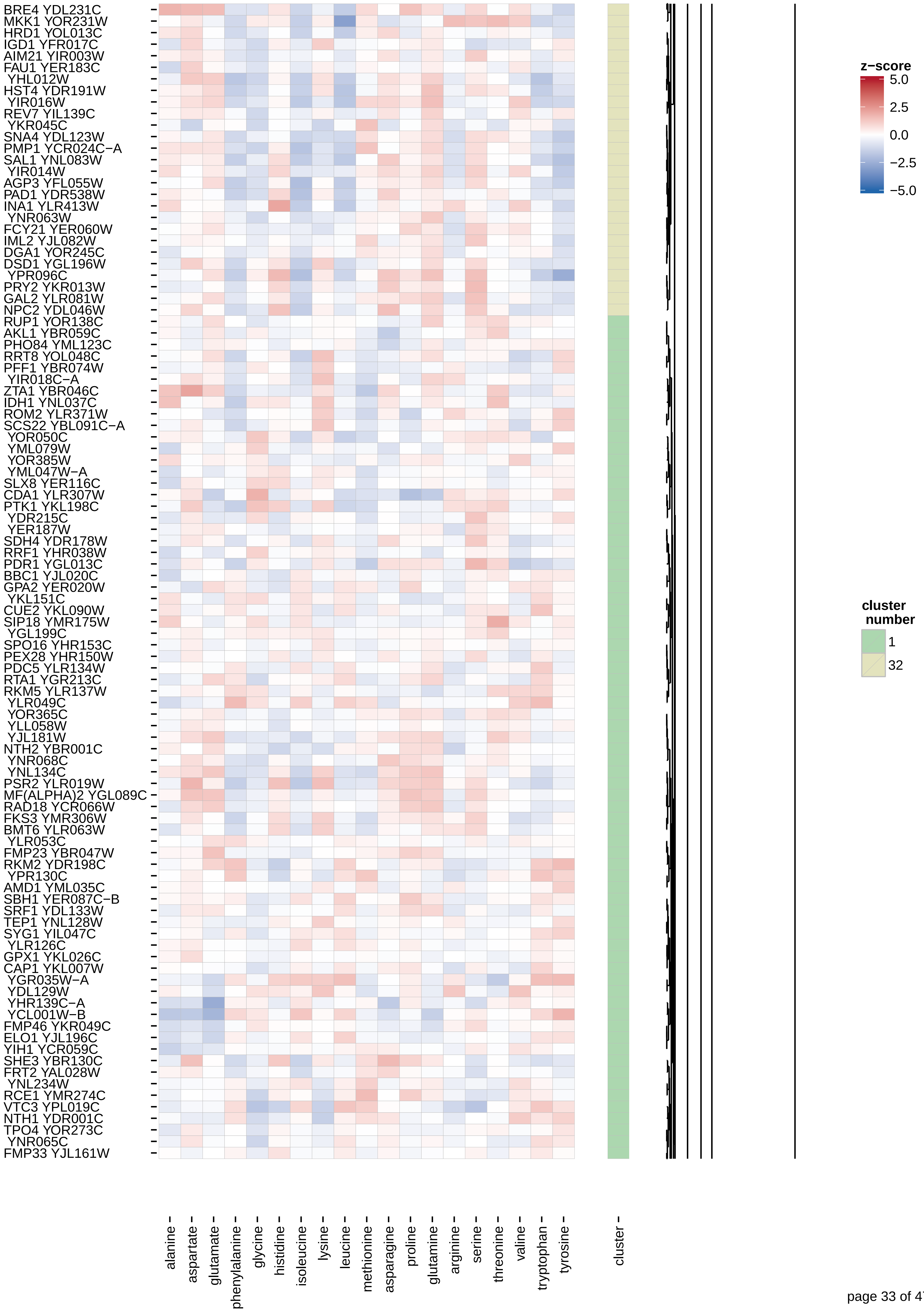
BRE4 YDL231C
MKK1 YOR231W
HRD1 YOL013C
IGD1 YFR017C
AIM21 YIR003W
FAU1 YER183C
YHL012W
HST4 YDR191W
YIR016W
REV7 YIL139C
YKR045C
SNA4 YDL123W
PMP1 YCR024C−A
SAL1 YNL083W
YIR014W
AGP3 YFL055W
PAD1 YDR538W
INA1 YLR413W
YNR063W
FCY21 YER060W
IML2 YJL082W
DGA1 YOR245C
DSD1 YGL196W
YPR096C
PRY2 YKR013W
GAL2 YLR081W
NPC2 YDL046W
RUP1 YOR138C
AKL1 YBR059C
PHO84 YML123C
RRT8 YOL048C
PFF1 YBR074W
YIR018C−A
ZTA1 YBR046C
IDH1 YNL037C
ROM2 YLR371W
SCS22 YBL091C−A
YOR050C
YML079W
YOR385W
YML047W−A
SLX8 YER116C
CDA1 YLR307W
PTK1 YKL198C
YDR215C
YER187W
SDH4 YDR178W
RRF1 YHR038W
PDR1 YGL013C
BBC1 YJL020C
GPA2 YER020W
YKL151C
CUE2 YKL090W
SIP18 YMR175W
YGL199C
SPO16 YHR153C
PEX28 YHR150W
PDC5 YLR134W
RTA1 YGR213C
RKM5 YLR137W
YLR049C
YOR365C
YLL058W
YJL181W
NTH2 YBR001C
YNR068C
YNL134C
PSR2 YLR019W
MF(ALPHA)2 YGL089C
RAD18 YCR066W
FKS3 YMR306W
BMT6 YLR063W
YLR053C
FMP23 YBR047W
RKM2 YDR198C
YPR130C
AMD1 YML035C
SBH1 YER087C−B
SRF1 YDL133W
TEP1 YNL128W
SYG1 YIL047C
YLR126C
GPX1 YKL026C
CAP1 YKL007W
YGR035W−A
YDL129W
YHR139C−A
YCL001W−B
FMP46 YKR049C
ELO1 YJL196C
YIH1 YCR059C
SHE3 YBR130C
FRT2 YAL028W
YNL234W
RCE1 YMR274C
VTC3 YPL019C
NTH1 YDR001C
TPO4 YOR273C
YNR065C
FMP33 YJL161W
alanine
aspartate
glutamate
phenylalanine
glycine
histidine
isoleucine
lysine
leucine
methionine
asparagine
proline
glutamine
arginine
serine
threonine
valine
tryptophan
tyrosine
cluster
z−score
5.0
2.5
0.0
−2.5
−5.0
cluster number
1
32

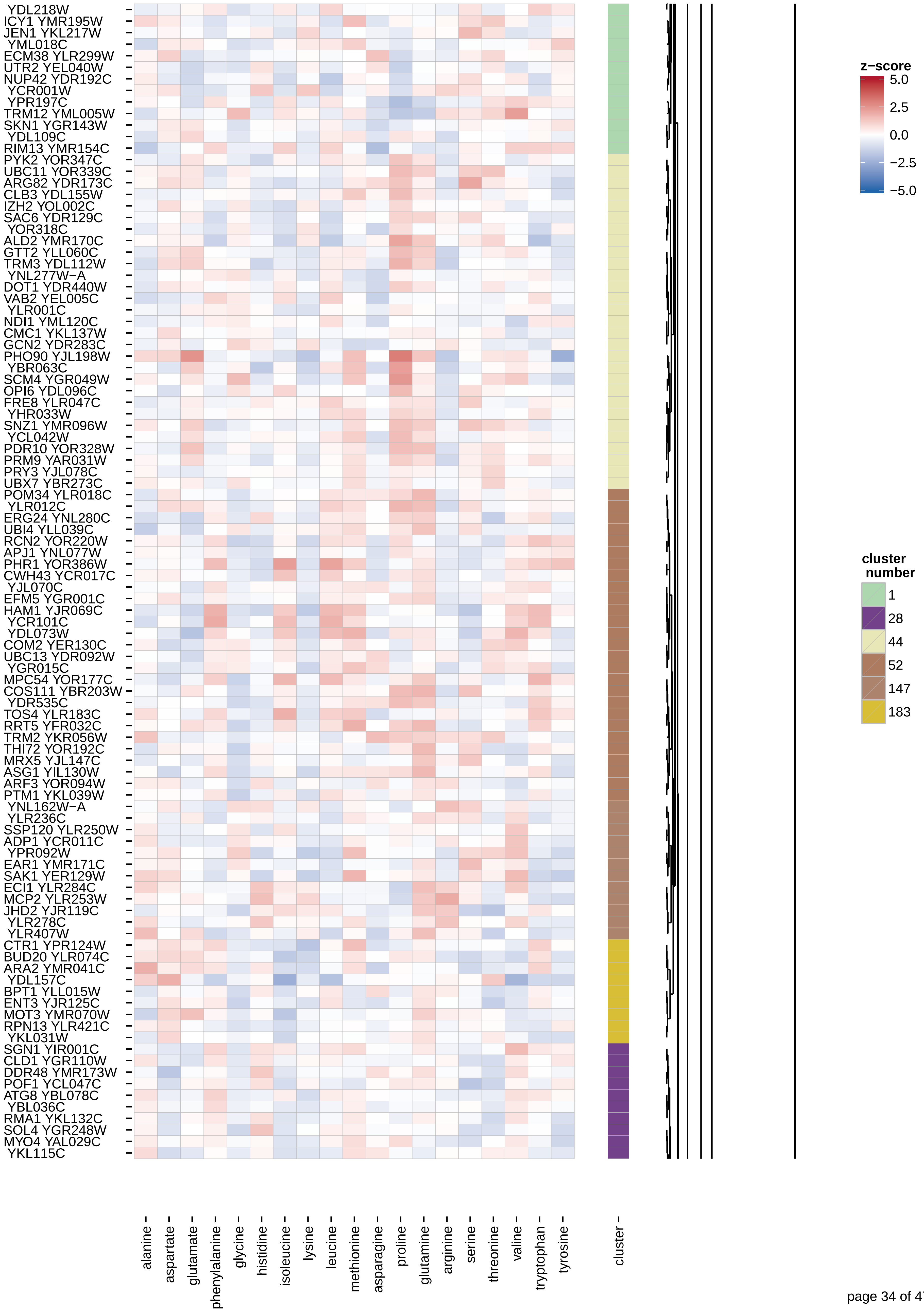

YDL218W
ICY1 YMR195W
JEN1 YKL217W
YML018C
ECM38 YLR299W
UTR2 YEL040W
NUP42 YDR192C
YCR001W
YPR197C
TRM12 YML005W
SKN1 YGR143W
YDL109C
RIM13 YMR154C
PYK2 YOR347C
UBC11 YOR339C
ARG82 YDR173C
CLB3 YDL155W
IZH2 YOL002C
SAC6 YDR129C
YOR318C
ALD2 YMR170C
GTT2 YLL060C
TRM3 YDL112W
YNL277W−A
DOT1 YDR440W
VAB2 YEL005C
YLR001C
NDI1 YML120C
CMC1 YKL137W
GCN2 YDR283C
PHO90 YJL198W
YBR063C
SCM4 YGR049W
OPI6 YDL096C
FRE8 YLR047C
YHR033W
SNZ1 YMR096W
YCL042W
PDR10 YOR328W
PRM9 YAR031W
PRY3 YJL078C
UBX7 YBR273C
POM34 YLR018C
YLR012C
ERG24 YNL280C
UBI4 YLL039C
RCN2 YOR220W
APJ1 YNL077W
PHR1 YOR386W
CWH43 YCR017C
YJL070C
EFM5 YGR001C
HAM1 YJR069C
YCR101C
YDL073W
COM2 YER130C
UBC13 YDR092W
YGR015C
MPC54 YOR177C
COS111 YBR203W
YDR535C
TOS4 YLR183C
RRT5 YFR032C
TRM2 YKR056W
THI72 YOR192C
MRX5 YJL147C
ASG1 YIL130W
ARF3 YOR094W
PTM1 YKL039W
YNL162W−A
YLR236C
SSP120 YLR250W
ADP1 YCR011C
YPR092W
EAR1 YMR171C
SAK1 YER129W
ECI1 YLR284C
MCP2 YLR253W
JHD2 YJR119C
YLR278C
YLR407W
CTR1 YPR124W
BUD20 YLR074C
ARA2 YMR041C
YDL157C
BPT1 YLL015W
ENT3 YJR125C
MOT3 YMR070W
RPN13 YLR421C
YKL031W
SGN1 YIR001C
CLD1 YGR110W
DDR48 YMR173W
POF1 YCL047C
ATG8 YBL078C
YBL036C
RMA1 YKL132C
SOL4 YGR248W
MYO4 YAL029C
YKL115C
alanine
aspartate
glutamate
phenylalanine
glycine
histidine
isoleucine
lysine
leucine
methionine
asparagine
proline
glutamine
arginine
serine
threonine
valine
tryptophan
tyrosine
cluster
z−score
5.0
2.5
0.0
−2.5
−5.0
cluster number
1
28
44
52
147
183

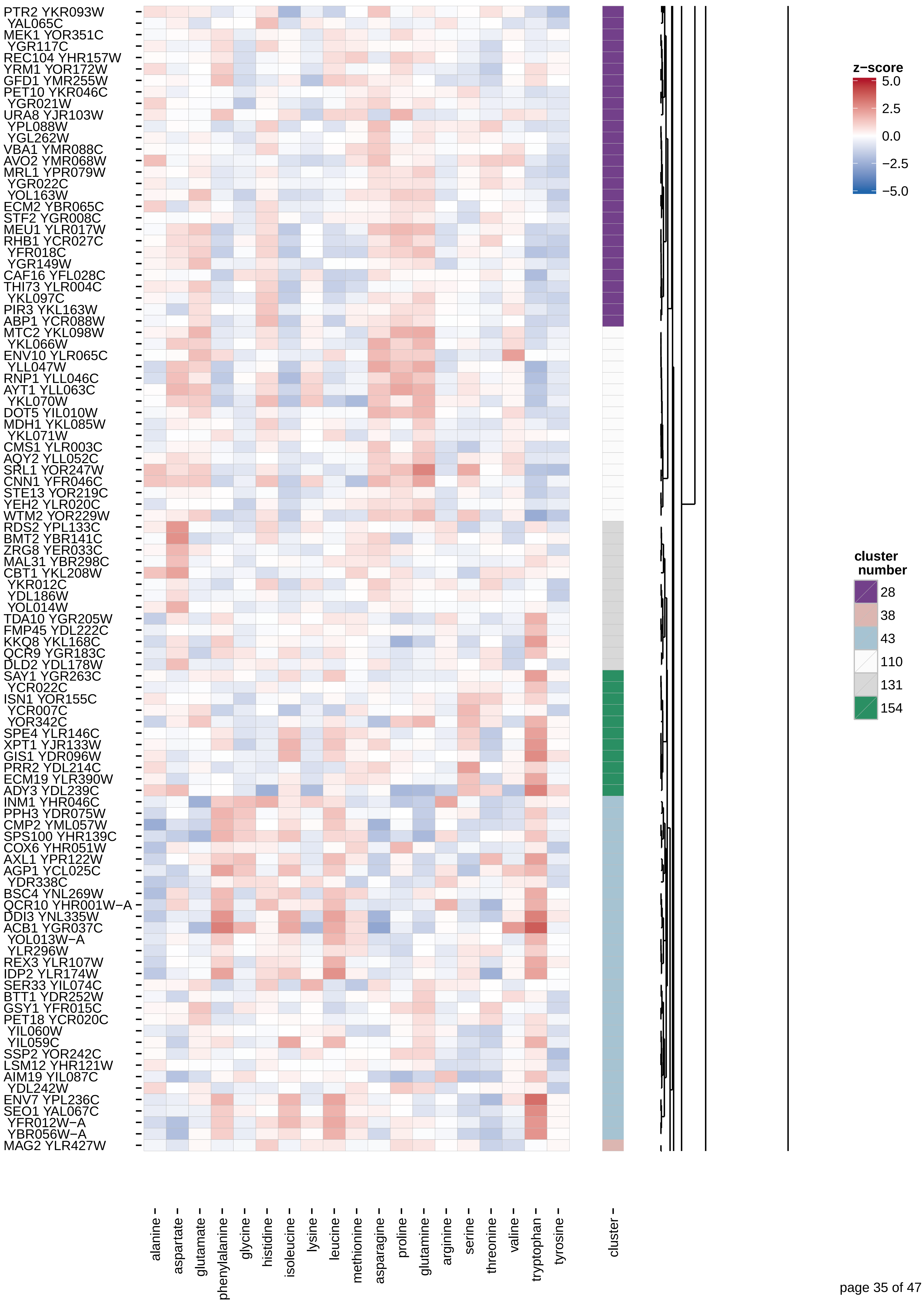
PTR2 YKR093W
YAL065C
MEK1 YOR351C
YGR117C
REC104 YHR157W
YRM1 YOR172W
GFD1 YMR255W
PET10 YKR046C
YGR021W
URA8 YJR103W
YPL088W
YGL262W
VBA1 YMR088C
AVO2 YMR068W
MRL1 YPR079W
YGR022C
YOL163W
ECM2 YBR065C
STF2 YGR008C
MEU1 YLR017W
RHB1 YCR027C
YFR018C
YGR149W
CAF16 YFL028C
THI73 YLR004C
YKL097C
PIR3 YKL163W
ABP1 YCR088W
MTC2 YKL098W
YKL066W
ENV10 YLR065C
YLL047W
RNP1 YLL046C
AYT1 YLL063C
YKL070W
DOT5 YIL010W
MDH1 YKL085W
YKL071W
CMS1 YLR003C
AQY2 YLL052C
SRL1 YOR247W
CNN1 YFR046C
STE13 YOR219C
YEH2 YLR020C
WTM2 YOR229W
RDS2 YPL133C
BMT2 YBR141C
ZRG8 YER033C
MAL31 YBR298C
CBT1 YKL208W
YKR012C
YDL186W
YOL014W
TDA10 YGR205W
FMP45 YDL222C
KKQ8 YKL168C
QCR9 YGR183C
DLD2 YDL178W
SAY1 YGR263C
YCR022C
ISN1 YOR155C
YCR007C
YOR342C
SPE4 YLR146C
XPT1 YJR133W
GIS1 YDR096W
PRR2 YDL214C
ECM19 YLR390W
ADY3 YDL239C
INM1 YHR046C
PPH3 YDR075W
CMP2 YML057W
SPS100 YHR139C
COX6 YHR051W
AXL1 YPR122W
AGP1 YCL025C
YDR338C
BSC4 YNL269W
QCR10 YHR001W−A
DDI3 YNL335W
ACB1 YGR037C
YOL013W−A
YLR296W
REX3 YLR107W
IDP2 YLR174W
SER33 YIL074C
BTT1 YDR252W
GSY1 YFR015C
PET18 YCR020C
YIL060W
YIL059C
SSP2 YOR242C
LSM12 YHR121W
AIM19 YIL087C
YDL242W
ENV7 YPL236C
SEO1 YAL067C
YFR012W−A
YBR056W−A
MAG2 YLR427W
alanine
aspartate
glutamate
phenylalanine
glycine
histidine
isoleucine
lysine
leucine
methionine
asparagine
proline
glutamine
arginine
serine
threonine
valine
tryptophan
tyrosine
cluster
z−score
5.0
2.5
0.0
−2.5
−5.0
cluster number
28
38
43
110
131
154

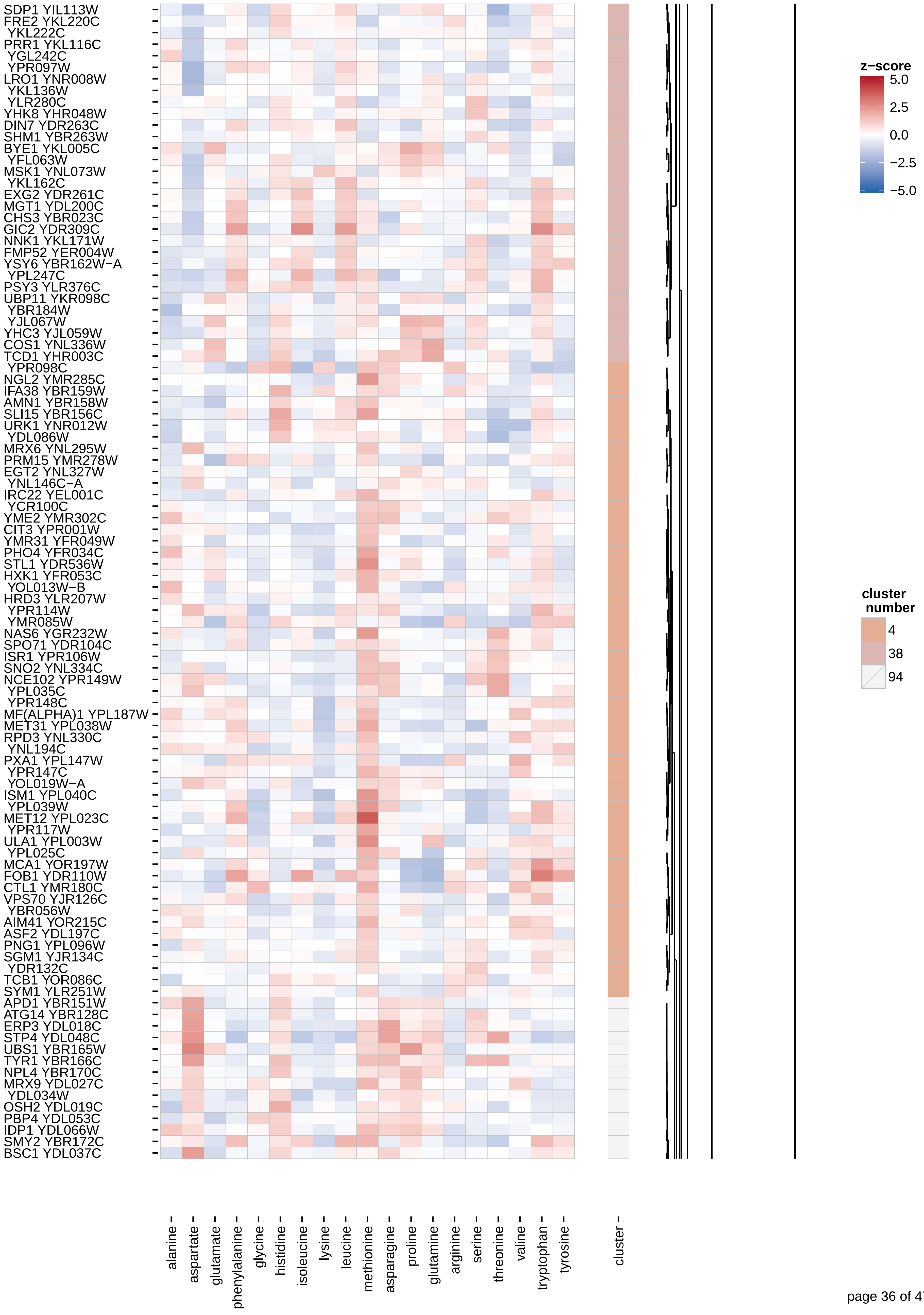
SDP1 YIL113W
FRE2 YKL220C
YKL222C
PRR1 YKL116C
YGL242C
YPR097W
LRO1 YNR008W
YKL136W
YLR280C
YHK8 YHR048W
DIN7 YDR263C
SHM1 YBR263W
BYE1 YKL005C
YFL063W
MSK1 YNL073W
YKL162C
EXG2 YDR261C
MGT1 YDL200C
CHS3 YBR023C
GIC2 YDR309C
NNK1 YKL171W
FMP52 YER004W
YSY6 YBR162W−A
YPL247C
PSY3 YLR376C
UBP11 YKR098C
YBR184W
YJL067W
YHC3 YJL059W
COS1 YNL336W
TCD1 YHR003C
YPR098C
NGL2 YMR285C
IFA38 YBR159W
AMN1 YBR158W
SLI15 YBR156C
URK1 YNR012W
YDL086W
MRX6 YNL295W
PRM15 YMR278W
EGT2 YNL327W
YNL146C−A
IRC22 YEL001C
YCR100C
YME2 YMR302C
CIT3 YPR001W
YMR31 YFR049W
PHO4 YFR034C
STL1 YDR536W
HXK1 YFR053C
YOL013W−B
HRD3 YLR207W
YPR114W
YMR085W
NAS6 YGR232W
SPO71 YDR104C
ISR1 YPR106W
SNO2 YNL334C
NCE102 YPR149W
YPL035C
YPR148C
MF(ALPHA)1 YPL187W
MET31 YPL038W
RPD3 YNL330C
YNL194C
PXA1 YPL147W
YPR147C
YOL019W−A
ISM1 YPL040C
YPL039W
MET12 YPL023C
YPR117W
ULA1 YPL003W
YPL025C
MCA1 YOR197W
FOB1 YDR110W
CTL1 YMR180C
VPS70 YJR126C
YBR056W
AIM41 YOR215C
ASF2 YDL197C
PNG1 YPL096W
SGM1 YJR134C
YDR132C
TCB1 YOR086C
SYM1 YLR251W
APD1 YBR151W
ATG14 YBR128C
ERP3 YDL018C
STP4 YDL048C
UBS1 YBR165W
TYR1 YBR166C
NPL4 YBR170C
MRX9 YDL027C
YDL034W
OSH2 YDL019C
PBP4 YDL053C
IDP1 YDL066W
SMY2 YBR172C
BSC1 YDL037C
alanine
aspartate
glutamate
phenylalanine
glycine
histidine
isoleucine
lysine
leucine
methionine
asparagine
proline
glutamine
arginine
serine
threonine
valine
tryptophan
tyrosine
cluster
z−score
5.0
2.5
0.0
−2.5
−5.0
cluster number
4
38
94

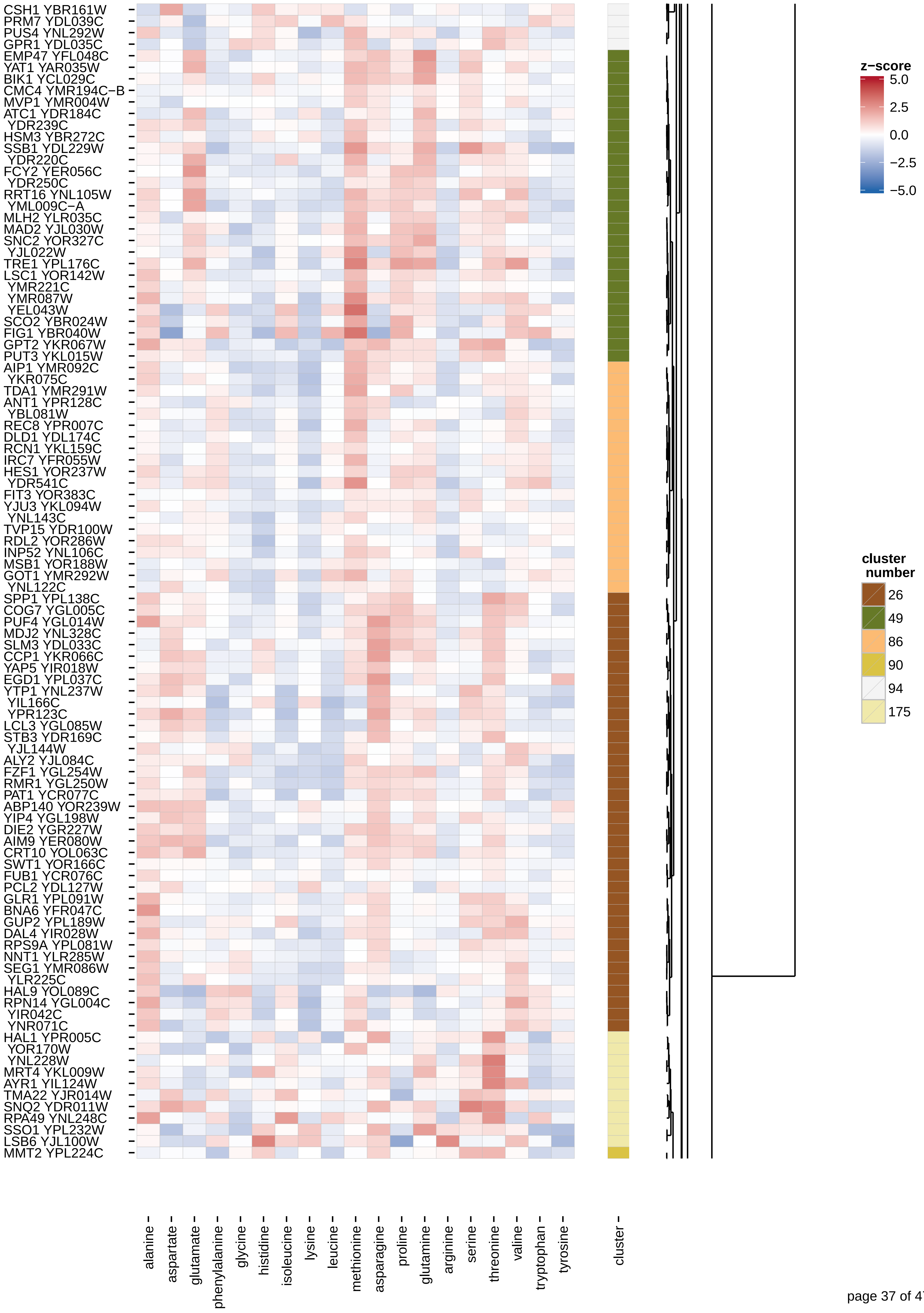
CSH1 YBR161W
PRM7 YDL039C
PUS4 YNL292W
GPR1 YDL035C
EMP47 YFL048C
YAT1 YAR035W
BIK1 YCL029C
CMC4 YMR194C−B
MVP1 YMR004W
ATC1 YDR184C
YDR239C
HSM3 YBR272C
SSB1 YDL229W
YDR220C
FCY2 YER056C
YDR250C
RRT16 YNL105W
YML009C−A
MLH2 YLR035C
MAD2 YJL030W
SNC2 YOR327C
YJL022W
TRE1 YPL176C
LSC1 YOR142W
YMR221C
YMR087W
YEL043W
SCO2 YBR024W
FIG1 YBR040W
GPT2 YKR067W
PUT3 YKL015W
AIP1 YMR092C
YKR075C
TDA1 YMR291W
ANT1 YPR128C
YBL081W
REC8 YPR007C
DLD1 YDL174C
RCN1 YKL159C
IRC7 YFR055W
HES1 YOR237W
YDR541C
FIT3 YOR383C
YJU3 YKL094W
YNL143C
TVP15 YDR100W
RDL2 YOR286W
INP52 YNL106C
MSB1 YOR188W
GOT1 YMR292W
YNL122C
SPP1 YPL138C
COG7 YGL005C
PUF4 YGL014W
MDJ2 YNL328C
SLM3 YDL033C
CCP1 YKR066C
YAP5 YIR018W
EGD1 YPL037C
YTP1 YNL237W
YIL166C
YPR123C
LCL3 YGL085W
STB3 YDR169C
YJL144W
ALY2 YJL084C
FZF1 YGL254W
RMR1 YGL250W
PAT1 YCR077C
ABP140 YOR239W
YIP4 YGL198W
DIE2 YGR227W
AIM9 YER080W
CRT10 YOL063C
SWT1 YOR166C
FUB1 YCR076C
PCL2 YDL127W
GLR1 YPL091W
BNA6 YFR047C
GUP2 YPL189W
DAL4 YIR028W
RPS9A YPL081W
NNT1 YLR285W
SEG1 YMR086W
YLR225C
HAL9 YOL089C
RPN14 YGL004C
YIR042C
YNR071C
HAL1 YPR005C
YOR170W
YNL228W
MRT4 YKL009W
AYR1 YIL124W
TMA22 YJR014W
SNQ2 YDR011W
RPA49 YNL248C
SSO1 YPL232W
LSB6 YJL100W
MMT2 YPL224C
alanine
aspartate
glutamate
phenylalanine
glycine
histidine
isoleucine
lysine
leucine
methionine
asparagine
proline
glutamine
arginine
serine
threonine
valine
tryptophan
tyrosine
cluster
z−score
5.0
2.5
0.0
−2.5
−5.0
cluster number
26
49
86
90
94
175

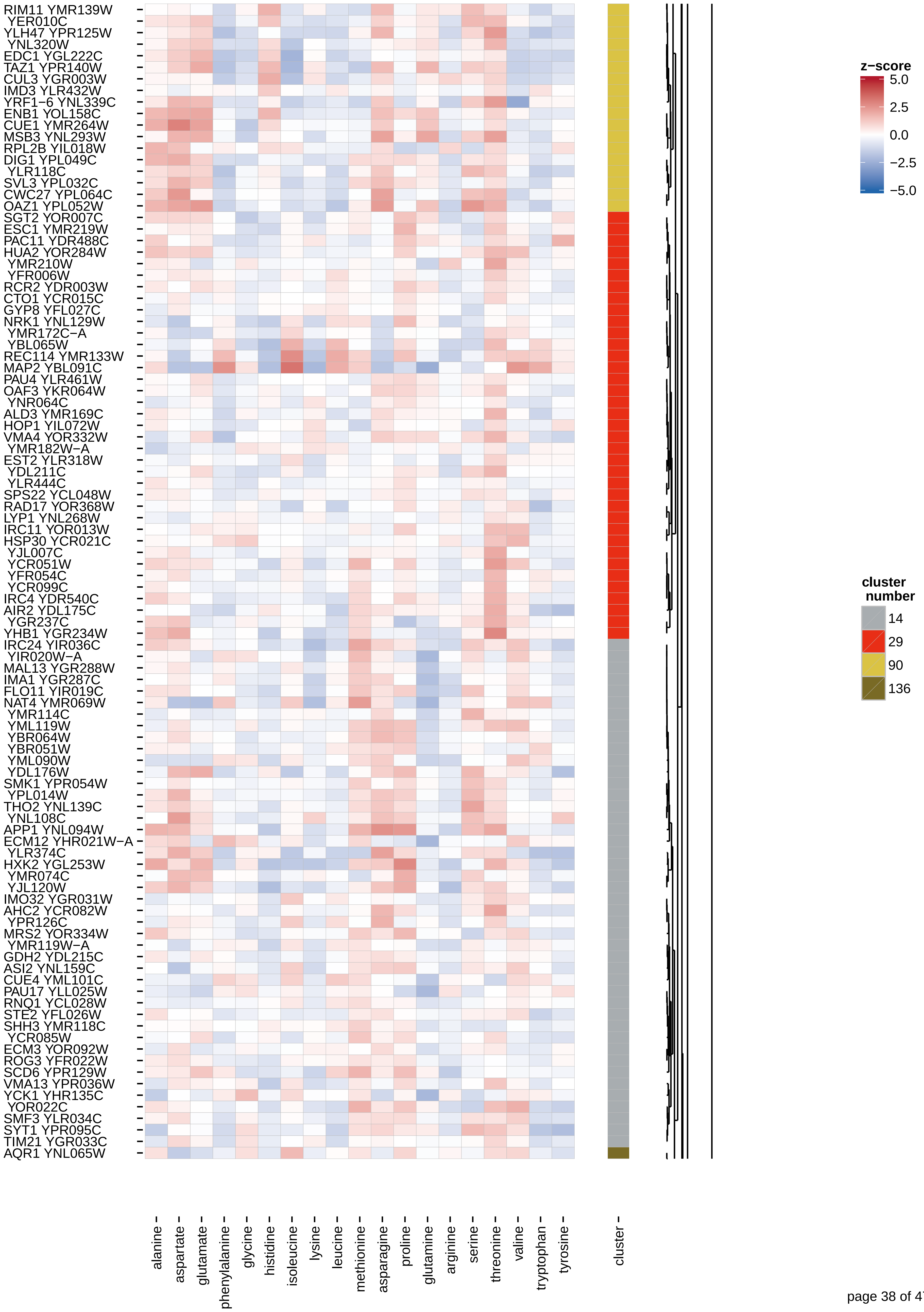
RIM11 YMR139W
YER010C
YLH47 YPR125W
YNL320W
EDC1 YGL222C
TAZ1 YPR140W
CUL3 YGR003W
IMD3 YLR432W
YRF1−6 YNL339C
ENB1 YOL158C
CUE1 YMR264W
MSB3 YNL293W
RPL2B YIL018W
DIG1 YPL049C
YLR118C
SVL3 YPL032C
CWC27 YPL064C
OAZ1 YPL052W
SGT2 YOR007C
ESC1 YMR219W
PAC11 YDR488C
HUA2 YOR284W
YMR210W
YFR006W
RCR2 YDR003W
CTO1 YCR015C
GYP8 YFL027C
NRK1 YNL129W
YMR172C−A
YBL065W
REC114 YMR133W
MAP2 YBL091C
PAU4 YLR461W
OAF3 YKR064W
YNR064C
ALD3 YMR169C
HOP1 YIL072W
VMA4 YOR332W
YMR182W−A
EST2 YLR318W
YDL211C
YLR444C
SPS22 YCL048W
RAD17 YOR368W
LYP1 YNL268W
IRC11 YOR013W
HSP30 YCR021C
YJL007C
YCR051W
YFR054C
YCR099C
IRC4 YDR540C
AIR2 YDL175C
YGR237C
YHB1 YGR234W
IRC24 YIR036C
YIR020W−A
MAL13 YGR288W
IMA1 YGR287C
FLO11 YIR019C
NAT4 YMR069W
YMR114C
YML119W
YBR064W
YBR051W
YML090W
YDL176W
SMK1 YPR054W
YPL014W
THO2 YNL139C
YNL108C
APP1 YNL094W
ECM12 YHR021W−A
YLR374C
HXK2 YGL253W
YMR074C
YJL120W
IMO32 YGR031W
AHC2 YCR082W
YPR126C
MRS2 YOR334W
YMR119W−A
GDH2 YDL215C
ASI2 YNL159C
CUE4 YML101C
PAU17 YLL025W
RNQ1 YCL028W
STE2 YFL026W
SHH3 YMR118C
YCR085W
ECM3 YOR092W
ROG3 YFR022W
SCD6 YPR129W
VMA13 YPR036W
YCK1 YHR135C
YOR022C
SMF3 YLR034C
SYT1 YPR095C
TIM21 YGR033C
AQR1 YNL065W
alanine
aspartate
glutamate
phenylalanine
glycine
histidine
isoleucine
lysine
leucine
methionine
asparagine
proline
glutamine
arginine
serine
threonine
valine
tryptophan
tyrosine
cluster
z−score
5.0
2.5
0.0
−2.5
−5.0
cluster number
14
29
90
136

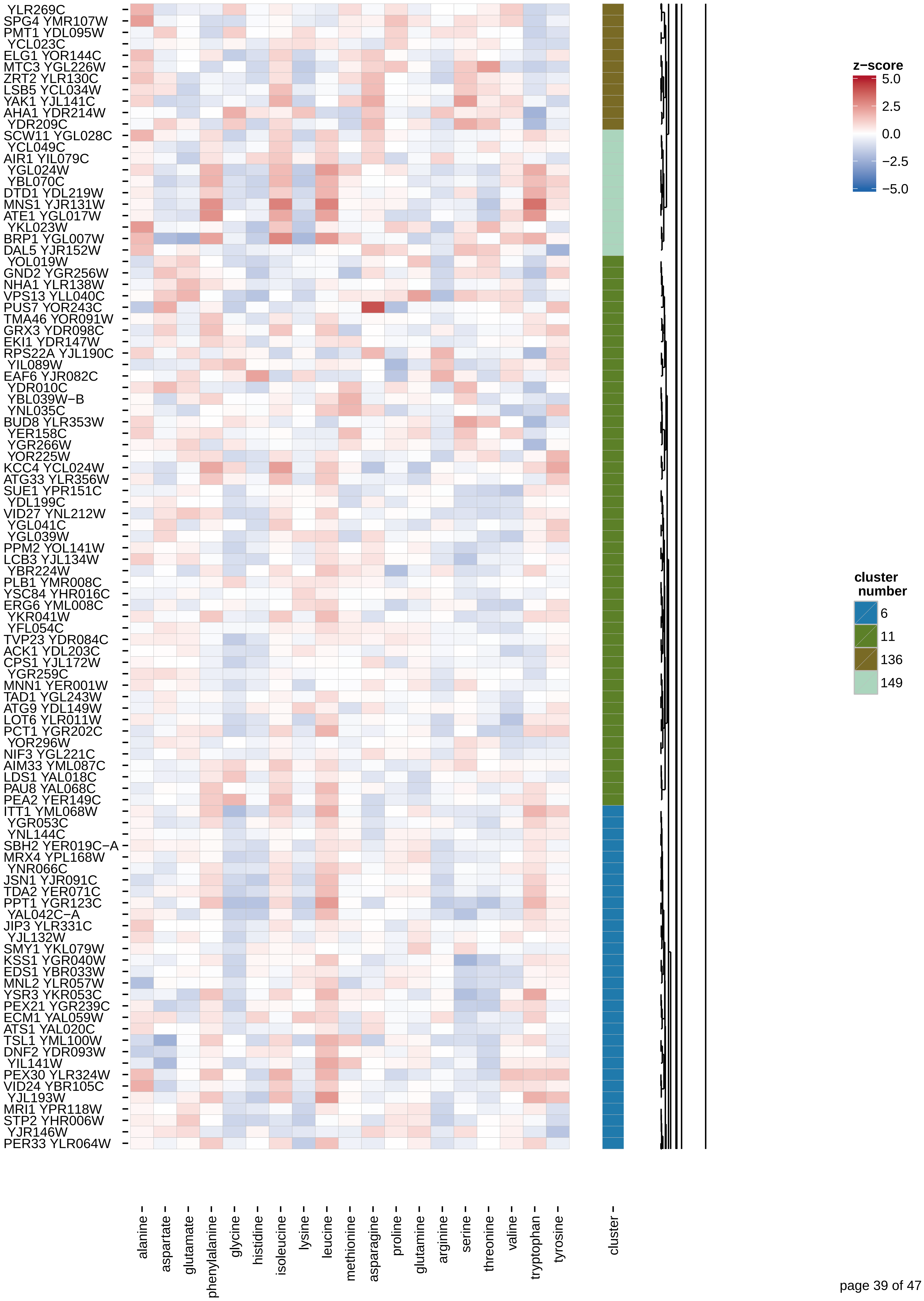
YLR269C
SPG4 YMR107W
PMT1 YDL095W
YCL023C
ELG1 YOR144C
MTC3 YGL226W
ZRT2 YLR130C
LSB5 YCL034W
YAK1 YJL141C
AHA1 YDR214W
YDR209C
SCW11 YGL028C
YCL049C
AIR1 YIL079C
YGL024W
YBL070C
DTD1 YDL219W
MNS1 YJR131W
ATE1 YGL017W
YKL023W
BRP1 YGL007W
DAL5 YJR152W
YOL019W
GND2 YGR256W
NHA1 YLR138W
VPS13 YLL040C
PUS7 YOR243C
TMA46 YOR091W
GRX3 YDR098C
EKI1 YDR147W
RPS22A YJL190C
YIL089W
EAF6 YJR082C
YDR010C
YBL039W−B
YNL035C
BUD8 YLR353W
YER158C
YGR266W
YOR225W
KCC4 YCL024W
ATG33 YLR356W
SUE1 YPR151C
YDL199C
VID27 YNL212W
YGL041C
YGL039W
PPM2 YOL141W
LCB3 YJL134W
YBR224W
PLB1 YMR008C
YSC84 YHR016C
ERG6 YML008C
YKR041W
YFL054C
TVP23 YDR084C
ACK1 YDL203C
CPS1 YJL172W
YGR259C
MNN1 YER001W
TAD1 YGL243W
ATG9 YDL149W
LOT6 YLR011W
PCT1 YGR202C
YOR296W
NIF3 YGL221C
AIM33 YML087C
LDS1 YAL018C
PAU8 YAL068C
PEA2 YER149C
ITT1 YML068W
YGR053C
YNL144C
SBH2 YER019C−A
MRX4 YPL168W
YNR066C
JSN1 YJR091C
TDA2 YER071C
PPT1 YGR123C
YAL042C−A
JIP3 YLR331C
YJL132W
SMY1 YKL079W
KSS1 YGR040W
EDS1 YBR033W
MNL2 YLR057W
YSR3 YKR053C
PEX21 YGR239C
ECM1 YAL059W
ATS1 YAL020C
TSL1 YML100W
DNF2 YDR093W
YIL141W
PEX30 YLR324W
VID24 YBR105C
YJL193W
MRI1 YPR118W
STP2 YHR006W
YJR146W
PER33 YLR064W
alanine
aspartate
glutamate
phenylalanine
glycine
histidine
isoleucine
lysine
leucine
methionine
asparagine
proline
glutamine
arginine
serine
threonine
valine
tryptophan
tyrosine
cluster
z−score
5.0
2.5
0.0
−2.5
−5.0
cluster number
6
11
136
149

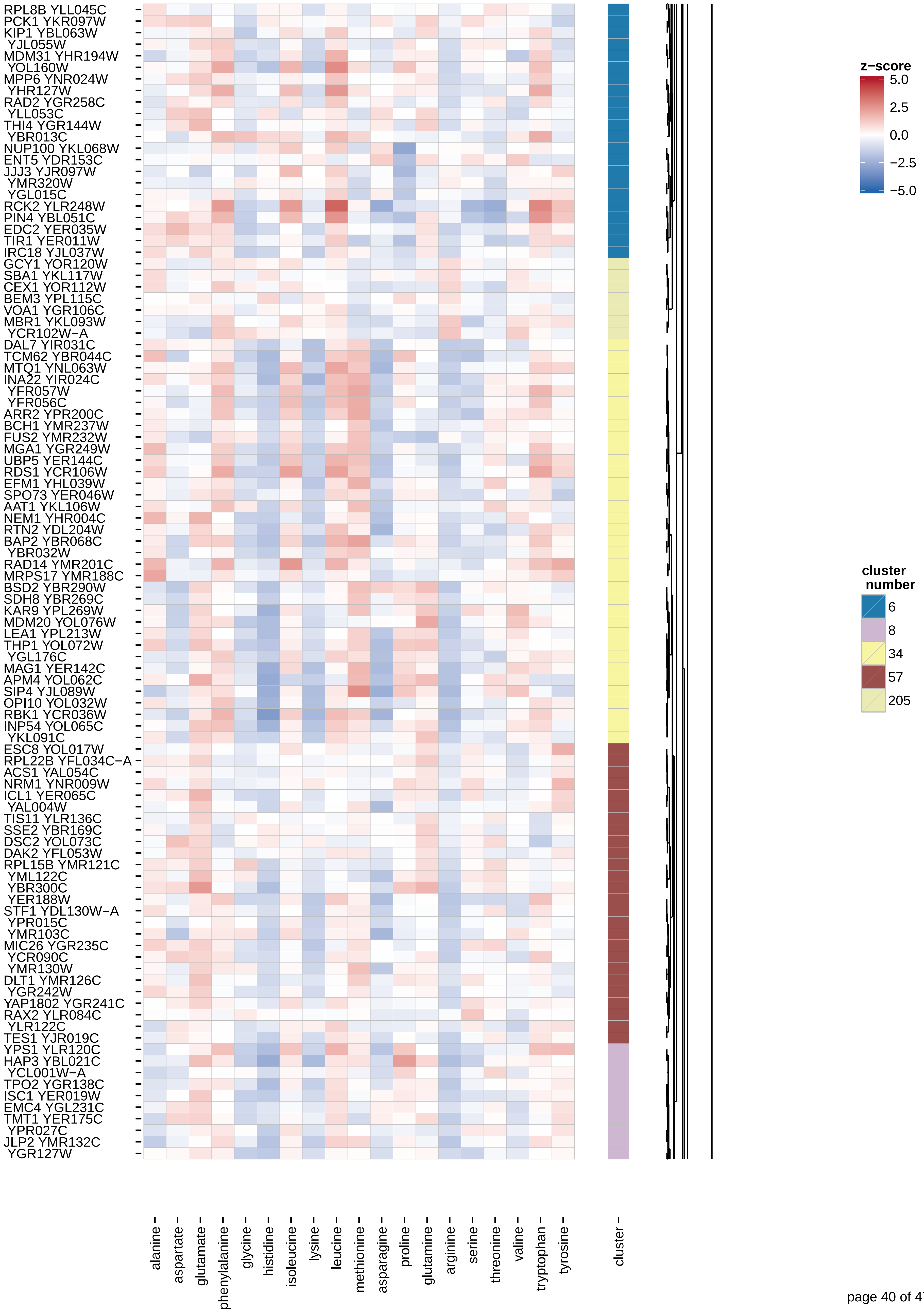
RPL8B YLL045C
PCK1 YKR097W
KIP1 YBL063W
YJL055W
MDM31 YHR194W
YOL160W
MPP6 YNR024W
YHR127W
RAD2 YGR258C
YLL053C
THI4 YGR144W
YBR013C
NUP100 YKL068W
ENT5 YDR153C
JJJ3 YJR097W
YMR320W
YGL015C
RCK2 YLR248W
PIN4 YBL051C
EDC2 YER035W
TIR1 YER011W
IRC18 YJL037W
GCY1 YOR120W
SBA1 YKL117W
CEX1 YOR112W
BEM3 YPL115C
VOA1 YGR106C
MBR1 YKL093W
YCR102W−A
DAL7 YIR031C
TCM62 YBR044C
MTQ1 YNL063W
INA22 YIR024C
YFR057W
YFR056C
ARR2 YPR200C
BCH1 YMR237W
FUS2 YMR232W
MGA1 YGR249W
UBP5 YER144C
RDS1 YCR106W
EFM1 YHL039W
SPO73 YER046W
AAT1 YKL106W
NEM1 YHR004C
RTN2 YDL204W
BAP2 YBR068C
YBR032W
RAD14 YMR201C
MRPS17 YMR188C
BSD2 YBR290W
SDH8 YBR269C
KAR9 YPL269W
MDM20 YOL076W
LEA1 YPL213W
THP1 YOL072W
YGL176C
MAG1 YER142C
APM4 YOL062C
SIP4 YJL089W
OPI10 YOL032W
RBK1 YCR036W
INP54 YOL065C
YKL091C
ESC8 YOL017W
RPL22B YFL034C−A
ACS1 YAL054C
NRM1 YNR009W
ICL1 YER065C
YAL004W
TIS11 YLR136C
SSE2 YBR169C
DSC2 YOL073C
DAK2 YFL053W
RPL15B YMR121C
YML122C
YBR300C
YER188W
STF1 YDL130W−A
YPR015C
YMR103C
MIC26 YGR235C
YCR090C
YMR130W
DLT1 YMR126C
YGR242W
YAP1802 YGR241C
RAX2 YLR084C
YLR122C
TES1 YJR019C
YPS1 YLR120C
HAP3 YBL021C
YCL001W−A
TPO2 YGR138C
ISC1 YER019W
EMC4 YGL231C
TMT1 YER175C
YPR027C
JLP2 YMR132C
YGR127W
alanine
aspartate
glutamate
phenylalanine
glycine
histidine
isoleucine
lysine
leucine
methionine
asparagine
proline
glutamine
arginine
serine
threonine
valine
tryptophan
tyrosine
cluster
z−score
5.0
2.5
0.0
−2.5
−5.0
cluster
number
6
8
34
57
205

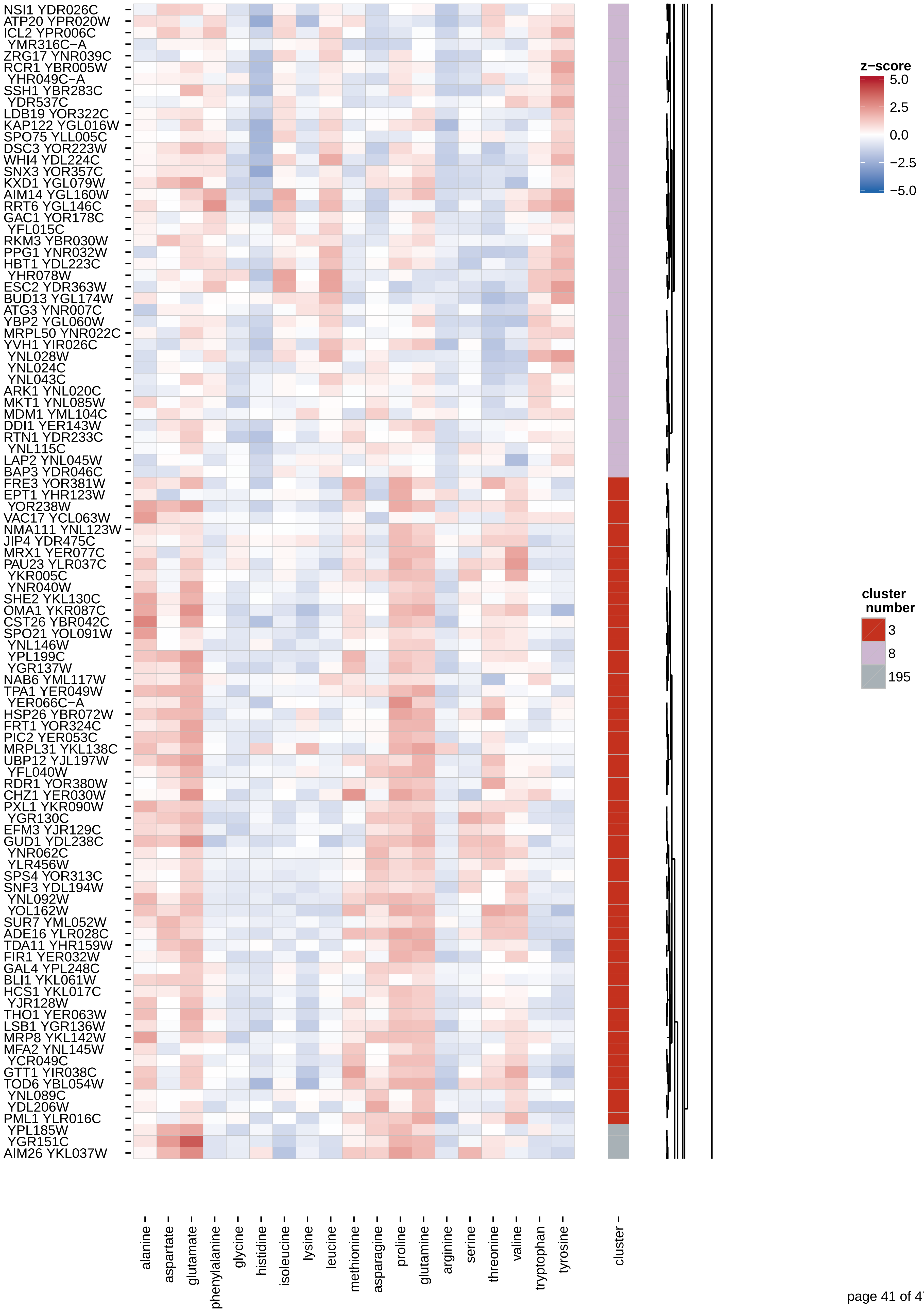
NSI1 YDR026C
ATP20 YPR020W
ICL2 YPR006C
YMR316C−A
ZRG17 YNR039C
RCR1 YBR005W
YHR049C−A
SSH1 YBR283C
YDR537C
LDB19 YOR322C
KAP122 YGL016W
SPO75 YLL005C
DSC3 YOR223W
WHI4 YDL224C
SNX3 YOR357C
KXD1 YGL079W
AIM14 YGL160W
RRT6 YGL146C
GAC1 YOR178C
YFL015C
RKM3 YBR030W
PPG1 YNR032W
HBT1 YDL223C
YHR078W
ESC2 YDR363W
BUD13 YGL174W
ATG3 YNR007C
YBP2 YGL060W
MRPL50 YNR022C
YVH1 YIR026C
YNL028W
YNL024C
YNL043C
ARK1 YNL020C
MKT1 YNL085W
MDM1 YML104C
DDI1 YER143W
RTN1 YDR233C
YNL115C
LAP2 YNL045W
BAP3 YDR046C
FRE3 YOR381W
EPT1 YHR123W
YOR238W
VAC17 YCL063W
NMA111 YNL123W
JIP4 YDR475C
MRX1 YER077C
PAU23 YLR037C
YKR005C
YNR040W
SHE2 YKL130C
OMA1 YKR087C
CST26 YBR042C
SPO21 YOL091W
YNL146W
YPL199C
YGR137W
NAB6 YML117W
TPA1 YER049W
YER066C−A
HSP26 YBR072W
FRT1 YOR324C
PIC2 YER053C
MRPL31 YKL138C
UBP12 YJL197W
YFL040W
RDR1 YOR380W
CHZ1 YER030W
PXL1 YKR090W
YGR130C
EFM3 YJR129C
GUD1 YDL238C
YNR062C
YLR456W
SPS4 YOR313C
SNF3 YDL194W
YNL092W
YOL162W
SUR7 YML052W
ADE16 YLR028C
TDA11 YHR159W
FIR1 YER032W
GAL4 YPL248C
BLI1 YKL061W
HCS1 YKL017C
YJR128W
THO1 YER063W
LSB1 YGR136W
MRP8 YKL142W
MFA2 YNL145W
YCR049C
GTT1 YIR038C
TOD6 YBL054W
YNL089C
YDL206W
PML1 YLR016C
YPL185W
YGR151C
AIM26 YKL037W
alanine
aspartate
glutamate
phenylalanine
glycine
histidine
isoleucine
lysine
leucine
methionine
asparagine
proline
glutamine
arginine
serine
threonine
valine
tryptophan
tyrosine
cluster
z−score
5.0
2.5
0.0
−2.5
−5.0
cluster
number
3
8
195

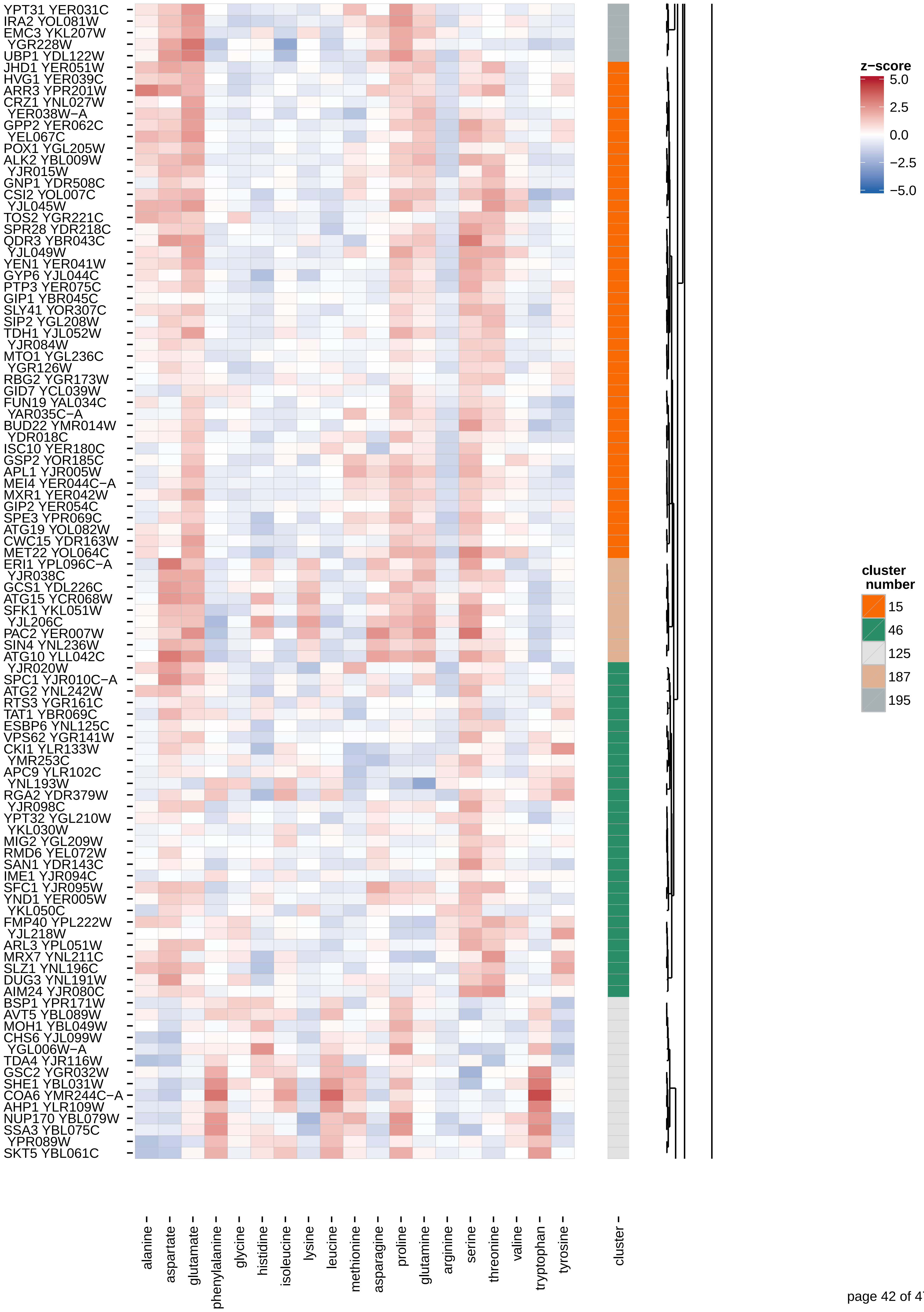
YPT31 YER031C
IRA2 YOL081W
EMC3 YKL207W
YGR228W
UBP1 YDL122W
JHD1 YER051W
HVG1 YER039C
ARR3 YPR201W
CRZ1 YNL027W
YER038W−A
GPP2 YER062C
YEL067C
POX1 YGL205W
ALK2 YBL009W
YJR015W
GNP1 YDR508C
CSI2 YOL007C
YJL045W
TOS2 YGR221C
SPR28 YDR218C
QDR3 YBR043C
YJL049W
YEN1 YER041W
GYP6 YJL044C
PTP3 YER075C
GIP1 YBR045C
SLY41 YOR307C
SIP2 YGL208W
TDH1 YJL052W
YJR084W
MTO1 YGL236C
YGR126W
RBG2 YGR173W
GID7 YCL039W
FUN19 YAL034C
YAR035C−A
BUD22 YMR014W
YDR018C
ISC10 YER180C
GSP2 YOR185C
APL1 YJR005W
MEI4 YER044C−A
MXR1 YER042W
GIP2 YER054C
SPE3 YPR069C
ATG19 YOL082W
CWC15 YDR163W
MET22 YOL064C
ERI1 YPL096C−A
YJR038C
GCS1 YDL226C
ATG15 YCR068W
SFK1 YKL051W
YJL206C
PAC2 YER007W
SIN4 YNL236W
ATG10 YLL042C
YJR020W
SPC1 YJR010C−A
ATG2 YNL242W
RTS3 YGR161C
TAT1 YBR069C
ESBP6 YNL125C
VPS62 YGR141W
CKI1 YLR133W
YMR253C
APC9 YLR102C
YNL193W
RGA2 YDR379W
YJR098C
YPT32 YGL210W
YKL030W
MIG2 YGL209W
RMD6 YEL072W
SAN1 YDR143C
IME1 YJR094C
SFC1 YJR095W
YND1 YER005W
YKL050C
FMP40 YPL222W
YJL218W
ARL3 YPL051W
MRX7 YNL211C
SLZ1 YNL196C
DUG3 YNL191W
AIM24 YJR080C
BSP1 YPR171W
AVT5 YBL089W
MOH1 YBL049W
CHS6 YJL099W
YGL006W−A
TDA4 YJR116W
GSC2 YGR032W
SHE1 YBL031W
COA6 YMR244C−A
AHP1 YLR109W
NUP170 YBL079W
SSA3 YBL075C
YPR089W
SKT5 YBL061C
alanine
aspartate
glutamate
phenylalanine
glycine
histidine
isoleucine
lysine
leucine
methionine
asparagine
proline
glutamine
arginine
serine
threonine
valine
tryptophan
tyrosine
cluster
z−score
5.0
2.5
0.0
−2.5
−5.0
cluster number
15
46
125
187
195

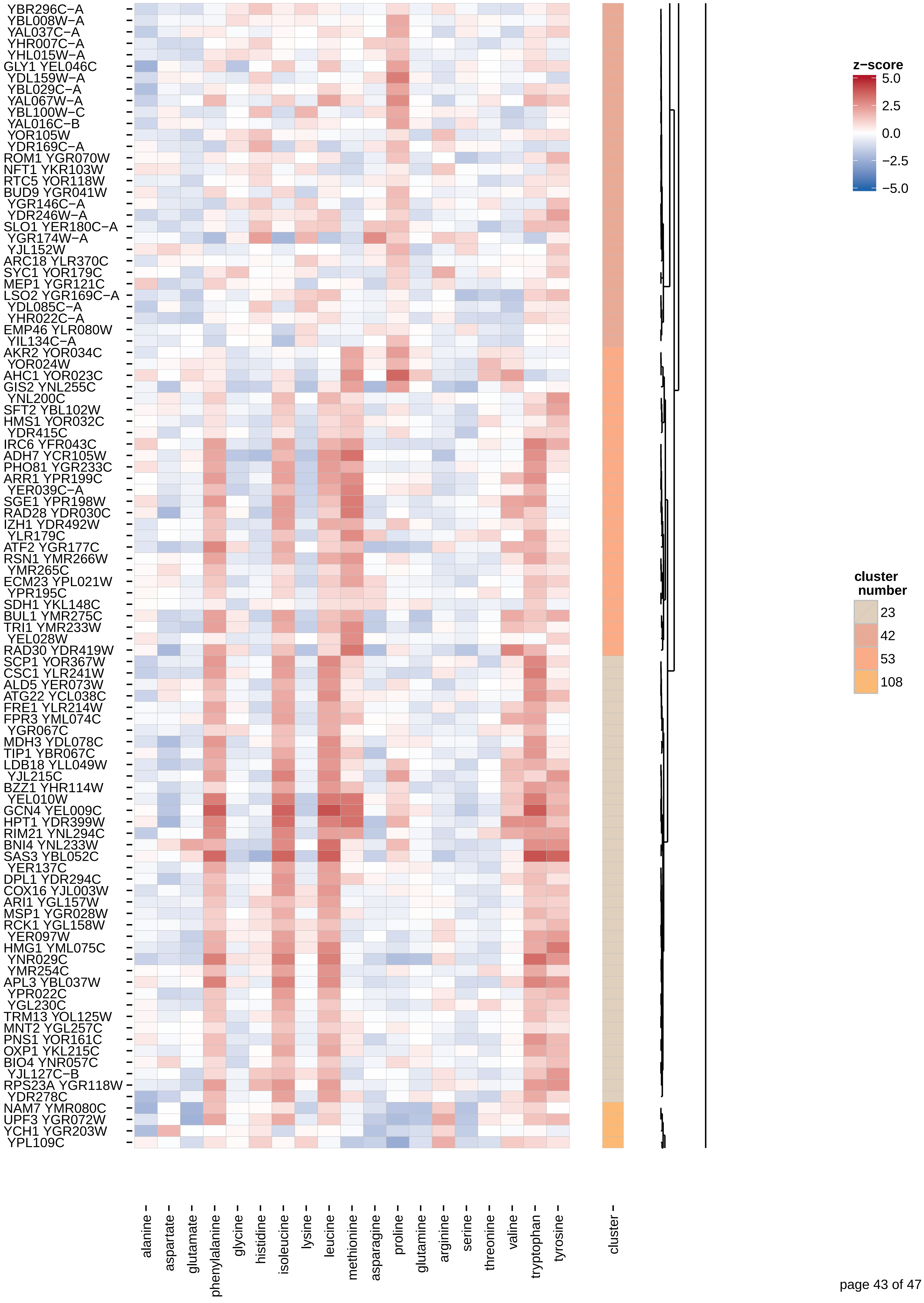
z−score
5.0
2.5
0.0
−2.5
−5.0
cluster number
23
42
53
108
YBR296C−A
YBL008W−A
YAL037C−A
YHR007C−A
YHL015W−A
GLY1 YEL046C
YDL159W−A
YBL029C−A
YAL067W−A
YBL100W−C
YAL016C−B
YOR105W
YDR169C−A
ROM1 YGR070W
NFT1 YKR103W
RTC5 YOR118W
BUD9 YGR041W
YGR146C−A
YDR246W−A
SLO1 YER180C−A
YGR174W−A
YJL152W
ARC18 YLR370C
SYC1 YOR179C
MEP1 YGR121C
LSO2 YGR169C−A
YDL085C−A
YHR022C−A
EMP46 YLR080W
YIL134C−A
AKR2 YOR034C
YOR024W
AHC1 YOR023C
GIS2 YNL255C
YNL200C
SFT2 YBL102W
HMS1 YOR032C
YDR415C
IRC6 YFR043C
ADH7 YCR105W
PHO81 YGR233C
ARR1 YPR199C
YER039C−A
SGE1 YPR198W
RAD28 YDR030C
IZH1 YDR492W
YLR179C
ATF2 YGR177C
RSN1 YMR266W
YMR265C
ECM23 YPL021W
YPR195C
SDH1 YKL148C
BUL1 YMR275C
TRI1 YMR233W
YEL028W
RAD30 YDR419W
SCP1 YOR367W
CSC1 YLR241W
ALD5 YER073W
ATG22 YCL038C
FRE1 YLR214W
FPR3 YML074C
YGR067C
MDH3 YDL078C
TIP1 YBR067C
LDB18 YLL049W
YJL215C
BZZ1 YHR114W
YEL010W
GCN4 YEL009C
HPT1 YDR399W
RIM21 YNL294C
BNI4 YNL233W
SAS3 YBL052C
YER137C
DPL1 YDR294C
COX16 YJL003W
ARI1 YGL157W
MSP1 YGR028W
RCK1 YGL158W
YER097W
HMG1 YML075C
YNR029C
YMR254C
APL3 YBL037W
YPR022C
YGL230C
TRM13 YOL125W
MNT2 YGL257C
PNS1 YOR161C
OXP1 YKL215C
BIO4 YNR057C
YJL127C−B
RPS23A YGR118W
YDR278C
NAM7 YMR080C
UPF3 YGR072W
YCH1 YGR203W
YPL109C
alanine
aspartate
glutamate
phenylalanine
glycine
histidine
isoleucine
lysine
leucine
methionine
asparagine
proline
glutamine
arginine
serine
threonine
valine
tryptophan
tyrosine
cluster

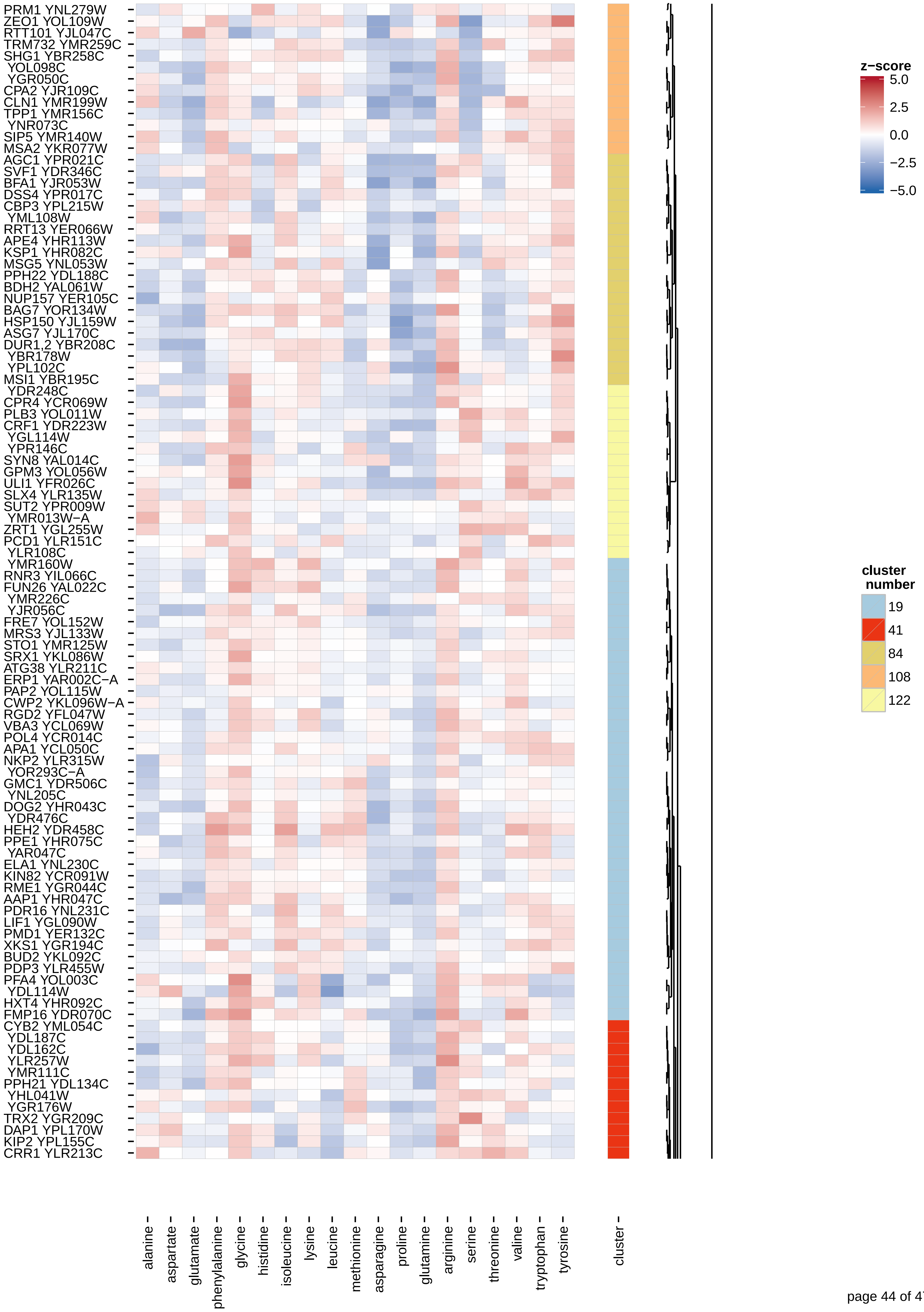
PRM1 YNL279W
ZEO1 YOL109W
RTT101 YJL047C
TRM732 YMR259C
SHG1 YBR258C
YOL098C
YGR050C
CPA2 YJR109C
CLN1 YMR199W
TPP1 YMR156C
YNR073C
SIP5 YMR140W
MSA2 YKR077W
AGC1 YPR021C
SVF1 YDR346C
BFA1 YJR053W
DSS4 YPR017C
CBP3 YPL215W
YML108W
RRT13 YER066W
APE4 YHR113W
KSP1 YHR082C
MSG5 YNL053W
PPH22 YDL188C
BDH2 YAL061W
NUP157 YER105C
BAG7 YOR134W
HSP150 YJL159W
ASG7 YJL170C
DUR1,2 YBR208C
YBR178W
YPL102C
MSI1 YBR195C
YDR248C
CPR4 YCR069W
PLB3 YOL011W
CRF1 YDR223W
YGL114W
YPR146C
SYN8 YAL014C
GPM3 YOL056W
ULI1 YFR026C
SLX4 YLR135W
SUT2 YPR009W
YMR013W−A
ZRT1 YGL255W
PCD1 YLR151C
YLR108C
YMR160W
RNR3 YIL066C
FUN26 YAL022C
YMR226C
YJR056C
FRE7 YOL152W
MRS3 YJL133W
STO1 YMR125W
SRX1 YKL086W
ATG38 YLR211C
ERP1 YAR002C−A
PAP2 YOL115W
CWP2 YKL096W−A
RGD2 YFL047W
VBA3 YCL069W
POL4 YCR014C
APA1 YCL050C
NKP2 YLR315W
YOR293C−A
GMC1 YDR506C
YNL205C
DOG2 YHR043C
YDR476C
HEH2 YDR458C
PPE1 YHR075C
YAR047C
ELA1 YNL230C
KIN82 YCR091W
RME1 YGR044C
AAP1 YHR047C
PDR16 YNL231C
LIF1 YGL090W
PMD1 YER132C
XKS1 YGR194C
BUD2 YKL092C
PDP3 YLR455W
PFA4 YOL003C
YDL114W
HXT4 YHR092C
FMP16 YDR070C
CYB2 YML054C
YDL187C
YDL162C
YLR257W
YMR111C
PPH21 YDL134C
YHL041W
YGR176W
TRX2 YGR209C
DAP1 YPL170W
KIP2 YPL155C
CRR1 YLR213C
alanine
aspartate
glutamate
phenylalanine
glycine
histidine
isoleucine
lysine
leucine
methionine
asparagine
proline
glutamine
arginine
serine
threonine
valine
tryptophan
tyrosine
cluster
z−score
5.0
2.5
0.0
−2.5
−5.0
cluster number
19
41
84
108
122

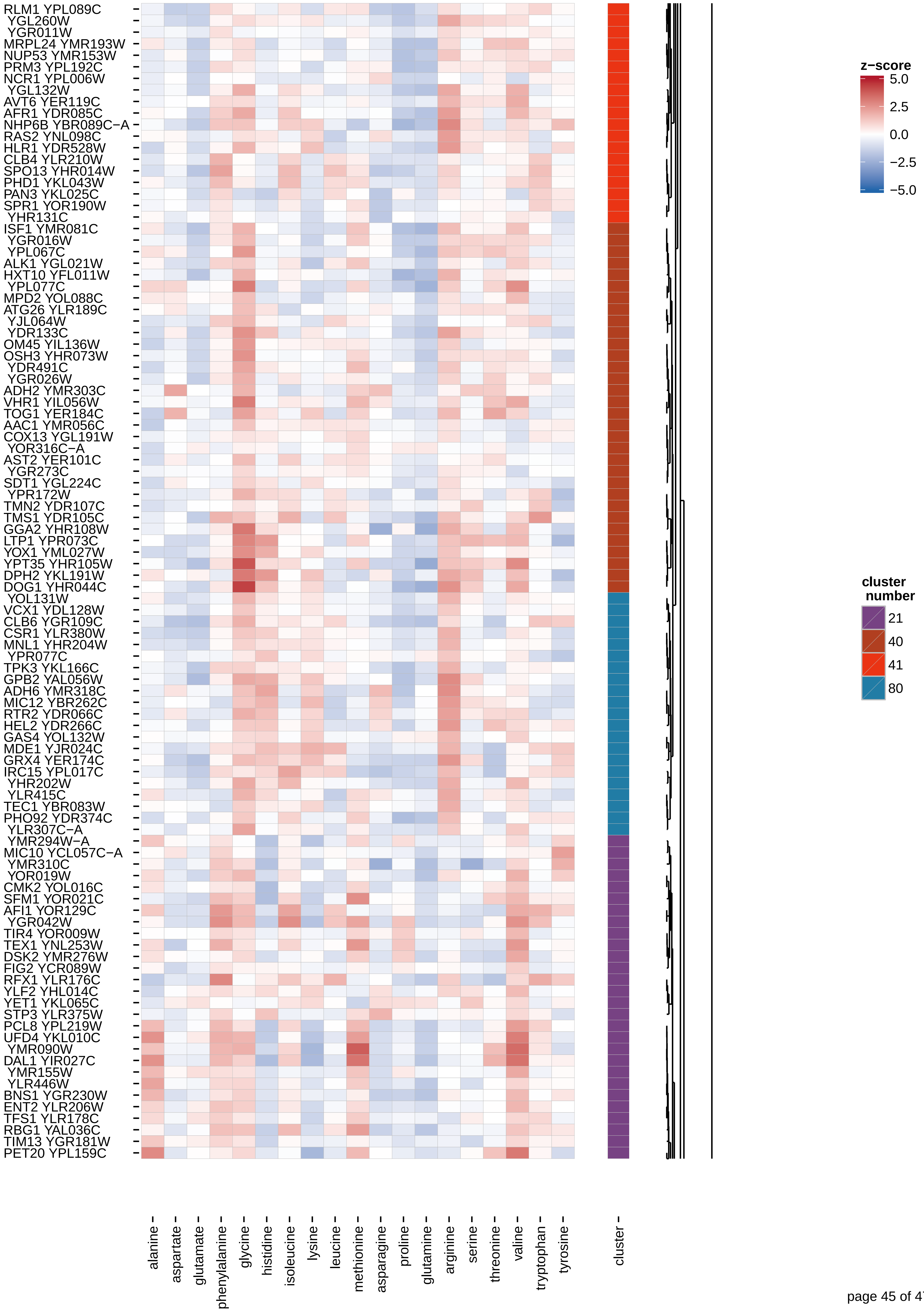
RLM1 YPL089C
YGL260W
YGR011W
MRPL24 YMR193W
NUP53 YMR153W
PRM3 YPL192C
NCR1 YPL006W
YGL132W
AVT6 YER119C
AFR1 YDR085C
NHP6B YBR089C−A
RAS2 YNL098C
HLR1 YDR528W
CLB4 YLR210W
SPO13 YHR014W
PHD1 YKL043W
PAN3 YKL025C
SPR1 YOR190W
YHR131C
ISF1 YMR081C
YGR016W
YPL067C
ALK1 YGL021W
HXT10 YFL011W
YPL077C
MPD2 YOL088C
ATG26 YLR189C
YJL064W
YDR133C
OM45 YIL136W
OSH3 YHR073W
YDR491C
YGR026W
ADH2 YMR303C
VHR1 YIL056W
TOG1 YER184C
AAC1 YMR056C
COX13 YGL191W
YOR316C−A
AST2 YER101C
YGR273C
SDT1 YGL224C
YPR172W
TMN2 YDR107C
TMS1 YDR105C
GGA2 YHR108W
LTP1 YPR073C
YOX1 YML027W
YPT35 YHR105W
DPH2 YKL191W
DOG1 YHR044C
YOL131W
VCX1 YDL128W
CLB6 YGR109C
CSR1 YLR380W
MNL1 YHR204W
YPR077C
TPK3 YKL166C
GPB2 YAL056W
ADH6 YMR318C
MIC12 YBR262C
RTR2 YDR066C
HEL2 YDR266C
GAS4 YOL132W
MDE1 YJR024C
GRX4 YER174C
IRC15 YPL017C
YHR202W
YLR415C
TEC1 YBR083W
PHO92 YDR374C
YLR307C−A
YMR294W−A
MIC10 YCL057C−A
YMR310C
YOR019W
CMK2 YOL016C
SFM1 YOR021C
AFI1 YOR129C
YGR042W
TIR4 YOR009W
TEX1 YNL253W
DSK2 YMR276W
FIG2 YCR089W
RFX1 YLR176C
YLF2 YHL014C
YET1 YKL065C
STP3 YLR375W
PCL8 YPL219W
UFD4 YKL010C
YMR090W
DAL1 YIR027C
YMR155W
YLR446W
BNS1 YGR230W
ENT2 YLR206W
TFS1 YLR178C
RBG1 YAL036C
TIM13 YGR181W
PET20 YPL159C
alanine
aspartate
glutamate
phenylalanine
glycine
histidine
isoleucine
lysine
leucine
methionine
asparagine
proline
glutamine
arginine
serine
threonine
valine
tryptophan
tyrosine
cluster
z−score
5.0
2.5
0.0
−2.5
−5.0
cluster
number
21
40
41
80

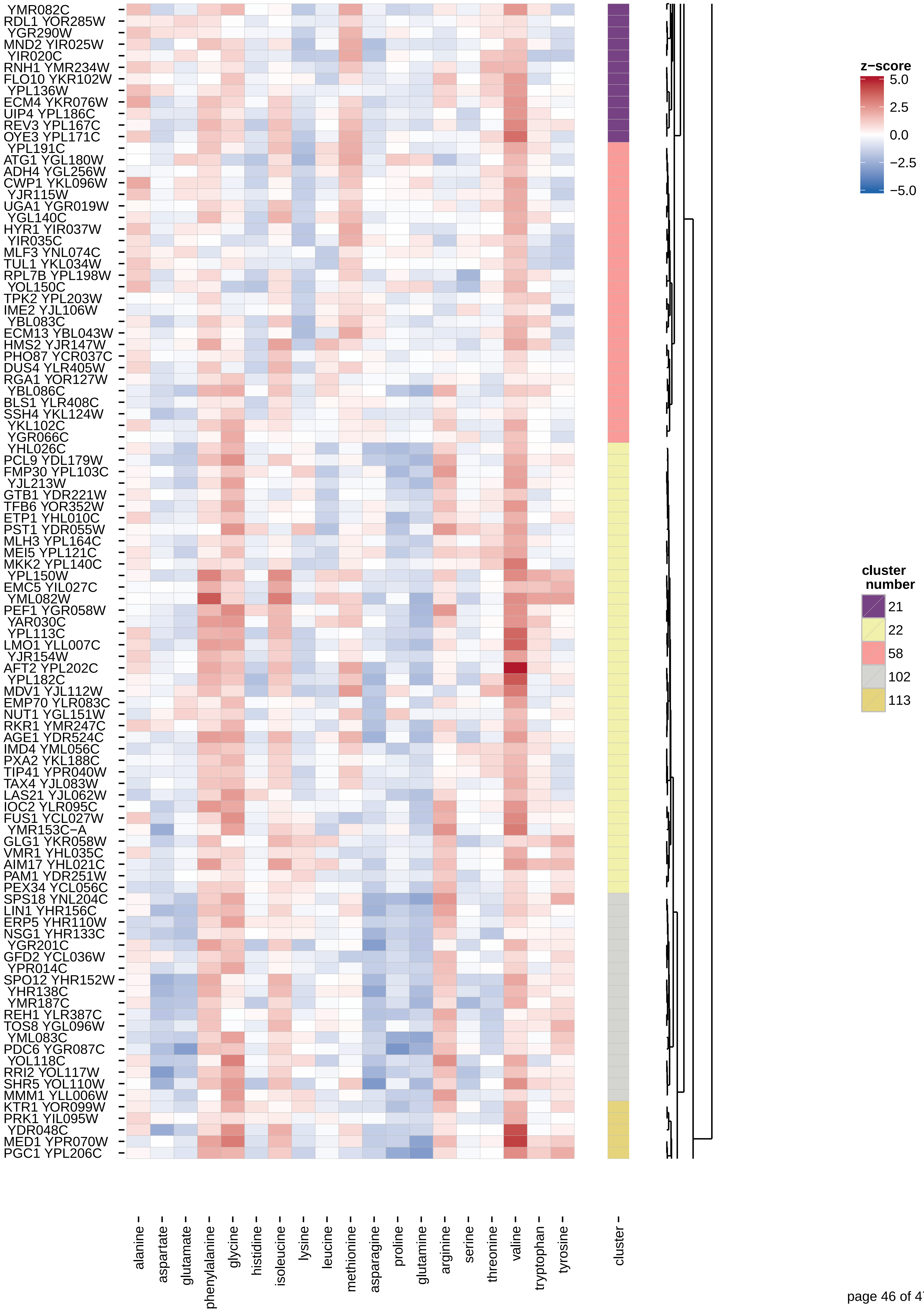
YMR082C
RDL1 YOR285W
YGR290W
MND2 YIR025W
YIR020C
RNH1 YMR234W
FLO10 YKR102W
YPL136W
ECM4 YKR076W
UIP4 YPL186C
REV3 YPL167C
OYE3 YPL171C
YPL191C
ATG1 YGL180W
ADH4 YGL256W
CWP1 YKL096W
YJR115W
UGA1 YGR019W
YGL140C
HYR1 YIR037W
YIR035C
MLF3 YNL074C
TUL1 YKL034W
RPL7B YPL198W
YOL150C
TPK2 YPL203W
IME2 YJL106W
YBL083C
ECM13 YBL043W
HMS2 YJR147W
PHO87 YCR037C
DUS4 YLR405W
RGA1 YOR127W
YBL086C
BLS1 YLR408C
SSH4 YKL124W
YKL102C
YGR066C
YHL026C
PCL9 YDL179W
FMP30 YPL103C
YJL213W
GTB1 YDR221W
TFB6 YOR352W
ETP1 YHL010C
PST1 YDR055W
MLH3 YPL164C
MEI5 YPL121C
MKK2 YPL140C
YPL150W
EMC5 YIL027C
YML082W
PEF1 YGR058W
YAR030C
YPL113C
LMO1 YLL007C
YJR154W
AFT2 YPL202C
YPL182C
MDV1 YJL112W
EMP70 YLR083C
NUT1 YGL151W
RKR1 YMR247C
AGE1 YDR524C
IMD4 YML056C
PXA2 YKL188C
TIP41 YPR040W
TAX4 YJL083W
LAS21 YJL062W
IOC2 YLR095C
FUS1 YCL027W
YMR153C−A
GLG1 YKR058W
VMR1 YHL035C
AIM17 YHL021C
PAM1 YDR251W
PEX34 YCL056C
SPS18 YNL204C
LIN1 YHR156C
ERP5 YHR110W
NSG1 YHR133C
YGR201C
GFD2 YCL036W
YPR014C
SPO12 YHR152W
YHR138C
YMR187C
REH1 YLR387C
TOS8 YGL096W
YML083C
PDC6 YGR087C
YOL118C
RRI2 YOL117W
SHR5 YOL110W
MMM1 YLL006W
KTR1 YOR099W
PRK1 YIL095W
YDR048C
MED1 YPR070W
PGC1 YPL206C
alanine
aspartate
glutamate
phenylalanine
glycine
histidine
isoleucine
lysine
leucine
methionine
asparagine
proline
glutamine
arginine
serine
threonine
valine
tryptophan
tyrosine
cluster
z−score
5.0
2.5
0.0
−2.5
−5.0
cluster
number
21
22
58
102
113

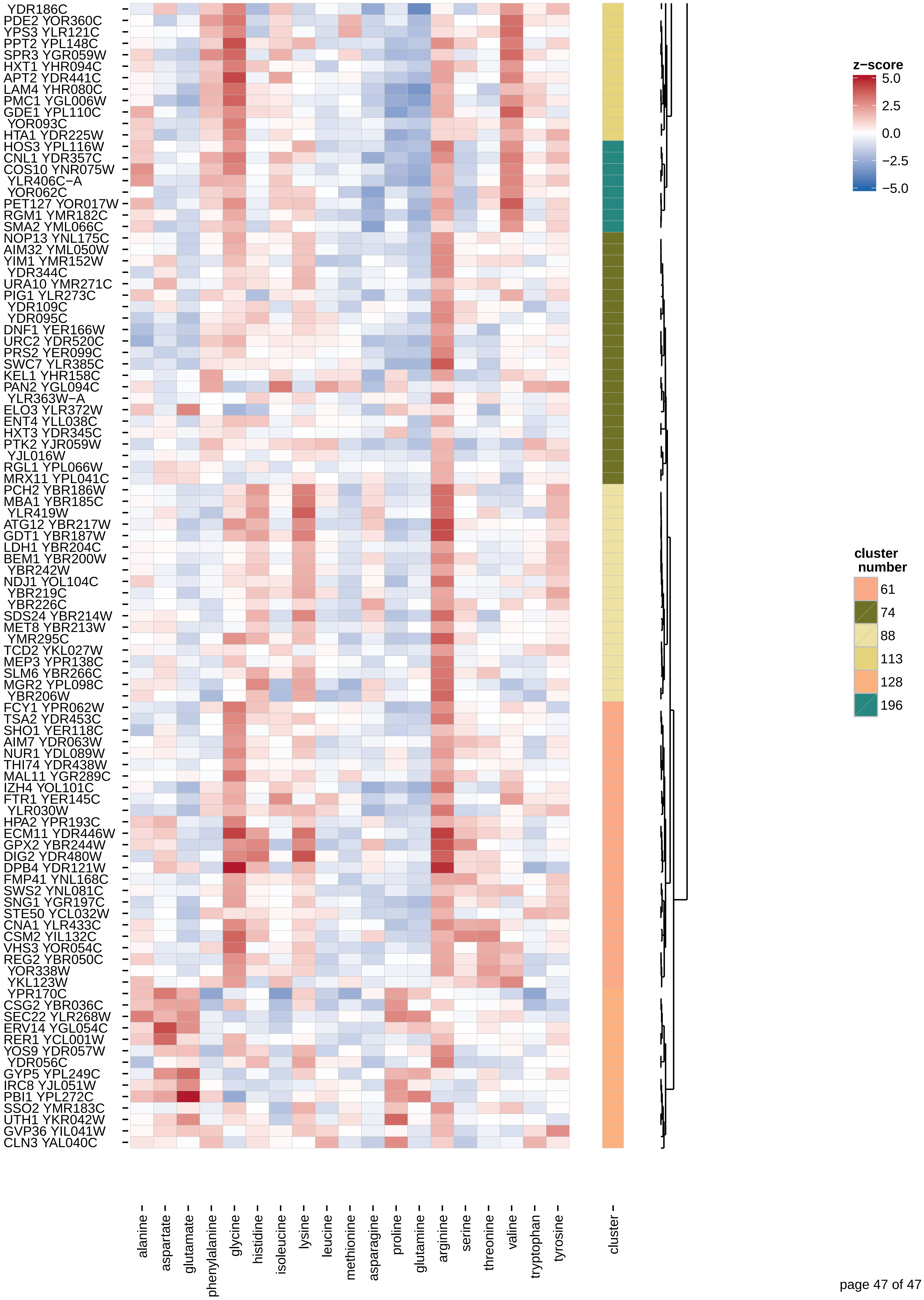

YDR186C
PDE2 YOR360C
YPS3 YLR121C
PPT2 YPL148C
SPR3 YGR059W
HXT1 YHR094C
APT2 YDR441C
LAM4 YHR080C
PMC1 YGL006W
GDE1 YPL110C
YOR093C
HTA1 YDR225W
HOS3 YPL116W
CNL1 YDR357C
COS10 YNR075W
YLR406C−A
YOR062C
PET127 YOR017W
RGM1 YMR182C
SMA2 YML066C
NOP13 YNL175C
AIM32 YML050W
YIM1 YMR152W
YDR344C
URA10 YMR271C
PIG1 YLR273C
YDR109C
YDR095C
DNF1 YER166W
URC2 YDR520C
PRS2 YER099C
SWC7 YLR385C
KEL1 YHR158C
PAN2 YGL094C
YLR363W−A
ELO3 YLR372W
ENT4 YLL038C
HXT3 YDR345C
PTK2 YJR059W
YJL016W
RGL1 YPL066W
MRX11 YPL041C
PCH2 YBR186W
MBA1 YBR185C
YLR419W
ATG12 YBR217W
GDT1 YBR187W
LDH1 YBR204C
BEM1 YBR200W
YBR242W
NDJ1 YOL104C
YBR219C
YBR226C
SDS24 YBR214W
MET8 YBR213W
YMR295C
TCD2 YKL027W
MEP3 YPR138C
SLM6 YBR266C
MGR2 YPL098C
YBR206W
FCY1 YPR062W
TSA2 YDR453C
SHO1 YER118C
AIM7 YDR063W
NUR1 YDL089W
THI74 YDR438W
MAL11 YGR289C
IZH4 YOL101C
FTR1 YER145C
YLR030W
HPA2 YPR193C
ECM11 YDR446W
GPX2 YBR244W
DIG2 YDR480W
DPB4 YDR121W
FMP41 YNL168C
SWS2 YNL081C
SNG1 YGR197C
STE50 YCL032W
CNA1 YLR433C
CSM2 YIL132C
VHS3 YOR054C
REG2 YBR050C
YOR338W
YKL123W
YPR170C
CSG2 YBR036C
SEC22 YLR268W
ERV14 YGL054C
RER1 YCL001W
YOS9 YDR057W
YDR056C
GYP5 YPL249C
IRC8 YJL051W
PBI1 YPL272C
SSO2 YMR183C
UTH1 YKR042W
GVP36 YIL041W
CLN3 YAL040C
alanine
aspartate
glutamate
phenylalanine
glycine
histidine
isoleucine
lysine
leucine
methionine
asparagine
proline
glutamine
arginine
serine
threonine
valine
tryptophan
tyrosine
cluster
z−score
5.0
2.5
0.0
−2.5
−5.0
cluster number
61
74
88
113
128
196
